# Bro̷nsted Acid-Catalyzed Reduction of Furans

**DOI:** 10.1021/jacs.4c18485

**Published:** 2025-02-19

**Authors:** Nils Frank, Markus Leutzsch, Benjamin List

**Affiliations:** Max-Planck-Institut für Kohlenforschung, Mülheim an der Ruhr 45470, Germany

## Abstract

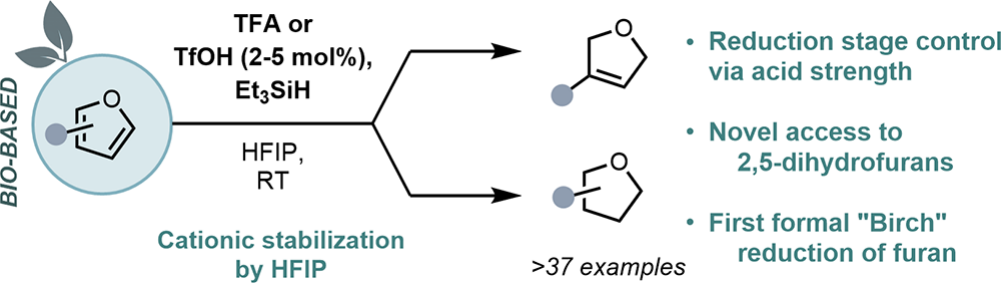

Bioderived furans play a pivotal role in advancing defossilized chemical pathways. The complete reduction of furans currently relies on impractical metal-catalyzed hydrogenations at high pressures and temperatures. In addition, the Birch reduction of unbiased furans to 2,5-dihydrofurans remains an unsolved synthetic challenge. Herein, we report a mild Bro̷nsted acid-catalyzed reduction of furans to 2,5-dihydro- and/or tetrahydrofuran derivatives using silanes as reducing agents. In particular, the first formal Birch reduction of furan itself is achieved. Mechanistic investigations reveal an intricate behavior of HFIP as the crucial solvent, preventing the intrinsic polymerization behavior of furans under acidic conditions and introducing additional driving force by specific product binding.

## 1 Introduction

**Figure 1:**
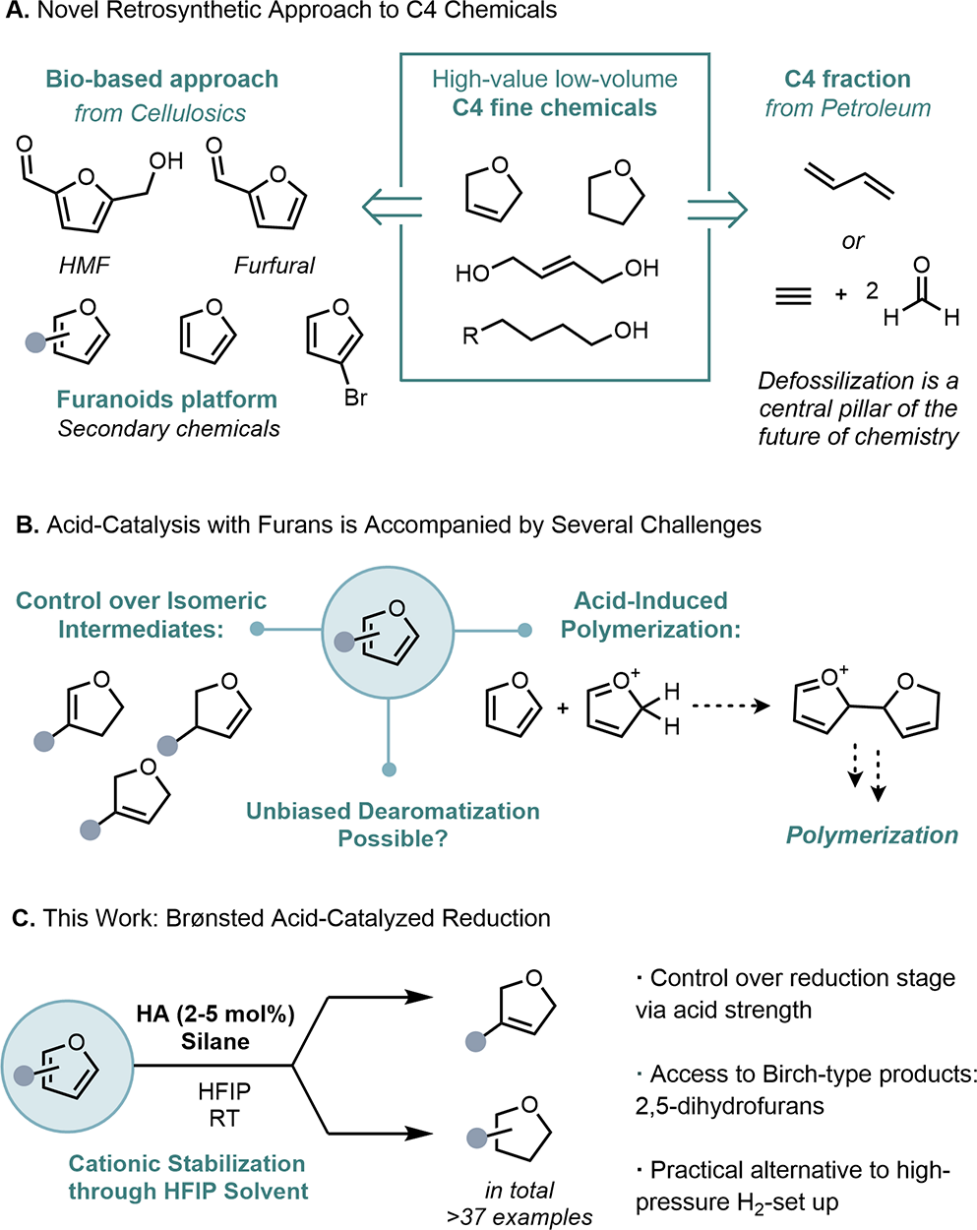
(A) Furan as a biobased source toward fine chemicals. (B) Unmet challenges of furan chemistry. (C) This work: Bro̷nsted acid-catalyzed reduction of furans.

Furans are viewed as critical for transforming the fossil-based chemical industry to biobased and sustainable production pathways of fine chemicals.^1,2^ For example, furfural and 5-hydroxymethylfurfural are accessed from lignocellulosic carbohydrates,^2^ from which more elaborate secondary furanoids, such as 3-bromofuran, can be obtained. This portfolio of biobased platform chemicals challenges chemists to innovate routes toward low-volume, high-value fine chemicals (Figure 1A).^3^ Despite its importance, furan-based chemistry remains significantly underdeveloped relative to its potential future impact. For example, general and site-selective control over the desaturation degree of furans toward dihydrofurans could enable novel, highly sought-after valorization strategies within biorefinery channels, such as for the fragrance industry (Figure 1B).^4,5^

Although homogeneous and heterogeneous metal-catalyzed reductions from furans to tetrahydrofurans employing elevated H_2_ pressures and specialized Rh-, Ir- or Ru-catalysts are well-established,^6−8^ partial dearomatization strategies of the furan core are scarce. Most prominently, Birch reductions of furans to dihydrofurans are limited to a handful of electron-poor, carboxylate-substituted examples.^6,9−13^ All other furans, including furan itself, suffer from uncontrolled dimerization and ring-openings, presumably due to their low resonance energy, high reduction potentials, and (vinylogous) enolether character.^14^

We hypothesized that Brønsted acid-catalysis might overcome these challenges by making furans susceptible to hydride attack after protonation, inverting the order of a classical Birch reduction.^15^ Here, we report the mild Bro̷nsted acid-catalyzed reduction of a panoply of furans to the formal Birch-reduced products 2,5-dihydrofurans and, furthermore, full reduction to tetrahydrofuran derivatives using silanes as reducing agents.

## 2 Results and Discussion

**Figure 2:**
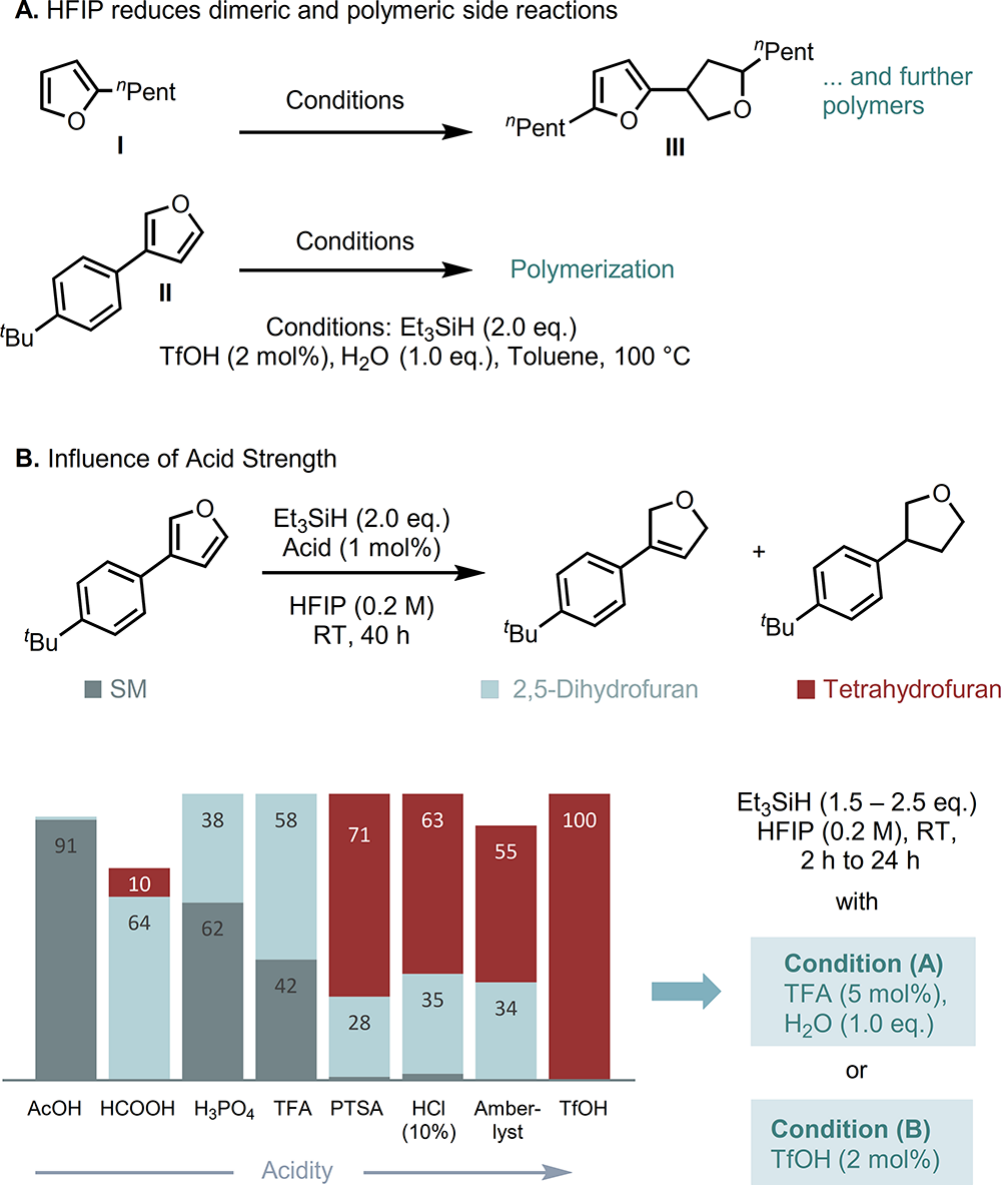
(A) HFIP reduces the polymerization reactivity in commonly employed solvents. (B) Screening of acids identified two sets of conditions to achieve partial reduction to dihydrofuran and complete reduction to tetrahydrofuran. Yields determined by ^1^H NMR spectroscopy relative to mesitylene as internal standard.

Our design is accompanied by two main challenges: controlling the access of isomeric intermediates can be difficult; moreover, furans are well-known to readily polymerize under acidic conditions (Figure 1B).^16^ Since acid-catalyzed reductions of furans are not known,^17−20^ we investigated if a catalytic Bro̷nsted acid (HA) would mitigate polymerization side reactions. The model substrates 2-pentyl furan **I** or 3-aryl furan **II** with triethylsilane (Et_3_SiH) were subjected to catalytic trifluoroacetic acid (TFA) or triflic acid (TfOH) along with a proton source (1.0 equiv), such as water (H_2_O) or phenol, across different solvents and temperatures (Figure 2A).

Generally, either no reactivity or dimeric products **III** accompanied by polymeric material (at 50–100 °C) were obtained (Figure 2A). To circumvent this, we hypothesized that the unique non-nucleophilic properties of perfluorinated alcohols might enable sufficient stabilization of cationic intermediates without participating in solvolysis.^21−24^ Indeed, upon employing hexafluoro-2-propanol (HFIP), productive reduction of **I** and **II** toward the 2,5-dihydrofuran and/or tetrahydrofuran at room temperature was observed with minimal polymerization degrees (0–10%). The addition of H_2_O (1.0 equiv) was crucial to achieve complete conversion, although the use of H_2_O as a solvent led to no conversion.

The reduction stage of **II** was found to be controllable by acid strength (Figure 2B). The use of phosphoric acid or TFA allowed clean conversion toward 2,5-dihydrofurans without overreduction. Notably, this selectivity was only achieved under catalytic acid regime; increasing the acid to 1.0 equiv led to complete reduction to tetrahydrofurans. Stronger acids, such as *p*-toluenesulfonic acid (PTSA) or aqueous hydrochloric acid (HCl), lead to parallel overreduction to a mixture of dihydro- and tetrahydrofurans (THF). For triflic acid (TfOH), only THF products were observed. In general, the degree of reduction was not efficiently controlled by the stoichiometry of the silane.

Based on these screening results, two sets of reaction conditions were selected to probe their general applicabilities to furan substitution and electronic character: (A) TFA (5 mol %), Et_3_SiH (1.5 equiv), H_2_O (1.0 equiv), and (B) TfOH (2 mol %), Et_3_SiH (2.5 equiv), both in HFIP (0.2 M) at room temperature.

**Figure 3:**
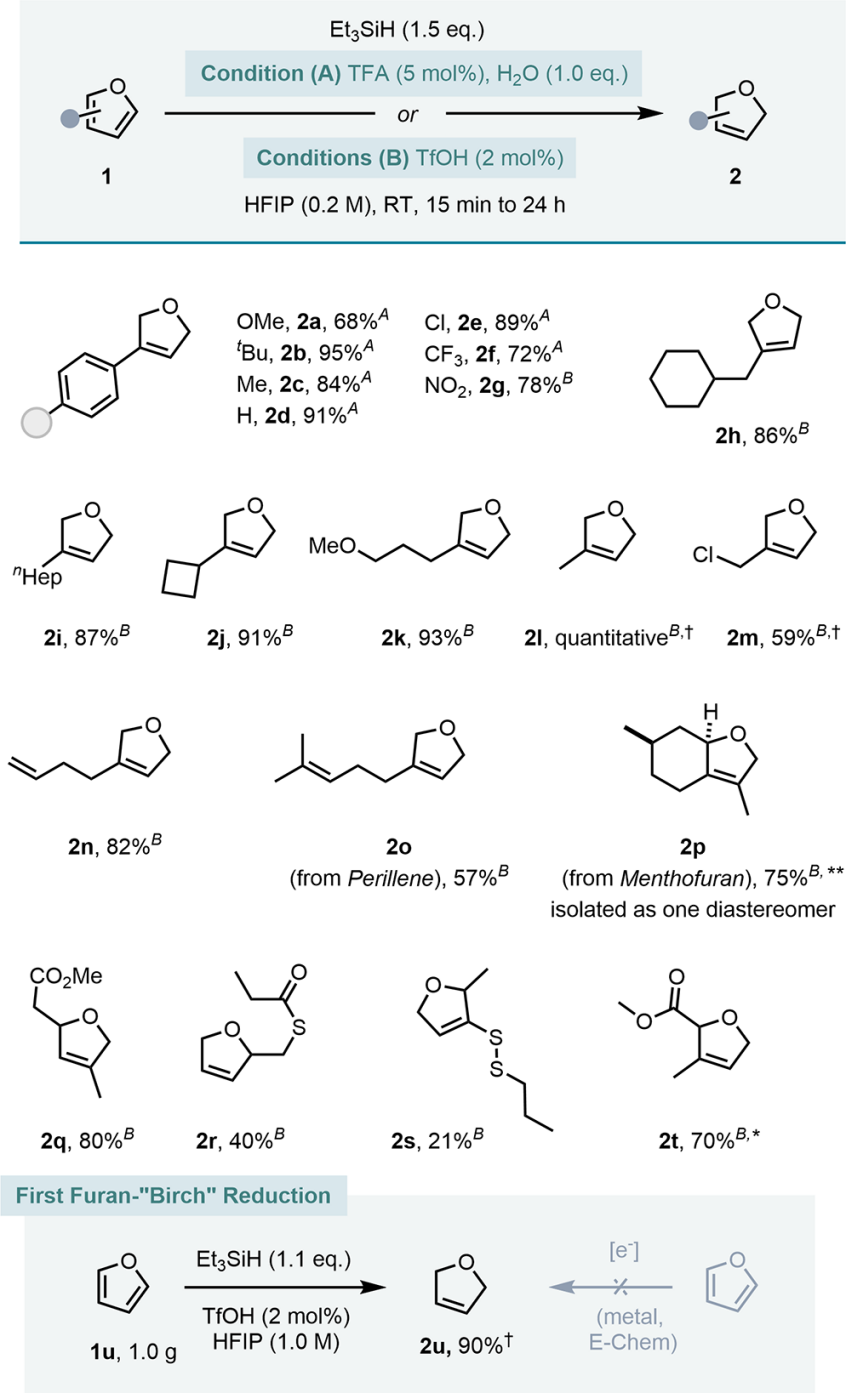
Substrate scope toward 2,5-dihydrofurans with superscripts A and B indicate Conditions (A) and (B). Reactions were performed on a 0.2–30.0 mmol scale. Yields are reported as isolated yields after column chromatography, except † indicates ^1^H NMR yields. *Incomplete conversion achieved: addition of further portions of Et_3_SiH. **5 mol% TfOH. See the Supporting Information for detailed reaction conditions.

Upon employing Condition (A), a wide range of 3-aryl substituted furans **1a**–**1g** can be selectively converted to 2,5-dihydrofurans **2a**–**2g** in good to excellent yields (68–95%, Figure 3). Variations of the electronic nature of the 3-aryl substituent were well tolerated, spanning the whole Hammett *σ*_*p*_-scale,^25^ albeit the stronger acidic Condition (B) was required to obtain the electron-poor nitro-derivate **2g** successfully. Similarly, 3-alkyl substituted furans **1h**–**1l** were converted in near-quantitative yields to 2,5-dihydrofurans **2h**–**2l** by employing Condition (B). Surprisingly, chloromethylene (**2m**, 59%) and terminal olefins (**2n**, 82%) were tolerated, as being commonly reduced in ionic silane reductions.^26^ As a result, the partial reduction of Perillene **1o**, a natural monoterpene in the essential oil of *perilla frutescens*, to 2,5-dihydroperillene **2o** in 57% yield was possible, leaving the homoprenyl-side chain untouched. Furthermore, conversion of the flavoring agent menthofuran^27^**1p** was feasible in 75%. The 2,4-substituted furan **1q** was cleanly reduced to **2q**. Further, **1r** can be coverted to **2r** in 40% yield, tolerating (thio-)esters.

Limitations of the methodology were shown by meat-flavoring supplement^28^**1s** yielding only 21% 2,5-dihydrofuran **2s**, presumably being too electron-rich and prone toward reductive disulfide cleavage. For electron-poor furans, only ester **1t** was successfully converted to the 2,5-dihydrofuran. Substrates lacking the stabilizing methyl group, such as 2-carboxyl furans or 3-carboxyl furans, remain unreacted, rendering the methodology complementary to the existing dissolving-metal reduction of electron-deficient furanoic acids.^6^

To our delight, furan itself (**1u**) proved to be a suitable substrate and was cleanly converted to 2,5-dihydrofuran (**2u**) in quantitative yield on a 1.0 g scale. This constitutes the first reduction of plain furan to a “Birch”-type product.

**Figure 4:**
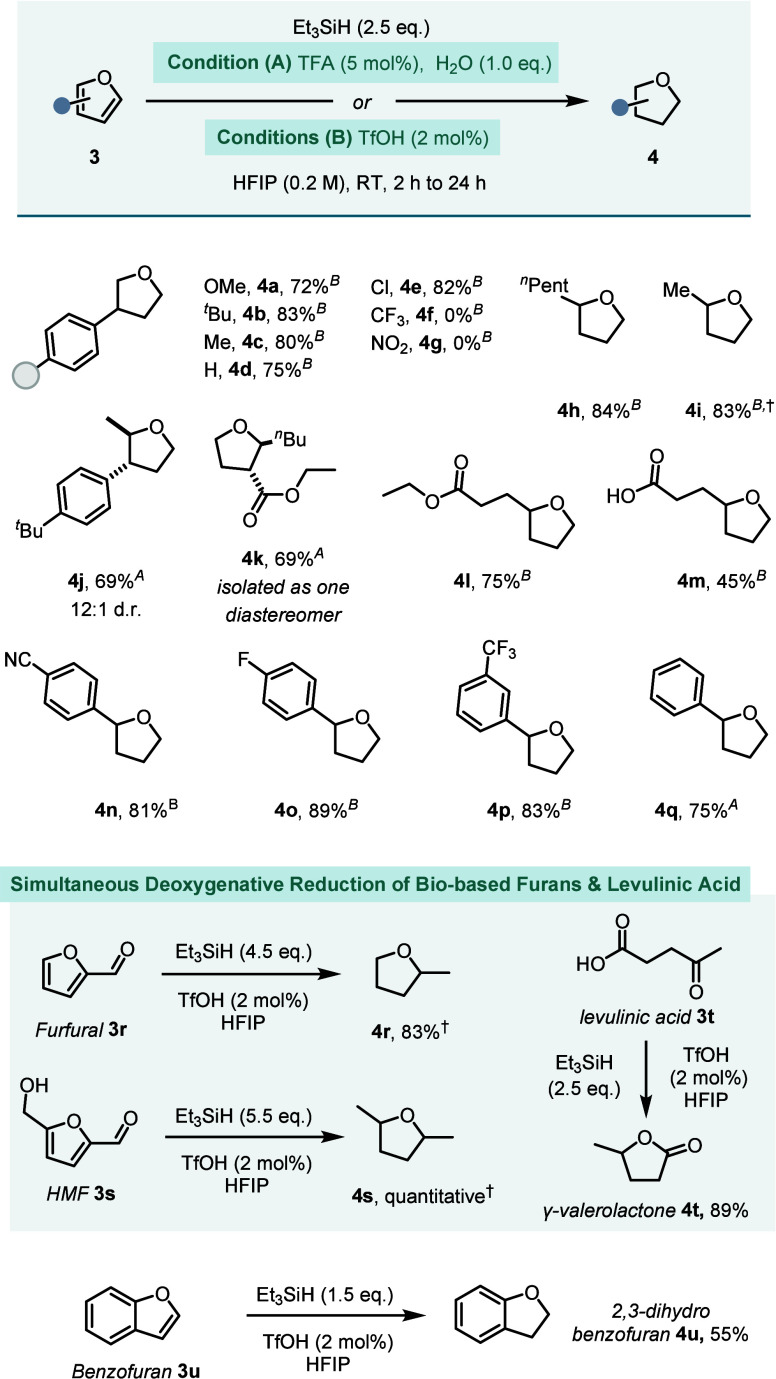
Substrate scope toward tetrahydrofurans with superscripts A and B indicating Conditions (A) and (B). Reactions performed on 0.1–7.2 mmol scale. Yields are reported as isolated yields after column chromatography, except † indicates ^1^H NMR yields. See the Supporting Information for detailed conditions.

Condition (B) was employed to fully reduce the 3-aryl furans to their respective THF derivatives **4a**–**4e** in good to very good yields (72–83%, Figure 4). This example showcases how selective control over the reduction stage via acid strength is possible under Conditions (A) and (B). Electron-deficient substrates **3f** and **3g** could not be reduced further than their 2,5-dihydro analogs. In contrast, 2-substituted furans, such as **3h**–**3m**, could not be selectively reduced to a dihydrofuran and instead consistently delivered their tetrahydrofuran product in high *trans*-diastereoselectivities (**4j**, **4k**). In particular, 2-aryl substituted furans were sensitive to the conditions employed. Electron-deficient **3n**–**3p** could readily be reduced using Condition (B), whereas 2-phenyl furan **3q** required Condition (A) to avoid side reactivity. The proton-affinity of the respective dihydrofuran intermediate is likely to be responsible for the susceptibility to different acid strengths (*vide infra*, Figure 6).

Interestingly, the highly acidic Condition (B) enables the total reduction and deoxygenation of biobased substrates, avoiding the use of commonly employed precious metal catalysts and high H_2_ pressures. For example, furfural **3r** and 4-hydroxymethyl furfural (HMF) **3s** were converted to methyl-THF **4r** (83%) and 2,4-dimethyl-THF **4s** (quantitative). Furthermore, the reductive cyclization of levulinic acid **3t** to γ-valerolactone **4t** was possible in 89% yield. Besides, benzofuran **3u** was successfully converted to 2,3-dihydrobenzofuran **4u** in 55% yield.

**Figure 5:**
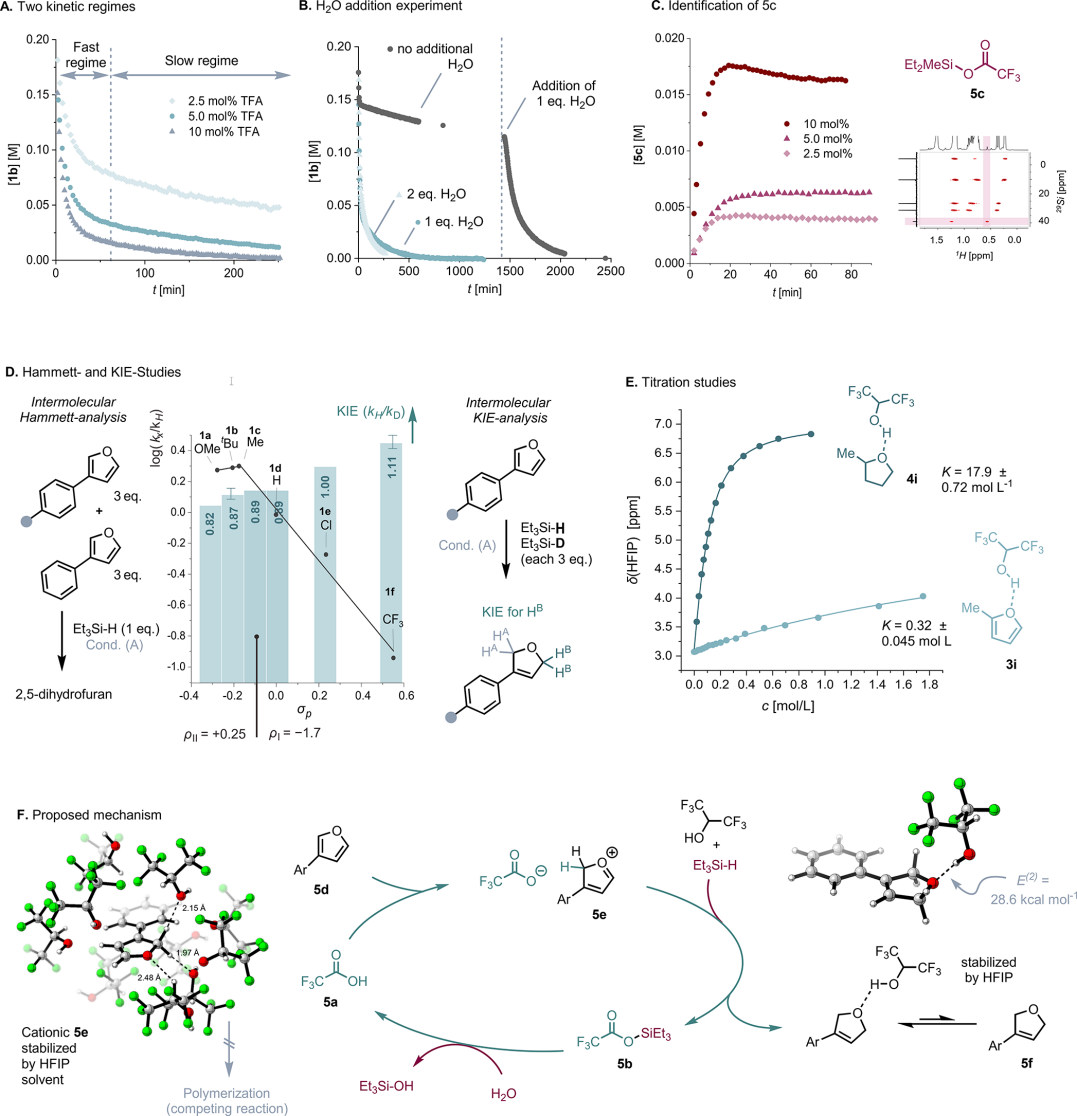
(A) Concentration profile of different catalyst concentrations for the reduction of **1b** under Condition (B) was monitored by No-D ^1^H NMR. (B) Concentration profile of varying water amounts and delayed water addition to the reaction mixture. (C) Identification of the silylated catalyst employing **1b** under Condition (B) with Et_2_MeSiH instead of Et_3_SiH. (D) Combined representation of Hammett- and KIE-analysis: The line diagram depicts the Hammett slope of substrates **1a**–**1f** in an intermolecular competition experiment with **1d**; the bar diagram shows the obtained KIE in intermolecular competition experiments between Et_3_Si–H and Et_3_Si–D. (E) Binding isotherm of 2-MeTHF and 2-methylfuran and HFIP at [HFIP] = 0.1 M host concentration and derived 1:1 binding constant *K*. See the Supporting Information for further details. (F) Proposed mechanism in combination with computed structures at CPCM(HFIP)-B2PLYP-D3BJ/def2-SVP (298 K/1 M) level of theory. *E*^*(2)*^ value stems from second order perturbation theory analysis of fock matrix in NBO basis. HFIP-solvation cloud was generated with ORCA 6.0-*Docker* at GFN2-xTB level of theory. See the Supporting Information for further details.

To understand the observed selectivities, the reaction of **1b** under Condition (A) was followed via No-D ^1^H NMR spectroscopy, varying the amounts of each reagent. First, the reaction kinetics was not significantly influenced by the equivalents of Et_3_SiH used, indicating a zeroth-order dependence on Et_3_SiH (see Supporting Information). Second, TFA-catalyst loading was investigated, and two regimes in the kinetic time course could be identified (Figure 5A). The fast consumption of furan **1b** in the first few minutes shows a clear dependency on the catalyst loading, where, as expected, higher catalyst loading leads to faster consumption. The profile then changes to a relatively linear decay. Lastly, the H_2_O equivalents were varied. Without H_2_O, the reaction stagnates at approximately 25% conversion; upon addition of 1.0 equiv H_2_O at *t* = 24 h, the conversion resumes, proceeding as previously observed (see Figure 5B).

Therefore, we hypothesized that catalyst **5a** is silylated to **5b** and is hydrolytically regenerated by H_2_O, as the HFIP solvent lacks sufficient nucleophilicity to efficiently drive the solvolysis.^21^ The hydrolysis may regulate the kinetics in the second phase and attenuate the initial reaction rate. To probe this, Et_3_SiH was exchanged for Et_2_MeSiH at different concentrations, and indeed, the formation of the silylated catalyst Et_2_MeSi-TFA **5c** could be monitored by its indicative Me-singlet assigned via ^1^H–^29^Si HMBC (Figure 5C).

An intermolecular competition experiment of substrates **1a**–**1f** relative to **1d** yielded a log(*k*_*x*_/*k*_*H*_) graph based on the relative ^1^H NMR integration of products. A nonlinear, concave, downward-shaped Hammett plot was obtained (Figure 5D, line chart), suggesting a change in the rate-limiting step for **1a**–**1f** under the same general mechanism.^25,29,30^ More specifically, for the electron-poor furans **1e** and **1f**, a negative reaction rate constant *ρ*_*I*_ = −1.7 was found, consistent with furan protonation being involved in the rate-determining step. The electron-rich substrates **1a**–**1c** form a slightly positive Hammett-slope of *ρ*_*II*_ = +0.25, associated with a negative charge build up, which would be in line with the reduction becoming the rate-limiting step.^29^

To investigate this reduction step further, the kinetic isotope effects (KIEs) of an equimolar intermolecular competition experiment between Et_3_Si–H and Et_3_Si–D with limiting amount of substrates **1a**–**1f** were determined (Figure 5D, bar diagram, see Supporting Information for further details). A positive KIE of 1.11 for **1f** was found, whereas a secondary inverse KIE of up to 0.82 was obtained for the electron-rich substrates **1a**–**1c**.^31,32^ Taking the obtained concave Hammett data into consideration, this suggests that the hydridic reduction of carbocation **5e** is the rate-determining step for electron-rich substrates, whereas for electron-poor substrates, it is the protonation of **5d**.^33^

To gain insight into the role of HFIP as a crucial solvent, an EXSY-NMR of **1a** with TFA (1.0 equiv) was measured. No direct TFA furan ion-pair was detected, instead there was a protic exchange between HFIP and H^A^ of furan **1a** (see Supporting Information for further details). This hints that HFIP functions as a reliable cationic-stabilizing solvent, inhibiting the dimerization and polymerization as observed in conventional solvents (Figure 2A) without interfering solvolytically as a nucleophile in the reaction. Implicit solvent modeling confirms the presence of multiple H-bonds between the HFIP solvent cage and protonated furan **5e** (Figure 5F).^34^

Further, titration studies (Figure 5E) show high 1:1 binding affinities of HFIP to dearomatized intermediates (e.g., **4i**: *K* = 17.9 ± 0.72 mol L^–1^) and negligible complexation constants for furans itself (e.g., **3i**: *K* = 0.32 ± 0.045 mol L^–1^). This was corroborated by computational analysis, locating the complexes between HFIP for **5f**. Natural bonding orbital (NBO) analysis confirms the presence of a strong H-bond between HFIP and **5f** (*E*^*(2)*^ = 28.6 kcal mol^–1^). This HFIP-complexation of ethereal oxygen adds additional driving force to the reduction reaction.

Collectively, these results support the catalytic cycle depicted in Figure 5F for 3-substituted furans. Upon protonation of the furan **5d**, the oxocarbenium cation **5e** is reduced by Et_3_SiH at the 2-position, resulting in a 2,5-dihydrofuran product **5f**. The catalytic trifluoroacetate anion recombines with the remaining silylium cation to form the stable intermediate **5b**. Subsequently, it is regenerated through a hydrodesilylation with H_2_O to the active catalyst TFA. This step becomes rate-limiting after the first induction period decelerating the reaction kinetics.

**Figure 6:**
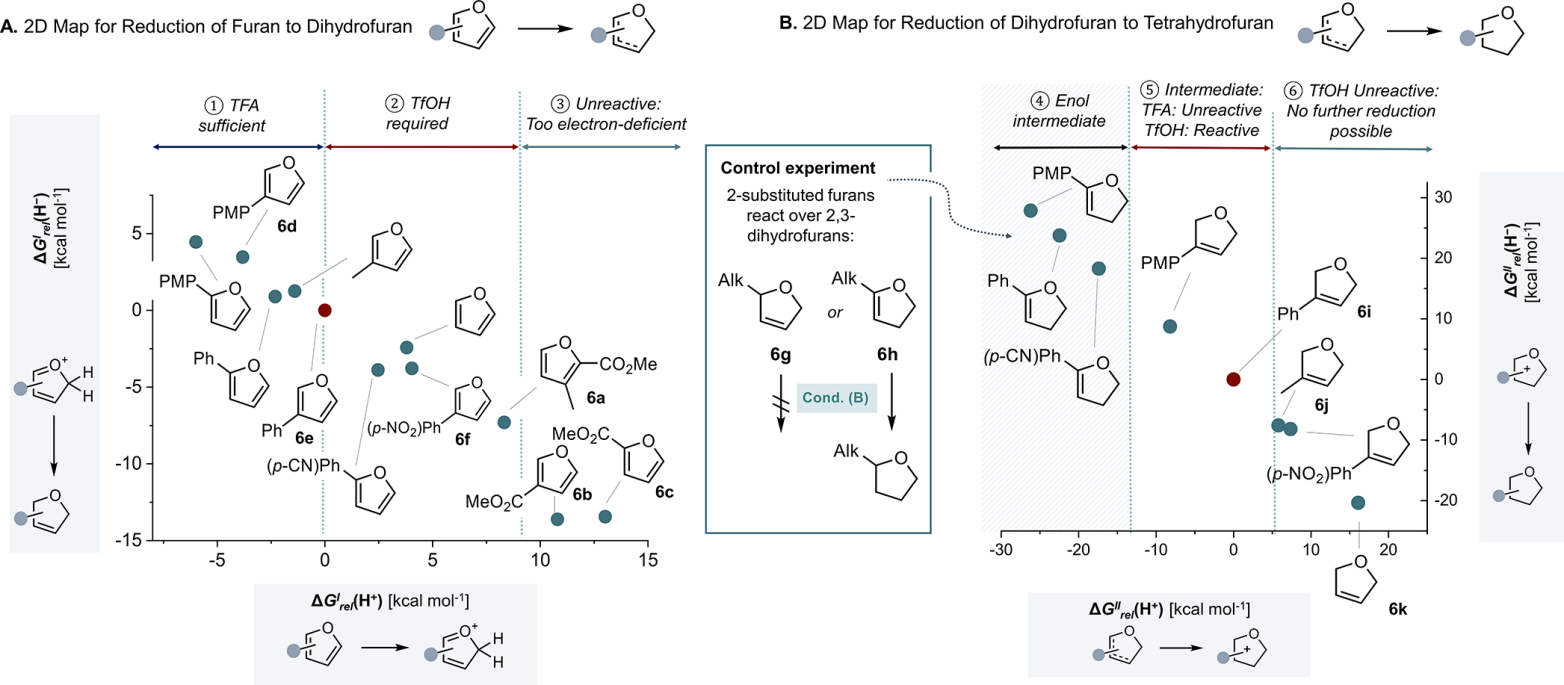
2D maps plotting Δ*G*_*rel*_(H^+^) and Δ*G*_*rel*_(H^–^) for the reduction of (A) furan to dihydrofuran and (B) dihydrofuran to tetrahydrofuran at CPCM(HFIP)-DLPNO–CCSD(T)/def2-TZVPP//CPCM(HFIP)-B2PLYP-D3BJ/def2-SVP (298 K/1 M) level of theory. See the Supporting Information for details. PMP = *para*-methoxyphenyl.

To put the found conditions in a broader context of furan reactivity, a 2D plot was constructed to rationalize the observed reactivity as described above. Computed Gibbs free energies Δ*G*_*rel*_(H^+^) of the protonation of furan were plotted on the *x*-axis, and on the *y*-axis were plotted the Δ*G*_*rel*_(H^–^) of the reduction of the protonated intermediate toward dihydrofuran *relative* to the chosen reference 3-phenyl furan **6e** (Δ*G*^I^_rel_(H^+^) = 0; Δ*G*^*I*^_*rel*_(H^–^) = 0; Figure 6A). A similar map was constructed from dihydrofuran to tetrahydrofuran with 3-phenyl-2,5-dihydrofuran **6i** chosen as the reference (Δ*G*^*II*^_*rel*_(H^+^) = 0; Δ*G*^*II*^_*rel*_(H^–^) = 0; Figure 6B). Both maps are based on computations at the DLPNO–CCSD(T)/def2-TZVPP//B2PLYP-D3BJ/def2-SVP (298 K/1 M) level of theory.

For each step, all possible protonation and reduction sites were computationally considered, and the energetically lowest-lying isomer was selected. The maps can be divided into segments for different selectivities based on the experimental results (Figures 3 and 4). For example, in Figure 6A, **6a** marks the marginal example of segment ②, which TfOH can still productively protonate; for **6b** or **6c** in segment ③, a stronger acid than TfOH would have been needed for reactivity. Similarly, the plot shows the substrate set **6d** to **6f**, spanning a range of ΔΔ*G*^*I*^_*rel*_(H^+^) = 7.8 kcal mol^–1^, where substrate **6d** is sufficiently protonated by TFA (segment ①), whereas for substrate **6f** TfOH is needed.

Moreover, we sought to explain why 2-substituted furans could not be halted at the partially reduced dihydrofuran. Subjecting a potential 2,5-dihydro intermediate **6g** to Condition (B) yielded no product, whereas the potential 2,3-dihydro intermediate **6h** did. This supports a variation of the mechanism depicted in Figure 5F for 2-substituted furans, potentially proceeding via a 2,3-dihydro instead of a 2,5-dihydro intermediate. Additionally, the enol 2,3-dihydro intermediate **6h** was found to be thermodynamically more stable by 4.3 kcal mol^–1^ and hence selected as the preferred intermediate for the 2D map (Figure 6B). Their high relative proton-affinities Δ*G*^*II*^_*rel*_(H^+^) explain why 2-substituted furans in segment ④ always reduce to tetrahydrofuran, as the second protonation is always preferred (|Δ*G*^*I*^_*rel*_(H^+^)| < |Δ*G*^*II*^_*rel*_(H^+^)|).

Relative to **6i**, the protonation of 3-alkyl substituted furans **6j**, as well as plain 2,5-dihydrofuran **6k** in segment ⑥, is too disfavored to achieve further conversion toward tetrahydrofuran. All these substrates halt at the partially reduced furan. For the intermediate segment ⑤, selectivity control can be achieved by acid strength (see Figure 2).

**Figure 7:**
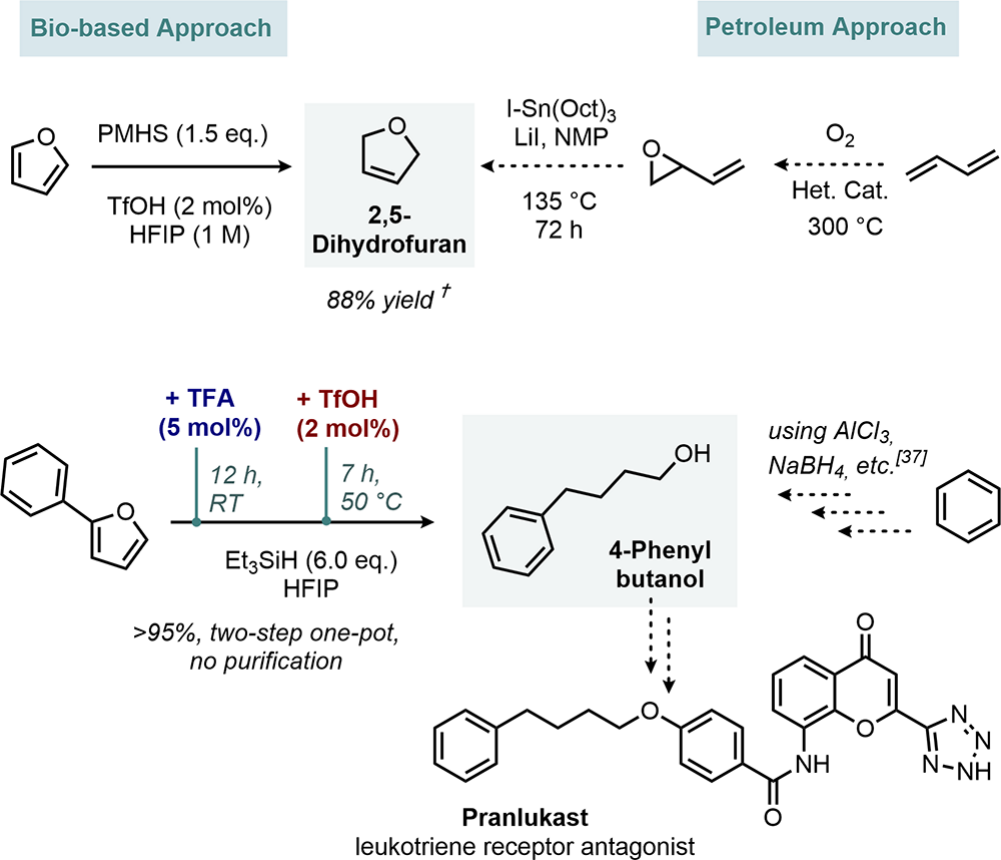
Novel synthetic pathways. † indicates NMR yields.

Finally, we were keen to demonstrate the utility of the dearomatization strategies in potential novel industrial pathways to fine chemicals. For example, 2,5-dihydrofuran is conventionally produced by epoxidation of butadiene and further thermal rearrangement (Figure 7).^35,36^ The direct access from furan might offer novel opportunities in biorefineries if HFIP recycling can be achieved efficiently. To avoid the use of Et_3_SiH, polymethylhydrosiloxane PMHS, a waste product of the silicon industry, was found to be a viable reduction agent for furan too.

When 2-phenyl furan is sequentially treated with TFA and TfOH in an excess of Et_3_SiH, complete opening to 4-phenylbutanol was achieved in quantitative yields without chromatographic purification. So far, this chemical has been produced in multistep sequences, employing stoichiometric amounts of AlCl_3_ and NaBH_4_ for the use as a key intermediate of the leukotriene receptor antagonist Pranlukast (Figure 7).^37,38^

## 3 Summary

In summary, a Bro̷nsted acid-catalyzed reduction of furans to 2,5-dihydro- and/or tetrahydrofuran derivatives using silanes as reducing agents is reported, leveraging the stabilizing effect of the HFIP solvent in preventing polymerization side reactivity. Mechanistic and computational analysis rationalize the selection of suitable acid strength, enabling control over the reduction degree for 3-aryl substituted furans. Further, a broad scope of unbiased furans was reduced in a substrate-controlled way to either their 2,5-dihydrofuran or tetrahydrofuran analogs. In particular, the first formal Birch reduction of furan itself was achieved. We anticipate that the now easy-to-access 2,5-dihydrofuran structures from a wide range of furans enable novel retrosynthetic pathways. Further investigations into the HFIP-effect and an asymmetric variant of the reported reaction are ongoing in our laboratories.

## Supporting Information Available

Experimental and mechanistic details and analytical data for all new compounds (PDF)
Cartesian coordinates (ZIP)

## Abbreviations

TFA: 2,2,2-Trifluoroacetic acid
TfOH: Trifluoromethanesulfonic acid, triflic acid
HFIP: 1,1,1,3,3,3-Hexafluoropropan-2-ol
PTSA: *p*-Toluenesulfonic acid
THF: Tetrahydrofuran
HMF: Hydroxymethyl furfural
KIE: Kinetic isotope effect
NMR: Nuclear magnetic resonance
HMBC: Heteronuclear multiple bond correlation
EXSY: Exchange spectroscopy
Δ*G*: Gibbs free energy
PMHS: Polymethylhydrosiloxane
NBO: Natural Bonding Orbital
HA: Brønsted Acid
