# Supplementary material for: Bro̷nsted Acid-Catalyzed Reduction of Furans
Source: J Am Chem Soc. 2025 Feb 19;147(9):7932–8. doi: 10.1021/jacs.4c18485 (PMC11887439; doi:10.1021/jacs.4c18485)
Supplement: Supplementary file 1 — ja4c18485_si_001.pdf [file ja4c18485_si_001.pdf]

Supporting Information for

Brønsted Acid-Catalyzed Reduction of Furans

Nils Frank, Markus Leutzsch, Benjamin List*

Max-Planck-Institut für Kohlenforschung, Kaiser-Wilhelm-Platz 1, 45470 Mülheim an der Ruhr, Germany
*E-mail: list@kofo.mpg.de

1. Materials and Methods 2
2. Troubleshooting 3
3. General Procedures 4
4. Unsuccessful Substrates 4
5. Identification of Sideproducts 5
6. Optimization 8
7. Mechanistic Studies 9
Deuteration Studies 9
EXSY-NMR Studies 11
Kinetic Isotope Effects 12
Hammett Studies 14
Kinetic Profiling 15
Identification of the Silylated Catalyst 18
NMR Titrations 20
Mechanism for 2-Substituted Furans 23
Catalytic Cycle for the Full Reduction 23
Rationale for trans-Diastereoselectivies for 2,3-Disubstituted Furans 24
8. Computational Analysis 25
Computational Details 25
Introduction 26
Relative Free Protonation and Hydride Energies 27
NBO Analysis 27
Explicit Solvation 29
9. Preparation of Starting Material 30
10. Substrates 42
11. Examples Characterized Within Crude Mixtures 59
12. Derivatisation 63
13. Spectra 64
14. Appendix 135
^{1}H NMR Yields 135
NMR Spectra of Dimeric Product III 143
Spectra for Deuteration Studies 146
Computational Discussion of 2D-Plot and Isomeric Analysis 152
Thermodynamic Data 155
15. References 159

1. Materials and Methods

Unless otherwise stated, all reactions were magnetically stirred and conducted under ambient conditions. Moisture-sensitive reactions were conducted in flame-dried glassware in anhydrous solvents and under argon, applying standard Schlenk techniques. Solvents and liquid reagents, as well as solutions of solid or liquid reagents were added via syringes, stainless steel or polyethylene cannulas through rubber septa or through a weak argon counter-flow. Especially, TfOH or TFA was added via Argon-spared *Hamilton Glass Syringes* or via freshly prepared-stock solution in HFIP. Reactions at lower temperatures (T < RT) were cooled to the specified temperature using appropriate cooling baths. Cooling baths were prepared in Dewar vessels, filled with ice/water (0 °C), ice/NaCl mixtures (~ –20 °C) or dry ice/acetone (–78 °C). Heated oil baths were used for reactions requiring elevated temperatures. Solvents were removed under reduced pressure at 30 °C using a rotary evaporator. All given yields are isolated yields of chromatographically and NMR-spectroscopically pure materials, unless otherwise stated.

Chemicals were purchased from commercial suppliers (including abcr, Acros Organics, Alfa Aesar, Fluorochem, SigmaAldrich, BLDPharm, Carbolution and TCI) and used without further purification unless otherwise stated. HFIP was purchased from SigmaAldrich and Carbolution and was used without further purification. Other solvents (CyH, DCM, Et_2O, THF, toluene) were dried by distillation from an appropriate drying agent in the technical department of the Max-Planck-Institut für Kohlenforschung and received in Schlenk flasks under argon. Reactions were monitored by thin layer chromatography (TLC) on silica gel pre-coated plastic sheets (0.2 mm, MachereyNagel). Visualization was accomplished by irradiation with UV light (254 nm and 366 nm) and/or phosphomolybdic acid (PMA) stain and/or permanganate stain. Flash column chromatography was carried out using Merck silica gel (60 Å, 230−400 mesh, particle size 0.040−0.063 mm) using technical grade solvents. Elution was accelerated using compressed air.

^{1}H, ^{13}C and ^{19}F nuclear magnetic resonance (NMR) spectra were recorded on a Bruker AVIII-500 MHz or Bruker NEO 600 MHz (equipped with a BBO CryoProbe) spectrometer in a suitable deuterated solvent. The solvent employed and respective measuring frequency are indicated for each experiment. Chemical shifts are reported in ppm (δ) relative to residual solvent resonance serving as the internal reference (δ 7.26 ppm for $CDCl_3$; δ 5.32 ppm for CD_2Cl_2; δ 7.16 ppm for C_6D_6). The resonance multiplicity is described as s (singlet), d (doublet), t (triplet), q (quadruplet), p (pentet), hept (heptet), m (multiplet), and b (broad) as well as app (apparent). All spectra were recorded at 298 K, processed with the program MestReNova 15.0.0, and coupling constants are reported as observed. Data are provided as follows: chemical shift in ppm, resonance multiplicity, coupling constant *J* in Hz, and number of protons. All spectra are broadband decoupled unless otherwise noted.

Electron impact (EI) mass spectrometry (MS) was performed on a Finnigan MAT 8200 (70 eV) or MAT 8400 (70 eV) spectrometer. Electrospray ionization (ESI) mass spectrometry was conducted on a Bruker ESQ 3000 spectrometer. High resolution mass spectrometry (HRMS) was performed on a Finnigan MAT 95 (EI) or Bruker APEX III FTMS (7T magnet, ESI). The ionization method and mode of detection employed is indicated for the respective experiment and all masses are reported in atomic units per elementary charge (*m/z*) with an intensity normalized to the most intense peak.

2. Troubleshooting

Question: The observed reaction time varies from the reported one. For example, the partial reduction of a 3-aryl furan to a 3-aryl-2,5-dihydrofuran takes longer than reported.

Depending on the HFIP batch used, certain sub-percentage impurities can contribute to catalyst inhibition, varying the required reaction time. Based on the batch of commercial HFIP used, slower/faster kinetics were observed. We recommend monitoring the reaction closely upon the first use of a new HFIP batch.

Question: Slight overreduction is observed in the partial reduction of a 3-aryl furan using catalytic TFA.

As stated above, impurity-free HFIP might contribute to exceptionally fast kinetics. Full conversion of starting material furan to a 3-aryl-2,5-dihydrofuran leads to a kinetic plateau, after which over-reduction slowly proceeds. Closer time monitoring of the reaction usually identifies the appropriate reaction time (12 h ± 3 h), not "overshooting" this plateau.

Question: How should the furan starting material be stored?

All furans should be stored long-term at −20 °C under an Ar atmosphere. For example, we observed slow degradation of 3-aryl furans under ambient temperature over a few weeks.

Question: While monitoring the reaction, stagnation of conversion has occurred.

Brønsted-acid catalyzed silylation of HFIP is a competing side reaction. For certain slow-reacting substrates like **2t**, complete silane consumption is reached before all of the furan substrate can be reduced. The addition of a second silane portion after stagnation usually solves this problem.

Question: Some substrates appear to react sluggish.

If a substrate is encountered that only shows partial conversion and further addition of silane does not help, it might be advantageous to perform the reaction with a slightly higher Brønsted-acid catalyst loading (5 mol% instead of 2 mol%). For example, in the case of Menthofuran **1p**, a catalyst loading of 2 mol% results in approximately 50% conversion. Performing the reaction at 5 mol% leads to complete conversion and a cleaner reaction profile.

Question: The used substrated decomposes in HFIP.

Sometimes, a change of addition sequence can avoid this problem. For example for 3-(chloromethyl)furan **1m**, the reaction mixture was added onto the neat substrate 3-(chloromethyl)furan, delivering the reduced product without much decomposition. Usually, 3-(chloromethyl)furan decomposes if dissolved in HFIP.

3. General Procedures

General Procedure for Conditions (A)

Triethylsilane (1.50 eq.), distilled water (1.00 eq.), and trifluoroacetic acid TFA (5.0 mol%) were dissolved in HFIP (0.40 M) under ambient conditions followed by addition of a solution of the substrate furan (1.00 eq.) in HFIP (0.40 M) in one portion under vigorous stirring. The final concentration of furan is 0.20 M. The reaction was stirred at ambient conditions for the indicated time. The reaction progress was monitored *via* TLC and/or ^{1}H NMR aliquots (see each substrate entry for detailed reaction times). Upon completion, the mixture was quenched with one drop of NEt_3, and concentrated. The residue was purified by flash column chromatography.

General Procedure for Conditions (B)

Triethylsilane (2.50 eq. or stated otherwise) and triflic acid (2.0 mol%) were dissolved in HFIP (0.40 M) under ambient conditions. A solution of the substrate furan (1.00 eq.) in HFIP (0.40 M) was added *immediately* to this in one portion under vigorous stirring. The final concentration of furan is 0.20 M. The reaction was stirred at ambient conditions for 10 min to 12 h. The reaction progress was monitored *via* TLC and/or ^{1}H NMR aliquots (see each substrate entry for detailed reaction times). Upon completion, the mixture was quenched with one drop of NEt_3, and concentrated. The residue was purified by flash column chromatography.

4. Unsuccessful Substrates

The following substrates were found to be too electron-poor to be converted under the reported Conditions (A) or (B):

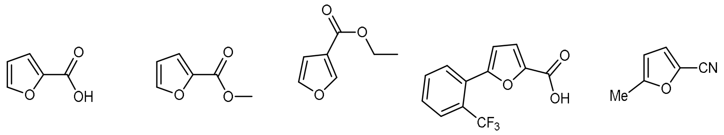

Further, only esters were tolerated under Conditions (A) or (B). Ketones or hydroxy-derivatives were further reduced off leading to fully and partially reduced and deoxygenated products:

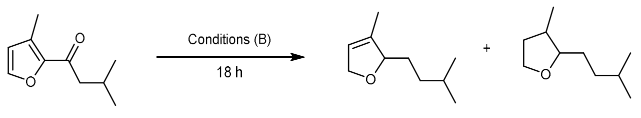

Elsholtzia Ketone

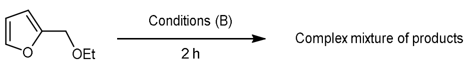

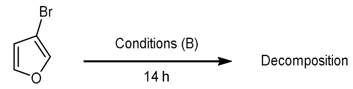

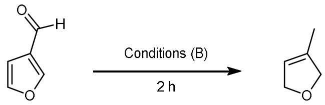

5. Identification of Sideproducts

One major challenge in furan chemistry is its inherent polymerization behavior—historically called "resinification".[1] In the methodology described here, HFIP as a solvent choice is crucial in suppressing this behavior compared to other tested solvents.

No significant polymerization behavior was usually detected when employing HFIP as a solvent in this methodology (compare Optimization, p. 8). Nevertheless, resignification behavior was observed for 2-aryl or 2-alkyl substituted furans. Under the reported optimized reaction conditions, 2-alkyl furan **3h** usually accompany an unavoidable 5-10% resignified byproduct **III**.

2-Pentyl-5-(pentyltetrahydrofuran-3-yl)furan **III**

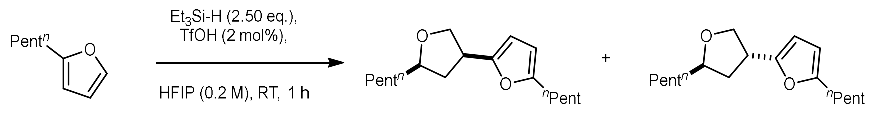

III

R$_f$ 0.23 (*n*-pentane / Et_2O, 95:5).

^{1}H NMR (600 MHz, $CDCl_3$) δ 5.92 (td, J = 0.9, 3.4 Hz, 1H), 5.86–5.84 (m, 1H), 4.16 (dd, J = 7.4, 8.5 Hz, 1H), 4.03 (t, J = 8.1 Hz, 1H), 4.02–3.98 (m, 1H), 3.97–3.92 (m, 1H), 3.83 (dd, J = 7.8, 8.2 Hz, 1H), 3.72 (dd, J = 7.3, 8.5 Hz, 1H), 3.48–3.41 (m, 2H), 2.58–2.54 (m), 1.84 (ddd, J = 7.4, 9.2, 12.4 Hz, 1H), 1.70– 1.64 (m), 1.64–1.59 (m), 1.51–1.40 (m), 1.37–1.24 (m), 0.91–0.87 (m).

Note: Due to multiple overlapping signals not all ^{1}H multiplets could be assigned as multiplets. Integration are given for indicative signals. Full spectra shown on p. 143.

^{13}C NMR (151 MHz, $CDCl_3$) δ 155.6, 154.3, 79.4, 77.2, 76.9, 72.1, 38.3, 37.3, 35.9, 32.1, 31.6, 28.2, 27.9, 26.1, 22.8, 22.6, 14.18, 14.16.

*Note: All ^{13}C NMR signals overlap of the two diastereomers (see assignment **Table S1** and **Table S2**).*

Table S1. Tabulated NMR assignments (^{1}H NMR: 600 MHz, ^{13}C NMR: 151 MHz, $CDCl_3$) and 2D NMR correlations for dimer **III**. A NOE between H12 - H11' and H10 - H11'' confirms the *trans* configuration.

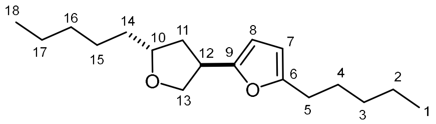

Atom	δ (ppm)	COSY	HSQC	HMBC	NOESY
**1 C**	14.16		1		
**H₃**	0.89	2	1		
**2 C**	22.55		2		
**H₂**	1.33	1	2	3	
**3 C**	31.55		3', 3''	2, 5', 5''	
**H'**	1.33	4	3		
**H''**	1.32		3		
**4 C**	27.86		4	5', 5''	
**H₂**	1.62	3', 5', 5''	4		5', 5'', 7
**5 C**	28.15		5', 5''		
**H'**	2.55	4	5	3, 4, 6, 7	4, 7
**H''**	2.57	4	5	3, 4, 6, 7	4, 7
**6 C**	155.64			5', 5''	
**7 C**	105.02		7	5', 5''	
**H**	5.85	8	7		4, 5', 5''
**8 C**	105.02		8		
**H**	5.92	7	8	9	11'', 13'
**9 C**	154.26			8, 11'', 13', 13''	
**10 C**	79.44		10	13''	
**H**	4.01	11', 11'', 14''	10		11'', 13', 14'
**11 C**	37.29		11', 11''		
**H'**	1.84	10, 12	11	12	12
**H''**	2.15	10, 12	11	9	8, 10
**12 C**	38.35		12	11'	
**H**	3.45	11', 11'', 13''	12		11', 13', 13''
**13 C**	72.11		13', 13''		
**H'**	3.72	13''	13	9	8, 10, 12, 13''
**H''**	4.16	12, 13'	13	9, 10	12, 13'
**14 C**	35.90		14', 14''		
**H'**	1.62		14		10
**H''**	1.48	10	14		
**15 C**	26.05		15', 15''		
**H'**	1.31		15		
**H''**	1.43		15		
**16 C**	32.07		16	18	
**H₂**	1.31		16		
**17 C**	22.78		17	18	
**H₂**	1.31	18	17		
**18 C**	14.18		18		
**H₃**	0.89	17	18	16, 17	

Table S2. Tabulated NMR assignments (^{1}H NMR: 600 MHz, ^{13}C NMR: 151 MHz, $CDCl_3$) and 2D NMR correlations for dimer **III**. A NOE between H12 - H10 confirms the *cis* configuration.

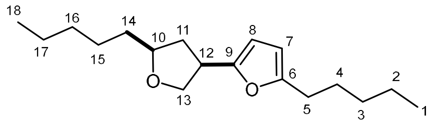

Atom	δ (ppm)	COSY	HSQC	HMBC	NOESY
**1 C**	14.16		1		
**H_3**	0.89	2	1		
**2 C**	22.55		2		
**H_2**	1.33	1	2	3	
**3 C**	31.55		3', 3"	2, 5', 5"	
**H'**	1.33	4	3		
**H"**	1.32		3		
**4 C**	27.86		4	5', 5"	
**H2**	1.62	3', 5', 5"	4		5', 5", 7
**5 C**	28.15		5', 5"		
**H'**	2.55	4	5	3, 4, 6, 7	4, 7
**H"**	2.57	4	5	3, 4, 6, 7	4, 7
**6 C**	155.60			5', 5"	
**7 C**	105.02		7	5', 5"	
**H**	5.85	8	7		4, 5', 5"
**8 C**	105.02		8		
**H**	5.92	7	8	9	11", 13'
**9 C**	153.92			8, 11", 13', 13"	
**10 C**	80.37		10	13"	
**H**	3.94	11', 11", 14"	10		11", 12, 13', 14'
**11 C**	38.03		11', 11"		
**H'**	2.34	10, 12	11	12	
**H"**	1.66	10, 12	11	9	8, 10
**12 C**	39.19		12	11'	
**H**	3.46	11', 11", 13"	12		10, 13', 13"
**13 C**	71.68		13', 13"		
**H'**	4.04	13"	13	9	8, 10, 12, 13"
**H"**	3.83	12, 13'	13	9, 10	12, 13'
**14 C**	35.81		14', 14"		
**H'**	1.62		14		10
**H"**	1.48	10	14		
**15 C**	26.14		15', 15"		
**H'**	1.31		15		
**H"**	1.43		15		
**16 C**	32.07		16	18	
**H_2**	1.31		16		
**17 C**	22.78		17	18	
**H_2**	1.31	18	17		
**18 C**	14.18		18		
**H_3**	0.89	17	18	16, 17	

6. Optimization

While developing the reaction, two model furans were subjected to conditions screening: 2-Pentylfuran **3h** and 3-arylfurans, such as 3-phenylfuran **1d**. The optimization campain was accompanied either with resinification (polymer precipitate) or successful conversion to the product in almost quantitative yields. In case of resinification, only *Dimer III* was able to be characterised fully (see p. 5). Other oligmers/polymers appeared as insoluble precipitate in the reaction vessel.

Under Conditions (A) and (B) following solvents were screened at room temperature and reflux conditions: dichloromethane, toluene, distilled water, isopropyl alcohol, methanol, acetonitrile, propylene carbonat:heptane mixture (5:4, *v:v*). In all cases slow resinification/polymerization is observed without productive conversion into any identifiable intermediate. HFIP proved to be the only tested solvent to be able to yield clean reaction profiles with significant product formation.

Further, different silanes and hydride sources were screened. Aliphatic hydrosilanes delivered similar reaction profiles. In contrast, diphenylsilane and 1,1,3,3-tetramethyldisiloxan and other reducing agents, such as 1,4-cyclohexadiene or $BH_3 \cdot THF$ solution were not effective (Table S3).

Table S3. Screening other reducing agents in HFIP (2.0 M), 20 h, RT.

	**2-Pentylfuran** *(2.5 eq. [H] source, 2 mol% TfOH)*	**3-Phenylfuran** *(1.5 eq. [H] source, 5 mol% TFA, 1.0 eq. H_2O)*
**Ph_2SiH_2**	no product observed, 30% dimer III	Returned SM
**Me_2EtSiH**	same reaction profile as with Et_3SiH	same reaction profile as with Et_3SiH
**$MeEt_2SiH$**	same reaction profile as with Et_3SiH	same reaction profile as with Et_3SiH
**Tetramethyldisiloxan**	no product, 20% of an unidentified furan-adduct	Returned SM
**1,4-Cyclohexadiene**	no product, 3% remaining SM, resinified precipitate	Returned SM
**$BH_3 \cdot THF$**	no product, 15% remaining SM, resinified precipitate	67% remaining SM, rest resinified

7. Mechanistic Studies

Deuteration Studies

If deuterated reagents are used in the reported reaction Conditions (A) or (B), the regiochemical behavior of each reagent can be determined. For that, Et_3Si-D, D_2O or d_2-HFIP was employed in three distinct reactions:

Conditions (A) on Substrate 1b

Employing Et_3Si-D, full deuteration (100%) at the C5-position is observed:

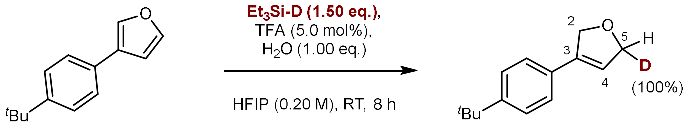

Employing d_2-HFIP, deuteration at the C2-position is observed. This indicates that protonation happens at the C2 position. In this case, even overdeuteration by 44% occurs. This is due to the fact that the C2-position rapidly exchanges with HFIP solvent, as shown by an EXSY NMR during the initial protonation event (see p. 11).

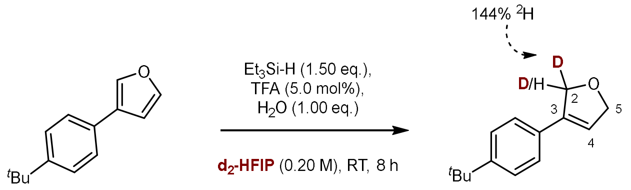

Interestingly, when using deuterated water, no significant deuterium incorporation was observed. This can be explained by the higher acidity of HFIP and its excess as a solvent. Thus, the role of water is only to regenerate the silylated catalyst (towards Et_3Si-OD and CF_3COO^- undergoing regeneration) through its higher nucleophilicity compared to HFIP (see Discussion in main text).

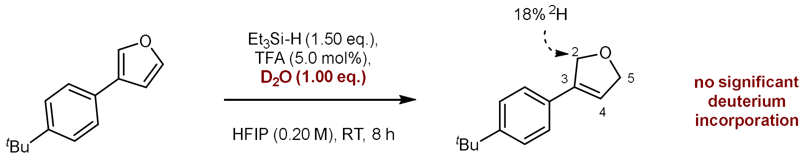

Conditions (B) on Substrate 3h

*Note: The following analysis was conducted from the ^1H and ^2H NMR spectra of the crude reaction mixtures. As these still contain the described dimer **III** (~10%), accurate integrations are hampered, and thus, the results will only be interpreted qualitatively. Detailed spectra and assignments are displayed in Appendix (p. 146).*

Employing Et_3Si-D, deuteration (84%) at the C2 position and C4 position (82%) is observed.

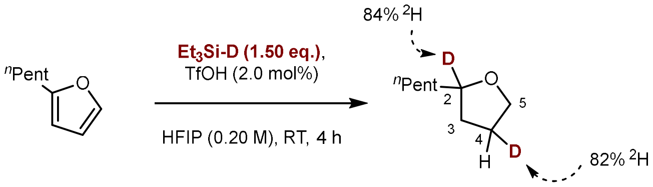

Employing d_2-HFIP, deuteration at the C5 position is observed, indicating protonation at this position. In this case overdeuteration (172%) is observed. It is expected that upon protonation, the C5 position rapidly exchanges with HFIP solvent, as shown by an EXSY NMR for compound **1a** (see p. 11). Secondly, the C3 position gets deuterated with a facial unselectivity of 42%:44%.

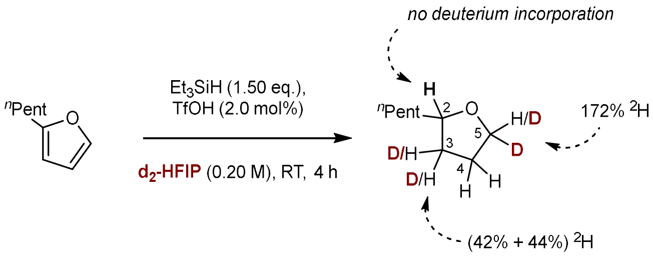

EXSY-NMR Studies

To characterize an ion pair, a stoichiometric experiment between **1a** and TFA was conducted.

Procedure: A *J. Young's* NMR tube was flame-dried under an Ar-atmosphere. 3-(4-Methoxyphenyl)furan **1a** (10.0 mg, 0.057 mmol, 1.00 eq.) was added against an Ar-stream. Subsequently, distilled HFIP (0.57 mL; final concentration 0.10 M) was added *via* syringe. Finally, TFA (4.4 µL, 0.057 mmol, 1.00 eq.) was added with a Hamilton syringe. The *J. Young's* NMR tube was closed and shaken vigorously before NMR measurements commenced immediately. The mixture slowly decomposes (~ hours).

No formation of an ion pair was observed. Instead, an exchange was observed between H_2 and H_A (Figure S1). No exchange of the H_5 was visible. Therefore, for substrate **1a**, protonation happens selectively at the H_2 position.

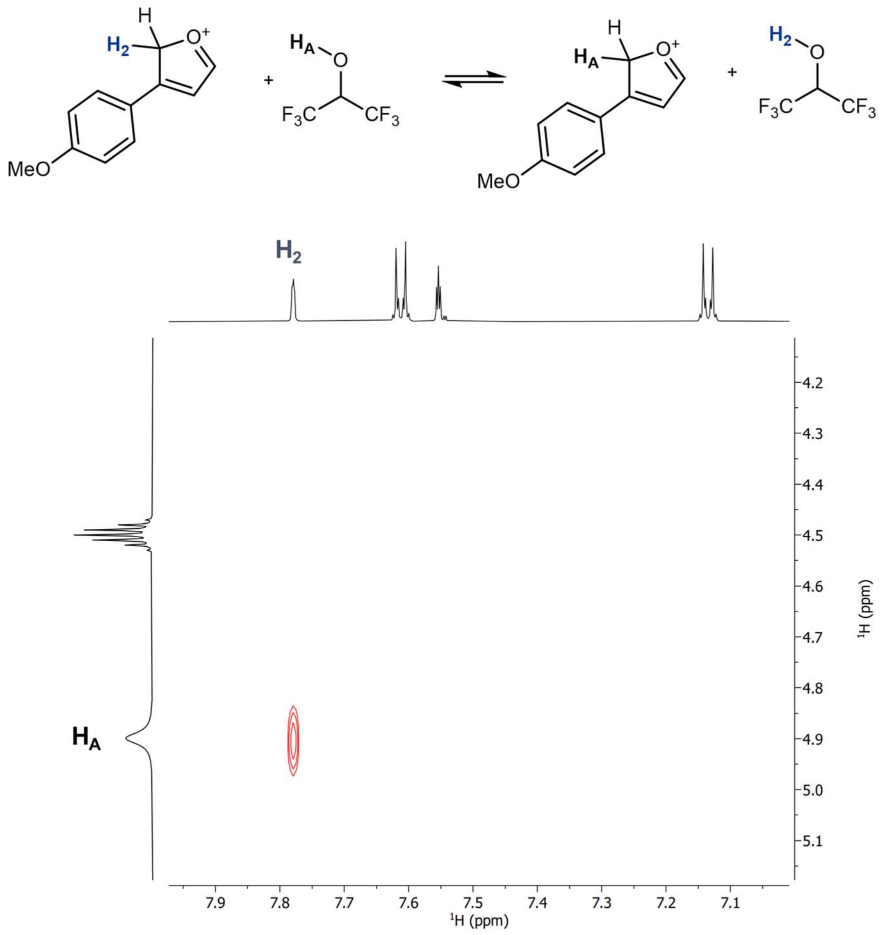

Figure S1. ^1H–^1H EXSY NMR of **1a** and TFA (1.0 eq.) in HFIP.

Other substrates were investigated similarly, albeit using TfOH (1.0 eq.) as an acid. Decomposition via polymerization was observed for plain furan **1u**. No EXSY exchange was visible for electron-poor substrate **1t**, indicating that the exchange/protonation is slower than the NMR timescale.

Kinetic Isotope Effects

An intermolecular competition experiment was conducted between Et_3Si-H and Et_3Si-D (3.0 eq. and 3.0 eq.) according to *Conditions (A)*, albeit stopping the reaction after 4 h:

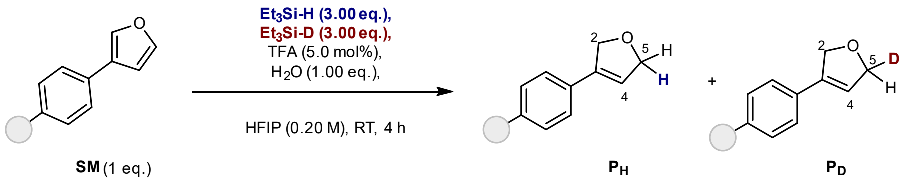

The crude reaction mixture was purified via preparative TLC (10% Et_2O in *n*-pentane, extracted with DCM from silica), isolating co-eluting P_H and P_D with residual solvents. Prior of use, the mixture of Et_3Si-H and Et_3Si-D was verified via ^1H NMR to have a equimolar H:D ratio of 1.0:1.0.

Determination On Basis of ^1H NMR Spectra

Quantitative ^1H NMR spectra were recorded. The spectra were baseline-corrected with Whittaker Smoother. The KIE was determined through the integral depletion (see Figure S2) of H5 with equation (1). All results are summarized in Table S4.

$$\mathrm{KIE} = \frac{[P_H]}{[P_D]} = \frac{\mathrm{Integral}(H5) - 1}{2 - \mathrm{Integral}(H5)} \qquad (1)$$

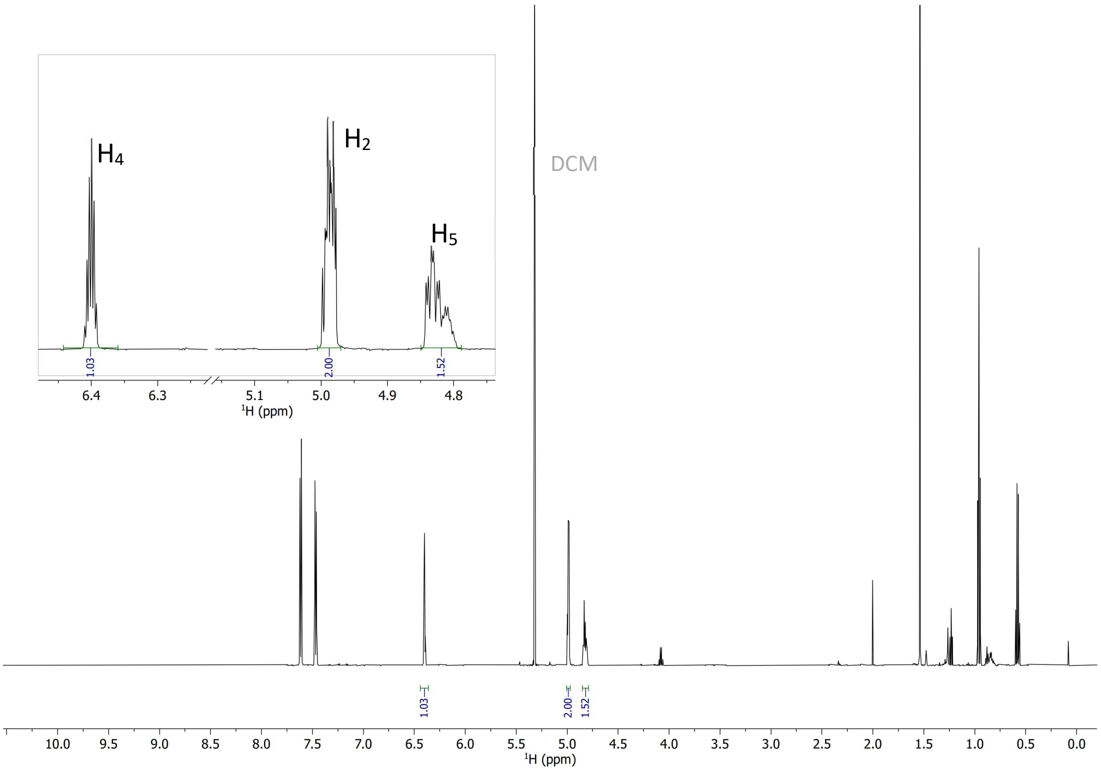

Figure S2. Example integration on compound **2f** (-CF_3).

Determination On Basis of ^{13}C NMR Spectra

Analog to the ^{1}H analysis, the carbon C4, adjacent to the deuteration center C5, can be used to determine the ratio of deuterated and non-deuterated species. The obtained data is shown in Table S4-A too.

Table S4-A. Determination of the KIE.

Exp. 1	Integral H2	Integral H5	KIE ^{1}H	Integral ^{13}C-H	Integral ^{13}C-D	KIE ^{13}C
–OMe	2.00	1.45	0.82	1.00	1.29	0.78
–^{t}Bu	2.00	1.47	0.89	1.00	1.23	0.81
–Me	2.00	1.47	0.89	1.00	1.26	0.79
–H	2.00	1.47	0.89	1.00	1.16	0.86
–Cl	2.00	1.50	1.00	1.00	1.19	0.84
–CF_3	2.00	1.52	1.08	1.00	1.04	0.96
Exp. 2	**Integral H2**	**Integral H5**	**KIE ^{1}H**	**Integral ^{13}C-H**	**Integral ^{13}C-D**	**KIE ^{13}C**
–OMe	2.00	1.45	0.82	1.00	1.24	0.81
–^{t}Bu	2.00	1.46	0.85	1.00	1.19	0.84
–Me	2.00	1.47	0.89	1.00	1.27	0.79
–H	2.00	1.47	0.89	1.00	1.16	0.86
–Cl	2.00	1.50	1.00	1.00	1.14	0.88
–CF_3	2.00	1.53	1.13	1.00	1.01	0.99

Compared to the obtained ^{1}H NMR spectroscopic data, it shows the same qualitative trend but is associated with a larger random error based on the chosen integration borders. Therefore, it was not included in the calculation of the KIE values depicted in the main manuscript (Table S4-B).

Table S4-B. Determination of the KIE for the main manuscript and associated standard deviation.

	Avarage μ(KIE)	Standard Deviation σ(KIE)
–OMe	0.82	0.000
–^{t}Bu	0.87	0.025
–Me	0.89	0.000
–H	0.89	0.000
–Cl	1.00	0.000
–CF_3	1.11	0.031

Determination with Mass Spectrometry

Complimentary determination of the deuteration degree via mass spectroscopy was impeded by rearomatisation of the 2,5-dihydrofuran samples within the gas phase.

Hammett Studies

An intermolecular competition experiment was conducted between reference substrate **1d** and the respective derivatives **1a**–**1e**:

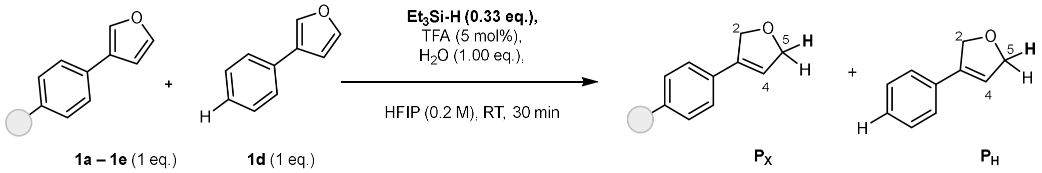

The crude reaction mixture was measured via ^{1}H NMR spectroscopy and the product ratio P_H and P_x was used as k_x/k_H. The raw data associated with the shown Hammett-plot in Figure 5D (main text) is shown in Table S5.

Table S5. Determination of the k_x/k_H on basis of ^{1}H NMR. Hammett substituent constants σ_p taken from [2]

	k_x/k_h	$\log(k_x/k_h)$	σ_p
**1e** (–Cl)	0.55	-0.25964	0.23
**1a** (–OMe)	1.95	0.29004	-0.27
**1b** (–^{t}Bu)	2.02	0.30535	-0.2
**1f** (–CF_3)	0.12	-0.93197	0.54
**1c** (–Me)	2.07	0.31597	-0.17
**1d** (–H)	1.00	0	0

Kinetic Profiling

Substrate **1b** was chosen for a detailed non-deuterated (No-D) ^{1}H NMR[3] kinetic measurement to monitor the reduction towards **2b**. In all the cases, the following procedure was followed:

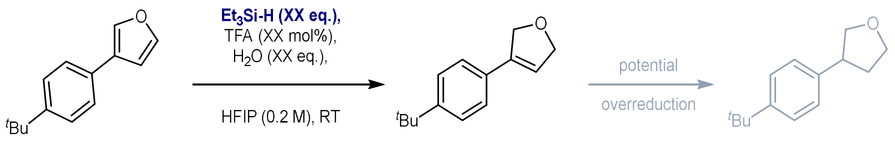

Preparation of stock solution: A stock solution of H_2O and TFA was prepared by dissolving a tenfold amount (needed for the experiment) of H_2O (18.0 µL, 1.00 mmol, 10.00 eq.) and TFA (3.8 µL, 0.05 mmol, 0.50 eq.) in 1.00 mL HFIP.

Procedure: An NMR tube was charged with the respective amount of silane and non-deuterated HFIP (100 µL) under ambient conditions. The above-described stock solution was vigorously shaken, and 100 µL (a tenth) was added *via* syringe to the above-prepared NMR tube containing silane and HFIP. The NMR tube was vigorously shaken. Compound **1b** (20.0 mg, 0.10 mmol) was dissolved in HFIP (300 µL) and vigorously shaken. Afterward, the compound solution was added to the NMR tube, vigorously shaken, and quickly transferred to the NMR probe.

NMR measurements: After the sample was transferred to a Bruker AVIIIHD 400 MHz NMR spectrometer preheated to 298 K, it was quickly shimmed (Topspin command: *topshim 1H o1p=[HFIP-CH] selwid=0.3 ppm lockoff*) and monitored with single scan ^{1}H NMR spectra every 2-5 minutes until an appropriate conversion was reached. Afterward, the data was imported with the Reaction Monitoring Plugin into MNVOVA 15.0.0 and processed therein (phasing, baseline correction, integration).

Note: Different absolute rate constants were observed depending on the batch of HFIP used (commercial supplier). Further, the used Et_2MeSi-H and $EtMe_2Si$-H contained THF as an impurity.

Silane equivalents

First, the Et_3SiH equivalents were varied. The above procedure was conducted using either 1.50 eq. Et_3SiH (24.0 µL, 0.15 mmol) or 3.00 eq. Et_3SiH (48.0 µL, 0.30 mmol) in combination with 5 mol% TFA (0.38 µL, 0.0050 mmol) and 1.00 eq. distilled H_2O (1.8 µL, 0.10 mmol).

The collected profiles are shown in Figure S3. The measured rate does not significantly differ between 1.50 eq. and 3.0 eq. of silane. Therefore, a 0th-order dependence on Et_3SiH can be approximated.

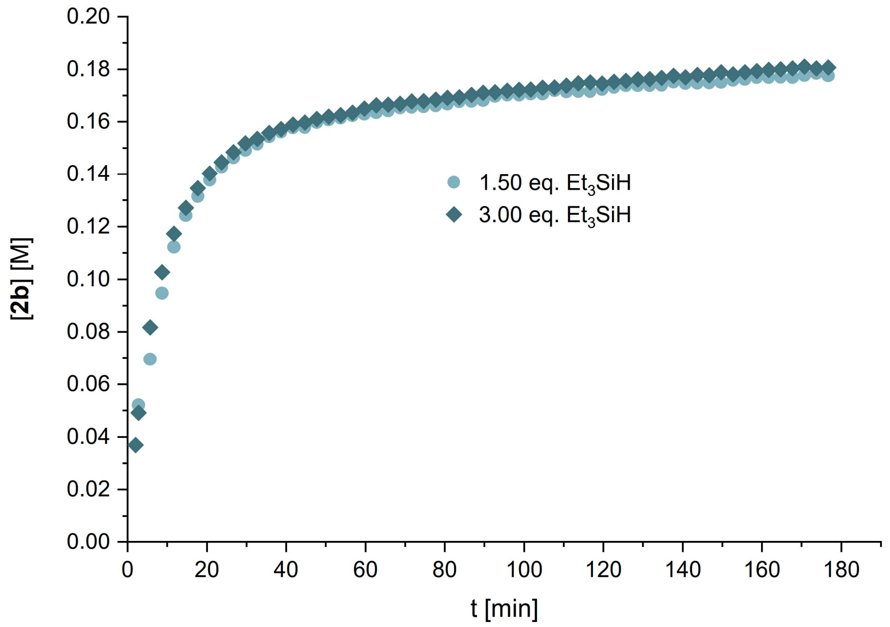

Figure S3. ^{1}H NMR reaction monitoring of different Et_3SiH equivalents using furan **1b**.

TFA catalyst loading

Secondly, the TFA catalyst loading was varied. The above procedure was conducted using either 2.5 mol% TFA (0.19 µL, 0.0025 mmol), 5.00 mol% TFA (0.38 µL, 0.0050 mmol), or 10.0 mol% TFA (0.77 µL, 0.010 mmol) in combination with 1.50 eq. Et_3SiH (24.0 µL, 0.15 mmol) and 1.00 eq. distilled H_2O (1.80 µL, 0.10 mmol).

Figure 5A (main text) shows the collected profiles, in which two regimes can be identified. In the first few minutes, a fast consumption of furan **1b** shows a clear dependence on the catalyst loading. As expected, higher catalyst loading leads to faster consumption. Interestingly, soon after, the reaction slows down significantly and changes to a linear regime. The onset of this is dependent on the catalyst concentration. However, the decay rate is no longer visibly dependent on the catalyst loading. It can be concluded that a mechanism change must happen during the reaction monitoring. Due to this complex profile, a VTNA analysis[4] in order to determine the catalyst order was not applicable.

H_2O equivalents

Thirdly, the H_2O equivalents were also varied. The above procedure was conducted using either no extra-added water, 1.00 eq. (1.8 µL, 0.10 mmol), or 2.00 eq. H_2O (3.6 µL, 0.20 mmol) combined with 1.50 eq. Et_3SiH (24.0 µL, 0.15 mmol) and 5.0 mol% TFA (0.38 µL, 0.005 mmol).

Without employing any extra-added water, the reaction conversion halts at around 25%. Only when 1.00 or 2.00 eq. of H_2O were added did the initial fast-decaying profile develop. The rate in the first regime is slower when 2.0 eq. of H_2O is employed compared to 1.00 eq. of H_2O. This relative trend inverts after the system enters the second regime, in which 2.0 eq. of H_2O proceeds slightly faster (see Figure S4).

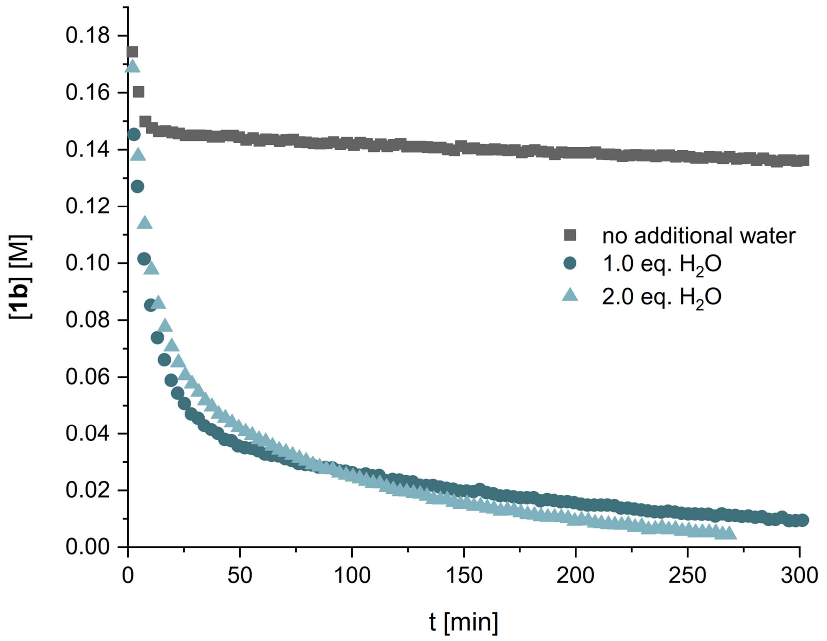

Figure S4. ^{1}H NMR reaction monitoring of different H_2O equivalents.

When a portion of 1.00 eq. H_2O (1.8 µL, 0.10 mmol) is added to the NMR sample containing no additional water after 24 h standing time, the exponential conversion starts as observed before (see Figure 5B, main text).

Identification of the Silylated Catalyst

If Et_2MeSiH is employed instead of Et_3SiH, the silylated catalyst **5c** can be clearly identified through evolution of a novel Me-singlet. In addition to the expected silylated byproducts, an additional, deshielded ^{29}Si signal could be detected which can be assigned to silylated TFA **5c**.

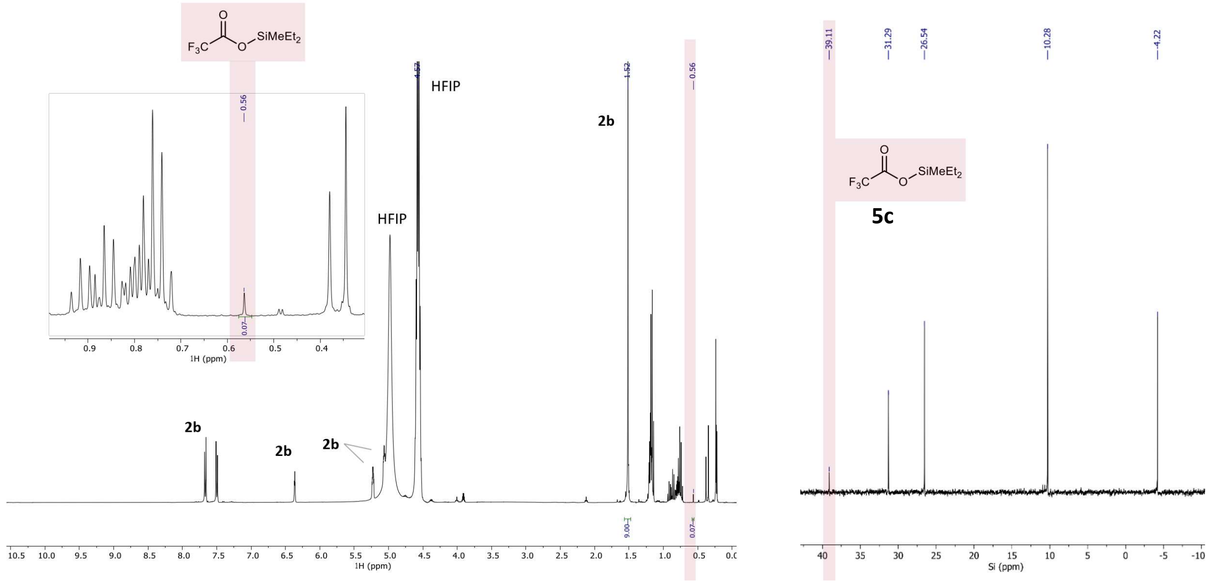

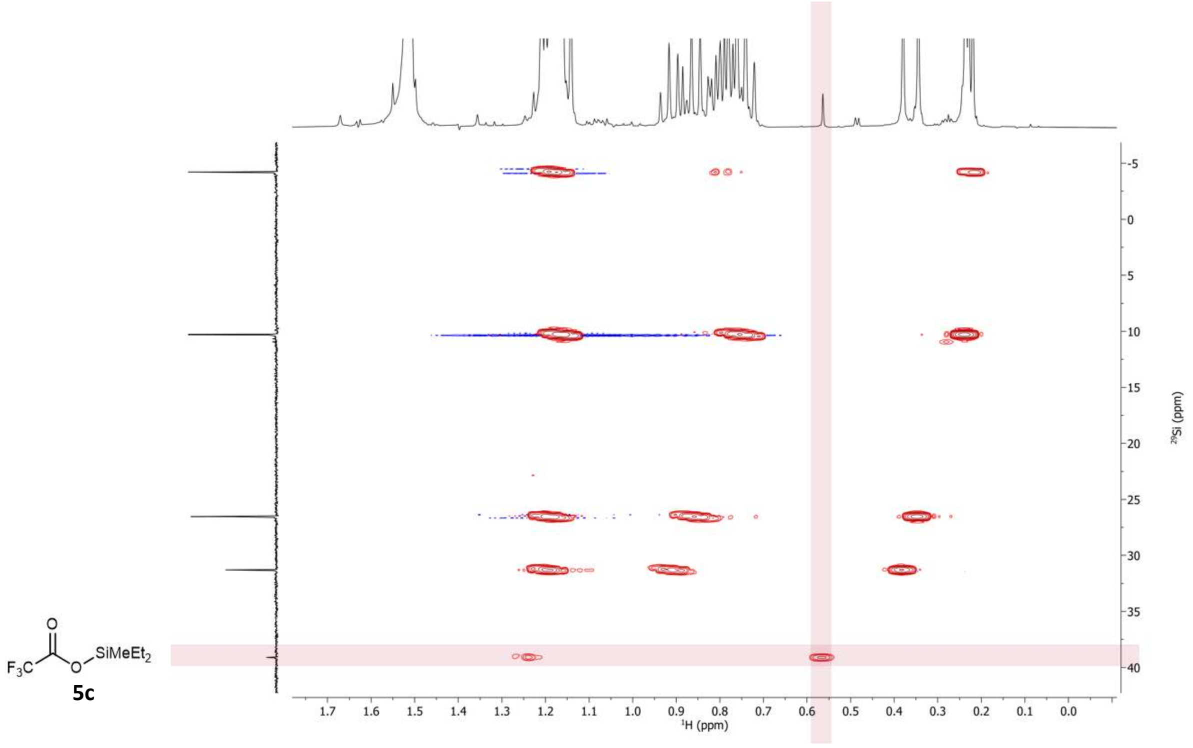

Figure S5. Identification of silane species, including **5c**, with a ^{1}H –^{29}Si-HMBC. Projections on the top and the left show acquired 1D ^{1}H and ^{29}Si-INEPT spectra of the reaction mixture.

As the Et_2MeSi-H methyl-group appears as a well-separated singlet in the ^1H NMR spectra (Figure S5), it is possible to quantify the silylated catalyst **5c** over time at different catalyst loadings (Figure S6). The silylated TFA species **5c** builds up quickly within the first 15 minutes and approaches a steady-state concentration asymptotically, albeit at 10 mol% catalyst loading the concentration slowly decays again over time. Notably, the silylated catalyst never reaches the theoretical maximum concentration of the initial TFA concentration. For example, 0.010 M for 5 mol% initial TFA loading was not reached. Instead, a limit of ca. 0.006 M silylated catalyst **5c** concentration corresponds to ca. 60% silylation degree.

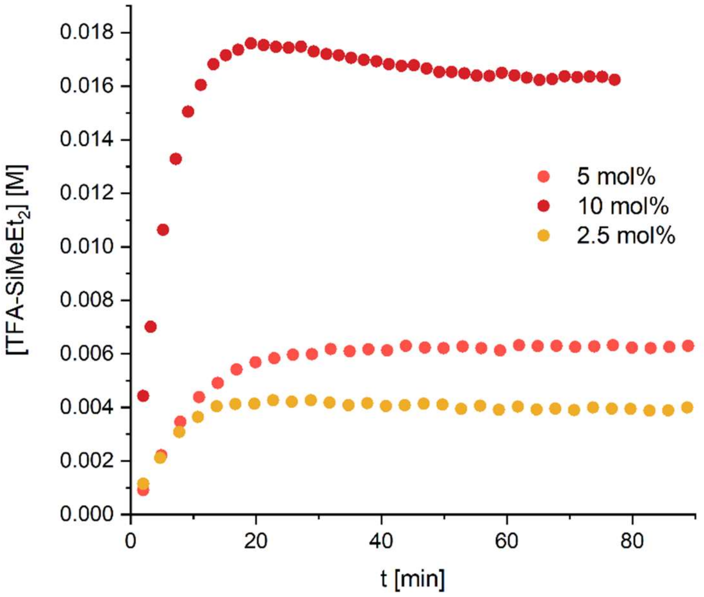

Figure S6. ^1H NMR reaction monitoring of [TFA-$SiMeEt_2$] **5c** throughout the reaction at different catalyst loadings.

NMR Titrations

A ^{1}H NMR titration was performed to study the HFIP solvent association behavior with **4i** and **3i** of the reaction mixture.

*2-Methyltetrahydrofuran **4i***

HFIP (10.5 µL, 0.10 mmol, 1.00 eq.) was dissolved in 1.0 mL $CDCl_3$ (final concentration 0.10 M) in an NMR tube. A ^{1}H NMR spectrum was measured. Then, commercial 2-methyltetrahydrofuran (2-MeTHF **4i)** was added in approximately 0.20 eq. portions (~ 2.0 µL, 0.020 mmol, 0.20 eq.). The mixture was vigorously mixed, and at every step, a ^{1}H NMR spectrum was measured. Based on the HFIP starting concentration $[HFIP]_0$, the relative [2-MeTHF] was calculated (Table S6).

Table S6. Derived relative [2-MeTHF] and associated δ(HFIP) shift.

Entry	[2-MeTHF][mol L^{-1}]	δ(HFIP)
1	0.000	3.07
2	0.019	3.59
3	0.036	4.03
4	0.057	4.41
5	0.072	4.66
6	0.089	4.88
7	0.107	5.11
8	0.128	5.34
9	0.164	5.64
10	0.208	5.94
11	0.281	6.24
12	0.377	6.45
13	0.500	6.62
14	0.644	6.75
15	0.893	6.83

The binding isotherms were plotted in Origin 2019b and a non-linear regression approach was followed based on Monaco et al.[5] to evaluate the equilibrium constant:

$$[HG] = \frac{1}{2}\left([G_0] + [H_0] + \frac{1}{K}\right) - \sqrt{\frac{1}{4}\left([G_0] + [H_0] + \frac{1}{K}\right)^2 - [H_0][G_0]}$$

$$\delta(HFIP) = \frac{[HG]}{[H_0]}\left(\delta(HG) - \delta(H)\right) + \delta(H)$$

A good correlation ($R^2 = 0.999$) confirms this 1:1 binding model with a binding constant of $K = 17.9 \pm 0.72$ mol L^{-1} between HFIP and 2-MeTHF (see Figure S7).

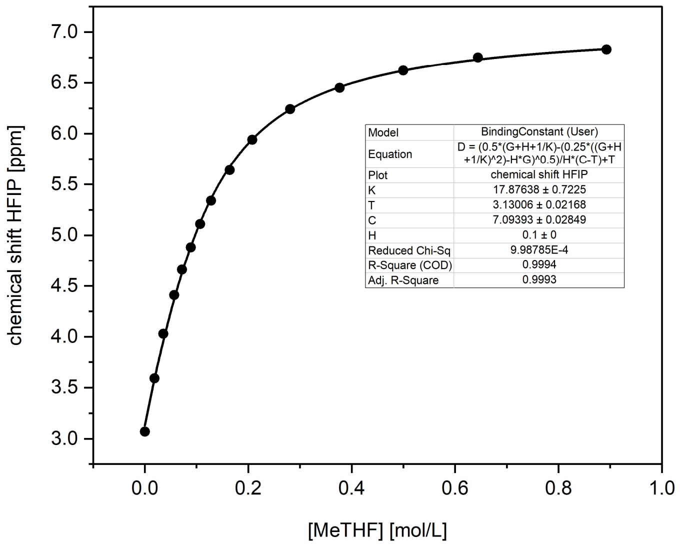

Figure S7. Binding isotherm of 2-MeTHF and HFIP at [HFIP] = 0.10 M host concentration. The found K is the binding constant, H is the host concentration [HFIP] = 0.10 M. T represents the associated ^{1}H NMR spectroscopic shift of HFIP and C that of the association complex.

*2-Methylfuran **3i***

HFIP (10.5 µL, 0.10 mmol, 1.00 eq.) was dissolved in 1.0 mL $CDCl_3$ (final concentration 0.10 M) in an NMR tube. A ^{1}H NMR spectrum was collected. Then, commercial 2-methyl furan **3i** was added in approximately 0.20 eq. portions (~ 2.0 µL, 0.020 mmol, 0.20 eq.). The mixture was mixed, and at every step, a ^{1}H NMR spectrum was collected. Based on the HFIP starting concentration $[HFIP]_0$, the relative [2-methylfuran] was calculated (Table S7).

Table S7. Derived relative [2-Methylfuran] and associated δ(HFIP) shift.

**Entry**	**[2-Methylfuran] [mol L^{-1}]**	**δ(HFIP)**
1	0.000	3.07
2	0.013	3.08
3	0.030	3.10
4	0.050	3.11
5	0.069	3.13
6	0.090	3.15
7	0.112	3.18
8	0.141	3.19
9	0.167	3.21
10	0.202	3.23
11	0.246	3.27
12	0.319	3.30
13	0.389	3.38
14	0.535	3.48
15	0.695	3.53
16	0.948	3.66
17	1.412	3.86
18	1.750	4.03

A good correlation (R^2 = 0.997) confirms this 1:1 binding model with a binding constant of K = 0.32 ± 0.05 mol L^{-1} between HFIP and 2-methylfuran (see Figure S8) although the titration endpoint was not found requiring significantly higher [2-Methylfuran] concentration.

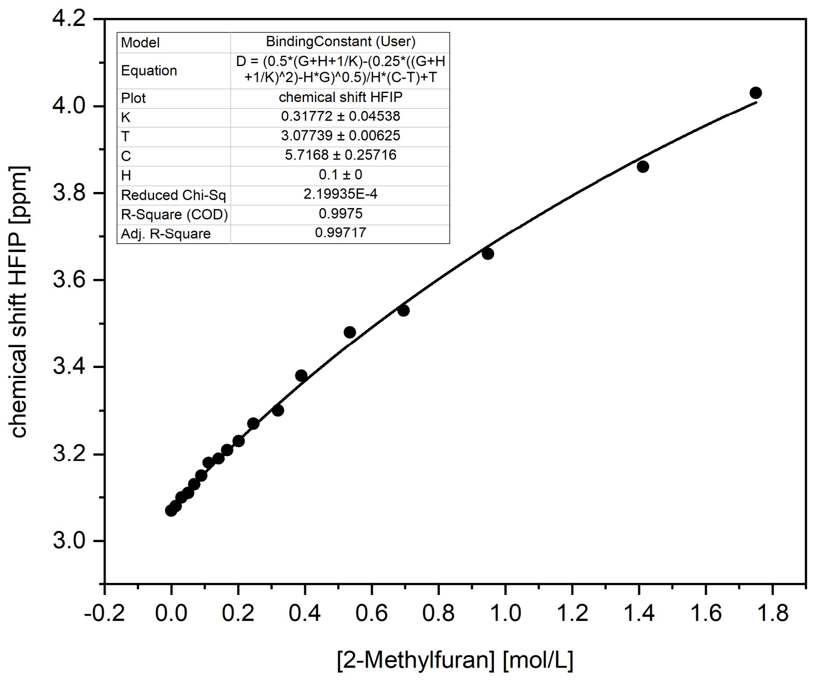

Figure S8. Binding isotherm of 2-methylfuran and HFIP at [HFIP] = 0.10 M host concentration. The found K is the binding constant, H is the host concentration [HFIP] = 0.10 M. T represents the associated ^{1}H NMR spectroscopic shift of HFIP and C that of the association complex.

Mechanism for 2-Substituted Furans

Partial reduction was not achieved for 2-substituted furans. Therefore, it is not clear whether the reaction mechanism proceeds through a 2,3- or 2,5-dihydrofuran intermediate. For that, the respective intermediates **S3** and **S4** were synthesized and subjected to the reaction conditions:

2-Pentyl-2,5-dihydrofuran **S3** remained unreactive under *Conditions (B)* whereas 2-hexyl-2,3-dihydrofuran **S4** reacted readily to the tetrahydrofuran product:

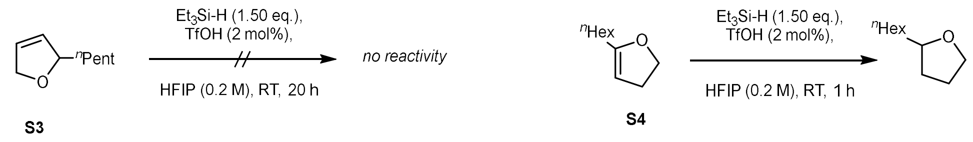

This finding is supported by computational results (further discussed on p. 152), which show that the 2,3-dihydrofuran intermediate for 2-substituted furan is thermodynamically more stable than the 2,5-dihydrofuran intermediate.

Catalytic Cycle for the Full Reduction

Figure S9 shows the second catalytic cycle for the second reduction step from dihydrofuran to tetrahydrofuran, being proposed to proceed analogously to the first reduction step. Upon benzylic protonation, the generated carbocation gets reduced through Et_3SiH, affording the final tetrahydrofuran product (Figure S9). It is to be expected that this catalytic cycle benefits less from HFIP stabilizing effects as all species bear an ether-like oxygen binding with similar affinities to HFIP.

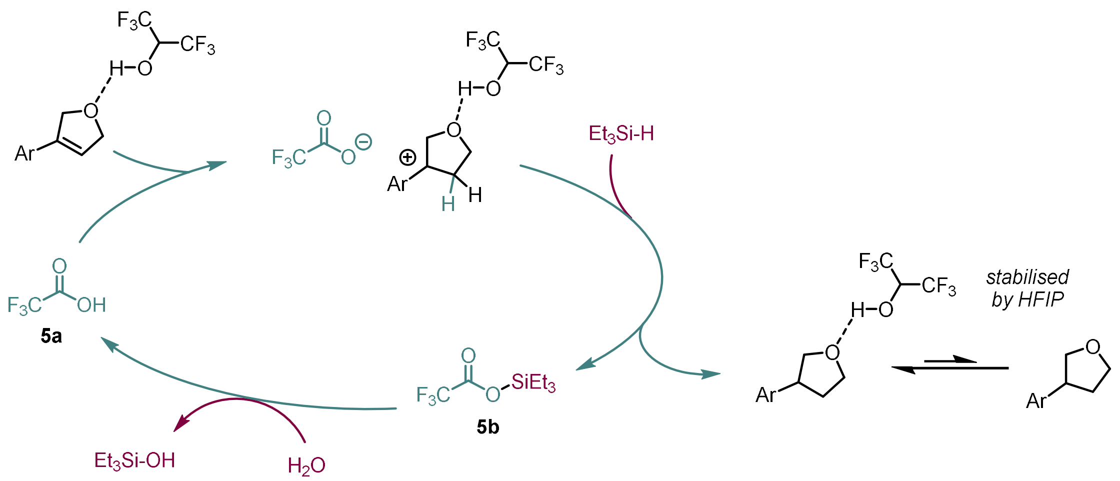

Figure S9. Proposed catalytic cycle for the second reduction.

Rationale for trans-Diastereoselectivies for 2,3-Disubstituted Furans

While stereochemical models for six-membered oxocarbenium ions adhere to pronounced steric and stereoelectronic preferences, five-membered oxocarbenium cations are influenced by more nuanced factors that determine diastereoselectivity. Pioneering work by Woerpel and co-workers established a model of "inside"-attack being the intrinsically favoured attacking trajectory on envelope-shaped furanosyl oxocarbenium ions avoiding a staggered product conformation. Furthermore, they attribute observed *trans*-selectivities to *cis*-repulsion between substituents in the product within the context of the Bell-Evans-Polanyi principle.[6]

To illustrate the here observed 1,2-*trans*-diastereoselectivity, the same line of arguments are followed. It can be speculated that the small size of the attacking hydride (on a triethylsilylium-backbone) might not affect steric repulsion towards the 3-substituent while attacking the oxocarbenium ion. Indeed, upon inspection of the computed furanosyl oxocarbenium ions structures (Figure S10), the 3-substituent is placed near-equatorial leading to minimal sterically interference with approaching nucleophiles towards the 2-position.

In contrast, the steric repulsion between the 2- and 3-substituent in the final tetrahydrofuran product leads to a more stable *trans*-product by 1.9 kcal mol^{-1} for **4j'** (simplified by the ^{t}Bu group) and 6.0 kcal mol^{-1} for **4k** at DLPNO-CCSD(T)/def2-TZVPP//B2PLYP-D3BJ/def-SVP level of theory (298 K / 1 M). Based on the Bell-Evans-Polanyi principle, this repulsion can partially be expected in the transition state too, as the substituents bent towards each other — kinetically disfavouring the production of the *cis*-product.

A more detailed computational study, similar to van Rijssel et al.,[7] would give more conclusive insight as the conformational landscape of substituted furanosyl-system is highly complex. Dynamic effects of the coordinating HFIP solvent can be expected, as well as acid strength (see p. 53), as observed for **4j**, which falls outside the scope of this study.

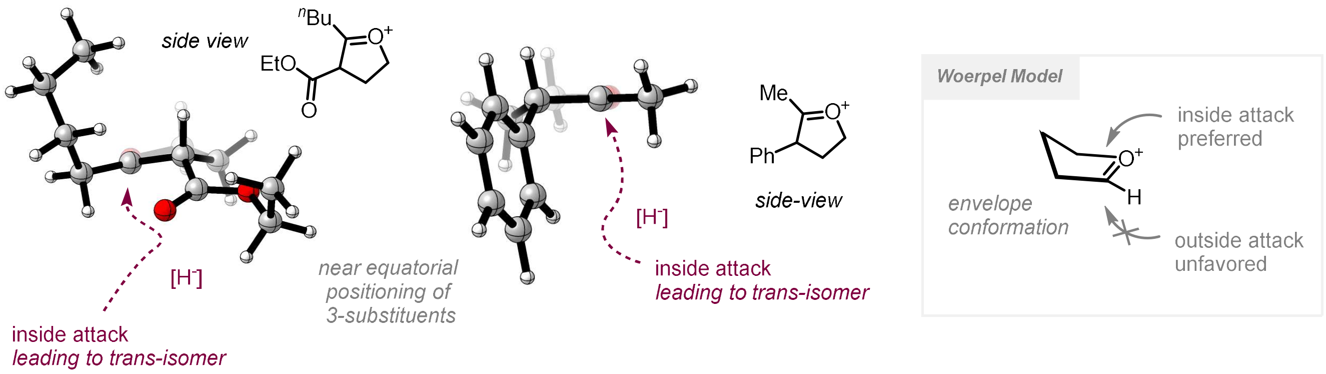

Figure S10. Kinetic arguments using Woerpel's "inside"-rule. The furanosyl oxocarbenium ions are depicted side on, in order to clearly identify the inside (envelope head direction) and outside half rooms. Structures computed at B2PLYP-D3BJ/def-SVP level of theory.

8. Computational Analysis

Computational Details

Quantum chemical calculations were run using the ORCA suite of programs (version 6.0).[8,9] All calculations used the resolution of the identity approximation (RIJCOSX),[10] with the appropriate auxiliary basis sets.[11] "VeryTight" optimisation criteria (10^{-9} Ha tolerance for SCF, $2 \cdot 10^{-7}$ Ha tolerance for optimisation step) were employed using the default grid.

If needed, the conformational space was sampled via CREST[12] GFN2-xTB[13] and the lowest-lying conformer used in further reoptimisation: Geometry optimizations were carried out using the double-hybrid functional B2PLYP[14], which has been shown to provide reliable predictions of geometries, rotational spectroscopic parameters, and vibrational properties.[15] The def2-TZVPP/C and def2-SVP/C correlation integrals were used as auxiliary basis set for the def2-TZVPP and def2-SVP basis sets, respectively.[11] The HFIP solvent was approximated through the implicit CPCM solvent model[16] using $\varepsilon = 16.7$ and a refractive index of 1.275. Additionally, the surfacetype VDW_GAUSSIAN was employed.

The Domain-based Local Pair Natural Orbital coupled cluster method with singles, doubles and perturbative triples (DLPNO-CCSD(T)) was used as a final single point providing reliable electronic energies using the def2-TZVPP basis set. DLPNO-CCSD(T) calculations were run using "NormalPNO" cut-offs. [17,18]

Vibrational frequencies were computed at the optimization level of theory to confirm whether the structures correspond to minima or transition states. All intermediate structures were verified to be minima by the absence of imaginary frequencies upon calculation of the Hessian. Grimme's quasi RRHO approach was used to calculate free energies at 298 K. A standard state correction from 1 atm to 1 M was applied by adding RTln(1/24.5) (T = 298 K) to the calculated free energy of each species. For calculating thermodynamic data, the python script *OTherm.py* (https://github.com/duartegroup/otherm) was used with $\omega_0 = 100$ cm^{-1} replacing harmonic oscillators with free-rotors below ω_0. All thermodynamic data forming the basis for the 2D plots in Figure 6 (main text) can be found in Table S9 (in Appendix, p. 155).

Introduction

Different substituted furans were analyzed computationally to explain the limitation of the substrate scope and the selectivity towards partial reduction to the 2,5-dihydrofuran products. As already outlined in the main text (Figure 5F), the mechanism consists of a protonation, then a reduction towards the dihydrofuran, and again a protonation and reduction further towards the tetrahydrofuran. As depicted in Figure S11, site-selectivity of the protonation and reduction is crucial in determining the thermodynamically most preferred intermediates. For the 3-substituted furans, the 2,5-dihydrofuran was experimentally found, whereas for most 2-substituted furans, the nature of the intermediate remains elusive. Therefore, different plausible possible intermediate isomers were computed (see Appendix p. 152).

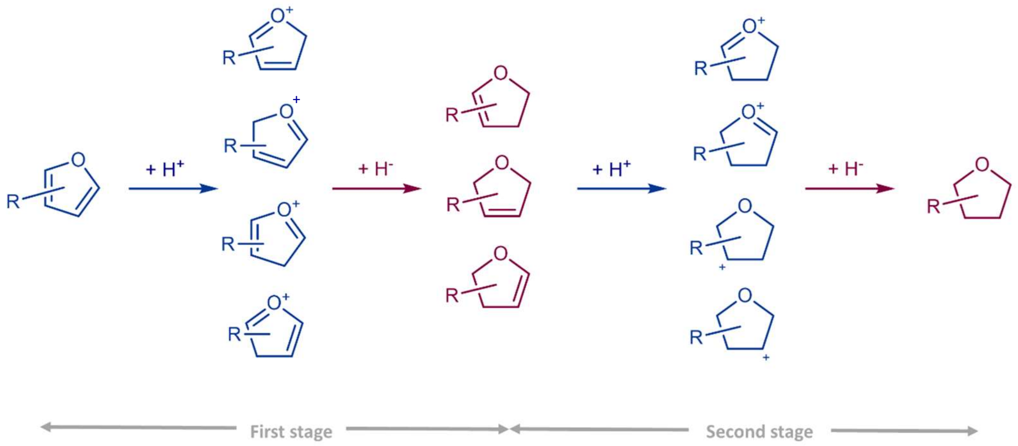

Figure S11. Schematic representation of the different isomeric intermediates towards 2,5-dihydrofurans (first stage) and tetrahydrofurans (second stages).

In general, protonation of furans happens at the C-position and not at the O-position as it would lead to dearomatization. This is widely supported in literature[19], our EXSY-NMR experiments (see p. 11) as well as computations shown in Figure S12.

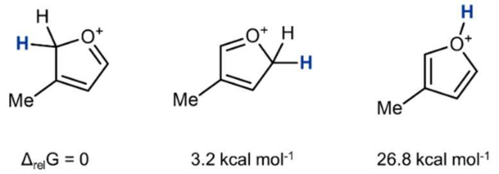

Figure S12. Relative Gibbs energy differences for different protonation sites at CPCM(HFIP)-DLPNO-CCSD(T)/def2-TZVPP//CPCM(HFIP)-B2PLYP-D3BJ/def2-SVP (298 K / 1 M) level of theory.

Relative Free Protonation and Hydride Energies

The Gibbs free energies of protonation $\Delta G^{I}(H^{+})$ and hydrid-addition $\Delta G^{I}(H^{-})$ based on reaction equations (I) and (II) were calculated for a diverse set of furans–using in each case the most preferred isomeric intermediates (see Figure S11 and S12). Comparing the relative Gibbs energies $\Delta G^{I}_{rel}(H^{+})$ and $\Delta G^{I}_{rel}(H^{-})$ to 3-phenylfuran **1d** (setting $\Delta G^{H+}_{rel} = 0$ and $\Delta G^{H-}_{rel} = 0$) makes comparison and rationalization easier.

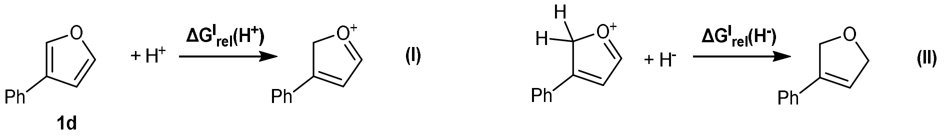

The data can be best interpreted in a 2D visualization, by plotting $\Delta G^{I}_{rel}(H^{+})$ and $\Delta G^{I}_{rel}(H^{-})$ on the x- and y-axes, respectively (see main text, Figure 6A). Analogously, a plot can be constructed for the second reduction step from the dihydrofuran to the tetrahydrofuran intermediate (see main text and Figure 6B). The Appendix (p. 152) provides an in-depth analysis of each molecule, including its preferred and less preferred isomers.

NBO Analysis

The HFIP-complex of **5f** was identified via a CREST GFN2-xTB (--NCI) conformer search and optimized at CPCM(HFIP)-B2PLYP-D3BJ/def2-SVP (298 K / 1 M) level of theory. The associated NBO output is displayed in Table S8.

Table S8. NBO output at CPCM(HFIP)-B2PLYP-D3BJ/def2-SVP (298 K / 1 M) level of theory.

from HFIP to **5f**		**E(2) [kcal mol^{-1}]**	**E(NL)-E(L) [a.u.]**	**F(L,NL) [a.u.]**
LP (2) O 2	RY (1) H 19	0.05	0.80	0.006
LP (2) O 2	RY (1) H 20	0.07	0.84	0.007
BD (1) H 1- O 2	BD*(1) C 12- O 16	0.32	1.17	0.017
BD (1) H 1- O 2	BD*(1) C 12- H 20	0.05	1.34	0.007
BD (1) H 1- O 2	BD*(1) C 15- O 16	0.32	1.17	0.017
BD (1) H 1- O 2	RY (1) C 12	0.06	1.57	0.008
BD (1) H 1- O 2	RY (2) O 16	0.16	2.44	0.017
BD (1) H 1- O 2	RY (1) H 20	0.07	1.23	0.008
From **5f** to HFIP				
LP (1) O 16	BD*(1) H 1- O 2	3.13	1.05	0.051
LP (2) O 16	BD*(1) H 1- O 2	28.61	1.02	0.153
LP (2) O 16	BD*(1) O 2- C 3	0.16	0.90	0.011
LP (2) O 16	RY (1) H 1	0.11	1.40	0.011
LP (2) O 16	RY (4) H 1	0.11	3.07	0.016
LP (2) O 16	RY (4) O 2	0.09	2.16	0.012
BD (1) C 12- O 16	BD*(1) H 1- O 2	0.30	1.37	0.018
BD (1) C 12- H 20	BD*(1) H 1- O 2	0.06	1.10	0.007
BD (1) C 12- H 20	RY (1) H 1	0.28	1.48	0.018
BD (1) C 15- O 16	BD*(1) H 1- O 2	0.24	1.37	0.016
BD (1) C 15- H 19	RY (1) H 1	0.26	1.48	0.017

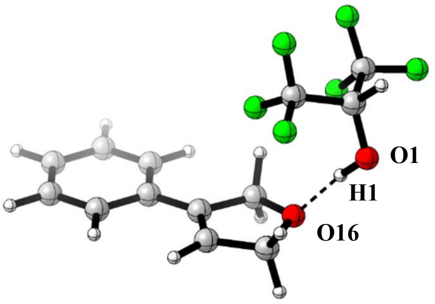

Electronic energy: -1250.093378527128

Number of imaginary frequencies: 0

H	0.93501759547744	-0.35144443645841	0.68927285282381
O	1.50418007022962	-0.07666421948529	1.45602392684370
C	2.73549779532104	0.34924095016584	0.98728510927660
C	3.48923230532366	-0.80405363027460	0.31691663514425
F	2.80351437444890	-1.31114675630441	-0.71913585714287
F	4.69289391390179	-0.42965689935910	-0.13406669456577
F	3.67089035026640	-1.79278344866453	1.19884693376611
C	2.59334914162604	1.58011374277386	0.08568992995700
F	1.93390409561019	2.54286384264752	0.73901140621054
F	3.78175468866627	2.07422156472395	-0.28259073754727
F	1.90141230582252	1.30657507706303	-1.03120574310950
C	-1.25153320836934	0.12523324353913	-0.47122646712294
C	-2.43659040338225	-0.77749516256949	-0.22151062748990
C	-2.01173040774904	-2.05507496342973	-0.21625263509723
C	-0.53773783993552	-2.12320049022552	-0.46034057286479
O	-0.11809613033944	-0.75042513544836	-0.50430401590772
H	3.35622401506803	0.66674960691906	1.83829811913565
H	-0.28859968896842	-2.62309128620750	-1.41356777905596
H	0.01484546027918	-2.63969631088559	0.34248526621866
H	-1.09794785961091	0.87011196809163	0.32652246824922
H	-1.34042160177177	0.66840862533407	-1.42819850178966
C	-6.20037270754530	-0.61689563314470	0.19646174278712
C	-4.91223463607351	-1.12267627500212	0.02421702018441
C	-3.80024992329704	-0.25813909713390	-0.04680514235520

C	-4.02604547896088	1.12842584301519	0.05234139499834
C	-5.31884475564481	1.63452678940367	0.22278248553100
C	-6.41062572085457	0.76526802681816	0.29698030518862
H	-7.04739087554072	-1.30369283176608	0.24851766872062
H	-4.76706340296374	-2.20058146974404	-0.06144490636518
H	-3.18719615611538	1.82414208158352	0.00153711651804
H	-5.47034512759414	2.71288843175209	0.29837453705883
H	-7.41992367702453	1.15908513748932	0.42948850630380
H	-2.62419374119967	-2.94474579104059	-0.07241694913757

Explicit Solvation

Protonated **5e** was pre-optimized at CPCM(HFIP)-B2PLYP-D3BJ/def2-SVP (298 K / 1 M) level of theory. Then it was subjected to ORCA SOLVATOR using 10 HFIP molecules employing ALPB(ETHANOL) as an approximate dielectric constant employing *Docker* algorithm at GFN2-xTB level of theory.[8] The obtained can be found in the raw data archive.

9. Preparation of Starting Material

General Procedure III–Synthesis of 3-arylfurans

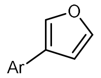

A Schlenk flask was placed under Argon and charged with the respective arylboronic acid (1.30 eq.), Cs_2CO_3 (1.40 eq.) and dissolved in dry toluene/methanol (4:1, *v:v*, final concentration 0.10 M). The suspension was degassed for 10 minutes by bubbling argon through the reaction medium. 3-Bromofuran (1.00 eq.) was added via syringe and $Pd(PPh_3)_4$ (5 mol%) was added as a solid. The Schlenk flask was closed, evacuated three times, and then heated to 105 °C for 12 h. Finally, the reaction mixture was allowed to cool to room temperature and quenched with saturated NH_4Cl solution. The aqueous phase was extracted twice with ethyl acetate. The combined organic phases were washed with brine, dried over $MgSO_4$, filtered, and concentrated under reduced pressure. The crude product was purified by flash column chromatography to afford the pure product.

General Procedure IV–Synthesis of 3-alkyl furans

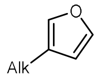

Preparation of the Grignard reagent

A Schlenk flask was placed under Argon and charged with Magnesium pellets (1.20 eq.) and dry Et_2O (0.5–1.0 M). An iodine crystal was added, followed by the addition of the alkyl bromide (1.00 eq.) under rapid stirring. Within a few minutes, the Grignard formation initiated and was allowed to stir for further 4 h. The concentration of the resulting Grignard solution was determined with a *Metrohm 859 Titrotherm* and used directly in the next step:

Kumada coupling

A Schlenk flask was placed under Argon and charged with $Ni(dppe)Cl_2$ (5 mol%) and dry THF (0.33 M). 3-Bromofuran was added dropwise via syringe. Under stirring, the Grignard solution was added dropwise over a period of 5 minutes. The reaction mixture was stirred for 4 h at room temperature and then quenched with NH_4Cl solution (aq. sat.). The aqueous phase was extracted with Et_2O two more times. The combined organics were washed with brine, dried over $MgSO_4$, filtered, and concentrated under reduced pressure. The crude product was purified by flash column chromatography to afford the pure product.

General Procedure V–Synthesis of 2-aryl furans

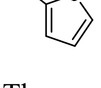

The respective aryl bromide (1.00 eq.), furan-2-boronic acid (1.50 eq.), and Cs_2CO_3 (1.10 eq.) were dissolved in a dry toluene/methanol (4:1, *v:v*, final concentration 0.10 M). The suspension was degassed for 10 minutes by bubbling Argon through the reaction medium. Then, $Pd(PPh_3)_4$ (5 mol%) was added as a solid. The Schlenk flask was closed and heated to 105 °C for 12 h. The mixture was diluted with NH_4Cl (aq. sat.) and extracted with EtOAc twice. The combined organics were washed with brine, dried over $MgSO_4$, filtered, and concentrated under reduced pressure. The crude product was purified by flash column chromatography to afford the pure product.

3-(4-Methoxyphenyl)furan, 1a / 3a

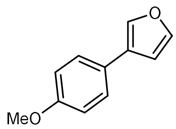

3-Bromofuran (61 µL, 0.68 mmol, 1.00 eq.), Cs_2CO_3 (310 mg, 0.95 mmol, 1.40 eq.), 4-methoxyphenylboronic acid (134 mg, 0.88 mmol, 1.30 eq.) and $Pd(PPh_3)_4$ (39 mg, 0.034 mmol, 5 mol%) in dry toluene (5.4 mL) and dry MeOH (1.4 mL) were subjected to *General Procedure III.* Purification by flash column chromatography (SiO_2, 5% Et_2O in *n*-pentane) afforded **1a** / **3a** (71 mg, 0.41 mmol, 60%) as a white solid.

R*f* 0.36 (*n*-pentane / EtOAc, 95:5).

^{1}H NMR (501 MHz, $CDCl_3$) δ 7.66–7.65 (m, 1H), 7.45 (t, *J* = 1.7 Hz, 1H), 7.43–7.40 (m, 2H), 6.96–6.90 (m, 2H), 6.65 (dd, *J* = 1.9, 0.9 Hz, 1H), 3.83 (s, 3H).

^{13}C NMR (126 MHz, $CDCl_3$) δ 158.9, 143.7, 137.8, 127.2, 126.2, 125.2, 114.4, 109.0, 55.5.

HRMS m/z (GC-EI): calculated for $C_{11}H_{10}O_2$ $[M]^+$ 174.06753; found 174.06768.

3-(4-(*tert*-Butyl)phenyl)furan, 1b / 3b

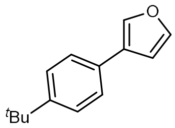

3-Bromofuran (245 µL, 2.72 mmol, 1.00 eq.), Cs_2CO_3 (1.24 g, 3.81 mmol, 1.40 eq.), 4-*tert*-butylphenylboronic acid (630 mg, 3.54 mmol, 1.30 eq.) and $Pd(PPh_3)_4$ (156 mg, 0.14 mmol, 5 mol%) in dry toluene (22 mL) and dry MeOH (5.4 mL) were subjected to *General Procedure III.* Purification by flash column chromatography (SiO_2, 100% hexanes) afforded **1b** / **3b** (163 mg, 0.82 mmol, 30%) as a white solid.

R*f* 0.69 (100% *n*-pentane).

^{1}H NMR (501 MHz, $CDCl_3$) δ 7.73–7.69 (app t, 1H), 7.47 (t, *J* = 1.7 Hz, 1H), 7.45–7.39 (m, 4H), 6.71–6.67 (m, 1H), 1.35 (s, 9H).

^{13}C NMR (126 MHz, $CDCl_3$) δ 150.2, 143.7, 138.4, 129.7, 126.5, 125.9, 125.8, 109.1, 34.7, 31.5.

HRMS m/z (GC-EI): calculated for $C_{14}H_{16}O_1$ $[M]^+$ 200.11957; found 200.11987.

3-(*para*-Tolyl)furan, 1c / 3c

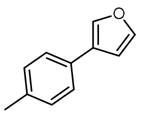

3-Bromofuran (245 µL, 2.72 mmol, 1.00 eq.), Cs_2CO_3 (1.24 g, 3.81 mmol, 1.40 eq.), 4-methylphenylboronic acid (480 mg, 3.54 mmol, 1.30 eq.) and $Pd(PPh_3)_4$ (156 mg, 0.14 mmol, 5 mol%) in dry toluene (22.0 mL) and

dry MeOH (5.40 mL) were subjected to *General Procedure III*. Purification by flash column chromatography (SiO_2, 100% hexanes) afforded **1c** / **3c** (130 mg, 0.82 mmol, 30%) as a white solid.

R$_f$ 0.40 (100% *n*-pentane).

^{1}H NMR (501 MHz, $CDCl_3$) δ 7.71 (br t, *J* = 1.2 Hz, 1H), 7.48 (t, *J* = 1.7 Hz, 1H), 7.42–7.38 (m, 2H), 7.21 (app d, *J* = 0.7 Hz, 2H), 6.70 (dd, *J* = 1.9, 0.9 Hz, 1H), 2.38 (s, 3H).

^{13}C NMR (126 MHz, $CDCl_3$) δ 143.7, 138.3, 136.8, 129.7, 129.6, 126.5, 125.9, 109.0, 21.3.

HRMS m/z (GC-EI): calculated for $C_{11}H_{10}O_1$ $[M]^+$ 158.07262; found: 158.07256.

3-Phenylfuran, 1d / **3d**

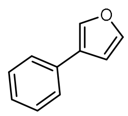

3-Bromofuran (245 µL, 2.72 mmol, 1.00 eq.), Cs_2CO_3 (1.24 g, 3.81 mmol, 1.40 eq.), phenylboronic acid (431 mg, 3.54 mmol, 1.30 eq.) and $Pd(PPh_3)_4$ (156 mg, 0.14 mmol, 5 mol%) in dry toluene (22 mL) and dry MeOH (5.4 mL) were subjected to *General Procedure III*. Purification by flash column chromatography (SiO_2, 100% hexanes) afforded **1d** / **3d** (210 mg, 1.46 mmol, 53%) as a white solid.

R$_f$ 0.50 (*n*-pentane / EtOAc, 95:5).

^{1}H NMR (501 MHz, $CDCl_3$) δ 7.75–7.71 (app t, 1H), 7.51–7.47 (m, 3H), 7.40–7.35 (m, 2H), 7.29–7.25 (m, 1H), 6.73–6.69 (m, 1H).

^{13}C NMR (126 MHz, $CDCl_3$) δ 143.8, 138.6, 132.6, 129.0, 127.1, 126.6, 126.0, 109.0.

HRMS m/z (GC-EI): calculated for $C_{10}H_8O_1$ [M]+ 144.05697; found: 144.05723.

3-(4-Chlorophenyl)furan, 1e / **3e**

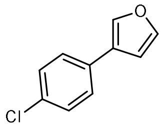

3-Bromofuran (61 µL, 0.68 mmol, 1.00 eq.), Cs_2CO_3 (310 mg, 0.95 mmol, 1.40 eq.), 4-chlorophenylboronic acid (138 mg, 0.88 mmol, 1.30 eq.) and $Pd(PPh_3)_4$ (39 mg, 0.034 mmol, 5 mol%) in dry toluene (5.4 mL) and dry MeOH (1.4 mL) were subjected to *General Procedure III*. Purification by flash column chromatography (SiO_2, 5% Et_2O in *n*-pentane) afforded **1e** / **3e** (49 mg, 0.27 mmol, 40%) as a white solid.

R$_f$ 0.44 (100% *n*-pentane).

^{1}H NMR (501 MHz, $CDCl_3$) δ 7.74–7.70 (app. t, 1H), 7.48 (t, *J* = 1.7 Hz, 1H), 7.43–7.39 (m, 2H), 7.36–7.33 (m, 2H), 6.69–6.65 (m, 1H).

^{13}C NMR (126 MHz, $CDCl_3$) δ 144.0, 138.7, 132.8, 131.1, 129.1, 127.2, 125.6, 108.8.

HRMS m/z (GC-EI): calculated for $C_{10}H_7O_1Cl$ $[M]^+$ 178.01799; found: 178.01819.

3-(4-(Trifluoromethyl)phenyl)furan, 1f / 3f

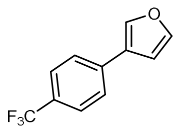

3-Bromofuran (245 µL, 2.72 mmol, 1.00 eq.), Cs_2CO_3 (1.24 g, 3.81 mmol, 1.40 eq.), 4-(trifluoromethyl)-phenylboronic acid (672 mg, 3.54 mmol, 1.30 eq.) and $Pd(PPh_3)_4$ (156 mg, 0.14 mmol, 5 mol%) in dry toluene (22.0 mL) and dry MeOH (5.40 mL) were subjected to *General Procedure III.* Purification by flash column chromatography (SiO_2, Et_2O in *n*-pentane, gradient 0% to 10%) afforded **1f** / **3f** (350 mg, 1.65 mmol, 61%) as a white solid.

R*f* 0.44 (100% *n*-pentane).

^{1}H NMR (501 MHz, $CDCl_3$) δ 7.80 (br t, *J* = 1.2 Hz, 1H), 7.63 (app d, *J* = 8.0 Hz, 2H), 7.58 (app d, *J* = 8.3 Hz, 2H), 7.52 (t, *J* = 1.7 Hz, 1H), 6.73 (dd, *J* = 1.8, 0.9 Hz, 1H).

^{13}C NMR (126 MHz, $CDCl_3$) δ 144.3, 139.5, 136.2, 129.1 (q, *J* = 32.6 Hz), 126.1, 125.9 (q, *J* = 3.8 Hz), 125.6, 124.4 (q, *J* = 271.8 Hz), 108.8.

^{19}F NMR (471 MHz, $CDCl_3$) δ −62.5.

HRMS m/z (GC-EI): calculated for $C_{11}H_7O_1F_3$ $[M]^+$ 212.04435; found: 212.04410.

3-(4-Nitrophenyl)furan, 1g / 3g

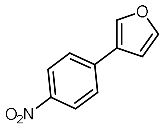

3-Bromofuran (61 µL, 0.68 mmol, 1.00 eq.), Cs_2CO_3 (310 mg, 0.95 mmol, 1.40 eq.), 4-nitrophenylboronic acid (147 mg, 0.88 mmol, 1.30 eq.) and $Pd(PPh_3)_4$ (39 mg, 0.034 mmol, 5 mol%) in dry toluene (5.40 mL) and dry MeOH (1.40 mL) were subjected to *General Procedure III.* Purification by flash column chromatography (SiO_2, 5% Et_2O in *n*-pentane) afforded **1g** / **3g** (68 mg, 0.36 mmol, 53%) as a yellow solid.

R*f* 0.29 (*n*-pentane / Et_2O, 95:5).

^{1}H NMR (501 MHz, $CDCl_3$) δ 8.26–8.22 (m, 2H), 7.87 (br t, *J* = 1.2 Hz, 1H), 7.64–7.61 (m, 2H), 7.54 (t, *J* = 1.7 Hz, 1H), 6.77–6.73 (m, 1H).

^{13}C NMR (126 MHz, $CDCl_3$) δ 146.7, 144.7, 140.4, 139.3, 126.3, 125.0, 124.5, 108.7.

HRMS m/z (GC-EI): calculated for $C_{10}H_7N_1O_3$ $[M]^+$ 189.04204; found: 189.04236.

3-(Cyclohexylmethyl)furan, 1h

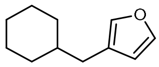

3-Bromofuran (225 µL, 2.50 mmol, 1.00 eq.), Ni(dppe)Cl_2 (66 mg, 0.13 mmol, 5.0 mol%), (cyclohexylmethyl)-magnesiumbromid (7.50 mL, 3.75 mmol, 1.50 eq., 0.50 M in Et_2O) and dry THF (7.50 mL) were subjected to *General Procedure IV*. Purification by flash column chromatography (SiO_2, 100% *n*-pentane) afforded **1h** (50 mg, 0.30 mmol, 12%) as a colorless oil.

R*f* 0.61 (100% *n*-pentane).

^{1}H NMR (501 MHz, $CDCl_3$) δ 7.34 (m, 1H), 7.21–7.16 (m, 1H), 6.24 (m, 1H), 2.29 (d, *J* = 7.0 Hz, 2H), 1.75–1.67 (m, 4H), 1.67–1.62 (m, 1H), 1.43 (m, 1H), 1.28–1.08 (m, 3H), 0.91 (m, 2H).

^{13}C NMR (126 MHz, $CDCl_3$) δ 142.6, 139.5, 123.7, 111.7, 38.7, 33.3, 32.7, 26.7, 26.4.

HRMS m/z (GC-EI): calculated for $C_{11}H_{16}O_1$ $[M]^+$ 164.11957; found: 164.11971.

3-Heptylfuran, 1i

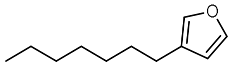

3-Bromofuran (225 µL, 2.50 mmol, 1.00 eq.), Ni(dppe)Cl_2 (66 mg, 0.13 mmol, 5.0 mol%), heptyl magnesium bromide solution (5.00

mL, 5.00 mmol, 2.00 eq., 1.0 M in Et_2O) and dry THF (7.50 mL) were subjected to *General Procedure IV*. Purification by flash column chromatography (SiO_2, 100% hexanes) afforded **1i** (245 mg, 1.47 mmol, 59%) as a colorless oil.

R*f* 0.53 (100% hexanes).

^{1}H NMR (501 MHz, $CDCl_3$) δ 7.36–7.32 (m, 1H), 7.22–7.18 (m, 1H), 6.29–6.26 (m, 1H), 2.42–2.38 (m, 2H), 1.59–1.51 (m, 2H), 1.36–1.23 (m, 8H), 0.99–0.84 (m, 3H).

^{13}C NMR (126 MHz, $CDCl_3$) δ 142.7, 138.9, 125.5, 111.2, 32.0, 30.2, 29.4, 29.3, 24.9, 22.8, 14.2.

HRMS m/z (GC-EI): calculated for $C_{11}H_{18}O_1$ $[M]^+$ 166.13522; found: 166.13544.

3-Cyclobutylfuran, 1j

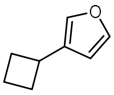

3-Bromofuran (225 µL, 2.50 mmol, 1.00 eq.), Ni(dppe)Cl_2 (66 mg, 0.13 mmol, 5 mol%), cyclobutylmagnesiumbromid (10.0 mL, 5.00 mmol, 2.00 eq., 0.50 M in Et_2O) and dry THF (7.50 mL) were

subjected to *General Procedure IV*. Purification by flash column chromatography (SiO_2, 100% *n*-pentane) afforded **1j** (80 mg, 0.66 mmol, 26%) as a colorless oil.

R*f* 0.46 (100% *n*-pentane).

^{1}H NMR (501 MHz, $CDCl_3$) δ 7.34 (t, *J* = 1.7 Hz, 1H), 7.21 (m, 1H), 6.32 (m, 1H), 3.43–3.29 (m, 1H), 2.34–2.22 (m, 2H), 2.07–1.91 (m, 3H), 1.91–1.83 (m, 1H).

^{13}C NMR (126 MHz, $CDCl_3$) δ 143.0, 138.0, 130.2, 109.8, 31.5, 30.0, 18.9.

HRMS m/z (GC-EI): calculated for $C_8H_{10}O_1$ $[M]^+$ 122.07262; found: 122.07279.

3-(3-Methoxypropyl)furan, 1k

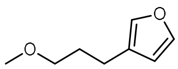

3-Bromofuran (225 µL, 2.50 mmol, 1.00 eq.), Ni(dppe)Cl_2 (66 mg, 0.13 mmol, 5.0 mol%), (3-methoxypropyl)magnesium bromide solution (5 mL, 5 mmol, 2.00 eq., 1.0 M in Et_2O) and dry Et_2O (7.50 mL) were subjected to *General Procedure IV*. Purification by flash column chromatography (SiO_2, 5% Et_2O in *n*-pentane) afforded **1k** (50 mg, 0.36 mmol, 14%) as a yellow oil.

R*f* 0.31 (*n*-pentane / Et_2O, 95:5).

^{1}H NMR (501 MHz, $CDCl_3$) δ 7.36–7.34 (app t, 1H), 7.24–7.20 (m, 1H), 6.27 (m, 1H), 3.39 (t, *J* = 6.4 Hz, 2H), 3.34 (s, 3H), 2.52–2.46 (app t, 2H), 1.87–1.78 (m, 2H).

^{13}C NMR (126 MHz, $CDCl_3$) δ 142.9, 139.1, 124.7, 111.1, 72.1, 58.7, 30.1, 21.4.

HRMS m/z (GC-EI): calculated for $C_8H_{12}O_2$ $[M]^+$ 140.08318; found: 140.08329.

3-(Chloromethyl)furan, 1m

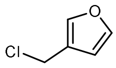

According to a procedure by Tanis[20]

3-Furanmethanol (1.72 mL, 20.0 mmol, 1.00 eq.) was mixed with 2,4,6-trimethylpyridin (2.90 mL, 22.0 mmol, 1.10 eq.). Under vigorous stirring, a suspension of LiCl (848 mg, 20.0 mmol, 1.00 eq.) in dry DMF (10.0 mL) was added and the mixture cooled to 0 °C. Then, MsCl (1.70 mL, 22.0 mmol, 1.10 eq.) was added dropwise. The mixture was allowed to stir for 4 h at 0 °C. Finally, the mixture was poured into ice water. The layers were separated, and the aqueous layer was extracted with a cold Et_2O/*n*-pentane (1:1, *v*:*v*) mixture. The combined organics were washed with $CuSO_4$ solution (aq. sat.) three times. The organics were washed with brine, dried over $MgSO_4$, filtered, and concentrated under reduced pressure. Purification by flash column chromatography (SiO_2, 100% *n*-pentane) afforded **1m** (560 mg, 4.81 mmol, 24%) as a colorless oil.

Note: The compound is very volatile.

R*f* 0.27 (100% *n*-pentane).

^{1}H NMR (501 MHz, $CDCl_3$) δ 7.47–7.45 (m, 1H), 7.42–7.40 (m, 1H), 6.47–6.45 (m, 1H), 4.49 (s, 2H).

^{13}C NMR (126 MHz, $CDCl_3$) δ 143.9, 140.9, 122.5, 110.5, 37.2.

HRMS m/z (GC-EI): calculated for $C_5H_5O_1Cl_1$ $[M]^+$ 116.00234; found: 116.00252.

3-(But-3-en-1-yl)furan, 1n

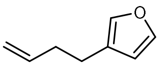

According to a procedure by New et al.[21]

To a solution of 3-furanmethanol (0.88 mL, 10.2 mmol, 1.00 eq.) in dry THF (10.0 mL) was added PBr_3 (0.34 mL, 3.57 mmol, 0.35 eq.) dropwise at 0 °C. The reaction was stirred for 2.5 h at 0 °C. Then, the mixture was quenched with distilled H_2O, and the mixture was extracted with Et_2O twice. The combined ethereal layers were washed with $NaHCO_3$ (aq. sat.) and brine, dried over $MgSO_4$, filtered, and concentrated under reduced pressure.

This crude material was redissolved in dry THF (30 mL) and cooled to 0 °C. To this, allylmagnesium bromide solution (22.4 mL, 22.4 mmol, 2.20 eq., 1.0 M in Et_2O) was added dropwise. The reaction mixture was allowed to warm up to room temperature and stirred for further 19 h. Finally, the mixture was quenched with aqueous H_2SO_4 (10.0 mL, 2.0 M) at 0 °C. The crude mixture was diluted with distilled H_2O and extracted with Et_2O twice. The combined etheral layers were washed with $NaHCO_3$ (aq. sat.) and brine, dried over $MgSO_4$, filtered, and concentrated under reduced pressure. Purification by flash column chromatography (SiO_2, 100% *n*-pentane) afforded **1n** (780 mg, 6.39 mmol, 63%) as a colorless oil.

R*f* 0.41 (100% *n*-pentane).

^{1}H NMR (501 MHz, $CDCl_3$) δ 7.35 (app t, 1H), 7.23 (m, 1H), 6.30–6.27 (m, 1H), 5.86 (ddt, *J* = 6.5, 10.2, 16.9 Hz, 1H), 5.06 (dq, *J* = 1.7, 17.2 Hz, 1H), 5.00 (ddt, *J* = 1.3, 2.0, 10.2 Hz, 1H), 2.55–2.50 (m, 2H), 2.36–2.28 (m, 2H).

^{13}C NMR (126 MHz, $CDCl_3$) δ 142.8, 139.0, 138.2, 124.7, 115.2, 111.1, 34.2, 24.5.

HRMS m/z (GC-EI): calculated for $C_8H_{10}O_1$ $[M]^+$ 122.07262; found: 122.07280.

3-(4-Methylpent-3-en-1-yl)furan *Perillene*, 1o

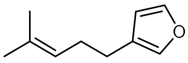

According to a procedure by Sun et al. [22]

A flame-dried Schlenk-flask was charged with dry THF (2.20 mL) under inert conditions and *n*-BuLi solution (2.2 mL, 5.50 mmol, 1.10 eq., 2.5 M in hexanes) was added. To this, 3-bromofuran (0.45 mL, 5.00 mmol, 1.00 eq.) was added dropwise over 15 minutes at −78 °C. After stirring for 2 h at −78 °C, NaI (824 mg, 5.50 mmol, 1.10 eq.) was added as a solid. Then, a solution of 5-bromo-2-methylpent-2-ene (0.63 mL, 5.00 mmol, 1.00 eq.) and HMPA (0.96 mL, 5.50 mmol, 1.10 eq.) in dry THF (1.0 mL) was added. The reaction mixture was allowed to stir for 4 days at −78 °C under inert conditions. Finally, the mixture was diluted with Et_2O and quenched with NH_4Cl (aq. sat.). The organic layer was separated, and the aqueous layer was washed with Et_2O twice. The combined organics were washed with brine, dried over $MgSO_4$, filtered, and concentrated under reduced pressure. Purification by flash column chromatography (SiO_2, 100% *n*-pentane) afforded **1o** (180 mg, 1.20 mmol, 24%) as a colorless oil.

R*f* 0.39 (100% *n*-pentane).

^{1}H NMR (600 MHz, $CDCl_3$) δ 7.35–7.33 (m, 1H), 7.21 (m, 1H), 6.28 (m, 1H), 5.15 (m, 1H), 2.46–2.41 (m, 2H), 2.30–2.19 (m, 2H), 1.69 (m, 3H), 1.61–1.58 (m, 3H).

^{13}C NMR (151 MHz, $CDCl_3$) δ 142.7, 139.0, 132.3, 125.1, 124.0, 111.2, 28.7, 25.8, 25.2, 17.9.

HRMS m/z (GC-EI): calculated for $C_{10}H_{14}O_1$ $[M]^+$ 150.10392; found: 150.10409.

Methyl 2-(4-methylfuran-2-yl)acetate, 1q

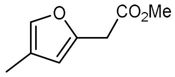

According to a procedure by Chen et al.[23]

(*Z*)-3-Iodo-2-propen-1-ol **S1** (396 mg, 2.00 mmol, 1.00 eq.) was dissolved in dry DCM (10.0 mL) under an inert atmosphere. PBu_3 (99 µL, 0.40 mmol, 20 mol%) was added, followed by the dropwise addition of methyl propiolate (0.27 mL, 3.00 mmol, 1.50 eq.) at room temperature. This mixture was stirred at room temperature for 1 h. Subsequently, the mixture was concentrated, re-dissolved in dry MeCN (10.0 mL), and transferred to a flask charged with $Pd(OAc)_2$ (45 mg, 0.20 mmol, 10 mol%) and tetrabutylammonium chloride (556 mg, 2.00 mmol, 1.00 eq.) under an inert atmosphere. Triethylamine (1.45 mL, 10.4 mmol, 5.20 eq.) was added and the mixture refluxed at 90 °C for 3 h. Finally, the mixture was allowed to cool to room temperature and then concentrated under reduced pressure. Purification of the residue by flash column chromatography (SiO_2, 5% Et_2O in *n*-pentane) afforded **1q** (200 mg, 1.30 mmol, 65%) as a yellow oil.

R_f 0.24 (*n*-pentane / EtOAc, 95:5).

^{1}H NMR (501 MHz, $CDCl_3$) δ 7.12 (app p, J = 1.2 Hz, 1H), 6.08 (s, 1H), 3.72 (s, 3H), 3.65–3.62 (br s, 2H), 2.00 (d, J = 1.2 Hz, 3H).

^{13}C NMR (126 MHz, $CDCl_3$) δ 170.1, 147.6, 138.9, 121.0, 110.9, 52.4, 34.2, 9.9.

HRMS m/z (GC-EI): calculated for $C_8H_{10}O_3$ $[M]^+$ 154.06245; found: 154.06255.

3-(4-(*tert*-Butyl)phenyl)-2-methylfuran, 3j

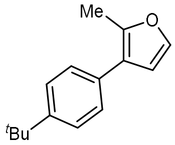

A Schlenk flask was placed under Argon and charged with 4-*tert*-butylphenylboronic acid (111 mg, 0.62 mmol, 1.15 eq.). Dry 1,4-dioxane (1.50 mL) and aqueous Na_2CO_3 solution (2 M, 0.54 mL) was added. The mixture was degassed for 10 minutes by bubbling argon through the reaction medium. 3-Bromo-2-methylfuran (87 mg, 0.54 mmol, 1.00 eq.) and $Pd(PPh_3)_4$ (19 mg, 0.016 mmol, 3.0 mol%) was added as a solid. The Schlenk flask was closed and heated to 130 °C for 12 h. Finally, the reaction mixture was allowed to cool to room temperature and quenched with distilled water. The aqueous phase was extracted twice with ethyl acetate. The combined organic phases were washed with brine, dried over $MgSO_4$, filtered, and concentrated under reduced pressure. Purification by flash column chromatography (SiO_2, EtOAc in *n*-pentane, gradient 0 to 5%) afforded **3j** (83 mg, 0.39 mmol, 72%) as a colorless oil.

R$_f$ 0.38 (100% *n*-pentane).

^{1}H NMR (501 MHz, $CDCl_3$) δ 7.45–7.41 (m, 2H), 7.36–7.33 (m, 2H), 7.32 (d, J = 2.0 Hz, 1H), 6.52 (d, J = 1.9 Hz, 1H), 2.46 (s, 3H), 1.35 (s, 9H).

^{13}C NMR (126 MHz, $CDCl_3$) δ 149.3, 147.7, 140.3, 131.4, 127.3, 125.6, 120.8, 111.4, 34.6, 31.5, 13.2.

HRMS m/z (GC-EI): calculated for $C_{15}H_{18}O_1$ $[M]^+$ 214.13522; found: 214.13542.

Ethyl 2-butylfuran-3-carboxylate, 3k

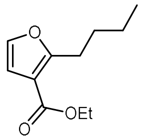

A mixture of ethyl-3-oxoheptanoat (1.00 g, 5.81 mmol, 1.00 eq.) and chloroacetaldehyde (1.15 mL, 8.71 mmol, 1.50 eq., 50% aq. solution) in pyridine (1.70 mL) was stirred at 50 °C under an inert atmosphere for 24 h. Finally, the reaction mixture was allowed to cool down and then diluted with ether. The organics were washed with HCl (10% aq.) twice. The organic layer was dried over $MgSO_4$, filtered and the solvents removed under reduced pressure. Purification of the residue by flash column chromatography (SiO_2, 5% Et_2O in *n*-pentane) afforded **3k** (350 mg, 1.78 mmol, 31%) as a colorless oil.

R$_f$ 0.37 (100% *n*-pentane).

^{1}H NMR (501 MHz, $CDCl_3$) δ 7.23 (d, J = 2.0 Hz, 1H), 6.63 (d, J = 2.0 Hz, 1H), 4.28 (q, J = 7.1 Hz, 2H), 2.98 (t, 2H), 1.71–1.57 (m, 2H), 1.43–1.29 (m, 5H, *overlapping signals*), 0.92 (t, J = 7.4 Hz, 3H).

^{13}C NMR (126 MHz, $CDCl_3$) δ 164.2, 163.4, 140.4, 113.3, 110.8, 60.2, 30.2, 27.5, 22.5, 14.5, 13.9.

HRMS m/z (GC-EI): calculated for $C_{11}H_{16}O_3$ $[M]^+$ 196.10940; found: 196.10944.

4-(Furan-2-yl)benzonitrile, 3n

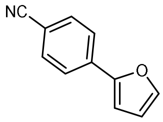

4-Bromobenzonitrile (364 mg, 2.00 mmol, 1.00 eq.), Cs_2CO_3 (717 mg, 2.20 mmol, 1.10 eq.), furan-2-boronic acid (336 mg, 3.00 mmol, 1.50 eq.) and $Pd(PPh_3)_4$ (116 mg, 0.10 mmol, 5 mol%) in dry toluene (16.0 mL) and dry MeOH (4.0 mL) were subjected to *General Procedure V.* Purification by flash column chromatography (SiO_2, Et_2O in *n*-pentane, gradient 5% to 10%) afforded **3n** (262 mg, 1.55 mmol, 77%) as a white powder.

R$_f$ 0.38 (*n*-pentane / Et_2O, 80:20).

^{1}H NMR (501 MHz, $CDCl_3$) δ 7.74 (d, J = 8.3 Hz, 2H), 7.69–7.63 (m, 2H), 7.56–7.49 (m, 1H), 6.81 (d, J = 3.4 Hz, 1H), 6.56–6.51 (m, 1H).

^{13}C NMR (126 MHz, $CDCl_3$) δ 152.1, 143.8, 134.8, 132.7, 124.1, 119.1, 112.4, 110.5, 108.3.

HRMS m/z (GC-EI): calculated for $C_{11}H_7N_1O_1$ $[M]^+$ 169.05221; found: 169.05213.

2-(4-Fluorophenyl)furan, 3o

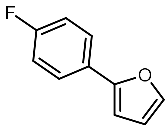

1-Bromo-4-fluorobenzene (0.22 mL, 2.00 mmol, 1.00 eq.), Cs_2CO_3 (717 mg, 2.20 mmol, 1.10 eq.), furan-2-boronic acid (336 mg, 3.00 mmol, 1.50 eq.) and $Pd(PPh_3)_4$ (116 mg, 0.10 mmol, 5 mol%) in dry toluene (16.0 mL) and dry MeOH (4.0 mL) were subjected to *General Procedure V.* Purification by flash column chromatography (SiO_2, 100% *n*-pentane) afforded **3o** (110 mg, 0.68 mmol, 34%) as an orange solid.

R*f* 0.44 (100% *n*-pentane).

^{1}H NMR (501 MHz, $CDCl_3$) δ 7.67–7.61 (m, 2H), 7.46 (d, *J* = 1.7 Hz, 1H), 7.15–7.04 (m, 2H), 6.58 (d, *J* = 3.3 Hz, 1H), 6.47 (dd, *J* = 1.8, 3.4 Hz, 1H).

^{13}C NMR (126 MHz, $CDCl_3$) δ 162.3 (d, *J* = 246.7 Hz), 153.3, 142.2, 127.4 (d, *J* = 3.2 Hz), 125.7 (d, *J* = 7.9 Hz), 115.8 (d, *J* = 22.2 Hz), 111.8, 104.8.

^{19}F NMR (471 MHz, $CDCl_3$) δ –114.4.

HRMS m/z (GC-EI): calculated for $C_{10}H_7O_1F_1$ $[M]^+$ 162.04754; found: 162.04769.

2-(3-(Trifluoromethyl)phenyl)furan, 3p

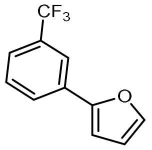

1-Bromo-3-(trifluoromethyl)benzene (450 mg, 2.00 mmol, 1.00 eq.), Cs_2CO_3 (717 mg, 2.20 mmol, 1.10 eq.), furan-2-boronic acid (336 mg, 3.00 mmol, 1.50 eq.) and $Pd(PPh_3)_4$ (116 mg, 0.10 mmol, 5 mol%) in dry toluene (16.0 mL) and dry MeOH (4.0 mL) were subjected to *General Procedure V.* Purification by flash column chromatography (SiO_2, 100% *n*-pentane) afforded **3p** (320 mg, 1.51 mmol, 75%) as a yellow oil.

R*f* 0.42 (100% *n*-pentane).

^{1}H NMR (501 MHz, $CDCl_3$) δ 7.95–7.92 (m, 1H), 7.86–7.79 (m, 1H), 7.56–7.46 (m, 3H), 6.74 (dd, *J* = 0.8, 3.5 Hz, 1H), 6.51 (dd, *J* = 1.8, 3.4 Hz, 1H).

^{13}C NMR (126 MHz, $CDCl_3$) δ 152.6, 142.9, 131.7, 131.3 (q, *J* = 32.3 Hz), 129.3, 126.9 (q, *J* = 1.4 Hz), 124.2 (q, *J* = 272.6 Hz), 123.9 (q, *J* = 3.8 Hz), 120.7 (q, *J* = 3.9 Hz), 112.0, 106.4.

^{19}F NMR (471 MHz, $CDCl_3$) δ –62.9.

HRMS m/z (GC-EI): calculated for $C_{11}H_7O_1F_3$ $[M]^+$ 212.04435; found: 212.04456.

(*Z*)-3-Iodo-2-methylprop-2-en-1-ol, S1

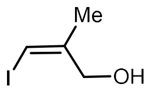

According to a procedure by Kumar et al.[24]

Propargyl alcohol (0.70 mL, 12.0 mmol, 1.00 eq.) was dissolved in dry THF (14.0 mL). CuI (229 mg, 1.20 mmol, 10 mol%) was added and the reaction flask was cooled to −10 °C. Methylmagnesium bromide solution (7.0 mL, 21.0 mmol, 1.75 eq., 3.0 M in Et_2O) was added dropwise, and the solution was stirred for 30 minutes at −10 °C. A solution of I_2 (3350 mg, 13.2 mmol, 1.10 eq.) in a THF (3.0 mL) / Et_2O (3.0 mL) mixture was added slowly via a dropping funnel. The solution was stirred for 10 h keeping the external reaction temperature between −7 and 0 °C. Upon complete conversion, the solution was diluted with Et_2O and washed with NH_4Cl (aq. sat.). The aqueous phase was extracted with Et_2O three times. The combined organic layers were washed with Na_2SO_3 solution (aq. sat.), dried over $MgSO_4$, filtered, and the solvents and volatile impurities were removed under the vacuum. The obtained dark-yellow oil **S1** (1.50 g, 7.58 mmol, 63%) was sufficiently pure to be used in the next step.

R_f 0.20 (*n*-pentane / EtOAc, 95:5).

^{1}H NMR (501 MHz, $CDCl_3$) δ 5.98 (m, 1H), 4.25 (d, *J* = 5.4 Hz, 2H), 1.98 (d, *J* = 1.5 Hz, 3H), 1.57 (t, *J* = 6.0 Hz, 1H, -O*H* peak).

^{13}C NMR (126 MHz, $CDCl_3$) δ 146.2, 75.1, 68.3, 21.8.

HRMS m/z (GC-EI): calculated for $C_4H_7O_1I_1$ $[M]^+$ 197.953612; found: 197.95388.

3-(Allyloxy)oct-1-ene, S2

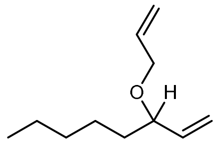

NaH (287 mg, 7.18 mmol, 2.00 eq., 60% in mineral oil) was added to a solution of oct-1-en-3-ol (0.55 mL, 3.59 mmol, 1.00 eq.) in dry THF (10.0 mL) at 0 °C under an inert atmosphere. The mixture was stirred at 0 °C for further 15 minutes. Then, allyl bromide (0.37 mL, 4.31 mmol, 1.20 eq.) was added dropwise to the mixture over 5 minutes. The mixture was stirred at room temperature for 20 h. Finally, the mixture was diluted with Et_2O and washed with HCl (0.1 M aq.). The organic layer was dried over $MgSO_4$, filtered and then concentrated under reduced pressure. Purification by flash column chromatography (SiO_2, EtOAc in *n*-pentane, gradient 0% to 2%) afforded **S2** (512 mg, 3.04 mmol, 84%) as a colorless oil.

R_f 0.38 (100% *n*-pentane).

^{1}H NMR (501 MHz, $CDCl_3$) δ 5.96–5.85 (m, 1H), 5.67 (ddd, *J* = 7.8, 10.6, 16.9 Hz, 1H), 5.25 (dq, *J* = 1.7, 17.2 Hz, 1H), 5.21–5.11 (m, 3H), 4.04 (ddt, *J* = 1.6, 5.2, 12.9 Hz, 1H), 3.82 (ddt, *J* = 1.4, 6.0, 12.8 Hz, 1H), 3.67 (app q, *J* = 6.9 Hz, 1H), 1.66–1.56 (m, 1H), 1.52–1.42 (m, 1H), 1.43–1.22 (m, 6H), 0.88 (t, *J* = 6.8 Hz, 3H).

^{13}C NMR (126 MHz, $CDCl_3$) δ 139.4, 135.4, 116.8, 116.6, 80.9, 69.3, 35.6, 32.0, 25.2, 22.7, 14.2.

HRMS m/z (API-MS): calculated for $C_{11}H_{20}O_1Na_1$ $[M+Na]^+$ 191.14063; found: 191.14049.

2-Pentyl-2,5-dihydrofuran, S3

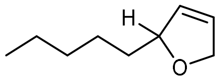

To flame-dried Schlenk-flask equipped with stir bar and molecular sieves, 3-(allyloxy)oct-1-ene **S2** (400 mg, 2.38 mmol, 1.00 eq.) and dry DCM (5.0 mL) were added under an inert atmosphere. Grubbs Generation I catalyst (75.0 mg, 0.12 mmol, 5 mol%) was added as a solid. The mixture was stirred for 40 minutes at room temperature. The crude mixture was filtered over celite and concentrated under reduced pressure. Purification by flash column chromatography (SiO_2, 2% Et_2O in *n*-pentane) afforded **S3** (112 mg, 0.80 mmol, 34%) as a colorless oil. The compound was stored under an inert atmosphere at −20 °C.

R*f* 0.40 (*n*-pentane / Et_2O, 95:5).

^{1}H NMR (501 MHz, $CDCl_3$) δ 5.90–5.84 (m, 1H), 5.81–5.76 (m, 1H), 4.84–4.79 (m, 1H), 4.69–4.57 (m, 2H), 1.60–1.49 (m, 2H), 1.43–1.23 (m, 6H), 1.01–0.71 (m, 3H).

HRMS m/z (GC-EI): calculated for $C_9H_{16}O_1$ $[M]^+$ 140.11957; found: 140.11974.

Analytical data matches that previously reported.[25]

5-Hexyl-2,3-dihydrofuran, S4

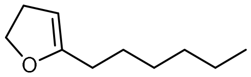

According to a procedure by Hendenström et al.[26]

Tert-butyllithium (2.94 mL, 5.00 mmol, 1.00 eq., 1.7 M in *n*-pentane) was added dropwise to a stirred solution of 2,3-dihydrofuran (0.38 mL, 5.00 mmol, 1.00 eq.) in dry THF (2 mL) at −78 °C. The mixture was slowly allowed to warm up to −5 °C during 1 h. Subsequently, the mixture was cooled to −20 °C, and l-iodohexane (0.52 mL, 3.50 mmol, 0.70 eq.) was added over 10 minutes. The mixture was allowed to warm up to room temperature and then refluxed for 3 h. Finally, the mixture was cooled to 0 °C and poured slowly into NH_4Cl solution (aq. sat.). The layers were separated, and the aqueous layer was extracted with another portion of Et_2O. The combined organics were dried over $MgSO_4$, filtered and the solvents removed under reduced pressure. The crude material **S4** was used without further purification, still containing residual 1-iodohexane (~26%).

Note: The compound is not stable over a prolonged time in $CDCl_3$ or neat and was, therefore, used immediately. The compound decomposes on silica.

R*f* 0.27 (100% *n*-pentane).

^{1}H NMR (501 MHz, CD_2Cl_2) δ 4.56 (tt, J = 2.3, 1.1 Hz, 1H), 4.25 (t, J = 9.3 Hz, 2H), 2.57 (tq, J = 9.3, 2.0 Hz, 2H), 2.10–2.02 (m, 2H), 1.51–1.43 (m, 2H), 1.36–1.23 (m, 8H), 0.92–0.85 (m, 3H).

^{13}C NMR (126 MHz, CD_2Cl_2) δ 159.6, 93.7, 70.1, 32.1, 30.4, 29.4, 28.3, 27.1, 23.0, 14.3.

HRMS m/z (GC-CI): calculated for $C_{10}H_{22}N_1O_1$ $[M+NH_4]^+$ 154.13522; found: 154.13543.

10. Substrates

3-(4-Methoxyphenyl)-2,5-dihydrofuran, 2a

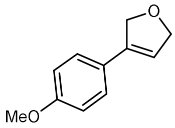

3-(4-Methoxyphenyl)furan **1a** / **3a** (34.8 mg, 0.20 mmol, 1.00 eq.), Et_3SiH (48 µL, 0.30 mmol, 1.50 eq.), distilled H_2O (3.6 µL, 0.20 mmol, 1.00 eq.) and TFA (0.77 µL, 0.0100 mmol, 5 mol%) in HFIP (1.0 mL) were subjected to *General Procedure A* for 4 h. Purification by flash column chromatography (SiO_2, 5% Et_2O in *n*-pentane) afforded **2a** (24.0 mg, 0.136 mmol, 68%) as a white solid.

Note: If reaction time is increased, overreduction is observed.

R_f 0.23 (*n*-pentane / Et_2O, 90:10).

^{1}H NMR (501 MHz, $CDCl_3$) δ 7.29–7.26 (m, 2H), 6.90–6.86 (m, 2H), 6.09 (p, *J* = 2.0 Hz, 1H), 4.98 (td, *J* = 4.8, 2.2 Hz, 2H), 4.83 (td, *J* = 4.8, 1.9 Hz, 2H), 3.82 (s, 3H).

^{13}C NMR (126 MHz, $CDCl_3$) δ 159.5, 138.0, 127.2, 125.5, 118.4, 114.2, 77.0, 75.6, 55.5.

HRMS m/z (GC-EI): calculated for $C_{11}H_{12}O_2$ $[M]^+$ 176.08318; found 176.08336.

3-(4-Methoxyphenyl)tetrahydrofuran, 4a

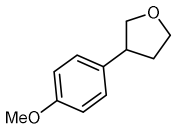

3-(4-Methoxyphenyl)furan **1a** / **3a** (17.4 mg, 0.10 mmol, 1.00 eq.), Et_3SiH (40 µL, 0.25 mmol, 2.50 eq.) and TfOH (0.18 µL, 0.00200 mmol, 2 mol%) in HFIP (0.50 mL) were subjected to *General Procedure B* for 12 h. Purification by flash column chromatography (SiO_2, 5% Et_2O in *n*-pentane) afforded **4a** (12.8 mg, 0.072 mmol, 72%) as a colorless oil.

R_f 0.22 (*n*-pentane / Et_2O, 90:10).

^{1}H NMR (501 MHz, $CDCl_3$) δ 7.21–7.14 (m, 2H), 6.90–6.83 (m, 2H), 4.15–4.09 (m, 1H), 4.05 (td, *J* = 8.4, 4.5 Hz, 1H), 3.91 (td, *J* = 8.2, 7.2 Hz, 1H), 3.80 (s, 3H), 3.67 (t, *J* = 8.1 Hz, 1H), 3.36 (p, *J* = 7.9 Hz, 1H), 2.33 (dtd, *J* = 12.3, 7.6, 4.5 Hz, 1H), 1.97 (dq, *J* = 12.3, 8.2 Hz, 1H).

^{13}C NMR (126 MHz, $CDCl_3$) δ 158.4, 134.7, 128.3, 114.1, 74.9, 68.6, 55.4, 44.4, 34.9.

HRMS m/z (GC-EI): calculated for $C_{11}H_{14}O_2$ $[M]^+$ 178.09883; found 178.09902.

3-(4-(*tert*-Butyl)phenyl)-2,5-dihydrofuran, 2b

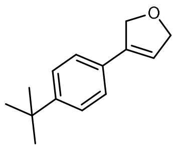

3-(4-(*tert*-Butyl)phenyl)furan **1b** / **3b** (40.1 mg, 0.20 mmol, 1.00 eq.), Et_3SiH (48 µL, 0.30 mmol, 1.50 eq.), distilled H_2O (3.6 µL, 0.20 mmol, 1.00 eq.) and TFA (0.77 µL, 0.010 mmol, 5 mol%) in HFIP (1.0 mL) were subjected to *General Procedure A* for 12 h. Purification by flash column chromatography (SiO_2, 5% Et_2O in *n*-pentane) afforded **2b** (38.4 mg, 0.190 mmol, 95%) as a white solid.

R*f* 0.25 (*n*-pentane / EtOAc, 95:5).

^{1}H NMR (501 MHz, $CDCl_3$) δ 7.40–7.36 (m, 2H), 7.31–7.27 (m, 2H), 6.18 (p, *J* = 2.0 Hz, 1H), 5.00 (td, *J* = 4.9, 2.2 Hz, 2H), 4.85 (td, *J* = 4.8, 1.9 Hz, 2H), 1.33 (s, 9H).

^{13}C NMR (126 MHz, $CDCl_3$) δ 151.3, 138.4, 129.9, 126.8, 125.7, 119.8, 76.9, 75.6, 34.8, 31.4.

HRMS m/z (GC-EI): calculated for $C_{14}H_{18}O_1$ $[M]^+$ 202.13522; found 202.13533.

3-(4-(*tert*-Butyl)phenyl)tetrahydrofuran, 4b

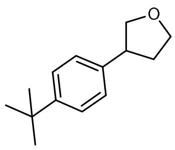

3-(4-(*tert*-Butyl)phenyl)furan **1b** / **3b** (20.0 mg, 0.10 mmol, 1.00 eq.), Et_3SiH (40 µL, 0.25 mmol, 2.50 eq.) and TfOH (0.18 µL, 0.0020 mmol, 2 mol%) in HFIP (0.50 mL) were subjected to *General Procedure B* for 12 h. Purification by flash column chromatography (SiO_2, 5% Et_2O in *n*-pentane) afforded **4b** (17.0 mg, 0.083 mmol, 83%) as a colorless oil.

R*f* 0.25 (*n*-pentane / EtOAc, 95:5).

^{1}H NMR (501 MHz, $CDCl_3$) δ 7.38–7.32 (m, 2H), 7.22–7.17 (m, 2H), 4.14 (t, *J* = 8.0 Hz, 1H), 4.06 (td, *J* = 8.3, 4.4 Hz, 1H), 3.92 (td, *J* = 8.2, 7.2 Hz, 1H), 3.71 (t, *J* = 8.1 Hz, 1H), 3.38 (p, *J* = 8.0 Hz, 1H), 2.35 (dtd, *J* = 12.2, 7.6, 4.4 Hz, 1H), 2.02 (dq, *J* = 12.3, 8.2 Hz, 1H), 1.32 (s, 9H).

^{13}C NMR (126 MHz, $CDCl_3$) δ 149.5, 139.6, 127.1, 125.6, 74.8, 68.7, 44.7, 34.8, 34.6, 31.5.

HRMS m/z (GC-EI): calculated for $C_{14}H_{20}O_1$ $[M]^+$ 204.15087; found 204.15099.

3-(*para*-Tolyl)-2,5-dihydrofuran, 2c

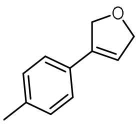

3-(*para*-Tolyl)furan **1c** / **3c** (15.8 mg, 0.10 mmol, 1.00 eq.), Et_3SiH (24.0 µL, 0.15 mmol, 1.50 eq.), distilled H_2O (1.8 µL, 0.10 mmol, 1.00 eq.) and TFA (0.38 µL, 5.0 µmol, 5 mol%) in HFIP (0.50 mL) were subjected to *General Procedure A* for 12 h. Purification by flash column chromatography (SiO_2, 5% MTBE in *n*-pentane) afforded **2c** (11.5 mg, 0.084 mmol, 84%) as a white solid.

R_f 0.25 (*n*-pentane / EtOAc, 95:5).

^{1}H NMR (501 MHz, $CDCl_3$) δ 7.25–7.21 (m, 2H), 7.18–7.14 (m, 2H), 6.17 (p, *J* = 2.1 Hz, 1H), 4.99 (td, *J* = 4.9, 2.2 Hz, 2H), 4.84 (td, *J* = 4.8, 1.9 Hz, 2H), 2.35 (s, 3H).

^{13}C NMR (126 MHz, $CDCl_3$) 138.5, 138.0, 129.9, 129.4, 125.8, 119.6, 76.9, 75.6, 21.4.

HRMS m/z (GC-EI): calculated for $C_{11}H_{12}O_1$ $[M]^+$ 160.0883; found 160.0885.

3-(*para*-Tolyl)tetrahydrofuran, 4c

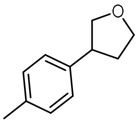

3-(*para*-Tolyl)furan **1c** / **3c** (31.6 mg, 0.20 mmol, 1.00 eq.), Et_3SiH (80 µL, 0.50 mmol, 2.50 eq.) and TfOH (0.35 µL, 4.0 µmol, 2 mol%) in HFIP (1.0 mL) were subjected to *General Procedure B* for 12 h. Purification by flash column chromatography (SiO_2, 5% Et_2O in *n*-pentane) afforded **4c** (28.0 mg, 0.16 mmol, 80%) as a colorless oil.

R_f 0.30 (*n*-pentane / EtOAc, 95:5).

^{1}H NMR (501 MHz, $CDCl_3$) δ 7.18–7.11 (m, 4H), 4.13 (t, *J* = 8.0 Hz, 1H), 4.06 (td, *J* = 4.4, 8.3 Hz, 1H), 3.92 (q, *J* = 7.9 Hz, 1H), 3.70 (t, *J* = 8.0 Hz, 1H), 3.37 (p, *J* = 7.9 Hz, 1H), 2.40–2.34 (m, 1H), 2.33 (s, 3H), 1.99 (dq, *J* = 8.2, 12.3 Hz, 1H).

^{13}C NMR (126 MHz, $CDCl_3$) δ 139.6, 136.2, 129.4, 127.3, 74.9, 68.7, 44.8, 34.8, 21.1.

HRMS m/z (GC-EI): calculated for $C_{11}H_{14}O_1$ $[M]^+$ 162.1039; found 162.1040.

3-Phenyl-2,5-dihydrofuran, 2d

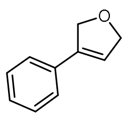

3-Phenylfuran **1d** / **3d** (28.8 mg, 0.20 mmol, 1.00 eq.), Et_3SiH (48 µL, 0.30 mmol, 1.50 eq.), distilled H_2O (3.6 µL, 0.20 mmol, 1.00 eq.) and TFA (0.77 µL, 10 µmol, 5 mol%) in HFIP (1.0 mL) were subjected to *General*

Procedure A for 12 h. Purification by flash column chromatography (SiO_2, 5% Et_2O in *n*-pentane) afforded **2d** (26.6 mg, 0.182 mmol, 91%) as a white solid.

R*f* 0.23 (*n*-pentane / EtOAc, 95:5).

^{1}H NMR (501 MHz, $CDCl_3$) δ 7.38–7.33 (m, 4H), 7.32–7.27 (m, 1H), 6.23 (p, *J* = 2.0 Hz, 1H), 5.01 (td, *J* = 4.9, 2.2 Hz, 2H), 4.86 (td, *J* = 4.9, 2.0 Hz, 2H).

^{13}C NMR (126 MHz, $CDCl_3$) δ 138.6, 132.6, 128.8, 128.2, 125.9, 120.6, 76.9 (overlap with $CDCl_3$ triplet), 75.5.

HRMS m/z (GC-EI): calculated for $C_{10}H_{10}O_1$ $[M]^+$ 146.07262; found 146.07263.

3-Phenyltetrahydrofuran, 4d

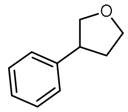

3-Phenylfuran **1d** / **3d** (14.4 mg, 0.10 mmol, 1.00 eq.), Et_3SiH (40 µL, 0.25 mmol, 2.50 eq.) and TfOH (0.18 µL, 2.0 µmol, 2 mol%) in HFIP (0.50 mL) were subjected to *General Procedure B* for 12 h. Purification by flash column chromatography (SiO_2, Et_2O in *n*-pentane, gradient 5 to 10%) afforded **4d** (11.1 mg, 0.075 mmol, 75%) as a colorless oil.

R*f* 0.23 (*n*-pentane / Et_2O, 95:5).

^{1}H NMR (501 MHz, $CDCl_3$) δ 7.34–7.29 (m, 2H), 7.28–7.18 (m, 3H, *overlapping with $CDCl_3$ signal*), 4.18–4.11 (m, 1H), 4.07 (td, *J* = 8.3, 4.5 Hz, 1H), 3.92 (td, *J* = 8.2, 7.2 Hz, 1H), 3.73 (dd, *J* = 8.5, 7.6 Hz, 1H), 3.40 (p, *J* = 7.9 Hz, 1H), 2.42–2.32 (m, 1H), 2.01 (dq, *J* = 12.3, 8.1 Hz, 1H).

^{13}C NMR (126 MHz, $CDCl_3$) δ 142.8, 128.7, 127.4, 126.7, 74.8, 68.7, 45.2, 34.8.

HRMS m/z (GC-EI): calculated for $C_{10}H_{12}O_1$ $[M]^+$ 148.08827; found 148.08842.

3-(4-Chlorophenyl)-2,5-dihydrofuran, 2e

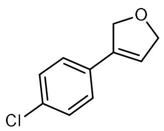

3-(4-Chlorophenyl)furan **1e** / **3e** (23.0 mg, 0.129 mmol, 1.00 eq.), Et_3SiH (31 µL, 0.19 mmol, 1.50 eq.), distilled H_2O (2.3 µL, 0.129 mmol, 1.00 eq.) and TFA (0.49 µL, 6.4 µmol, 5 mol%) in HFIP (0.65 mL) were subjected to *General Procedure A* for 12 h. Purification by flash column chromatography (SiO_2, 5% Et_2O in *n*-pentane) afforded **2e** (20.7 mg, 0.115 mmol, 89%) as a white solid.

R*f* 0.25 (*n*-pentane / EtOAc, 95:5).

^{1}H NMR (501 MHz, $CDCl_3$) δ 7.33–7.30 (m, 2H), 7.27–7.24 (m, 2H), 6.22 (p, *J* = 2.1 Hz, 1H), 4.97 (td, *J* = 4.9, 2.2 Hz, 2H), 4.84 (td, *J* = 4.9, 2.0 Hz, 2H).

^{13}**C NMR** (126 MHz, $CDCl_3$) δ 137.6, 133.9, 131.1, 129.0, 127.1, 121.4, 76.9 *(overlap with $CDCl_3$ peak),* 75.4.

HRMS m/z (GC-EI): calculated for $C_{10}H_9O_1Cl_1$ $[M]^+$ 180.03364; found 180.03363.

3-(4-Chlorophenyl)tetrahydrofuran, 4e

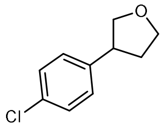

3-(4-Chlorophenyl)furan **1e** / **3e** (17.9 mg, 0.10 mmol, 1.00 eq.), Et_3SiH (40 µL, 0.25 mmol, 2.50 eq.) and TfOH (0.18 µL, 2.0 µmol, 2 mol%) in HFIP (0.50 mL) were subjected to *General Procedure B* for 12 h. Purification by flash column chromatography (SiO_2, Et_2O in *n*-pentane, gradient 5 to 10%) afforded **4e** (15.0 mg, 0.082 mmol, 82%) as a colorless oil.

$\mathbf{R}_f$ 0.19 (*n*-pentane / EtOAc, 95:5).

^{1}**H NMR** (501 MHz, $CDCl_3$) δ 7.31–7.25 (m, 2H, *overlap with $CDCl_3$ peak*), 7.21–7.15 (m, 2H), 4.11 (dd, J = 8.5, 7.5 Hz, 1H), 4.06 (td, J = 8.4, 4.6 Hz, 1H), 3.91 (q, J = 7.8 Hz, 1H), 3.70 (dd, J = 8.5, 7.2 Hz, 1H), 3.38 (p, J = 7.7 Hz, 1H), 2.36 (dtd, J = 12.4, 7.8, 4.6 Hz, 1H), 1.96 (dq, J = 12.4, 8.0 Hz, 1H).

^{13}**C NMR** (126 MHz, $CDCl_3$) δ 141.5, 132.3, 128.8, 128.7, 74.7, 68.6, 44.5, 34.8.

HRMS m/z (GC-EI): calculated for $C_{10}H_{11}O_1Cl_1$ $[M]^+$ 182.04929; found 182.04954.

3-(4-(Trifluoromethyl)phenyl)-2,5-dihydrofuran, 2f

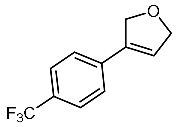

3-(4-(Trifluoromethyl)phenyl)furan **1f** / **3f** (21.2 mg, 0.10 mmol, 1.00 eq.), Et_3SiH (24.0 µL, 0.15 mmol, 1.50 eq.), distilled H_2O (1.8 µL, 0.10 mmol, 1.00 eq.) and TFA (0.38 µL, 5.0 µmol, 5 mol%) in HFIP (0.50 mL) were subjected to *General Procedure A* for 12 h. Purification by flash column chromatography (SiO_2, MTBE in *n*-pentane, gradient 5% to 10%) afforded **2f** (15.4 mg, 0.072 mmol, 72%) as a white solid.

$\mathbf{R}_f$ 0.18 (*n*-pentane / EtOAc, 95:5).

^{1}**H NMR** (500 MHz, $CDCl_3$) δ 7.66–7.56 (m, 2H), 7.49–7.38 (m, 2H), 6.36 (p, J = 2.0 Hz, 1H), 5.02 (td, J = 4.9, 2.2 Hz, 2H), 4.88 (td, J = 4.9, 1.9 Hz, 2H).

^{13}**C NMR** (151 MHz, $CDCl_3$) δ 137.6, 136.0 (q, J = 1.3 Hz), 130.0 (q, J = 32.7 Hz), 126.1, 125.8 (q, J = 3.8 Hz), 124.2 (q, J = 272.0 Hz), 123.5, 77.0 *(overlap with $CDCl_3$ peak),* 75.3.

^{19}**F NMR** (565 MHz, $CDCl_3$) δ −62.7.

HRMS m/z (GC-EI): calculated for $C_{11}H_9F_3O_1$ $[M]^+$ 214.0600; found 214.0601.

3-(4-Nitrophenyl)-2,5-dihydrofuran, 2g

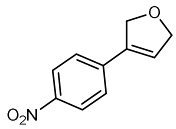

3-(4-Nitrophenyl)furan **1g** / **3g** (18.9 mg, 0.10 mmol, 1.00 eq.), Et_3SiH (40 µL, 0.25 mmol, 2.50 eq.) and TfOH (0.18 µL, 2.0 µmol, 2 mol%) in HFIP (0.50 mL) were subjected to *General Procedure B* for 12 h. After 12 h reaction time, another portion of Et_3SiH (40 µL, 0.25 mmol, 2.50 eq.) was added, stirred for further 3 h, to achieve full conversion. Purification by flash column chromatography (SiO_2, Et_2O in *n*-pentane, gradient 30% to 40%) afforded **2g** (15.0 mg, 0.078 mmol, 78%) as an orange solid.

R_f 0.40 (*n*-pentane / EtOAc, 80:20).

^{1}H NMR (501 MHz, $CDCl_3$) δ 8.24–8.18 (m, 2H), 7.53–7.43 (m, 2H), 6.48 (p, *J* = 2.1 Hz, 1H), 5.03 (td, *J* = 4.9, 2.1 Hz, 2H), 4.90 (td, *J* = 5.0, 2.0 Hz, 2H).

^{13}C NMR (126 MHz, $CDCl_3$) δ 147.3, 138.8, 137.2, 126.5, 125.8, 124.2, 77.0, 75.2.

HRMS m/z (GC-EI): calculated for $C_{10}H_9N_1O_3$ $[M]^+$ 191.05769; found 191.05778.

3-(Cyclohexylmethyl)-2,5-dihydrofuran, 2h

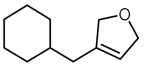

3-(Cyclohexylmethyl)furan **1h** (16.4 mg, 0.10 mmol, 1.00 eq.), Et_3SiH (24.0 µL, 0.15 mmol, 1.50 eq.) and TfOH (0.18 µL, 2.0 µmol, 2 mol%) in HFIP (0.50 mL) were subjected to *General Procedure B* for 15 minutes. Purification by flash column chromatography (SiO_2, 3% Et_2O in *n*-pentane) afforded **2h** (14.3 mg, 0.086 mmol, 86%) as a colorless oil.

R_f 0.20 (*n*-pentane / Et_2O, 95:5).

^{1}H NMR (501 MHz, $CDCl_3$) δ 5.45 (m, 1H), 4.63 (m, 2H), 4.50 (m, 2H), 2.00 (d, 2H), 1.76–1.62 (m, 5H), 1.38 (m, 1H), 1.29–1.08 (m, 3H), 0.94–0.82 (m, 2H).

^{13}C NMR (126 MHz, $CDCl_3$) δ 139.2, 120.1, 77.3, 76.0, 36.4, 35.3, 33.5, 26.6, 26.4.

HRMS m/z (GC-EI): calculated for $C_{11}H_{18}O_1$ $[M]^+$ 166.13522; found: 166.13525.

3-Heptyl-2,5-dihydrofuran, 2i

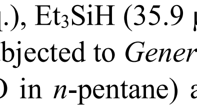

3-Heptylfuran **1i** (24.9 mg, 0.15 mmol, 1.00 eq.), Et_3SiH (35.9 µL, 0.225 mmol, 1.50 eq.) and TfOH (0.27 µL, 3.0 µmol, 2 mol%) in HFIP (0.75 mL) were subjected to *General Procedure B* for 15 minutes. Purification by flash column chromatography (SiO_2, 5% Et_2O in *n*-pentane) afforded **2i** (22.0 mg, 0.131 mmol, 87%) as a colorless oil.

R_f 0.38 (*n*-pentane / Et_2O, 95:5).

^{1}H NMR (501 MHz, $CDCl_3$) δ 5.48–5.43 (m, 1H), 4.65–4.60 (m, 2H), 4.54–4.50 (m, 2H), 2.12–2.04 (m, 2H), 1.47 (m, 2H), 1.42–1.18 (m, 10H), 0.92–0.86 (m, 3H).

^{13}C NMR (126 MHz, $CDCl_3$) δ 140.8, 118.8, 77.2, 76.1, 31.9, 29.5, 29.3, 27.8, 27.3, 22.8, 14.2.

HRMS m/z (GC-EI): calculated for $C_{11}H_{20}O_1$ $[M]^+$ 168.15087; found 168.15082.

3-Cyclobutyl-2,5-dihydrofuran, 2j

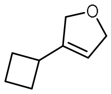

3-Cyclobutylfuran **1j** (36.6 mg, 0.30 mmol, 1.00 eq.), Et_3SiH (71.8 µL, 0.45 mmol, 1.50 eq.) and TfOH (0.53 µL, 6.0 µmol, 2 mol%) in HFIP (1.5 mL) were subjected to *General Procedure B* for 15 minutes. Purification by flash column chromatography (SiO_2, 5% Et_2O in *n*-pentane) afforded **2j** (33.9 mg, 0.273 mmol, 91%) as a colorless oil.

R_f 0.24 (*n*-pentane / Et_2O, 95:5).

^{1}H NMR (501 MHz, $CDCl_3$) δ 5.44 (app h, 1H), 4.64 (m, 2H), 4.55–4.49 (m, 2H), 3.12–2.87 (m, 1H), 2.26–2.08 (m, 2H), 2.04–1.91 (m, 3H), 1.91–1.77 (m, 1H).

^{13}C NMR (126 MHz, $CDCl_3$) δ 144.6, 117.5, 76.2, 75.7, 33.3, 27.9, 18.9.

HRMS m/z (GC-EI): calculated for $C_8H_{12}O_1$ $[M]^+$ 124.08827; found: 124.08844.

3-(3-Methoxypropyl)-2,5-dihydrofuran, 2k

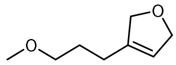

3-(3-Methoxypropyl)furan **1k** (21.0 mg, 0.15 mmol, 1.00 eq.), Et_3SiH (35.9 µL, 0.225 mmol, 1.50 eq.) and TfOH (0.27 µL, 3.0 µmol, 2 mol%) in HFIP (0.75 mL) were subjected to *General Procedure B* for 15 minutes. Purification by flash column chromatography (SiO_2, Et_2O in *n*-pentane, gradient 20% to 30%) afforded **2k** (20.0 mg, 0.141 mmol, 93%) as a yellow oil.

R_f 0.26 (*n*-pentane / Et_2O, 80:20).

^{1}H NMR (501 MHz, $CDCl_3$) δ 5.48 (m, 1H), 4.63 (m, 2H), 4.52 (m, 2H), 3.39 (t, J = 6.4 Hz, 2H), 3.33 (s, 3H), 2.19–2.12 (m, 2H), 1.80–1.71 (m, 2H).

^{13}C NMR (126 MHz, $CDCl_3$) δ 140.1, 119.2, 77.2, 76.2, 72.3, 58.8, 27.8, 23.8.

HRMS m/z (API-MS): calculated for $C_8H_{14}Na_1O_2$ $[M+Na]^+$ 165.08860; found 165.08872.

3-(But-3-en-1-yl)-2,5-dihydrofuran, 2n

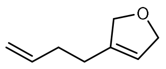

3-(But-3-en-1-yl)furan **1n** (30.0 mg, 0.25 mmol, 1.00 eq.), Et_3SiH (58.9 µL, 0.37 mmol, 1.50 eq.) and TfOH (0.44 µL, 4.9 µmol, 2 mol%) in HFIP (1.25 mL) were subjected to *General Procedure B* for 20 minutes. Purification by flash column chromatography (SiO_2, 3% Et_2O in *n*-pentane) afforded **2n** (25.2 mg, 0.201 mmol, 82%) as a colorless oil.

$\mathbf{R}_f$ 0.23 (*n*-pentane / Et_2O, 95:5).

^{1}H NMR (501 MHz, $CDCl_3$) δ 5.83 (ddt, *J* = 6.3, 10.2, 16.8 Hz, 1H), 5.49 (app hept, 1H), 5.05 (dq, *J* = 1.7, 17.1 Hz, 1H), 4.99 (dq, *J* = 1.4, 10.3 Hz, 1H), 4.63 (m, 2H), 4.52 (m, 2H), 2.24 (m, 2H), 2.19 (m, 2H).

^{13}C NMR (126 MHz, $CDCl_3$) δ 140.0, 138.0, 119.4, 115.2, 77.2, 76.1, 31.9, 26.6.

HRMS m/z (GC-EI): calculated for $C_8H_{12}O_1$ $[M]^+$ 124.08827; found: 124.08838.

3-(4-methylpent-3-en-1-yl)-2,5-dihydrofuran, 2,5-Dihydro-*Perillene*, 2o

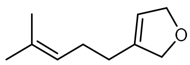

Perillene **1o** (22.5 mg, 0.15 mmol, 1.00 eq.), Et_3SiH (35.9 µL, 0.225 mmol, 1.50 eq.) and TfOH (0.27 µL, 3.0 µmol, 2 mol%) in HFIP (0.75 mL) were subjected to *General Procedure B* for 30 minutes. Purification by flash column chromatography (SiO_2, Et_2O in *n*-pentane, gradient 2% to 5%) afforded **2o** (13.0 mg, 0.085 mmol, 57%) as a colorless oil.

$\mathbf{R}_f$ 0.27 (*n*-pentane / Et_2O, 95:5).

^{1}H NMR (501 MHz, $CDCl_3$) δ 5.48 (app hept, 1H), 5.11 (m, 1H), 4.63 (m, 2H), 4.51 (m, 2H), 2.21–2.13 (m, 2H), 2.13–2.08 (m, 2H), 1.69 (s, 3H), 1.61 (s, 3H).

^{13}C NMR (126 MHz, $CDCl_3$) δ 140.5, 132.3, 123.8, 119.1, 77.3, 76.1, 27.4, 26.4, 25.8, 17.9.

HRMS m/z (GC-EI): calculated for $C_{10}H_{16}O_1$ $[M]^+$ 152.11957; found: 152.11956.

(6*R*,7a*R*)-3,6-Dimethyl-2,4,5,6,7,7a-hexahydrobenzofuran, 2,5-Dihydro-*menthofuran*, 2p

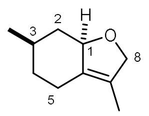

Deviation from the General Procedure B: 5 mol% instead of 2 mol% TfOH loading used as well as slightly higher Et_3SiH equivalents.

Commercial (+)-Menthofuran (30.0 mg, 0.20 mmol, 1.00 eq.), Et_3SiH (63.9 µL, 0.40 mmol, 2.00 eq.) and TfOH (0.88 µL, 10 µmol, 5 mol%) in HFIP (1.0 mL) were subjected to *General Procedure B* for 12 h. Purification by two subsequent flash column chromatography (SiO_2, Et_2O in *n*-pentane, gradient 0% to 5%; *then*: SiO_2, 2% Et_2O in *n*-pentane) afforded **2p** (22.8 mg, 0.15 mmol, 75%) as a colorless oil.

R_f 0.50 (*n*-pentane / EtOAc, 90:10).

^{1}H NMR (600 MHz, $CDCl_3$) δ 4.56–4.50 (m, 2H), 4.50–4.45 (m, 1H), 2.49 (ddd, *J* = 14.1, 4.5, 2.1 Hz, 2H), 2.06 (dddd, *J* = 11.7, 5.2, 3.0, 1.8 Hz, 1H), 1.87–1.78 (m, 1H), 1.71 (ddddd, *J* = 12.8, 5.2, 3.4, 2.1, 1.8 Hz, 1H), 1.60 (tt, *J* = 1.6, 1.0 Hz, 3H), 1.57–1.47 (m, 1H), 0.95 (td, *J* = 12.0, 10.5 Hz, 1H), 0.94 (d, *J* = 6.6 Hz, 4H), 0.84 (tdd, *J* = 13.2, 11.9, 4.5 Hz, 1H).

^{13}C NMR (151 MHz, $CDCl_3$) δ 133.0, 123.6, 86.3, 78.8, 43.7, 34.9, 30.2, 23.8, 22.1, 9.6.

HRMS m/z (GC-EI): calculated for $C_{10}H_{16}O_1$ $[M]^+$ 152.11957; found: 152.11981.

Assignment of relative stereochemistry

The compound shows many ^4J and ^5J couplings, so a detailed *J* coupling determination was difficult in many cases (e.g., for $H5_{ax}$, H1, H8', H8"). Based on NOEs between $H5_{ax}$, H3 and H1 (see model below), the relative stereochemistry is supported. Additionally, $H2_{ax}$ shows 3 large couplings (^{2}J to $H2_{eq}$; and two *trans*-couplings to H3 and H1). For the 1,3-*trans*-substitution of H1 and H3, smaller and different couplings would have been expected from $H2_{ax}$. This is clearly not the case in this compound.

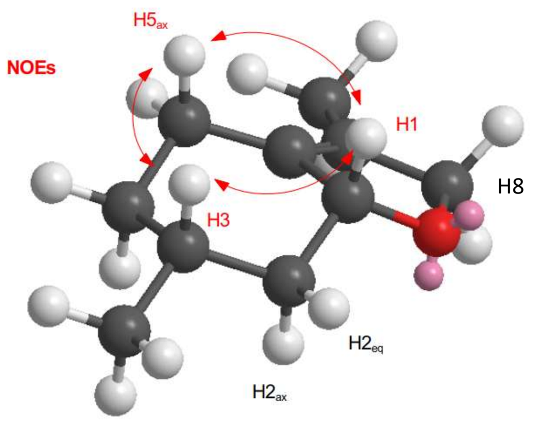

Investigations into diastereoselectivity

Inspection of the ^{1}H NMR of the crude reaction mixture revealed no other diastereomeric product. Furthermore, the crude reaction mixture (purified from silyl-byproducts) was subjected to GC-MS analysis. Also here, only one peak with m/z = 154 was observed, hinting towards high diastereoselectivity dr > 1:20.

Methyl 2-(4-methyl-2,5-dihydrofuran-2-yl)acetate, 2q

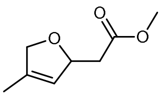

Methyl 2-(4-methylfuran-2-yl)acetate **1q** (30.8 mg, 0.20 mmol, 1.00 eq.), Et_3SiH (47.9 µL, 0.30 mmol, 1.50 eq.) and TfOH (0.35 µL, 4.0 µmol, 2 mol%) in HFIP (1.0 mL) were subjected to *General Procedure B* for 15 minutes. Purification by flash column chromatography (SiO_2, 20% Et_2O in *n*-pentane) afforded **2q** (25.0 mg, 0.16 mmol, 80%) as a colorless oil.

R*f* 0.17 (*n*-pentane / Et_2O, 90:10).

^{1}H NMR (501 MHz, $CDCl_3$) δ 5.48–5.41 (m, 1H), 5.17 (ddtq, *J* = 9.0, 5.4, 3.5, 1.8 Hz, 1H), 4.53 (m, 1H), 4.49–4.44 (m, 1H), 3.69 (s, 3H), 2.61–2.42 (m, 2H), 1.74 (t, *J* = 1.6 Hz, 3H).

^{13}C NMR (126 MHz, $CDCl_3$) δ 171.7, 137.5, 122.8, 83.1, 78.0, 51.8, 41.5, 12.4.

HRMS m/z (GC-EI): calculated for $C_8H_{12}O_3$ $[M]^+$ 156.07810; found 156.07821.

S-((2,5-dihydrofuran-2-yl)methyl) propanethioate, 2r

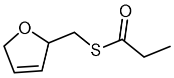

S-(Furan-2-ylmethyl)-propanthioat **1r** (68.1 mg, 0.40 mmol, 1.00 eq.), Et_3SiH (96 µL, 0.60 mmol, 1.50 eq.) and TfOH (0.71 µL, 8.0 µmol, 2 mol%) in HFIP (2.0 mL) were subjected to *General Procedure B* for 2 h. Purification by flash column chromatography (SiO_2, Et_2O in *n*-pentane, gradient 5% to 10%) afforded **2r** (27.6 mg, 0.16 mmol, 40%) as a colorless oil.

Note: The fully reduced tetrahydrofuran product appears as a sideproduct. It is accumulating at the same time as the reported dihydrofuran ***2r****, and not subsequently as observed for the 3-aryl furan derivatives. Therefore, we suspect that the reaction involves a more complex mechanism compared to the one described in the main text.*

R*f* 0.24 (*n*-pentane / Et_2O, 90:10).

^{1}H NMR (501 MHz, $CDCl_3$) δ 5.96 (m, 1H), 5.73 (m, 1H), 4.99 (m, 1H), 4.68 (m, 1H), 4.60 (m, 1H), 3.11 (qd, *J* = 13.7, 5.1 Hz, 2H), 2.58 (q, *J* = 7.5 Hz, 2H), 1.17 (t, *J* = 7.5 Hz, 3H).

^{13}C NMR (126 MHz, $CDCl_3$) δ 200.1, 128.4, 127.9, 84.8, 75.8, 37.5, 34.2, 9.9.

HRMS m/z (API-MS): calculated for $C_8H_{12}O_2S_1Na_1$ $[M+Na]^+$ 195.04502; found 195.04518.

2-Methyl-3-(propyldisulfaneyl)-2,5-dihydrofuran, 2s

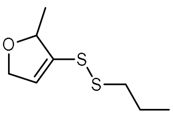

Commercial 2-methyl-3-(propyldisulfanyl)furan **1s** (37.7 mg, 0.20 mmol, 1.00 eq.), Et_3SiH (48 µL, 0.30 mmol, 1.50 eq.) and TfOH (0.36 µL, 4.0 µmol, 2 mol%) in HFIP (1.0 mL) were subjected to *General Procedure B* for 1 h. Purification by flash column chromatography (SiO_2, 2% Et_2O in *n*-pentane) afforded **2s** (8.0 mg, 0.042 mmol, 21%) as a colorless oil.

Note: Starting material and product are highly malodorous.

R*f* 0.29 (*n*-pentane / Et_2O, 95:5).

^{1}H NMR (501 MHz, CD_2Cl_2) δ 5.95 (app q, 1H), 4.90 (m, 1H), 4.65 (m, 1H), 4.60 (m, 1H), 2.77–2.69 (app t, 2H), 1.71 (d, *J* = 7.3 Hz, 2H), 1.31 (d, *J* = 6.3 Hz, 3H), 0.99 (t, *J* = 7.3 Hz, 3H).

^{13}C NMR (151 MHz, CD_2Cl_2) δ 139.0, 124.1, 82.6, 74.7, 40.8, 22.7, 20.8, 13.2.

HRMS m/z (GC-EI): calculated for $C_8H_{14}O_1S_2$ $[M]^+$ 190.04806; found 190.04793.

Methyl 3-methyl-2,5-dihydrofuran-2-carboxylate, 2t

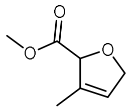

Commercial methyl-3-methylfuroat **1t** (280 mg, 2.00 mmol, 1.00 eq.), Et_3SiH (479 µL, 3.00 mmol, 1.50 eq.) and TfOH (3.53 µL, 40 µmol, 2 mol%) in HFIP (10 mL) were subjected to *General Procedure B* for 12 h. After 12 h reaction time, another portion of Et_3SiH (479 µL, 3.00 mmol, 1.50 eq.) was added. This process was repeated twice to achieve 76% conversion. Purification by flash column chromatography (SiO_2, Et_2O in *n*-pentane, gradient 10% to 20%) afforded **2t** (196 mg, 1.40 mmol, 70%) as a colorless oil.

Note: Higher concentration was found to be detrimental to the conversion.

R*f* 0.23 (*n*-pentane / EtOAc, 95:5).

^{1}H NMR (501 MHz, $CDCl_3$) δ 5.65 (app hept, 1H), 5.04–5.00 (m, 1H), 4.85–4.78 (m, 1H), 4.69–4.63 (m, 1H), 3.76 (s, 3H), 1.95–1.76 (m, 3H).

^{13}C NMR (126 MHz, $CDCl_3$) δ 171.8, 134.0, 123.5, 86.7, 76.6, 52.2, 12.5.

HRMS m/z (GC-CI): calculated for $C_7H_{10}O_3$ $[M]^+$ 142.06245; found 142.06258.

2-Pentyltetrahydrofuran, 4h

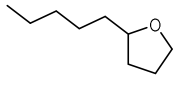

Note: Due to the larger scale, a higher concentration was chosen (1.0 M instead of 0.2 M).

Commercial 2-pentylfuran (1.13 mL, 7.24 mmol, 1.00 eq.), Et_3SiH (2.89 mL, 18.1 mmol, 2.50 eq.) and TfOH (12.8 µL, 0.145 mmol, 2 mol%) in HFIP (7.2 mL, 1.0 M) were subjected to *General Procedure B* for 4 h. Purification by flash column chromatography (SiO_2, EtOAc in *n*-pentane, gradient 2% to 3%) afforded **4h** (865 mg, 6.08 mmol, 84%) as a colorless oil.

R$_f$ 0.44 (*n*-pentane / EtOAc, 95:5).

^{1}H NMR (501 MHz, $CDCl_3$) δ 3.89–3.81 (m, 1H), 3.81–3.74 (m, 1H), 3.70 (td, *J* = 8.0, 6.3 Hz, 1H), 1.96 (m, 1H), 1.85 (m, 2H), 1.61–1.50 (m, 1H), 1.49–1.36 (m, 2H), 1.30 (m, 6H), 0.99–0.84 (m, 3H).

^{13}C NMR (126 MHz, $CDCl_3$) δ 79.6, 67.7, 35.9, 32.1, 31.5, 26.2, 25.9, 22.8, 14.2.

HRMS m/z (GC-CI Ammonia): calculated for $C_9H_{19}O_1$ $[M+H]^+$ 143.14304; found 143.14307.

***trans*-3-(4-(*tert*-Butyl)phenyl)-2-methyltetrahydrofuran, 4j**

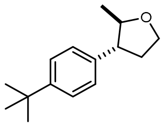

3-(4-(*tert*-Butyl)phenyl)-2-methylfuran **3j** (21.4 mg, 0.10 mmol, 1.00 eq.), Et_3SiH (39.9 µL, 0.25 mmol, 2.50 eq.), distilled H_2O (1.8 µL, 0.10 mmol, 1.00 eq.) and TFA (0.38 µL, 5.0 µmol, 5 mol%) in HFIP (0.50 mL) were subjected to *General Procedure A* for 20 h. Purification by two subsequent column chromatographies (SiO_2, EtOAc in *n*-pentane, gradient 5% to 10%; *then:* 2% MTBE in *n*-pentane) afforded **4j** (15.1 mg, 0.069 mmol, 69%) as a white solid in a diastereomeric mixture of 12:1 with the *cis*-isomer as the minor component.

R$_f$ 0.42 (*n*-pentane / MTBE, 9:1).

^{1}H NMR (501 MHz, $CDCl_3$) δ 7.37–7.32 (m, 2H), 7.19–7.16 (m, 2H), 4.05–4.00 (m, 2H), 3.85 (dq, *J* = 8.6, 6.0 Hz, 1H), 2.79 (app q, *J* = 8.9 Hz, 1H), 2.37 (m, 1H), 2.18–2.08 (m, 1H), 1.32 (s, 9H), 1.23 (d, *J* = 6.0 Hz, 3H).

^{13}C NMR (126 MHz, $CDCl_3$) δ 149.6, 138.5, 127.4, 125.6, 82.3, 67.5, 52.6, 35.5, 34.6, 31.5, 19.2.

HRMS m/z (GC-EI): calculated for $C_{15}H_{22}O_1$ $[M]^+$ 218.16652; found 218.16646.

The relative stereochemistry as well as the minor diastereomer was characterized by analysis of the reaction mixture employing TfOH (2 mol%), delivering inferior diastereoselectivities (d.r. ~ 5:1) compared to above employed TFA:

3-(4-(*tert*-Butyl)phenyl)-2-methylfuran **3j** (21.4 mg, 0.10 mmol, 1.00 eq.), Et_3SiH (39.9 µL, 0.25 mmol, 2.50 eq.) and TfOH (0.18 µL, 0.0020 mmol, 2 mol%) in HFIP (0.50 mL) were subjected to *General Procedure A* for 20 h. Purification by two subsequent column chromatographies (SiO_2, MTBE in *n*-pentane, 2%) afforded a diastereomeric mixture of *cis*- and *trans*-**4j**.

Ethyl *trans*-2-butyltetrahydrofuran-carboxylate, 4k

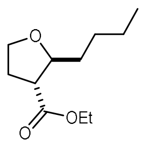

Ethyl 2-butylfuran-3-carboxylate **3k** (29.4 mg, 0.15 mmol, 1.00 eq.), Et_3SiH (59.9 µL, 0.38 mmol, 2.50 eq.) and TfOH (0.27 µL, 3.0 µmol, 2 mol%) in HFIP (0.75 mL) were subjected to *General Procedure B* for 2 h.

Purification by flash column chromatography (SiO_2, Et_2O in *n*-pentane, gradient 5% to 10%) afforded **4k** (20.7 mg, 0.104 mmol, 69%) as a colorless oil.

Note: Under Conditions A only starting material was recovered.

R*f* 0.30 (*n*-pentane / Et_2O, 90:10).

^{1}H NMR (600 MHz, C_6D_6) δ 4.09 (td, *J* = 7.3, 5.0 Hz, 1H), 3.97–3.90 (m, 2H), 3.74–3.65 (m, 2H), 2.49 (ddd, *J* = 9.5, 7.2, 6.6 Hz, 1H), 2.10 (dddd, *J* = 12.3, 7.3, 6.6, 5.7 Hz, 1H), 1.76 (ddtd, *J* = 12.3, 9.5, 7.5, 0.4 Hz, 1H), 1.67–1.59 (m, 2H), 1.55–1.48 (m, 1H), 1.44–1.35 (m, 1H), 1.32–1.24 (m, 2H), 0.93 (t, *J* = 7.1 Hz, 3H), 0.86 (t, *J* = 7.3 Hz, 3H).

^{13}C NMR (150 MHz, C_6D_6) δ 173.6, 128.1, 82.6, 67.5, 60.5, 49.9, 35.3, 30.7, 28.6, 23.0, 14.2.

HRMS m/z (API-MS): calculated for $C_{11}H_{20}O_3Na_1$ $[M+Na]^+$ 223.13046; found 223.13044.

Assignment of relative stereochemistry

Overlapping ^{1}H signals of spectra measured in C_6D_6 or $CDCl_3$ made the assignment of the relative stereochemistry impossible. Therefore, the sample in C_6D_6 was titrated with $CDCl_3$ in order to have all of the signals separated. An approx. 50 vol% solution of $CDCl_3$ in C_6D_6 gave separated signals. 1D selective NOESY experiments with excitation of H4 and H3 showed an intense NOE of H4 to H1'' and NOE of H3 to H1' and H1'' (ratio 3.4:1). Although, considering the high flexibility of 5-membered rings, this data is better in line with a *trans* configuration of the furan ring (for full spectra see Spectra section).

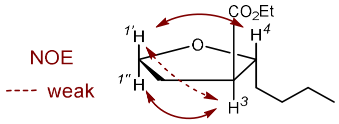

Ethyl 3-(tetrahydrofuran-2-yl)propanoate, 4l

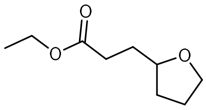

Commercial ethyl 3-(furan-2-yl)propanoate **3l** (67 mg, 0.40 mmol, 1.00 eq.), Et_3SiH (160 µL, 1.00 mmol, 1.50 eq.) and TfOH (0.71 µL, 8.0 µmol, 2 mol%) in HFIP (0.80 mL) were subjected to *General Procedure B* for 2 h. Purification by flash column chromatography (SiO_2, EtOAc in *n*-pentane, gradient 10% to 20%) afforded **4l** (52 mg, 0.30 mmol, 75%) as a colorless oil.

R*f* 0.30 (*n*-pentane / Et_2O, 60:40).

^{1}H NMR (501 MHz, $CDCl_3$) δ 4.12 (app q, 2H), 3.88–3.80 (m, 2H), 3.71 (m, 1H), 2.49–2.32 (m, 2H), 2.06–1.93 (m, 1H), 1.93–1.77 (m, 4H), 1.53–1.41 (m, 1H), 1.25 (t, *J* = 7.1 Hz, 3H).

^{13}C NMR (126 MHz, $CDCl_3$) δ 173.8, 78.4, 76.9, 67.8, 60.5, 31.3, 30.9, 25.8, 14.4.

HRMS m/z (API-MS): calculated for $C_9H_{17}O_3$ $[M+H]^+$ 173.11722; found 173.11731.

3-(Tetrahydrofuran-2-yl)propanoic acid, 4m

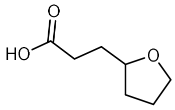

Commercial 3-(furan-2-yl)propanoic acid **3m** (56.1 mg, 0.40 mmol, 1.00 eq.), Et_3SiH (160 µL, 1.00 mmol, 1.50 eq.) and TfOH (0.71 µL, 8.0 µmol, 2 mol%) in HFIP (0.80 mL) were subjected to *General Procedure B* for 5 h. Purification by flash column chromatography (SiO_2, 2% MeOH in DCM with a few drops of AcOH) afforded **4m** (26.0 mg, 0.180 mmol, 45%) as a colorless oil.

R_f 0.20 (2% MeOH in DCM with 1 drop AcOH).

^{1}H NMR (501 MHz, $CDCl_3$) δ 3.95–3.85 (m, 2H), 3.79–3.71 (m, 1H), 2.58–2.43 (m, 2H), 2.07–1.97 (m, 1H), 1.96–1.85 (m, 3H), 1.85–1.77 (m, 1H), 1.56–1.46 (m, 1H).

Note: -COO*H* peak not detected.

^{13}C NMR (126 MHz, $CDCl_3$) δ 178.0, 78.5, 68.0, 31.3, 31.2, 30.4, 25.9.

HRMS m/z (API-MS): calculated for $C_7H_{12}O_3Na_1$ $[M+Na]^+$ 167.06786; found 167.067890.

4-(Tetrahydrofuran-2-yl)benzonitrile, 4n

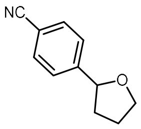

4-(Furan-2-yl)benzonitrile **3n** (25.4 mg, 0.15 mmol, 1.00 eq.), Et_3SiH (59.9 µL, 0.38 mmol, 2.50 eq.) and TfOH (0.27 µL, 3.0 µmol, 2 mol%) in HFIP (0.75 mL) were subjected to *General Procedure B* for 2 h. Purification by flash column chromatography (SiO_2, 20% Et_2O in *n*-pentane) afforded **4n** (21.0 mg, 0.121 mmol, 81%) as a colorless oil.

R_f 0.32 (*n*-pentane / Et_2O, 80:20).

^{1}H NMR (501 MHz, $CDCl_3$) δ 7.77–7.56 (m, 2H), 7.50–7.39 (m, 2H), 4.93 (t, *J* = 7.2 Hz, 1H), 4.09 (dt, *J* = 8.3, 6.7 Hz, 1H), 3.96 (dt, *J* = 8.4, 6.9 Hz, 1H), 2.48–2.30 (m, 1H), 2.07–1.95 (m, 2H), 1.74 (dq, *J* = 12.3, 7.8 Hz, 1H).

^{13}C NMR (126 MHz, $CDCl_3$) δ 149.4, 132.3, 126.3, 119.1, 111.0, 80.0, 69.1, 34.8, 26.1.

HRMS m/z (API-MS): calculated for $C_{11}H_{11}O_1N_1Na_1$ $[M+Na]^+$ 196.07328; found 196.07343.

2-(4-Fluorophenyl)tetrahydrofuran, 4o

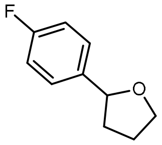

2-(4-Fluorophenyl)furan **3o** (24.3 mg, 0.15 mmol, 1.00 eq.), Et_3SiH (59.9 µL, 0.38 mmol, 2.50 eq.), distilled H_2O (2.7 µL, 0.15 mmol, 1.00 eq.) and TFA (0.57 µL, 7.5 µmol, 5 mol%) in HFIP (0.75 mL) were subjected to *General Procedure A* for 18 h. Purification by flash column chromatography (SiO_2, 5% Et_2O in *n*-pentane) afforded **4o** (22.2 mg, 0.134 mmol, 89%) as a colorless oil.

Note: Under Conditions B a messy reaction profile was obtained.

R_f 0.37 (*n*-pentane / Et_2O, 90:10).

^{1}H NMR (501 MHz, $CDCl_3$) δ 7.32–7.27 (m, 2H), 7.05–6.98 (m, 2H), 4.85 (t, *J* = 7.2 Hz, 1H), 4.09 (dt, *J* = 8.2, 6.8 Hz, 1H), 3.92 (td, *J* = 7.9, 6.5 Hz, 1H), 2.31 (dtd, *J* = 12.5, 7.2, 5.6 Hz, 1H), 2.08–1.94 (m, 2H), 1.76 (dq, *J* = 12.3, 7.9 Hz, 1H).

^{13}C NMR (126 MHz, $CDCl_3$) δ 162.2 (d, *J* = 244.7 Hz), 139.2 (d, *J* = 3.2 Hz), 127.4 (d, *J* = 7.9 Hz), 115.2 (d, *J* = 21.2 Hz), 80.3, 68.8, 34.8, 26.2.

2-(3-(Trifluoromethyl)phenyl)tetrahydrofuran, 4p

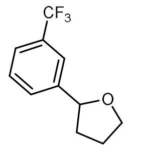

2-(3-(Trifluoromethyl)phenyl)furan **3p** (31.8 mg, 0.15 mmol, 1.00 eq.), Et_3SiH (59.9 µL, 0.38 mmol, 2.50 eq.) and TfOH (0.27 µL, 3.0 µmol, 2 mol%) in HFIP (0.75 mL) were subjected to *General Procedure B* for 2 h. Purification by flash column chromatography (SiO_2, 5% Et_2O in *n*-pentane) afforded **4p** (27.0 mg, 0.125 mmol, 83%) as a colorless oil.

Note: Under Conditions A the reaction took 3 days until full conversion of starting material.

R_f 0.21 (*n*-pentane / Et_2O, 95:5).

^{1}H NMR (501 MHz, $CDCl_3$) δ 7.65–7.62 (m, 1H), 7.56–7.52 (m, 2H), 7.50–7.44 (m, 1H), 4.96 (t, *J* = 7.2 Hz, 1H), 4.14 (dt, *J* = 8.4, 6.9 Hz, 1H), 3.99 (dt, *J* = 8.4, 7.0 Hz, 1H), 2.40 (dq, *J* = 12.7, 6.7, Hz, 1H), 2.19–1.95 (m, 2H), 1.81 (dq, *J* = 7.8, 12.2 Hz, 1H).

^{13}C NMR (126 MHz, $CDCl_3$) δ 144.8, 130.8 (q, *J* = 32.3 Hz), 129.1 (q, *J* = 1.3 Hz), 128.9, 124.4 (q, *J* = 272.4 Hz), 124.1 (q, *J* = 3.8 Hz), 122.5 (q, *J* = 3.9 Hz), 80.2, 69.0, 34.9, 26.1.

^{19}F NMR (471 MHz, $CDCl_3$) δ –62.6.

HRMS m/z (GC-EI): calculated for $C_{11}H_{11}O_1F_3$ $[M]^+$ 216.07565; found 216.07572.

2-Phenyltetrahydrofuran, 4q

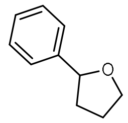

Commercial 2-phenylfuran **3q** (57.7 mg, 0.40 mmol, 1.00 eq.), Et_3SiH (160 µL, 1.00 mmol, 2.50 eq.), distilled H_2O (7.2 µL, 0.40 mmol, 1.00 eq.) and TFA (1.53 µL, 20 µmol, 5 mol%) in HFIP (2.0 mL) were subjected to *General Procedure A* for 12 h. Purification by flash column chromatography (SiO_2, 5% Et_2O in *n*-pentane) afforded **4q** (44.5 mg, 0.30 mmol, 75%) as a yellow oil.

R*f* 0.30 (*n*-pentane / Et_2O, 9:1).

^{1}H NMR (501 MHz, $CDCl_3$) δ 7.36–7.31 (m, 4H), 7.29–7.21 (m, 1H, *overlap with $CDCl_3$ peak*), 4.89 (t, *J* = 7.2 Hz, 1H), 4.10 (dt, *J* = 8.3, 6.8 Hz, 1H), 3.94 (td, *J* = 7.9, 6.4 Hz, 1H), 2.33 (dtd, *J* = 12.4, 7.2, 5.4 Hz, 1H), 2.07–1.94 (m, 2H), 1.81 (ddt, *J* = 7.6, 8.6, 12.2 Hz, 1H).

^{13}C NMR (126 MHz, $CDCl_3$) δ 143.6, 128.4, 127.3, 125.8, 80.8, 68.8, 34.8, 26.2.

HRMS m/z (GC-EI): calculated for $C_{10}H_{12}O_1$ $[M]^+$ 148.08827; found 148.08835.

γ-Valerolactone, 4t

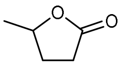

Et_3SiH (479 µL, 3.00 mmol, 3.00 eq.) and TfOH (1.77 µL, 20 µmol, 2 mol%) in HFIP (1.0 mL) were dissolved in HFIP (0.50 mL). To that, a solution of levulinic acid **3t** (103 µL, 1.00 mmol, 1.00 eq.) in HFIP (0.50 mL, final concentration 1.0 M) was added dropwise at room temperature. The reaction was allowed to stirr for 20 h at room temperature. Then, the reaction was quenched with one drop of NEt_3. The resulting mixture was biphasic; the silane layer was discarded (containing no product), and only the HFIP layer was further used. The HFIP was removed under reduced pressure and the obtained residue was purified by flash column chromatography (SiO_2, gradient EtOAc in *n*-pentane, 20% to 100%) to afford γ-valerolactone **4t** (90.0 mg, 0.90 mmol, 89%) as a colorless oil.

R*f* 0.30 (*n*-pentane / EtOAc, 1:1) [$KMnO_4$ stain].

^{1}H NMR (501 MHz, $CDCl_3$) δ 4.63 (m, 1H), 2.64–2.46 (m, 2H), 2.42–2.29 (m, 1H), 1.82 (dtd, *J* = 12.7, 9.5, 7.9 Hz, 1H), 1.41 (d, *J* = 6.2 Hz, 3H).

^{13}C NMR (126 MHz, $CDCl_3$) δ 177.3, 77.4, 29.8, 29.2, 21.2.

HRMS m/z (API-MS): calculated for $C_5H_8O_2Na_1$ $[M+Na]^+$ 123.04165; found 123.04163.

2,3-Dihydrobenzofuran 4u

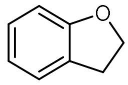

Commercial 2,3-benzofuran **3u** (33 µL, 0.30 mmol, 1.00 eq.), Et_3SiH (72 µL, 0.45 mmol, 1.50 eq.), and TfOH (0.53 µL, 6.0 µmol, 2 mol%) in HFIP (0.75 mL) were subjected to *General Procedure B* for 10 h. In this case, the benzofuran was added as a solution in HFIP to the reaction mixture (additional 0.75 mL, final concentration: 0.20 M). Purification by flash column chromatography (SiO_2, 5% to 10% Et_2O in *n*-pentane) afforded **4u** (19.8 mg, 0.165 mmol, 55%) as a colorless oil.

R*f* 0.33 (*n*-pentane / Et_2O, 95:5).

^{1}H NMR (600 MHz, $CDCl_3$) δ 7.22 – 7.17 (m, 1H), 7.11 (dddt, J = 0.8, 1.5, 7.5, 8.2 Hz, 1H), 6.85 (td, J = 1.0, 7.4 Hz, 1H), 6.80 (ddq, J = 0.5, 1.0, 8.0 Hz, 1H), 4.58 – 4.54 (app t, 2H), 3.22 (app t, 2H).

^{13}C NMR (151 MHz, $CDCl_3$) δ 160.1, 128.1, 127.0, 125.0, 120.5, 109.5, 71.1, 29.9.

HRMS m/z (GC-EI): calculated for $C_8H_8O_1$ $[M]^+$ 120.05697; found 120.05708.

11. Examples Characterized Within Crude Mixtures

3-Methyl-2,5-dihydrofuran, 2l

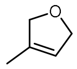

Commercial 3-methylfuran **1l** (32.8 mg, 0.40 mmol, 1.00 eq.), Et_3SiH (95.8 µL, 0.60 mmol, 1.50 eq.) and TfOH (0.71 µL, 8.0 µmol, 2 mol%) in HFIP (2.0 mL) were subjected to *General Procedure B* for 4 h. The solvents were removed under reduced pressure. Then, mesitylene (18.4 µL, 0.132 mmol, 0.33 eq.) was added as an internal standard to determine an ^{1}H NMR yield of **2l** (quantitative). Due to volatility, no further purification was performed.

^{1}H NMR (501 MHz, $CDCl_3$) δ 5.46 (m, 1H), 4.64 (m, 2H), 4.49 (m, 2H), 1.76 (m, 3H).

^{13}C NMR (126 MHz, $CDCl_3$) δ 135.4, 119.5, 78.1, 76.3, 12.2.

HRMS m/z (GC-EI): calculated for $C_5H_8O_1$ $[M]^+$ 84.05697; found 84.05692.

3-(Chloromethyl)-2,5-dihydrofuran, 2m

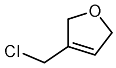

Note: Addition sequence was altered due to instability of the substrate in HFIP:

Et_3SiH (47.9 µL, 0.30 mmol, 1.50 eq.) and TfOH (0.35 µL, 4.0 µmol, 2 mol%) were dissolved in HFIP (1.0 mL). This mixture was added in one portion to neat 3-(chloromethyl)furan **1m** (23.3 mg, 0.20 mmol, 1.00 eq.) under stirring. The mixture was allowed to stir for 15 min. Finally, a drop of NEt_3 was added. Then, the solvents were removed under reduced pressure. Mesitylene (9.1 µL, 0.065 mmol, 0.33 eq.) was added as an internal standard to determine an ^{1}H NMR yield of 59%. The crude reaction mixture was purified by flash column chromatography (SiO_2, 10% MTBE in *n*-pentane) to afford a sample of **2m** for NMR-characterization containing residual MTBE and *n*-pentane.

Note: Compound is volatile. Characterization was conducted with residual MTBE and *n*-pentane.

R_f 0.21 (*n*-pentane / MTBE, 9:1).

^{1}H NMR (600 MHz, CD2Cl2) δ 5.92–5.89 (m, 1H), 4.67–4.60 (m, 4H), 4.22–4.19 (m, 2H).

^{13}C NMR (151 MHz, CD_2Cl_2) δ 136.8, 125.7, 76.2, 75.5, 39.6.

HRMS m/z (GC-CI Ammonia): calculated for $C_5H_7O_1Cl_1$ $[M]^+$ 118.01799; found 118.01812.

2,5-Dihydrofuran, 2u

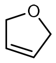

Employing PMHS

Poly(methylhydrosiloxane) PMHS; average Mn 1700-3200 (7.30 mL, 22.0 mmol, 1.50 eq.) was suspended in HFIP (14.6 mL). TfOH (26.0 µL, 0.29 mmol, 2 mol%) was added under rapid stirring at room temperature. Then, furan **1u** (1.07 mL, 14.7 mmol, 1.00 eq.) was added dropwise at room temperature. The reaction was allowed to stirr for 8 h. Mesitylene (0.67 mL, 4.85 mmol, 0.33 eq.) was added as an internal standard and after vigorous mixing an aliquot was taken to determine an ^{1}H NMR yield of **2u** in $CDCl_3$ (88%). For collecting a set of ^{13}C- and 2D-NMR-spectra the volatile HFIP portion was removed (150 mbar, 30 °C) leaving product **2u**, residual HFIP and polymeric Silane left.

Note: Upon addition of TfOH the PMHS-HFIP suspension becomes clear, turning into a deep-red color after addition of the furan (see Figure S13).

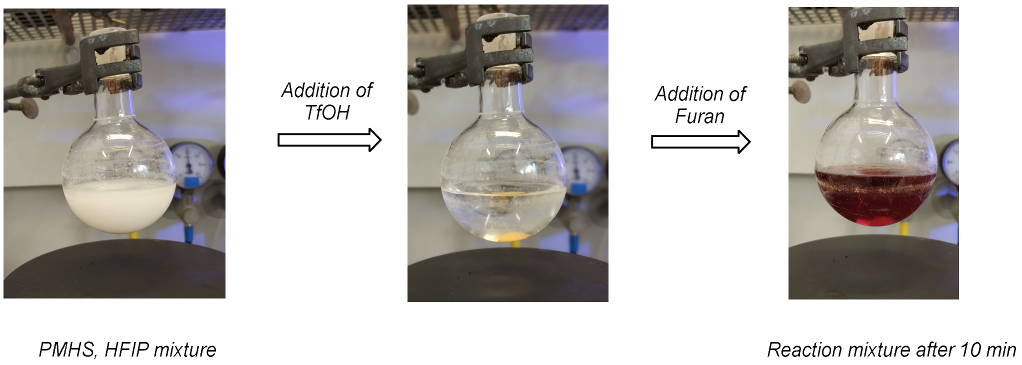

Figure S13. Visualization of the reaction.

Employing Et_3SiH

The reaction can similarly be conducted using Et_3SiH (3.52 mL, 22.0 mmol, 1.50 eq.) in above described procedure affording 90% of 2,5-dihydrofuran (NMR yield).

^{1}H NMR (501 MHz, $CDCl_3$) δ 5.91–5.89 (m, 2H), 4.68–4.66 (m, 2H).

^{13}C NMR (126 MHz, $CDCl_3$) δ 125.7, 75.6.

The obtained spectral data is in accordance with the literature (slight shift observed due to HFIP complexation) and the spectra shown on p. 135.[27]

2-Methyltetrahydrofuran, 3r

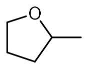

Commercial 2-methylfuran **3i** (36.1 µL, 0.40 mmol, 1.00 eq.), Et_3SiH (160 µL, 1.00 mmol, 2.50 eq.) and TfOH (0.71 µL, 8.0 µmol, 2 mol%) in HFIP (2.0 mL) were subjected to *General Procedure B* for 4 h. The solvents

were removed under reduced pressure. Then mesitylene (18.4 µL, 0.132 mmol, 0.33 eq.) was added as an internal standard to determine an ^{1}H NMR yield of **4r** (83%). Due to volatility, no further purification was performed (see p. 135 for crude spectra).

Observed ^{1}H NMR chemical shifts within the crude reaction mixture spectra match to reported data.[28] The spectrum is depicted on p. 137.

cis/trans-2,5-Dimethyltetrahydrofuran, 4s

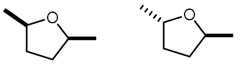

Commercial 2,5-dimethylfuran (42.6 µL, 0.40 mmol, 1.00 eq.), Et_3SiH (160 µL, 1.00 mmol, 2.50 eq.) and TfOH (0.71 µL, 8.0 µmol, 2 mol%) in HFIP (2.0 mL) were subjected to *General Procedure B* for 4 h. The solvents were removed under reduced pressure. Due to volatility, no further purification was performed (see p. 138 for crude spectra).

Observed ^{1}H NMR chemical shifts of **4s** within the crude reaction mixture spectra match to reported data.[29] The spectrum is depicted on p. 138 with the integration of the diastereomeric ratio dr = 1:1.6 (*trans*:*cis*) and a ^{1}H NMR yield of **4s** of 81% relative to DCM as internal standard (1.00 eq. added).

cis/trans-2,5-Dimethyltetrahydrofuran from hydroxymethylfurfural (HMF), 4s

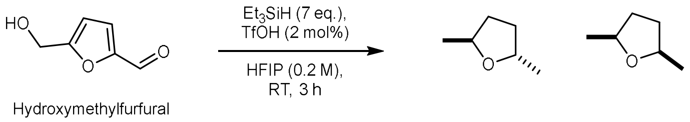

Note: The addition rate was found crucial in preventing the formation of oligomeric impurities.

Et_3SiH (559 µL, 3.50 mmol, 7.00 eq.) and TfOH (0.88 µL, 10 µmol, 2 mol%) were dissolved in HFIP (2.0 mL). Hydroxymethylfurfural HMF **3s** (63 mg, 0.50 mmol, 1.00 eq.) was added as a solution in HFIP (0.5 mL) under rapid stirring with a syringe pump (rate 20 µL min^{-1}) and then stirred for further 3 h. The solvents were removed under reduced pressure. Mesitylene (23.0 µL, 0.165 mmol, 0.33 eq.) was added as an internal standard to determine an ^{1}H NMR yield of **4s**. A near-quantitative ^{1}H NMR yield (>95%) was determined. Due to volatility, no further purification was performed (see p. 138 for crude spectra).

Observed ^{1}H NMR chemical shifts within the crude reaction mixture spectra matches the reported data of [29] of a *cis/trans* mixture of 2,5-dimethyltetrahydrofuran **4s**. The spectrum is depicted on p. 138 with the integration of the diastereomeric ratio dr = 1:1.7 (*trans*:*cis*). In general, a less clean profile compared to 2,5-dimethylfuran was obtained and is dependent on the addition rate of the HMF.

2-Methyltetrahydrofuran from Furfural, 4r

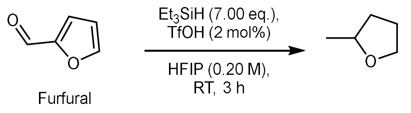

Et_3SiH (319 µL, 2.00 mmol, 5.00 eq.) and TfOH (0.71 µL, 8.0 µmol, 2 mol%) were dissolved in HFIP (2.0 mL). Commercial furfural **3r** (33.1 µL, 0.40 mmol, 1.00 eq.) was added as a solution in HFIP (0.5 mL) under rapid stirring with a syringe pump (rate 20 µL min^{-1}) and then stirred for further 3 h. The solvents were removed under reduced pressure. Mesitylene (18.4 µL, 0.132 mmol, 0.33 eq.) was added as an internal standard to determine an ^1H NMR yield. An approximate ^1H NMR yield of approximately 77 to 83% was determined. Due to volatility, no further purification was performed. Observed ^1H NMR chemical shifts within the crude reaction mixture spectra match to reported data of **4r**.[28] The spectrum is depicted on p. 137.

12. Derivatisation

4-Phenylbutanol

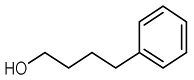

2-Phenylfuran (57.7 mg, 0.40 mmol, 1.00 eq.) was dissolved in HFIP (2.0 mL, 0.2 M) and Et_3SiH (319 µL, 2.00 mmol, 5.00 eq.) and TFA (1.53 µL, 20 µmol, 5 mol%) was added. The mixture was stirred for 12 h and afterwards a portion of TfOH (0.72 µL, 0.0080 mmol, 2 mol%) was added. Then, the mixture was heated to 50 °C for 7 h. Finally, the reaction was allowed to cool down, a drop of NEt_3 was added and the solvents were removed under reduced pressure. The remaining volatiles were removed under a high vacuum (10^{-3} mbar) to yield the product 4-phenylbutanol (58 mg, 0.388 mmol, 97%) in sufficient purity (~95% NMR purity).

R*f* 0.24 (*n*-pentane / Et_2O, 80:20)

^{1}H NMR (501 MHz, $CDCl_3$) δ 7.31–7.26 (m, 2H), 7.19 (m, 3H), 3.66 (t, *J* = 6.5 Hz, 2H), 2.65 (t, *J* = 7.6 Hz, 2H), 1.76–1.67 (m, 2H), 1.66–1.57 (m, 2H).

Note: –OH peak not observed.

^{13}C NMR (126 MHz, $CDCl_3$) δ 142.4, 128.54, 128.46, 125.9, 63.1, 35.8, 32.3, 27.6.

HRMS m/z (GC-EI): calculated for $C_{10}H_{14}O_1$ $[M]^+$ 150.10392; found 150.10394.

13. Spectra

3-(4-Methoxyphenyl)furan, 1a / 3a

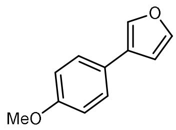

^{1}H NMR (501 MHz, $CDCl_3$)

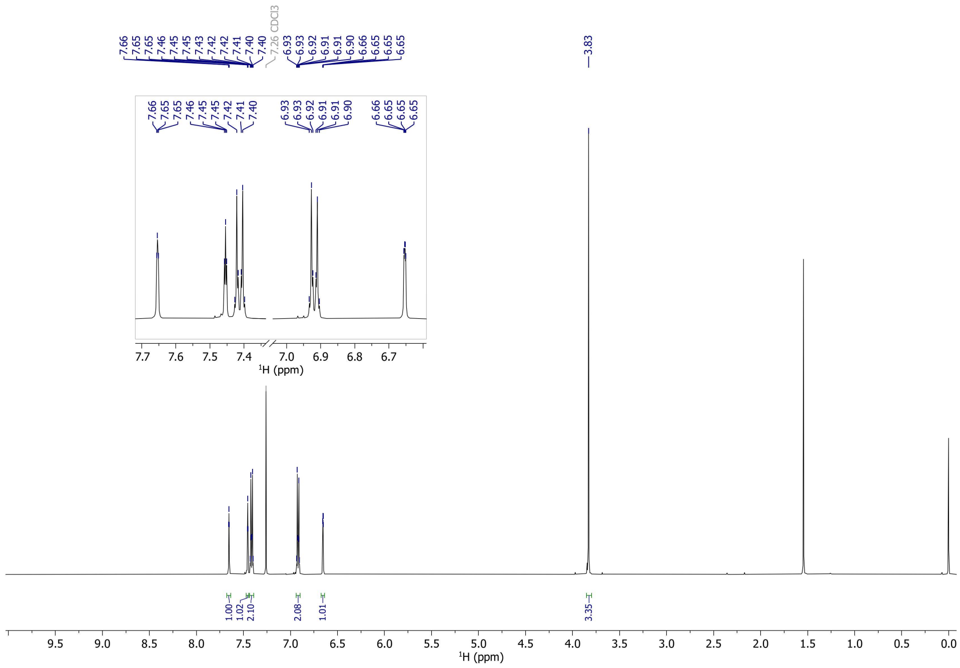

^{13}C NMR (126 MHz, $CDCl_3$)

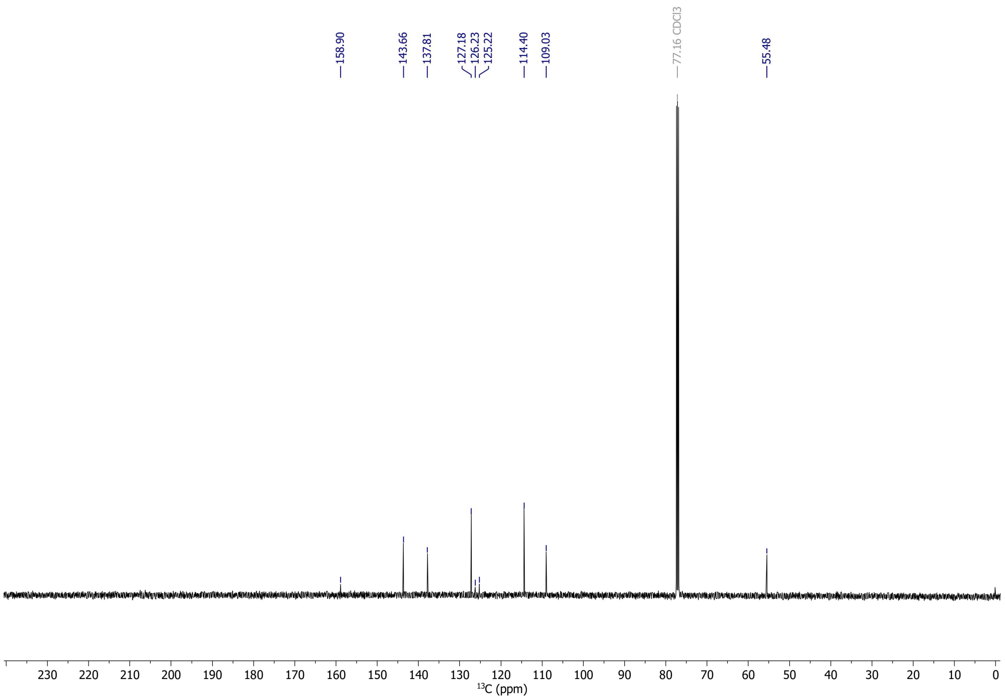

3-(4-(*Tert*-butyl)phenyl)furan, 1b / 3b

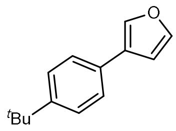

^{1}H NMR (501 MHz, $CDCl_3$)

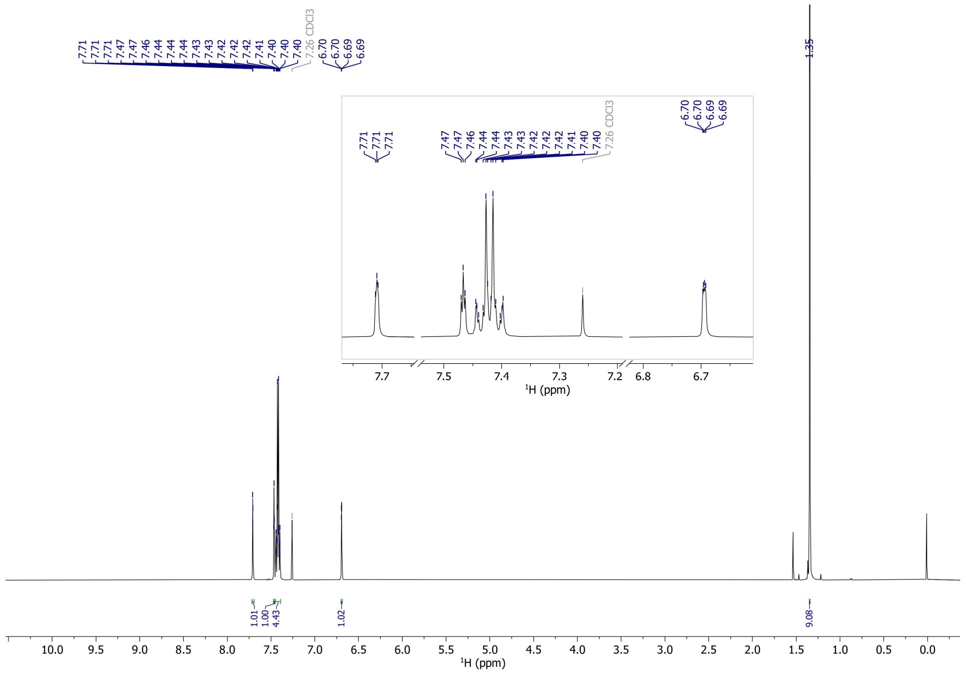

^{13}C NMR (126 MHz, $CDCl_3$)

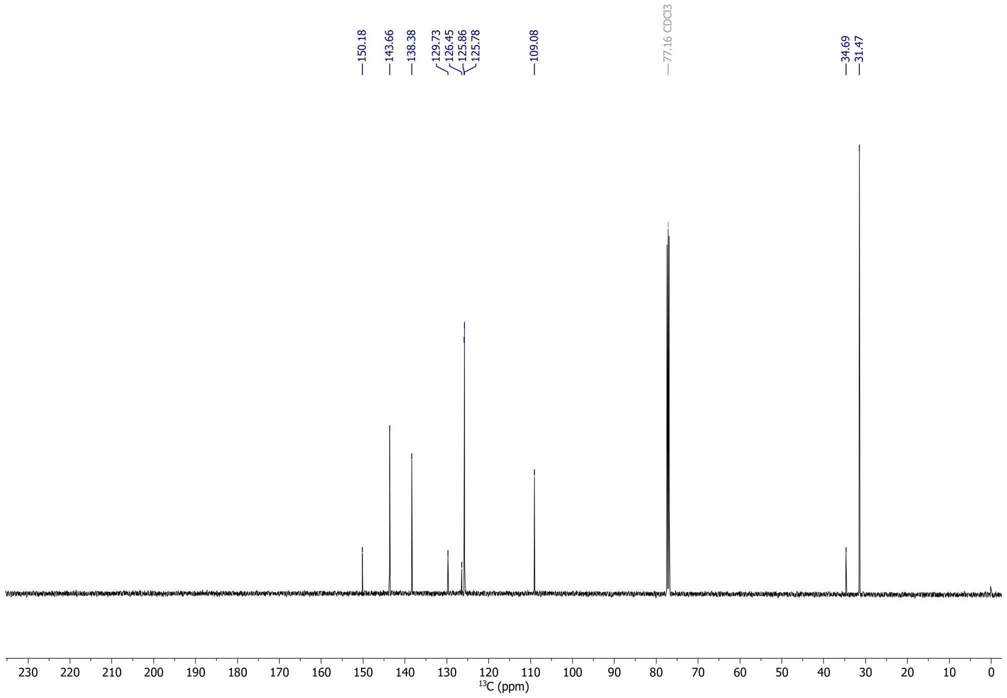

3-(*para*-Tolyl)furan, 1c / 3c

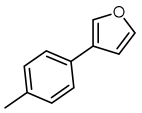

^{1}H NMR (501 MHz, $CDCl_3$)

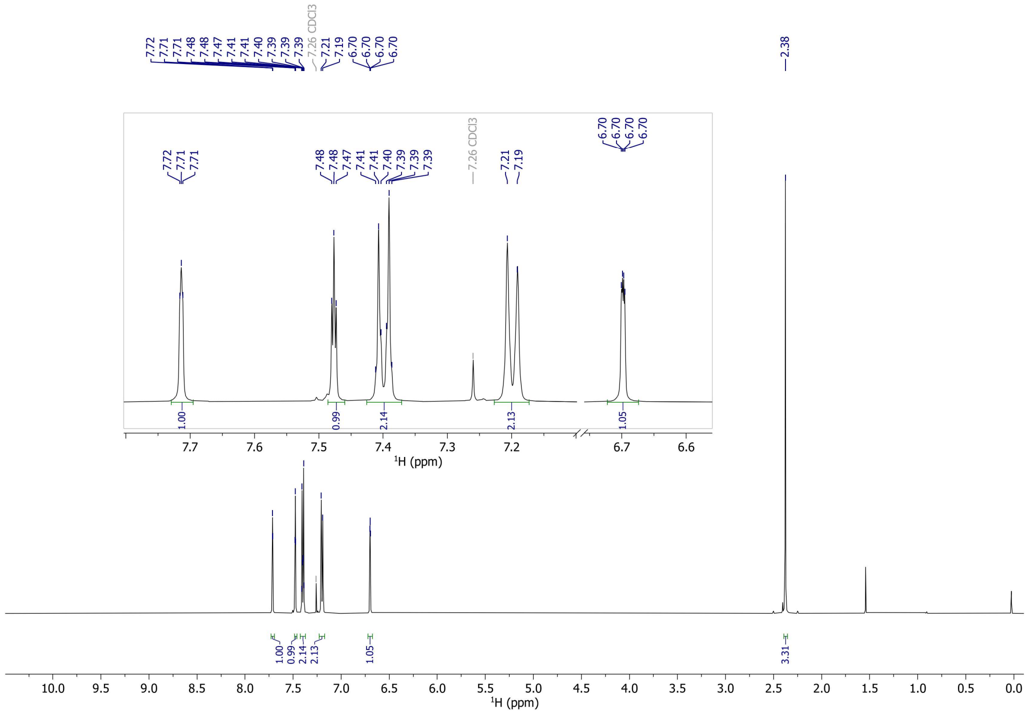

^{13}C NMR (126 MHz, $CDCl_3$)

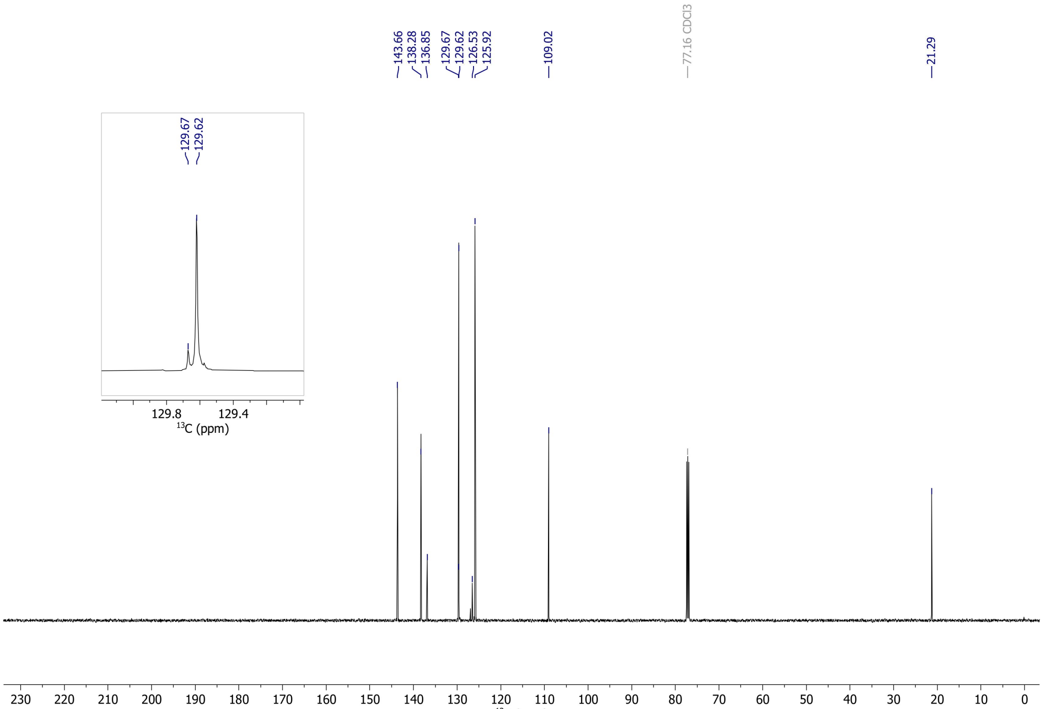

3-Phenylfuran, 1d / 3d

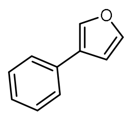

^{1}H NMR (501 MHz, $CDCl_3$)

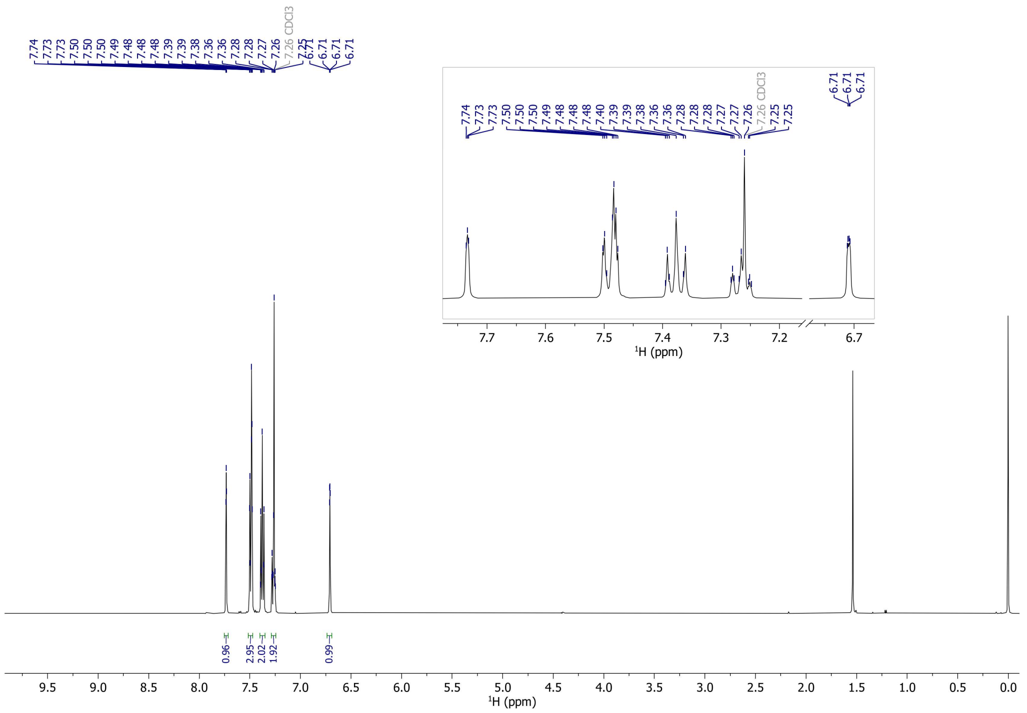

^{13}C NMR (126 MHz, $CDCl_3$)

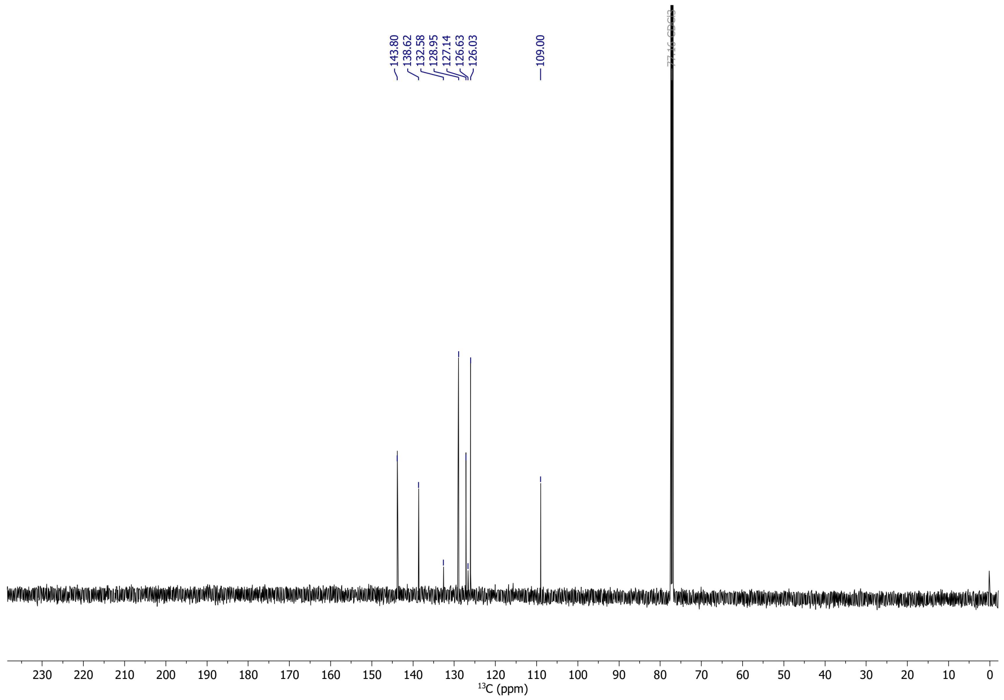

3-(4-Chlorophenyl)furan, 1e / 3e

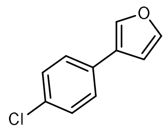

^{1}H NMR (501 MHz, $CDCl_3$)

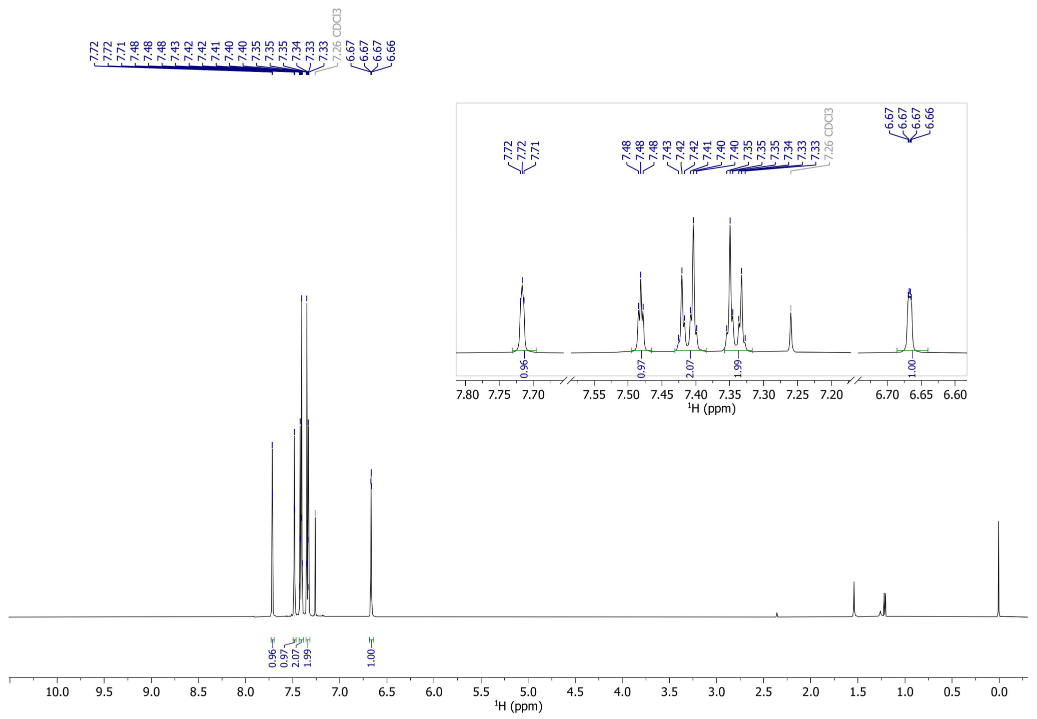

^{13}C NMR (126 MHz, $CDCl_3$)

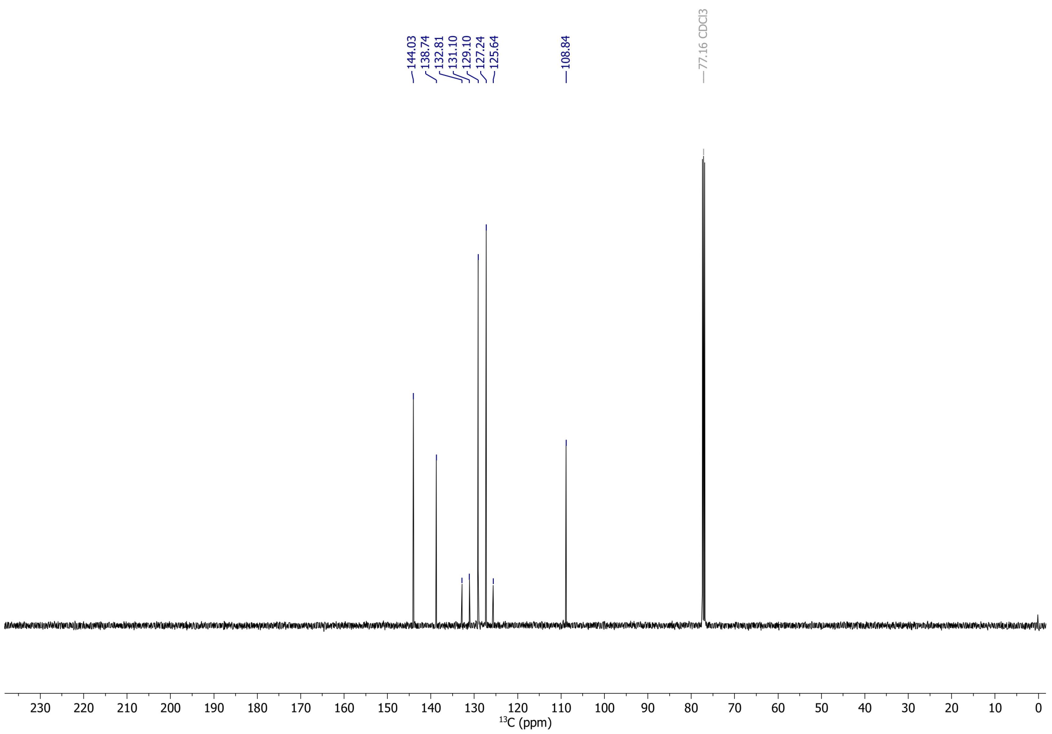

3-(4-(Trifluoromethyl)phenyl)furan, 1f / 3f

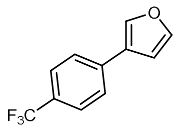

¹H NMR (501 MHz, $CDCl_3$)

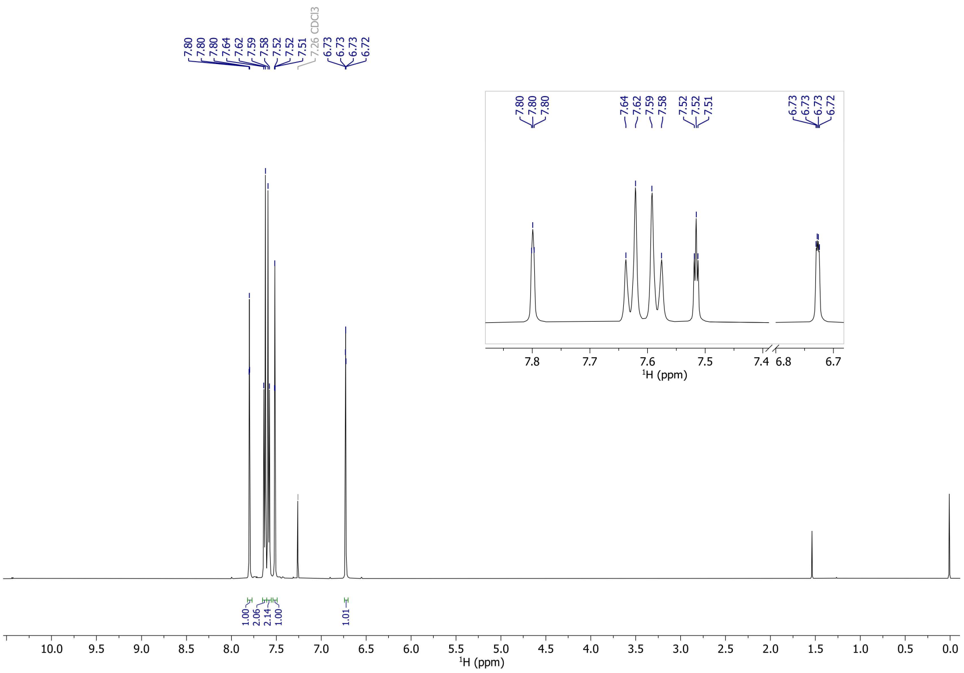

¹³C NMR (126 MHz, $CDCl_3$)

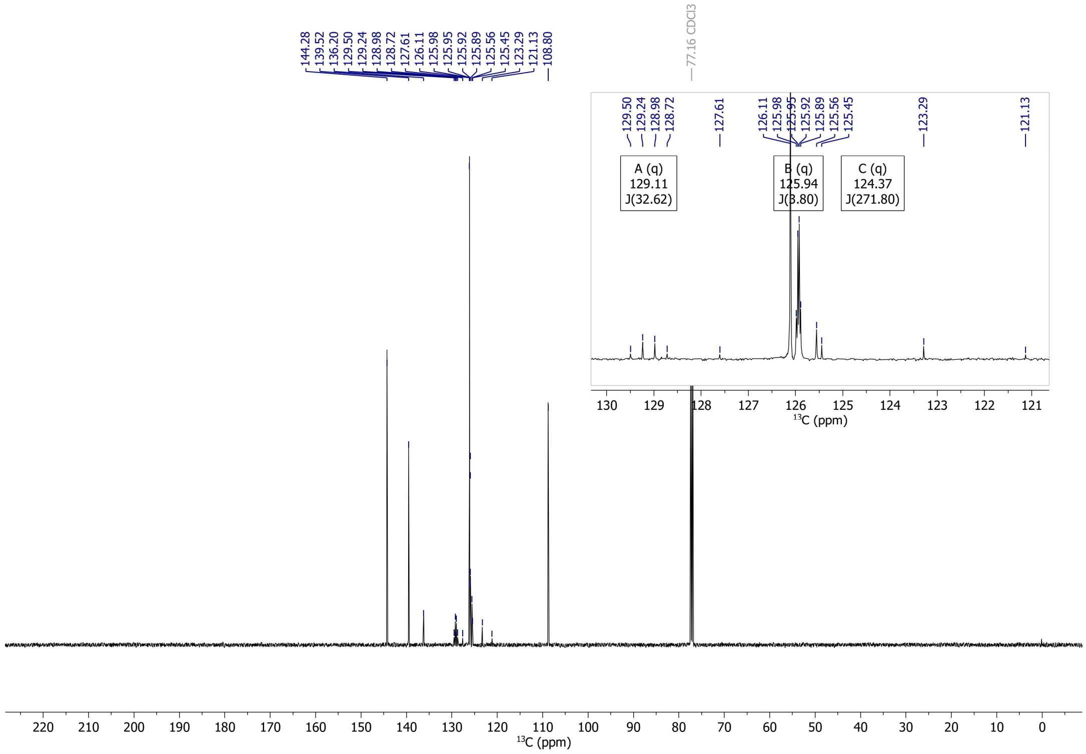

^{19}F NMR (471 MHz, $CDCl_3$)

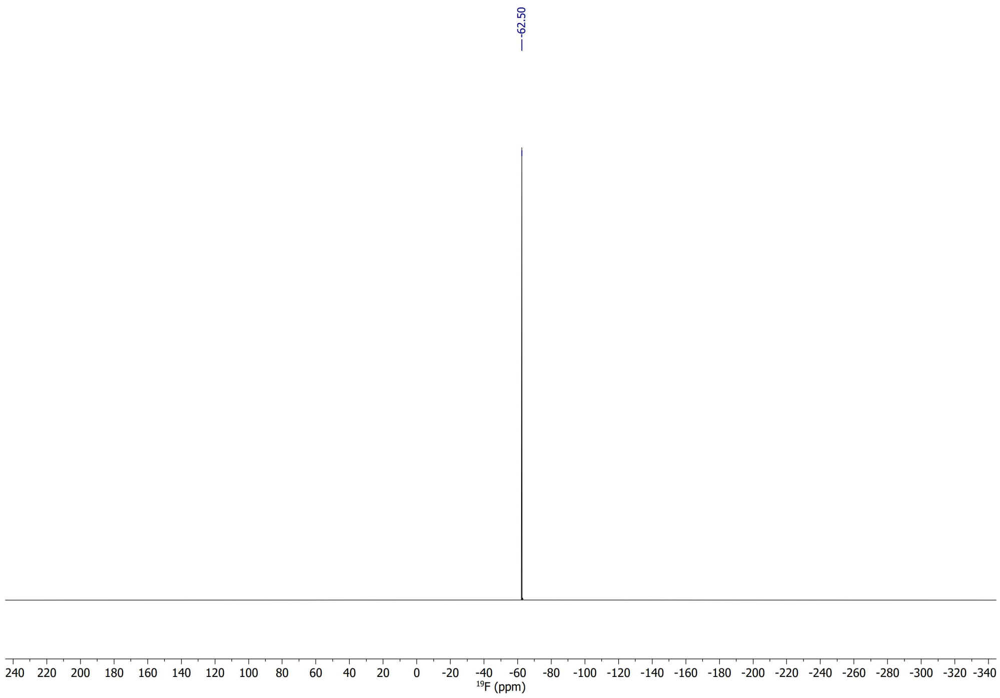

3-(4-Nitrophenyl)furan, 1g / 3g

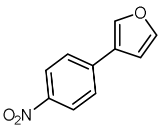

^{1}H NMR (501 MHz, $CDCl_3$)

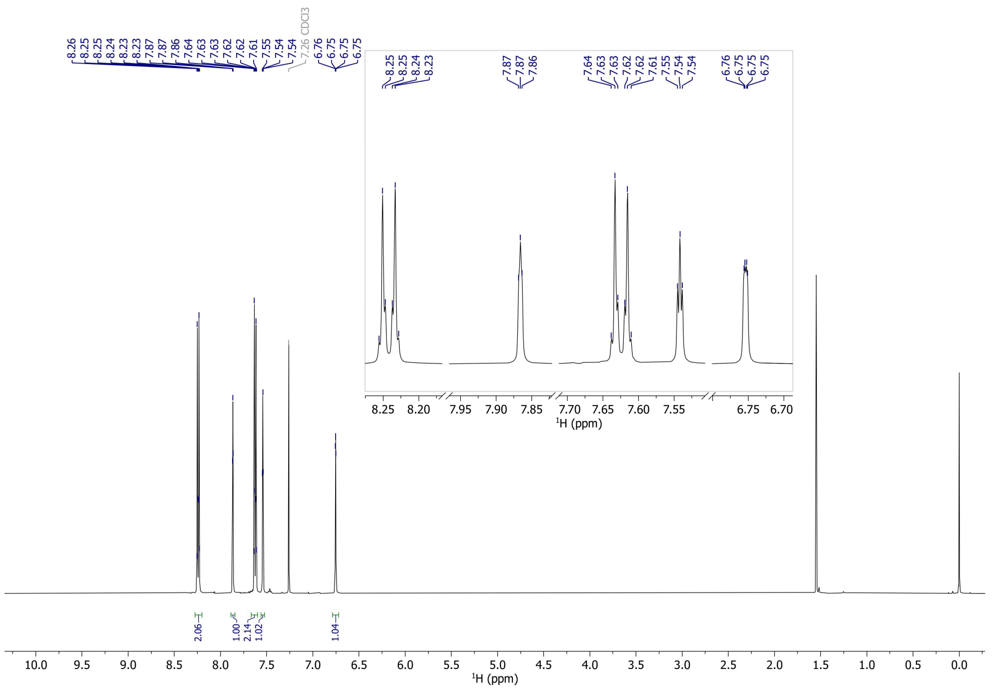

^{13}C NMR (126 MHz, $CDCl_3$)

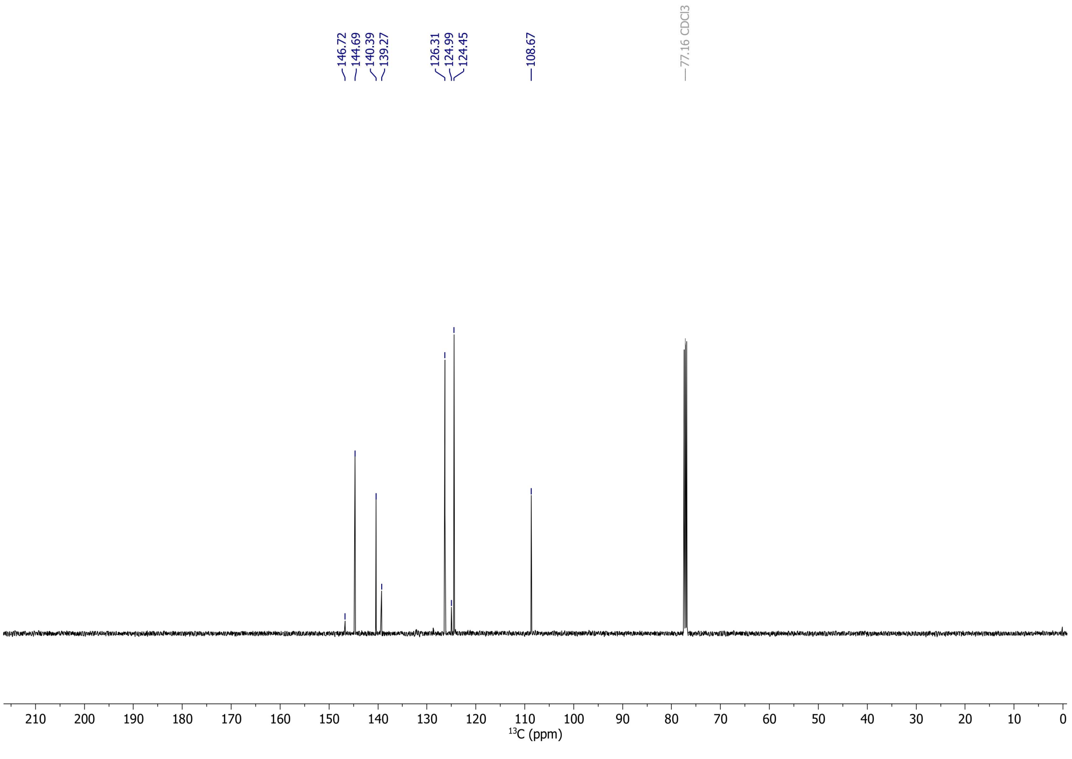

3-(Cyclohexylmethyl)furan, 1h

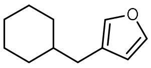

^{1}H NMR (501 MHz, $CDCl_3$)

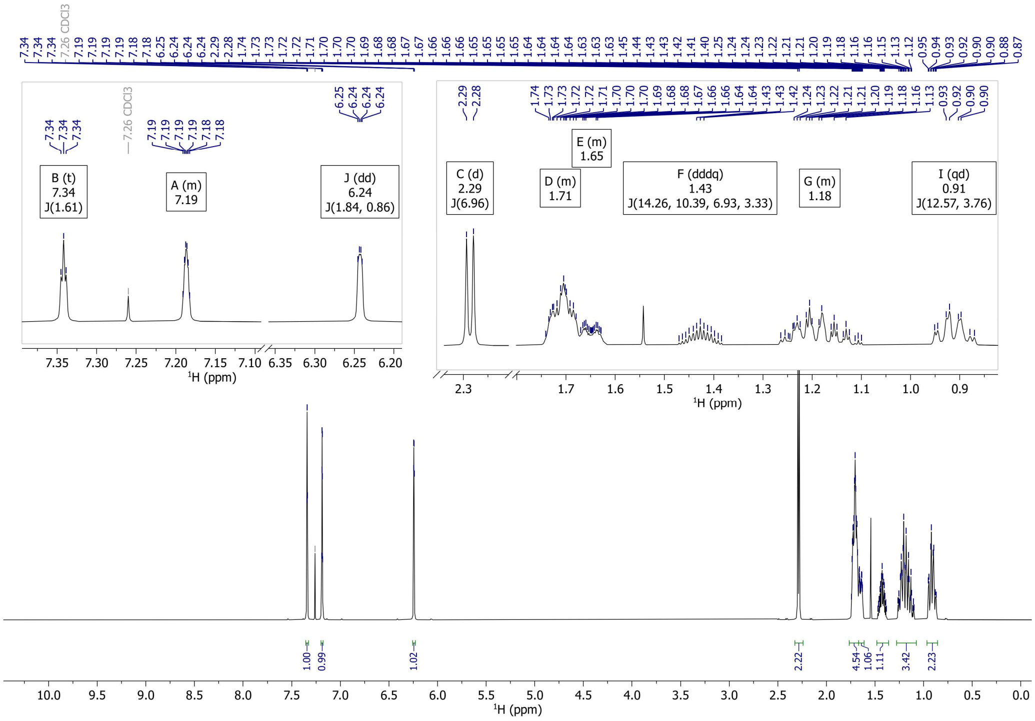

^{13}C NMR (126 MHz, $CDCl_3$)

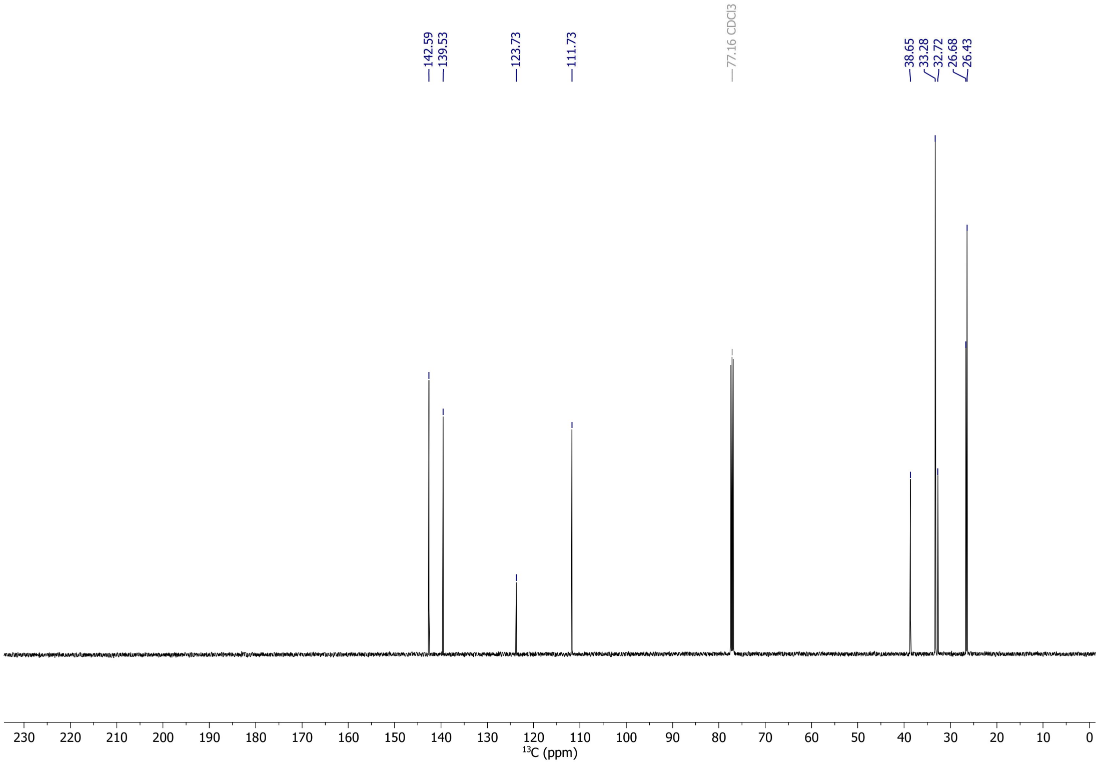

3-Heptylfuran, 1i

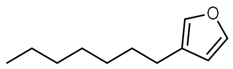

^{1}H NMR (501 MHz, $CDCl_3$)

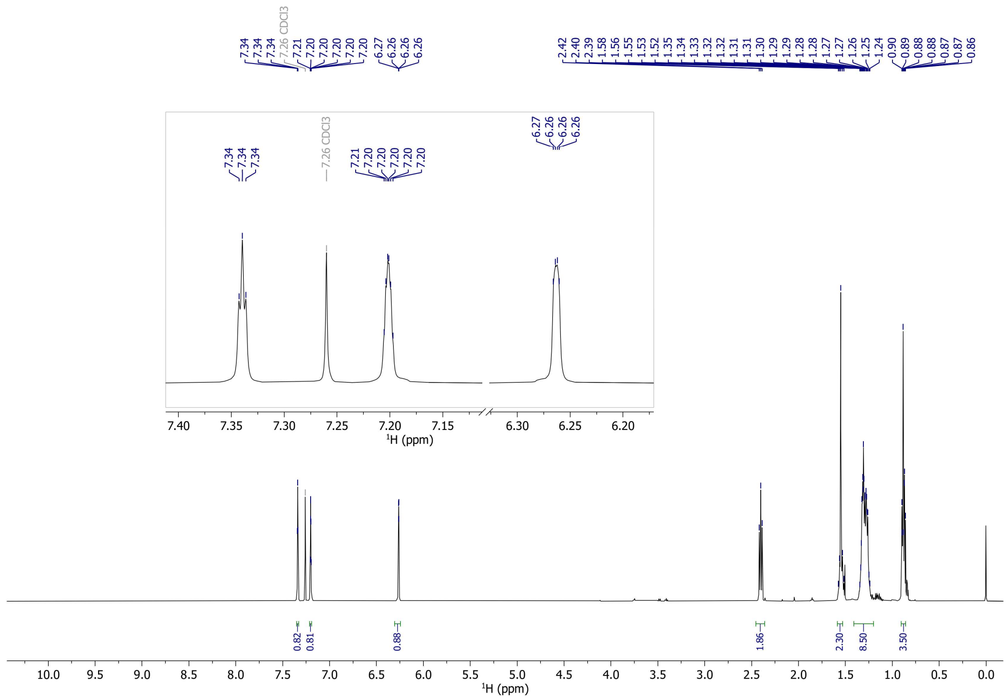

^{13}C NMR (126 MHz, $CDCl_3$)

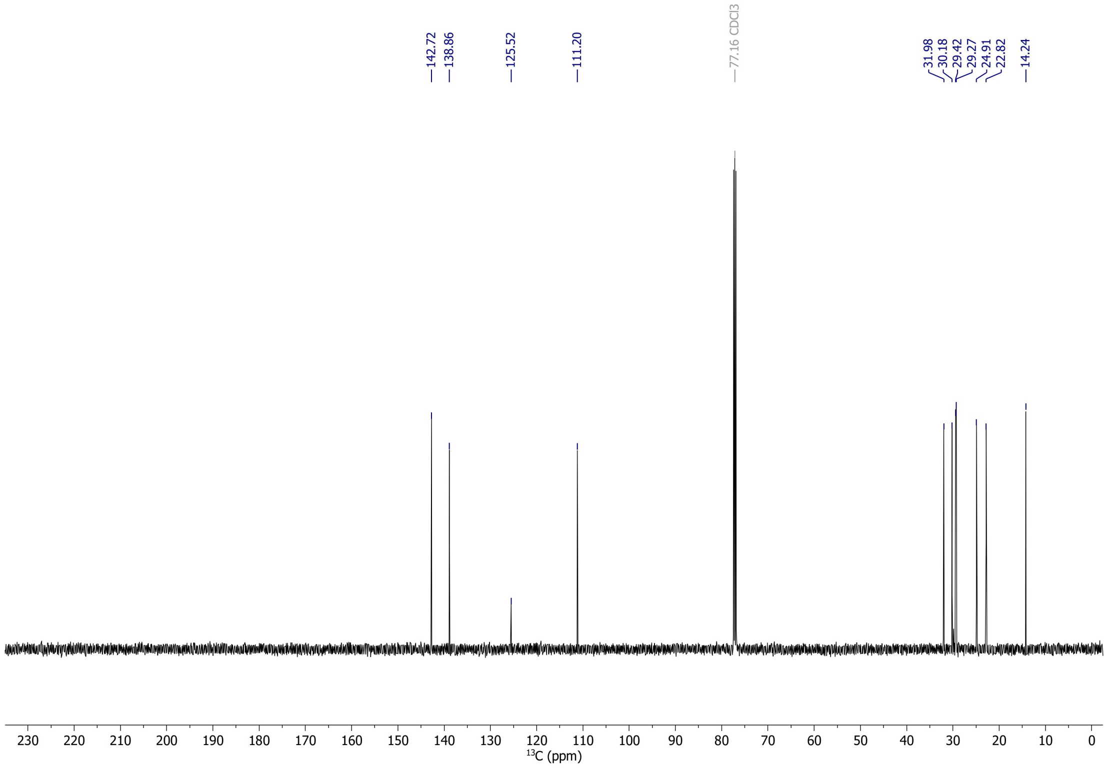

3-Cyclobutylfuran, 1j

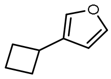

^{1}H NMR (501 MHz, $CDCl_3$)

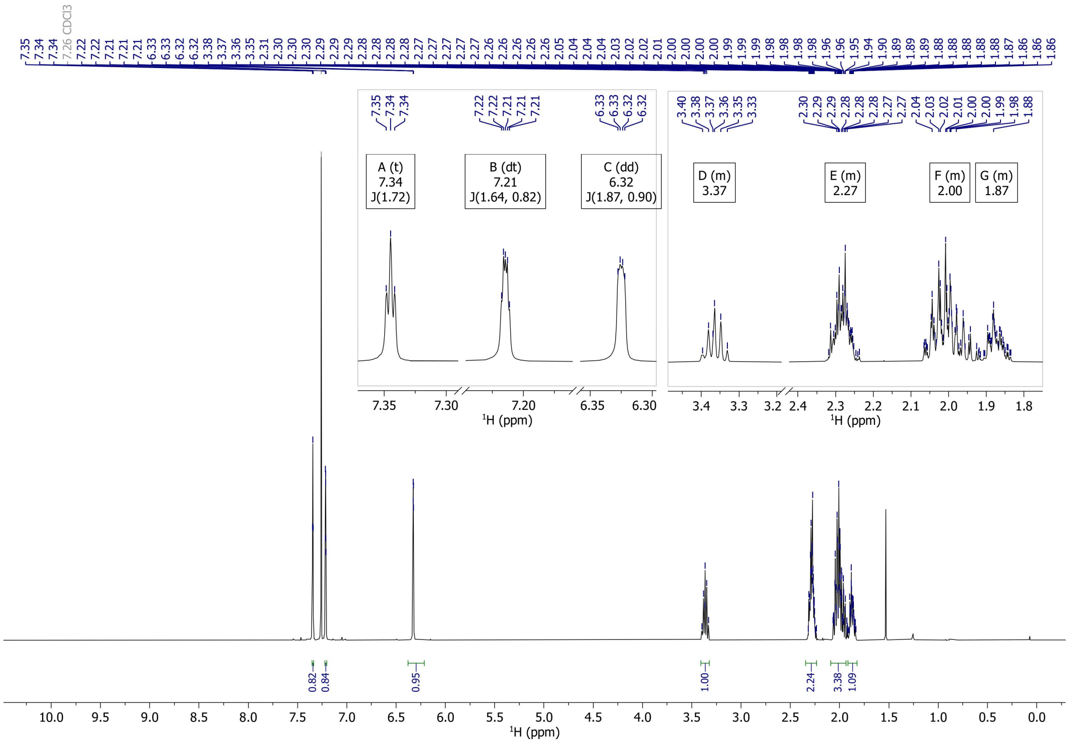

^{13}C NMR (126 MHz, $CDCl_3$)

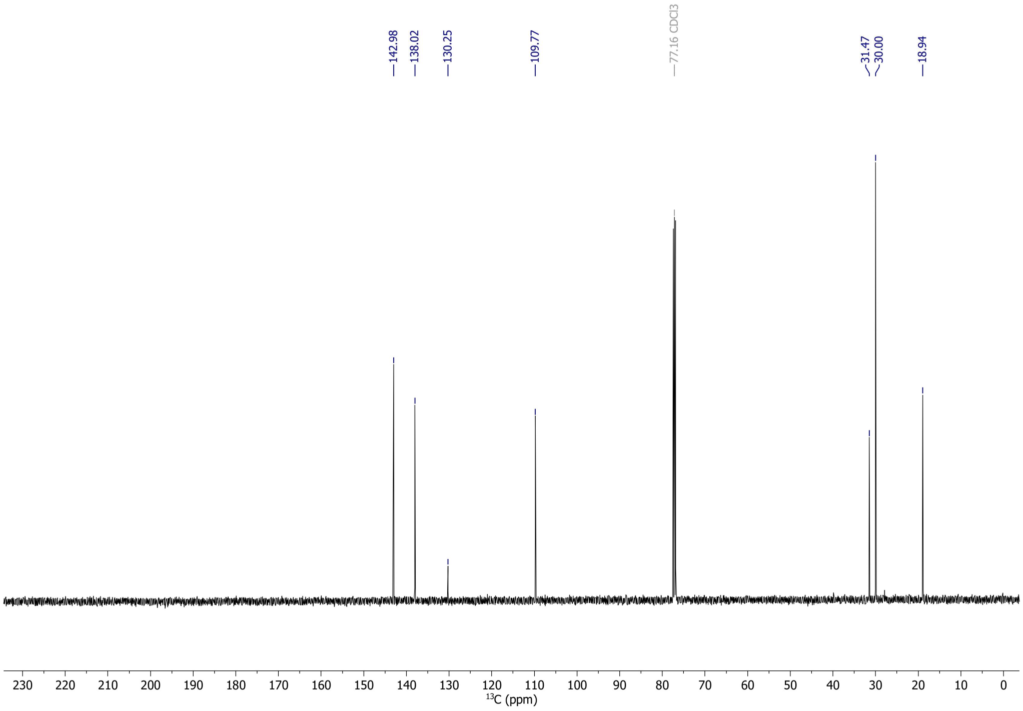

3-(3-Methoxypropyl)furan, 1k

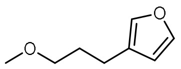

^{1}H NMR (501 MHz, $CDCl_3$)

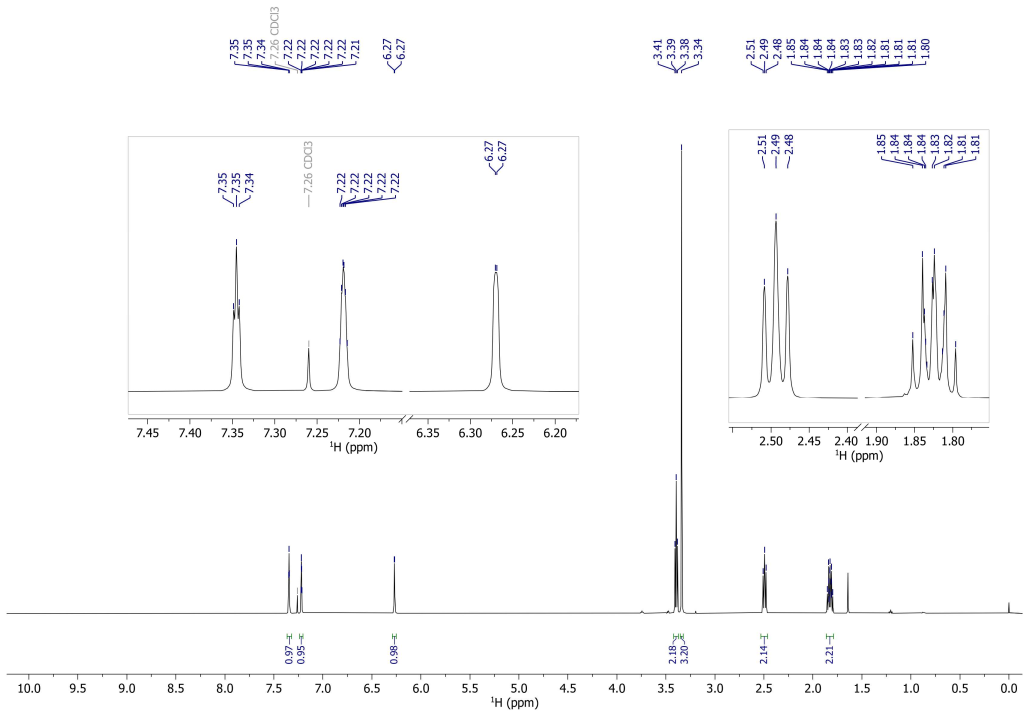

^{13}C NMR (126 MHz, $CDCl_3$)

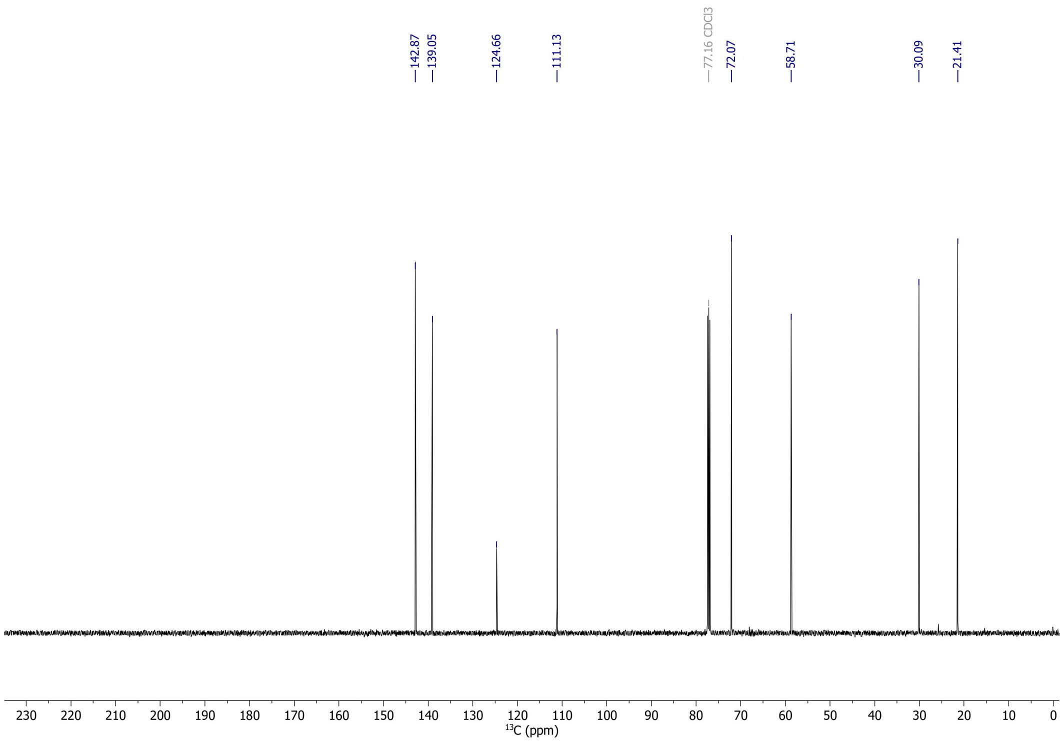

3-(But-3-en-1-yl)furan, 1n

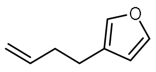

^{1}H NMR (501 MHz, $CDCl_3$)

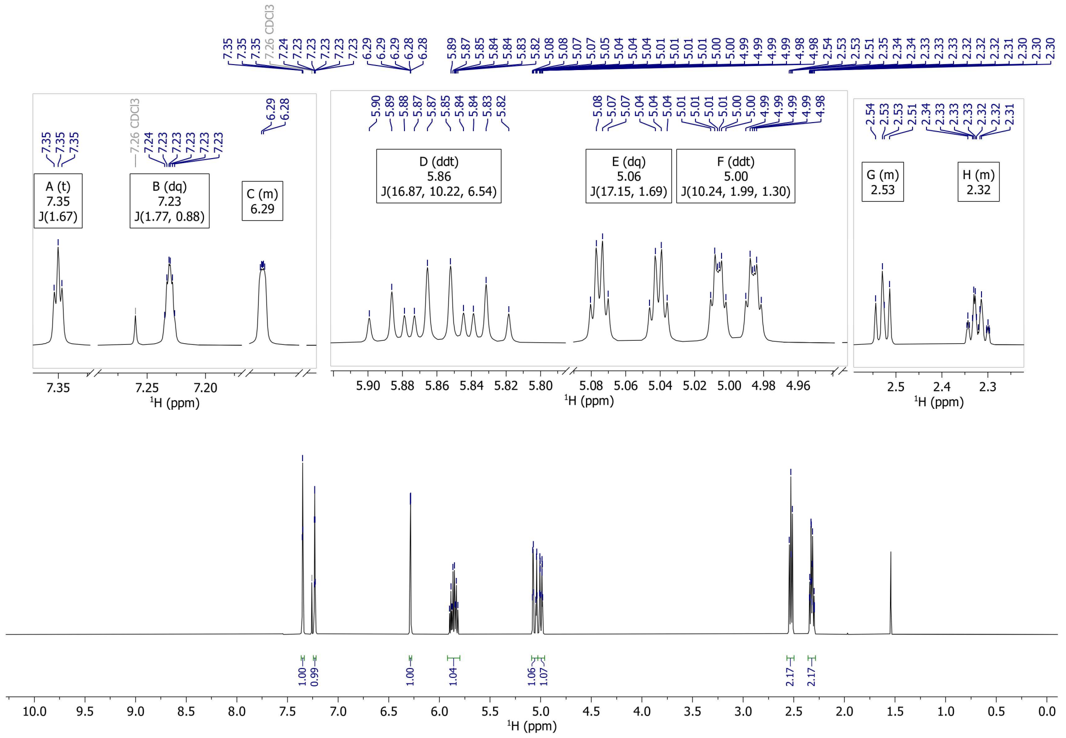

^{13}C NMR (126 MHz, $CDCl_3$)

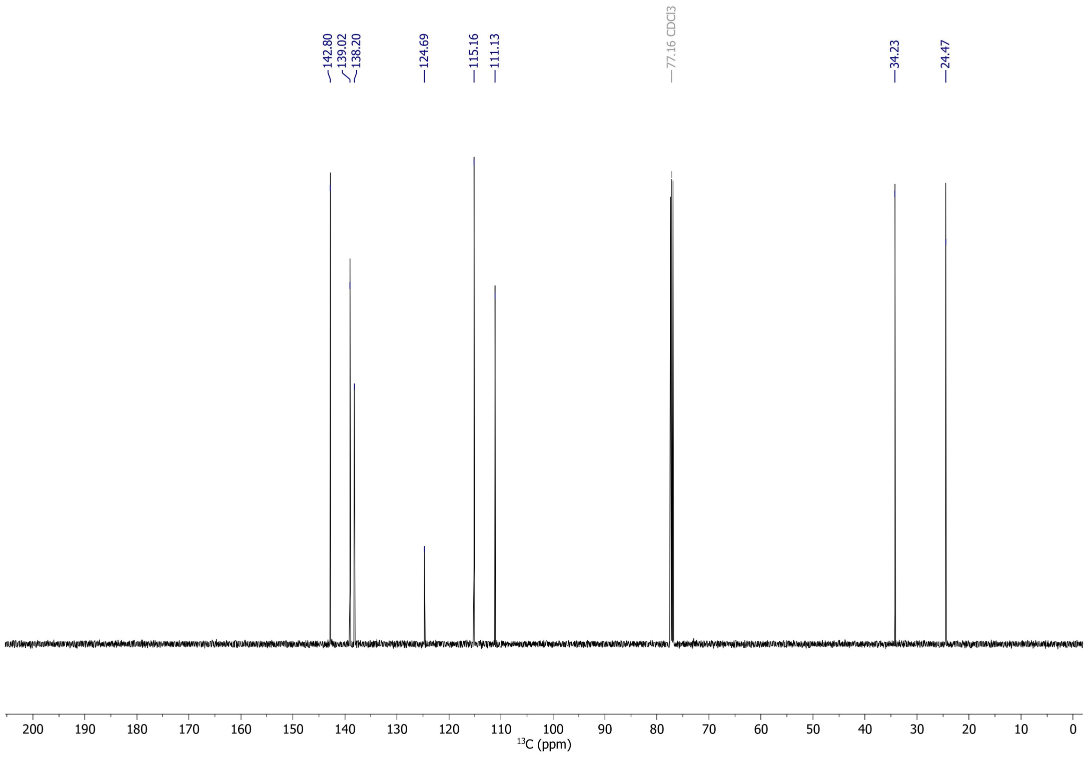

3-(Chloromethyl)furan, 1m

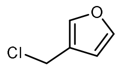

^{1}**H NMR** (501 MHz, $CDCl_3$)

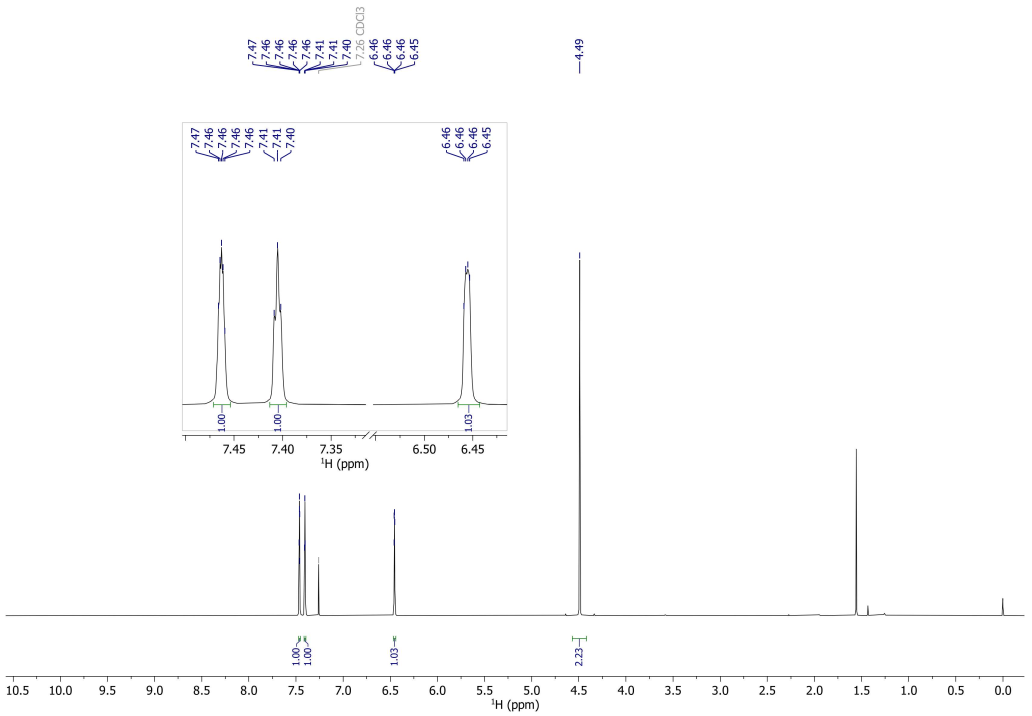

^{13}**C NMR** (126 MHz, $CDCl_3$)

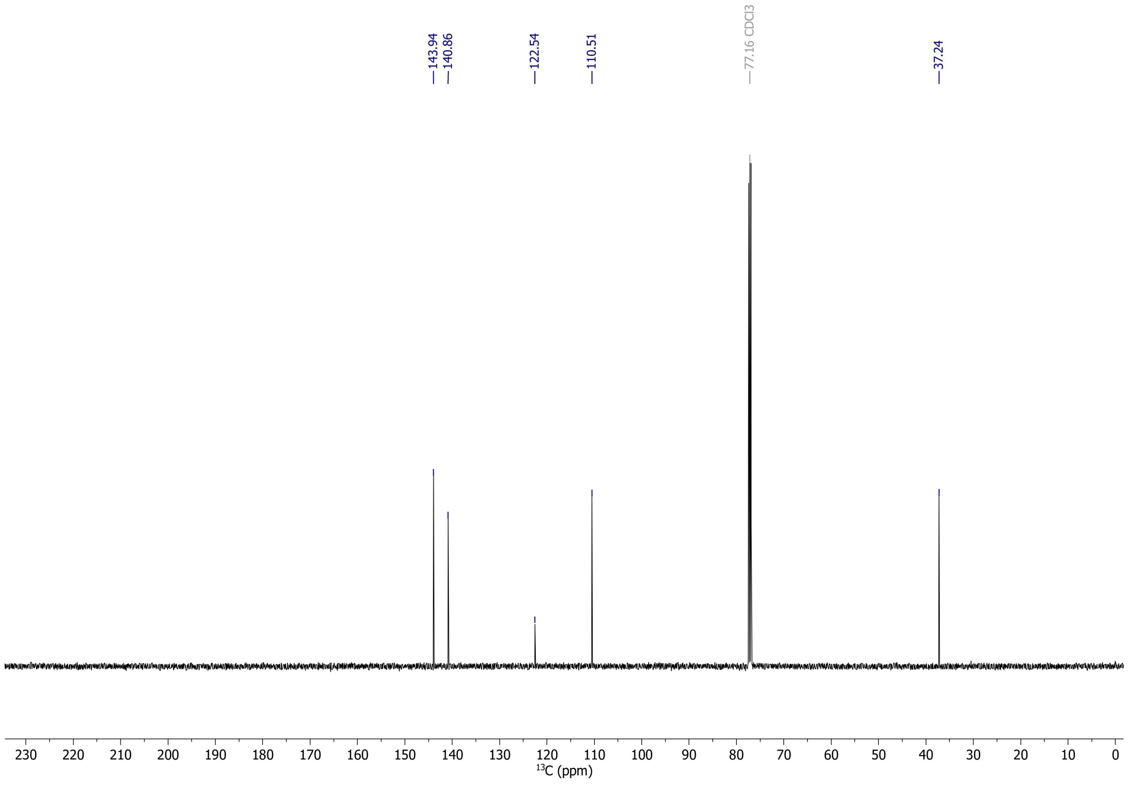

***Perillene*, 1o**

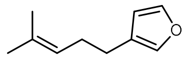

^{1}H NMR (600 MHz, $CDCl_3$)

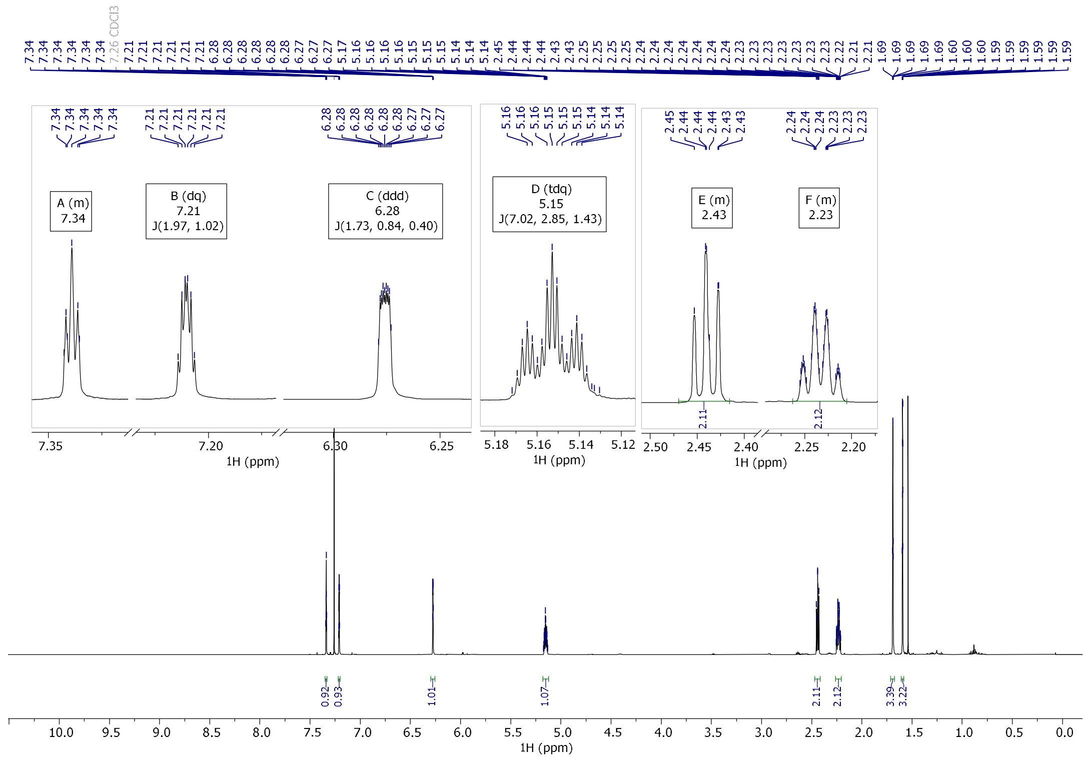

^{13}C NMR (151 MHz, $CDCl_3$)

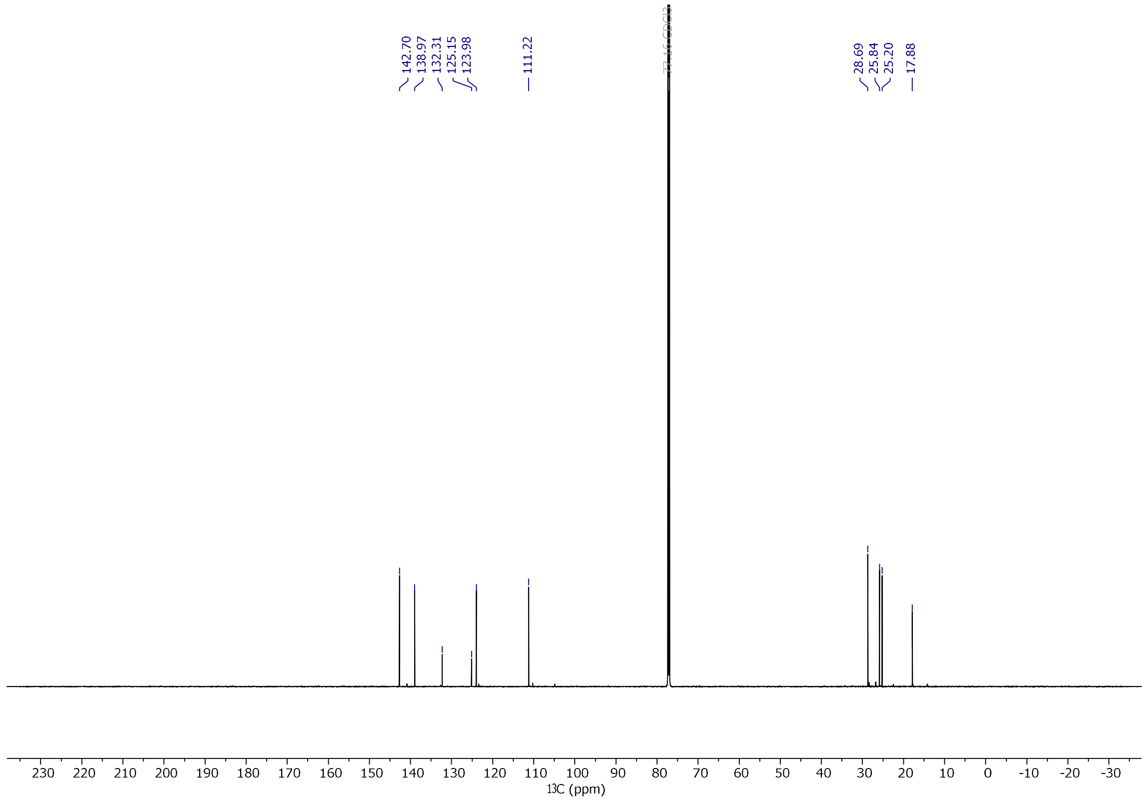

Methyl 2-(4-methylfuran-2-yl)acetate, 1q

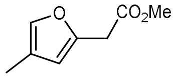

¹H NMR (501 MHz, $CDCl_3$)

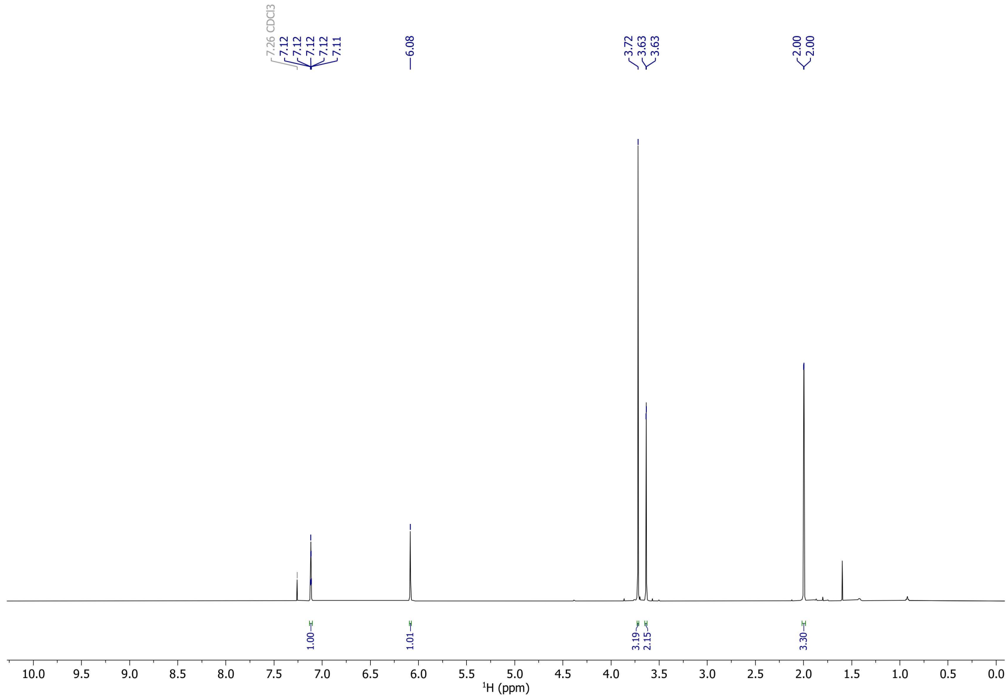

¹³C NMR (126 MHz, $CDCl_3$)

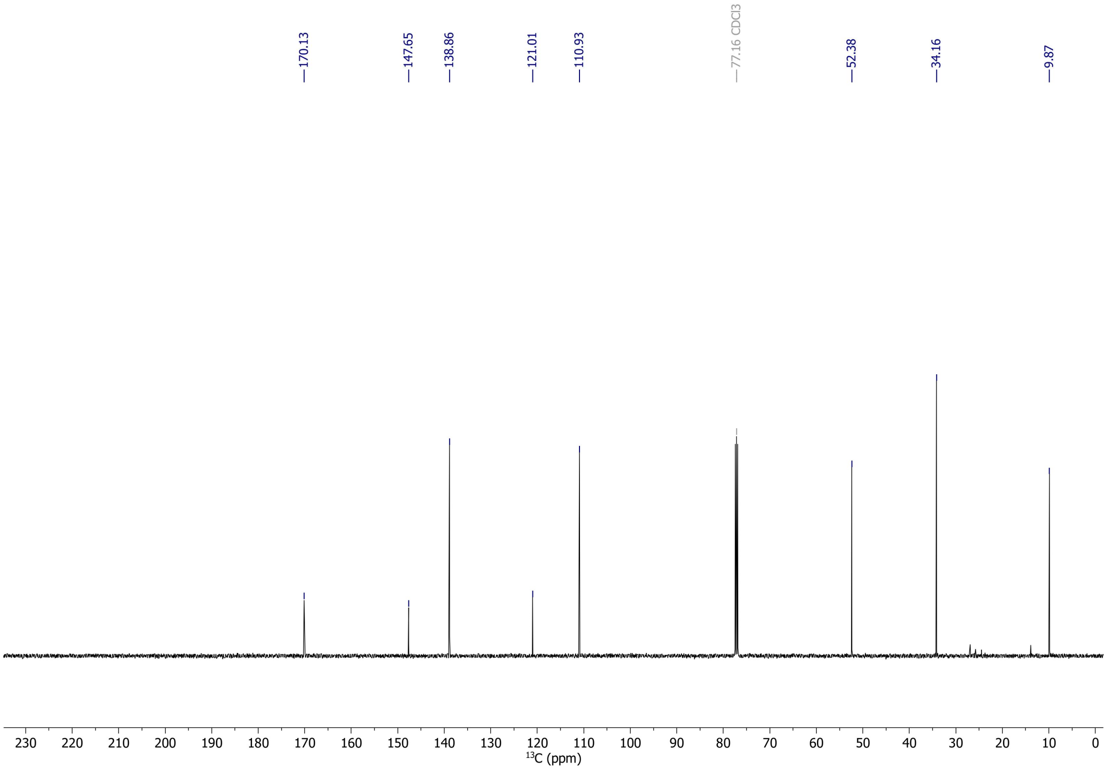

3-(4-(*tert*-Butyl)phenyl)-2-methylfuran, 3j

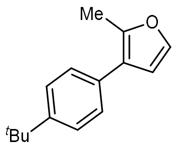

^{1}H NMR (501 MHz, $CDCl_3$)

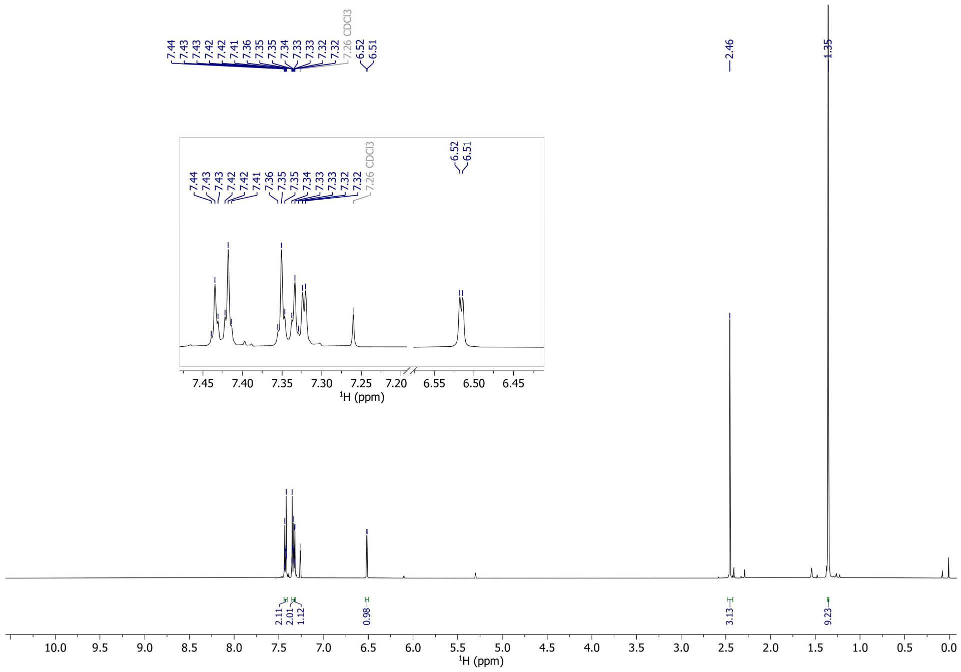

^{13}C NMR (126 MHz, $CDCl_3$)

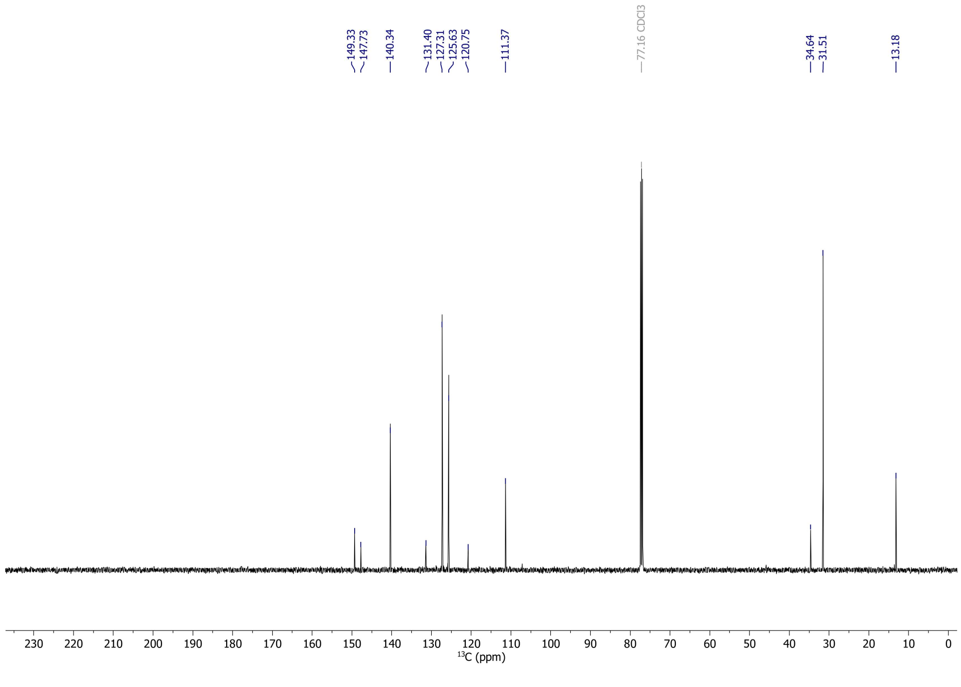

Ethyl 2-butylfuran-3-carboxylate, 3k

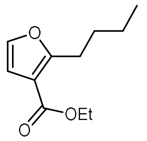

^{1}H NMR (501 MHz, $CDCl_3$)

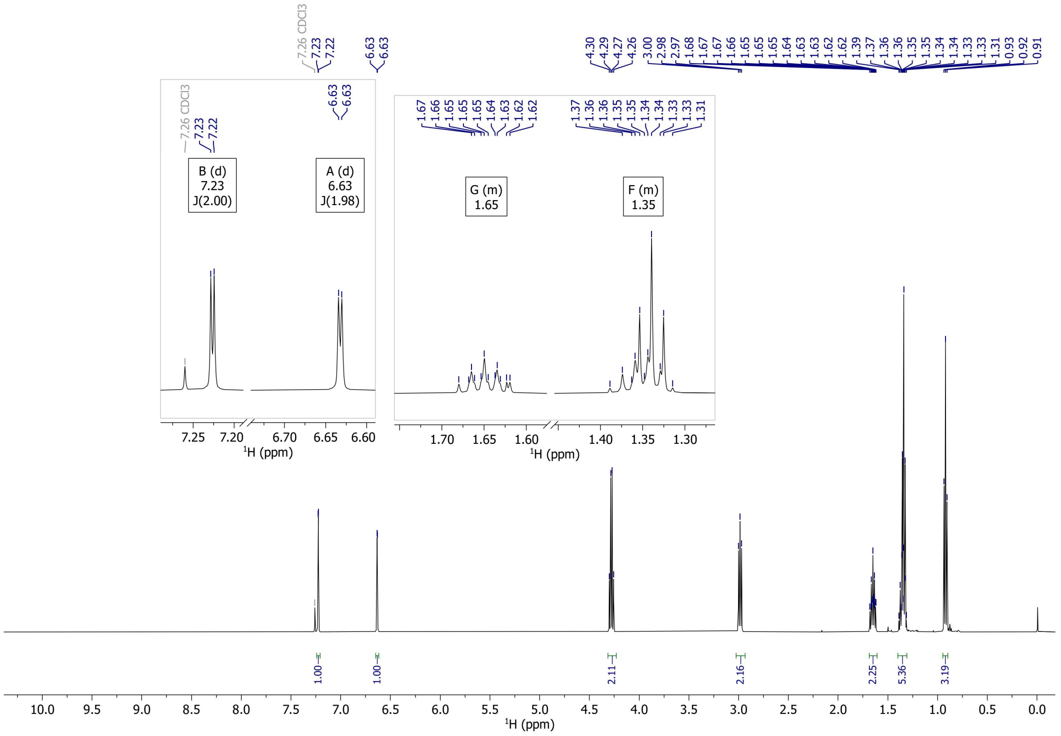

^{13}C NMR (126 MHz, $CDCl_3$)

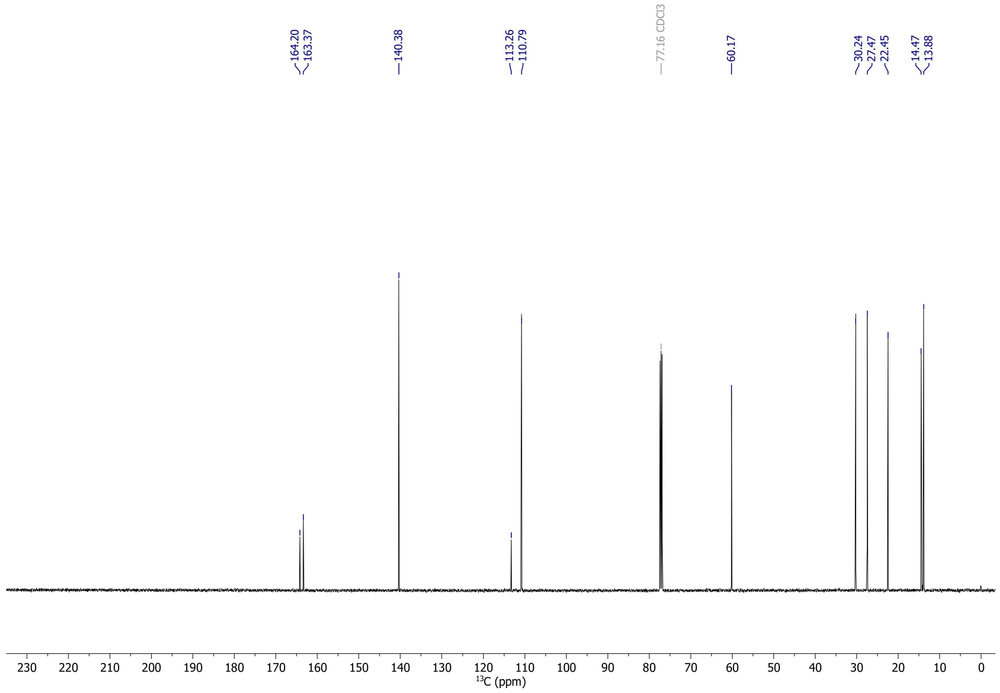

4-(Furan-2-yl)benzonitrile, 3n

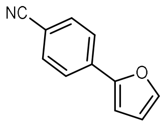

^{1}H NMR (501 MHz, $CDCl_3$)

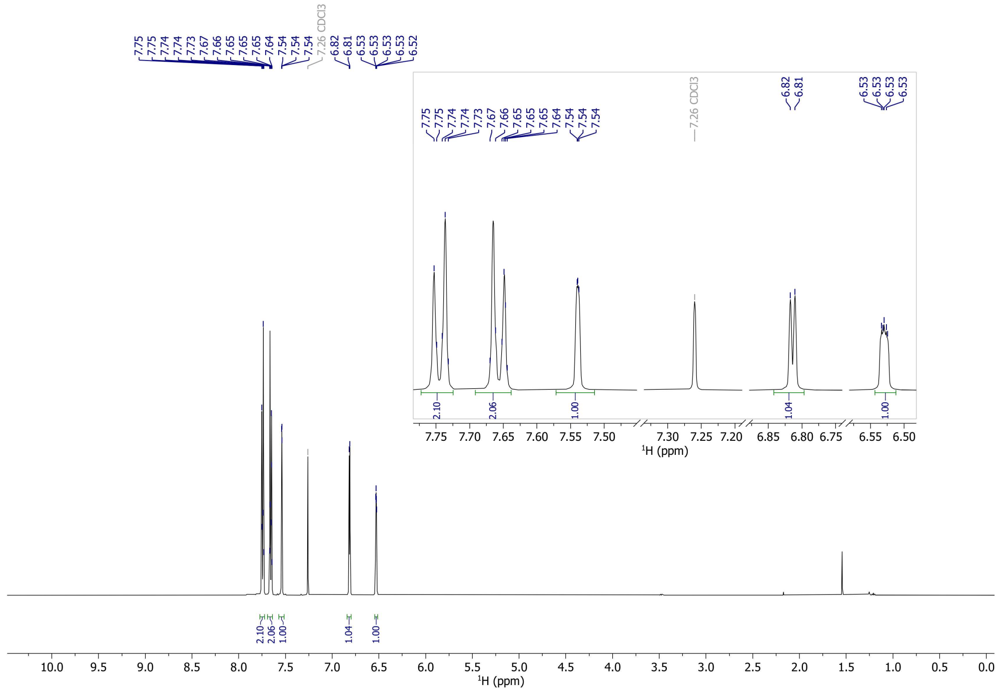

^{13}C NMR (126 MHz, $CDCl_3$)

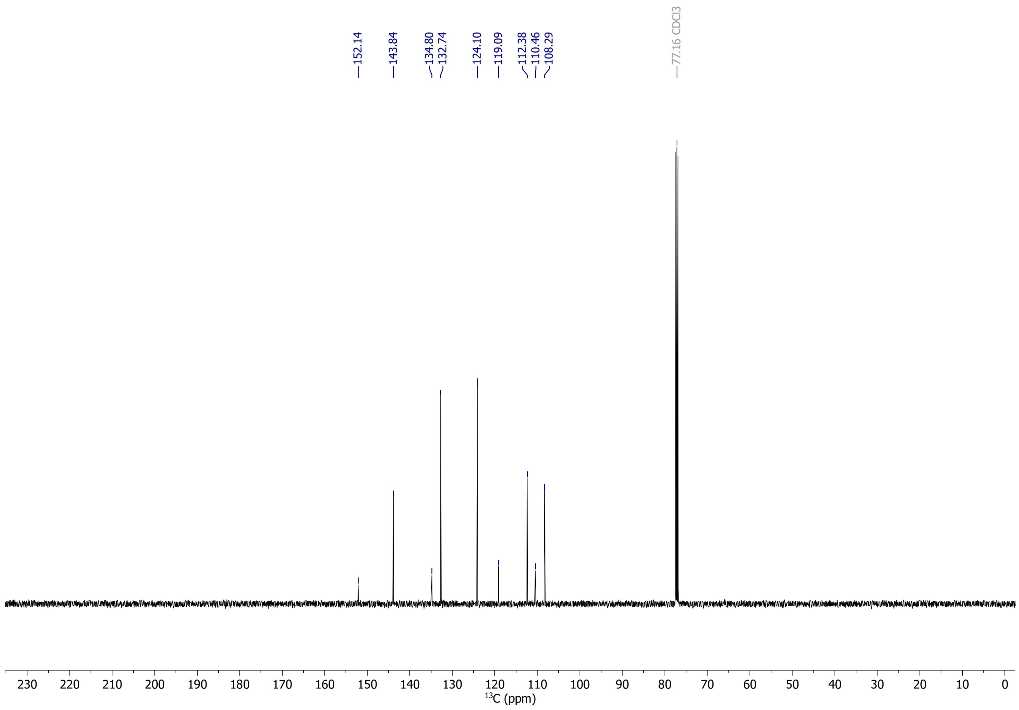

2-(4-Fluorophenyl)furan, 3o

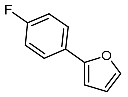

^{1}H NMR (501 MHz, $CDCl_3$)

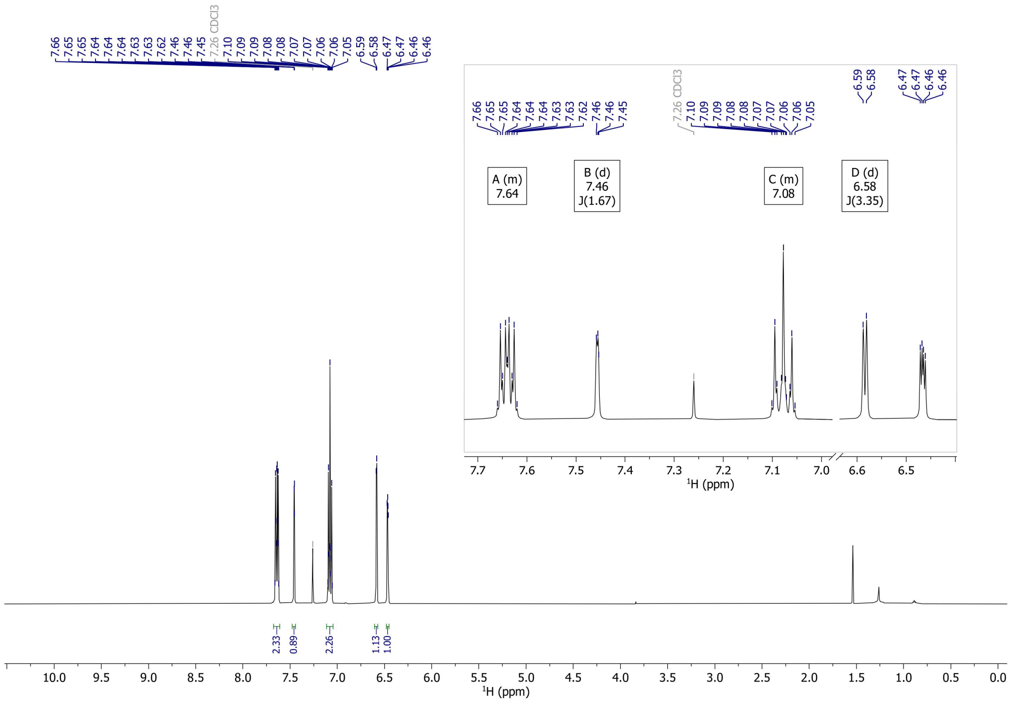

^{13}C NMR (126 MHz, $CDCl_3$)

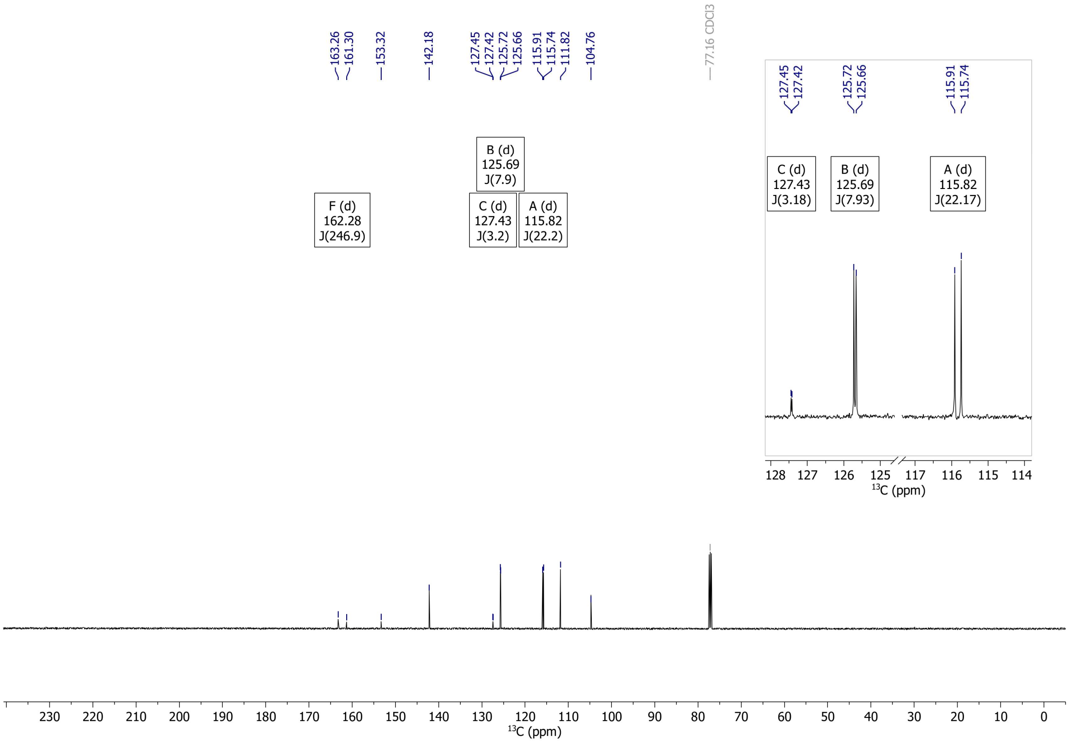

^{19}F NMR (471 MHz, $CDCl_3$)

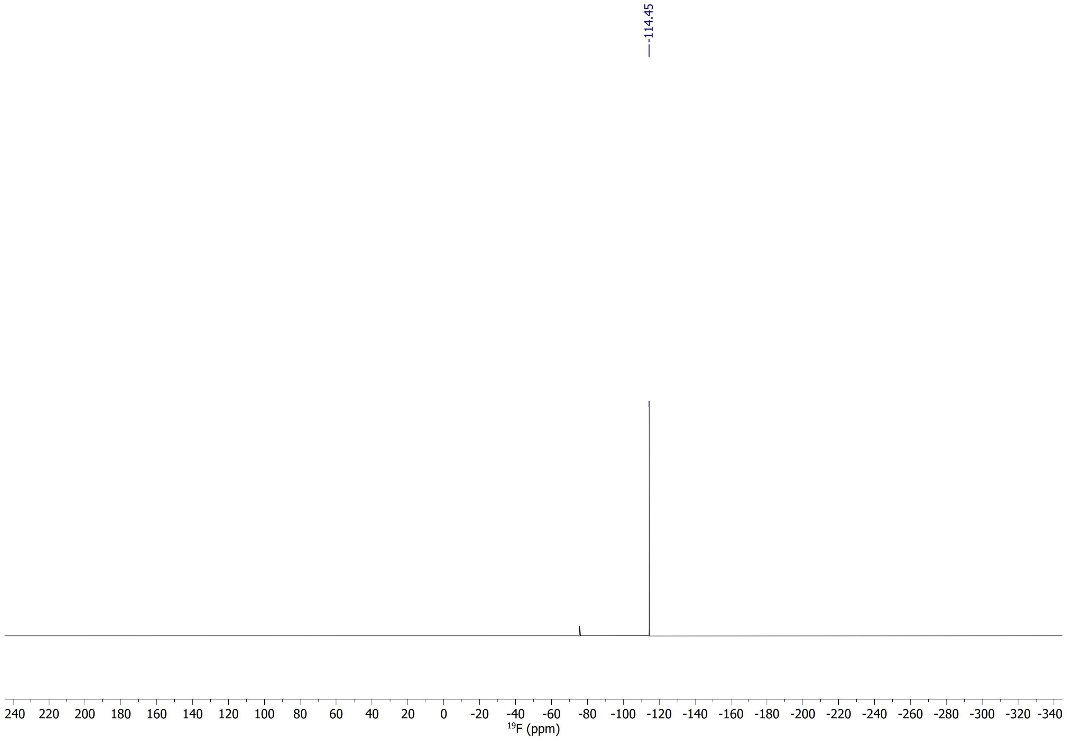

2-(3-(Trifluoromethyl)phenyl)furan, 3p

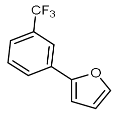

^{1}H NMR (501 MHz, $CDCl_3$)

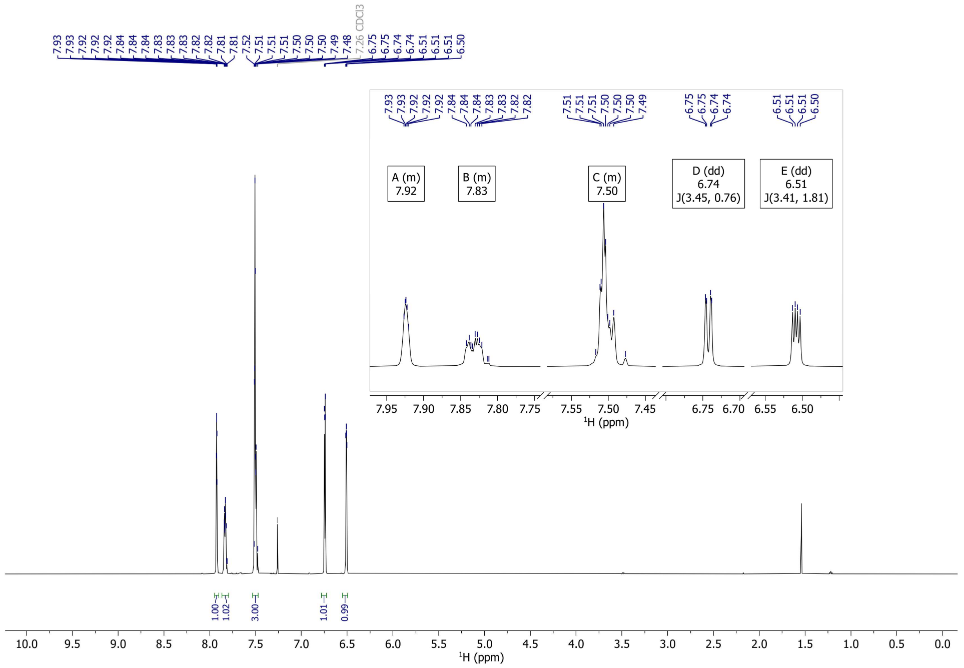

^{13}C NMR (126 MHz, $CDCl_3$)

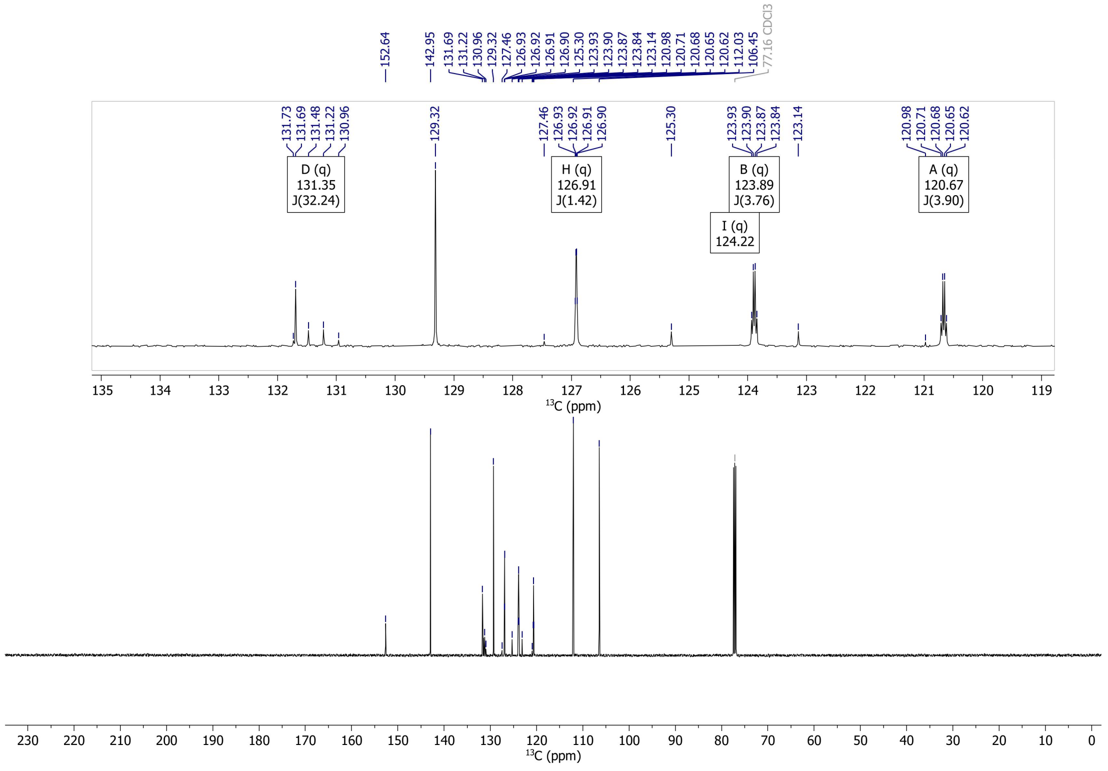

^{19}F NMR (471 MHz, $CDCl_3$)

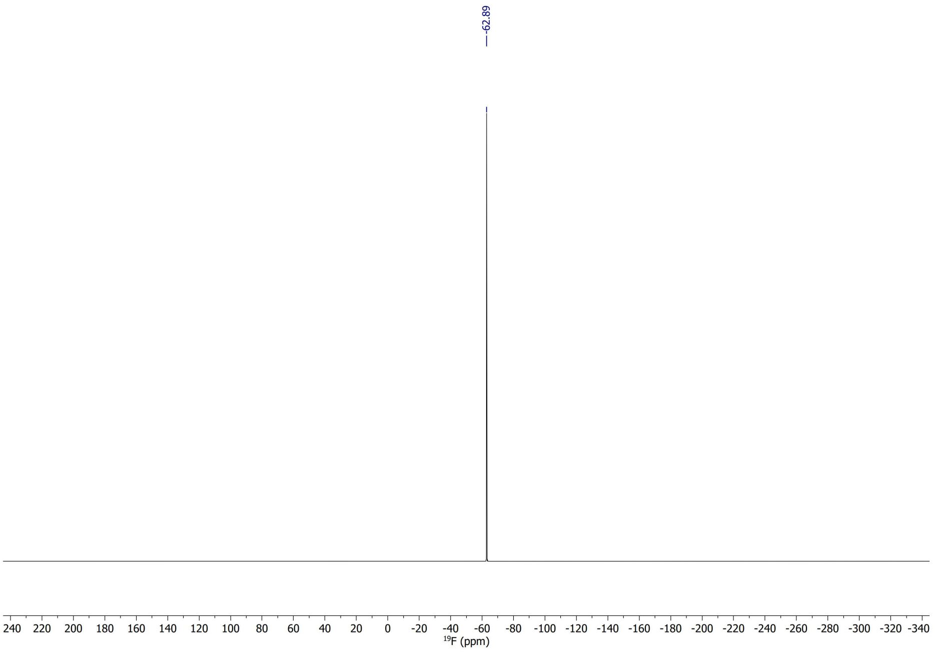

(*Z*)-3-Iodo-2-methylprop-2-en-1-ol, S1

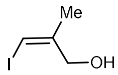

^{1}H NMR (501 MHz, $CDCl_3$)

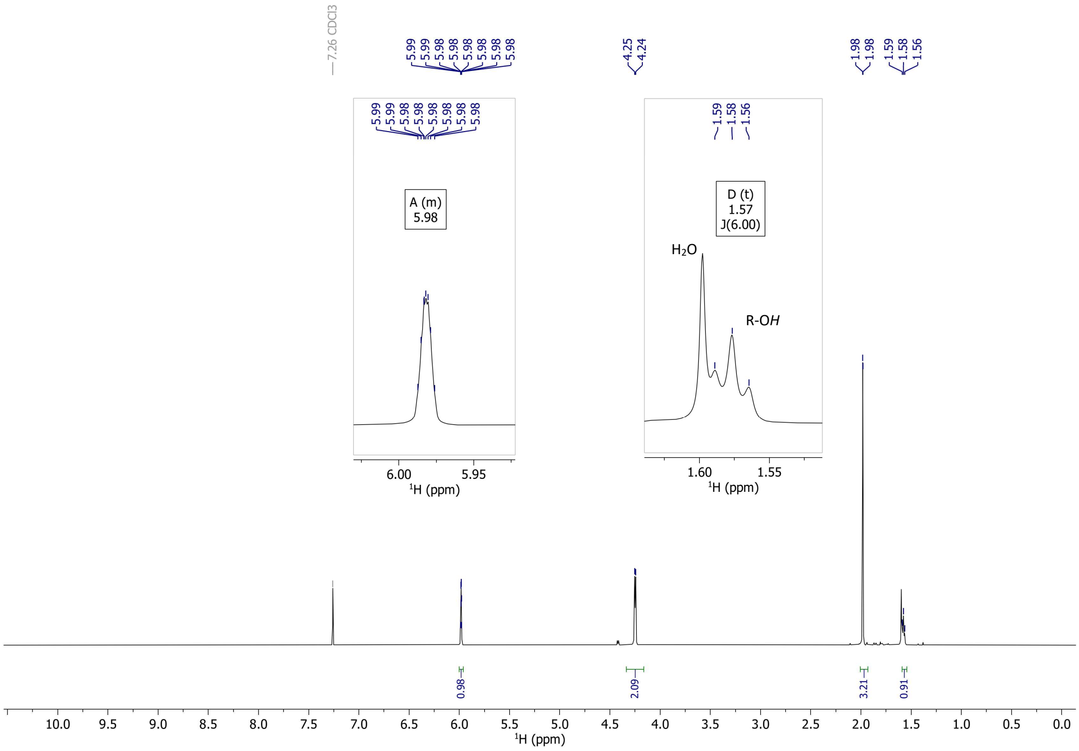

^{13}C NMR (126 MHz, $CDCl_3$)

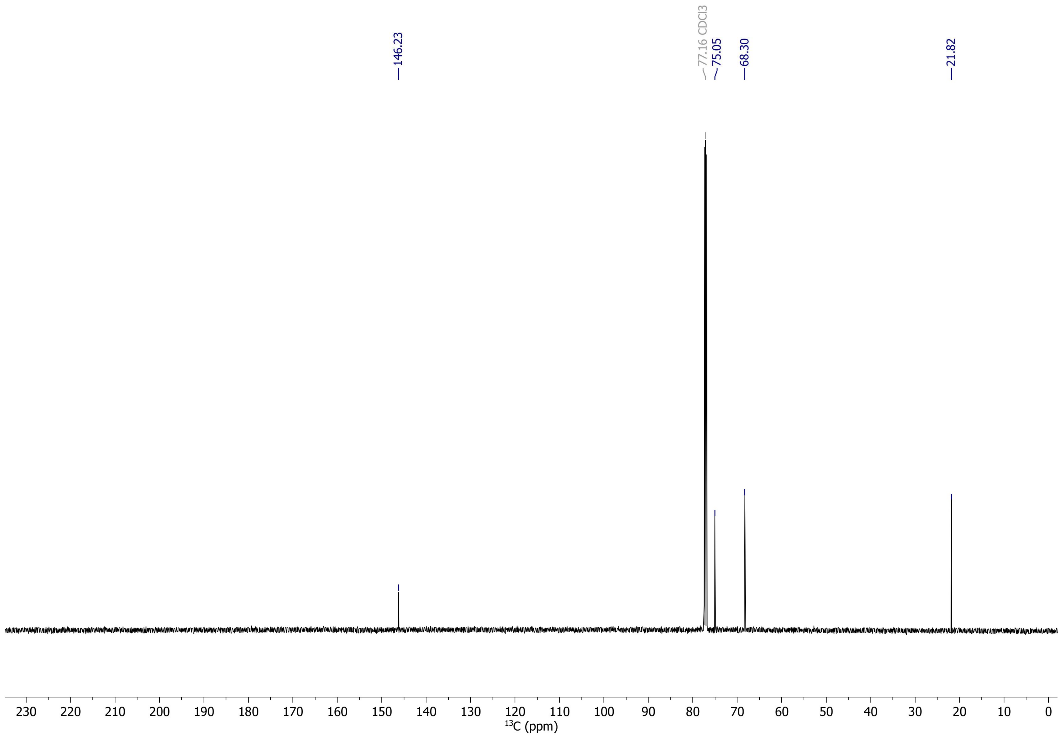

3-(Allyloxy)oct-1-ene, S2

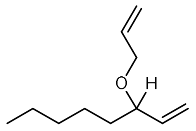

^{1}H NMR (501 MHz, $CDCl_3$)

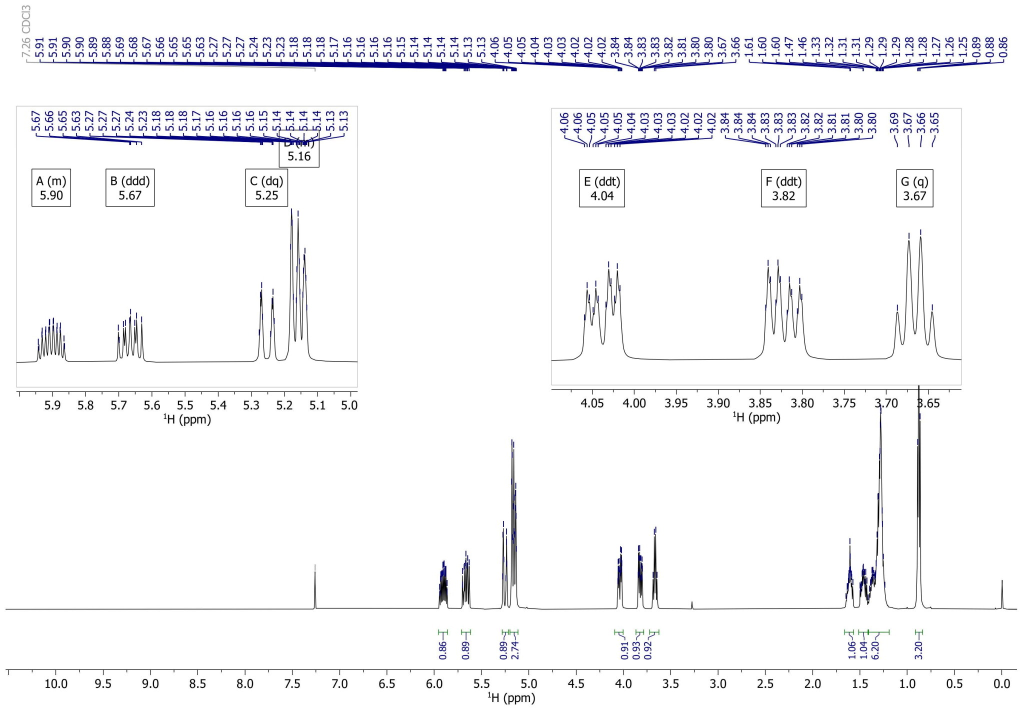

^{13}C NMR (126 MHz, $CDCl_3$)

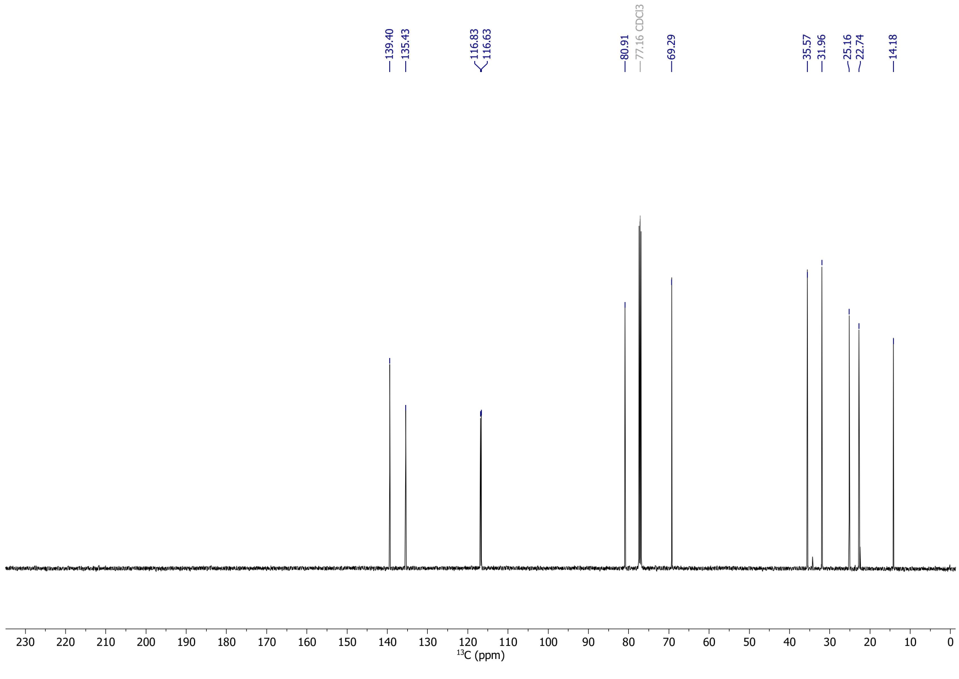

5-Hexyl-2,3-dihydrofuran, S4

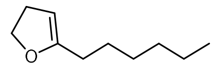

^{1}H NMR (501 MHz, CD_2Cl_2)

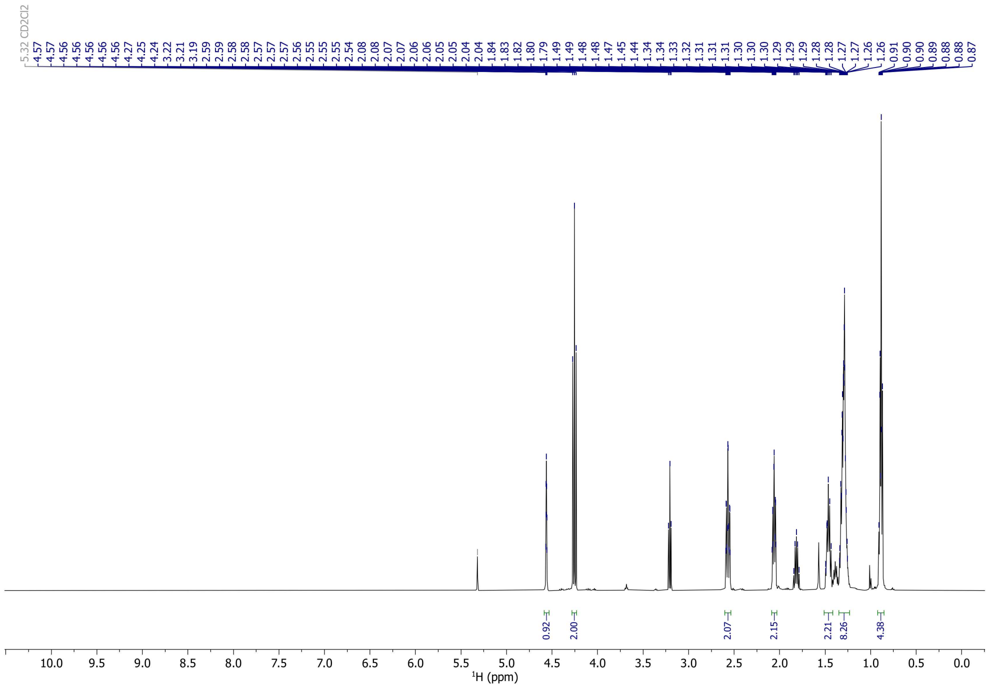

^{13}C NMR (126 MHz, CD_2Cl_2)

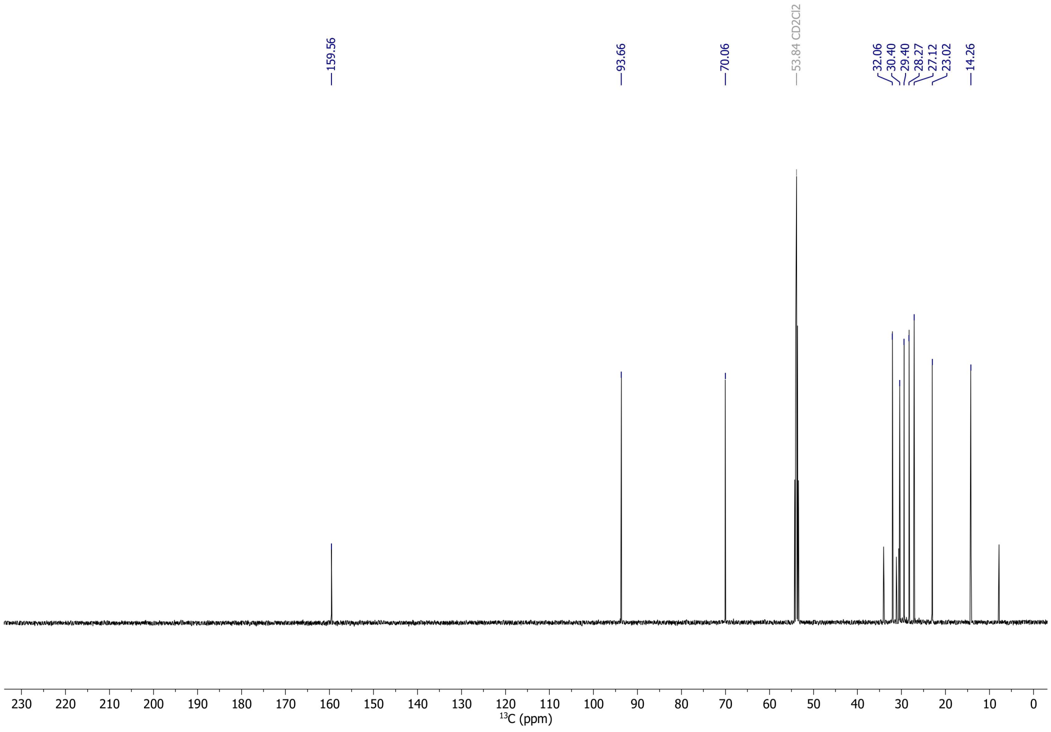

3-(4-Methoxyphenyl)-2,5-dihydrofuran, 2a

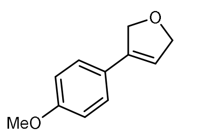

^{1}H NMR (501 MHz, $CDCl_3$)

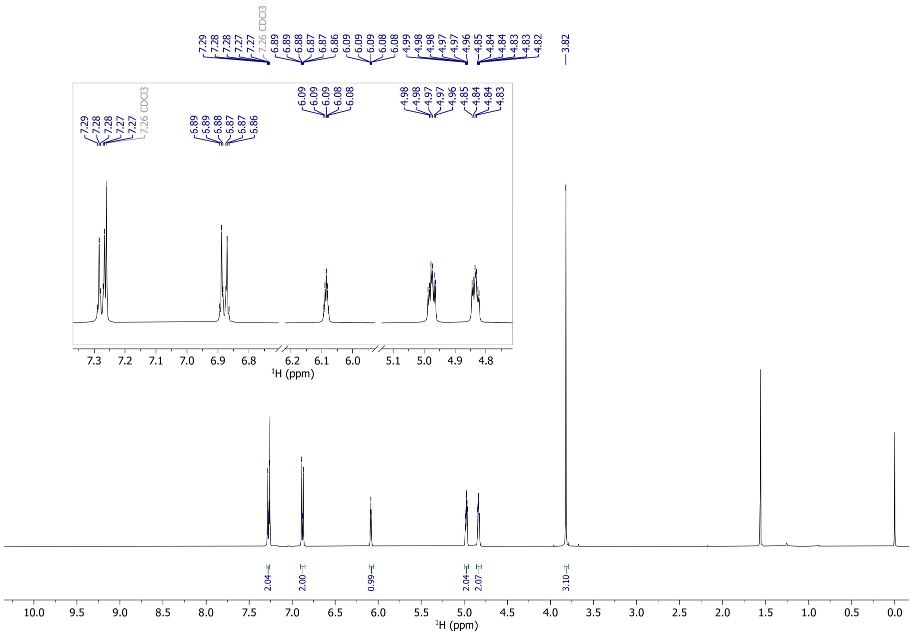

^{13}C NMR (126 MHz, $CDCl_3$)

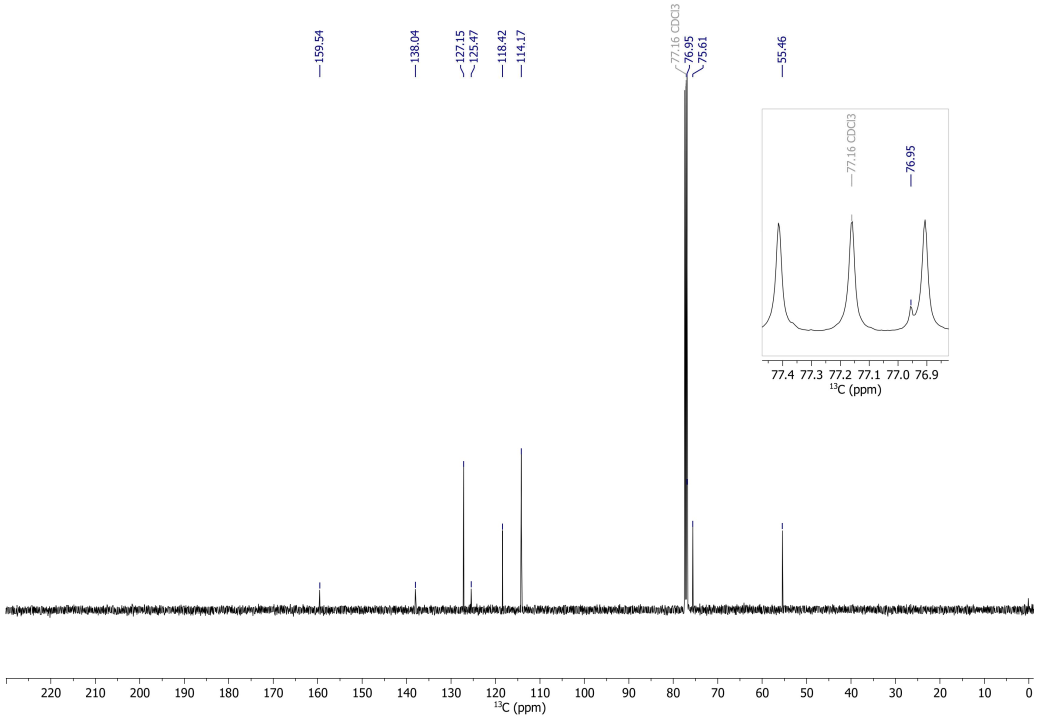

3-(4-Methoxyphenyl)tetrahydrofuran, 4a

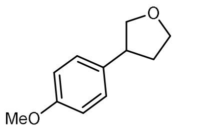

^{1}H NMR (501 MHz, $CDCl_3$)

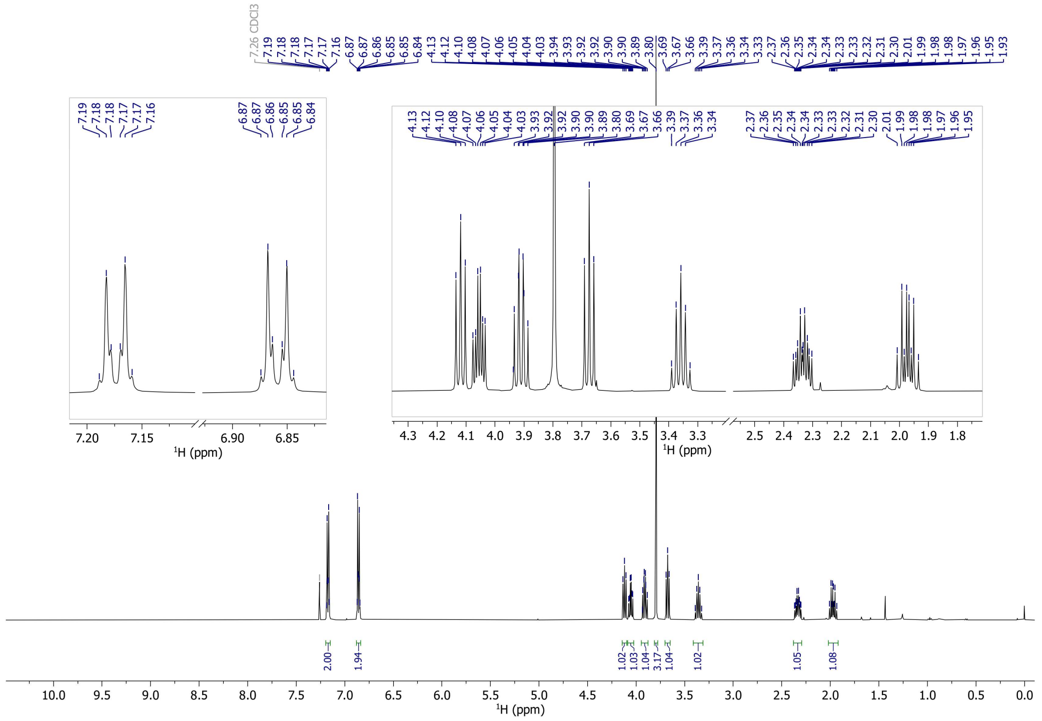

^{13}C NMR (126 MHz, $CDCl_3$)

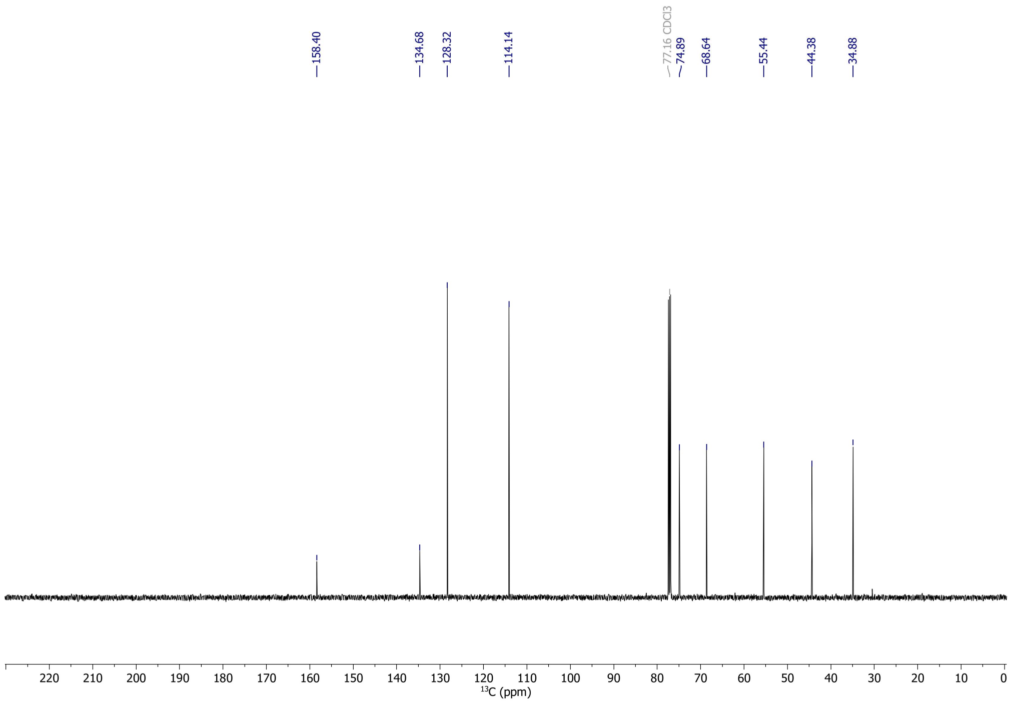

3-(4-(*Tert*-butyl)phenyl)-2,5-dihydrofuran, 2b

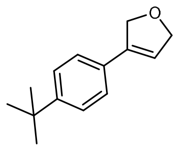

^{1}H NMR (501 MHz, CDCl$_3$)

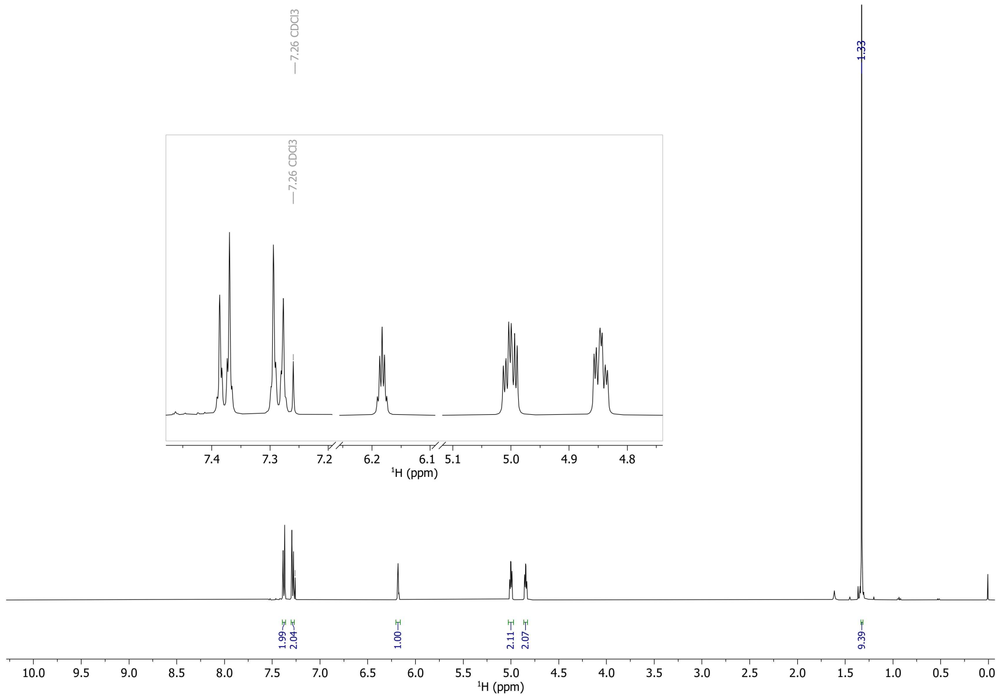

^{13}C NMR (126 MHz, CDCl$_3$)

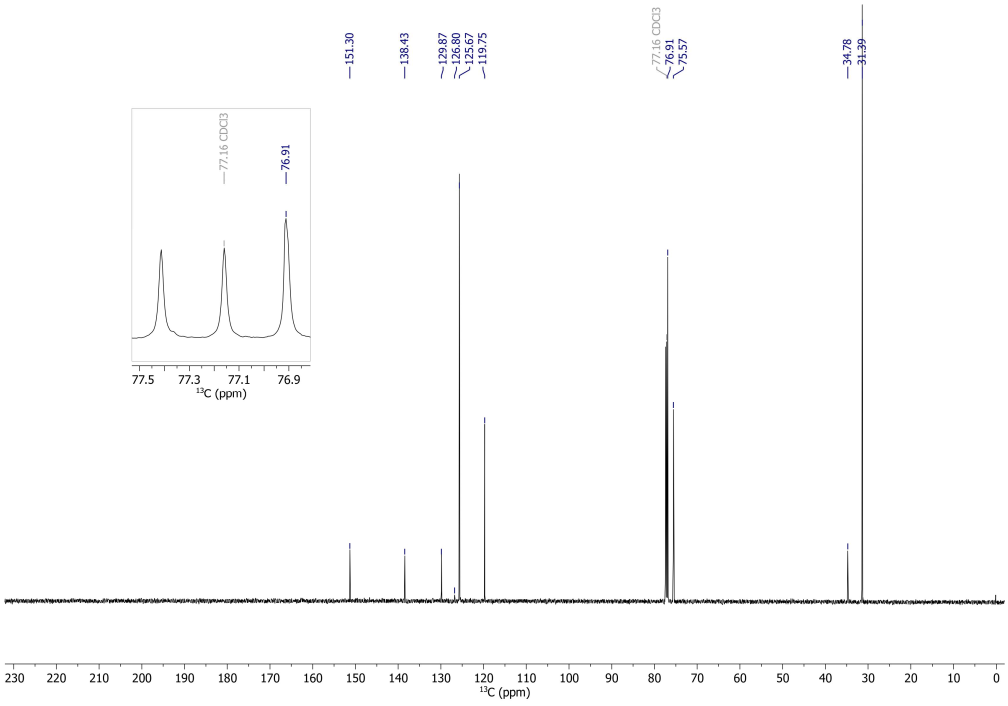

3-(4-(*Tert*-butyl)phenyl)tetrahydrofuran, 4b

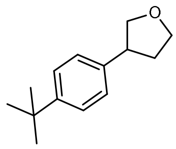

^{1}**H NMR** (501 MHz, $CDCl_3$)

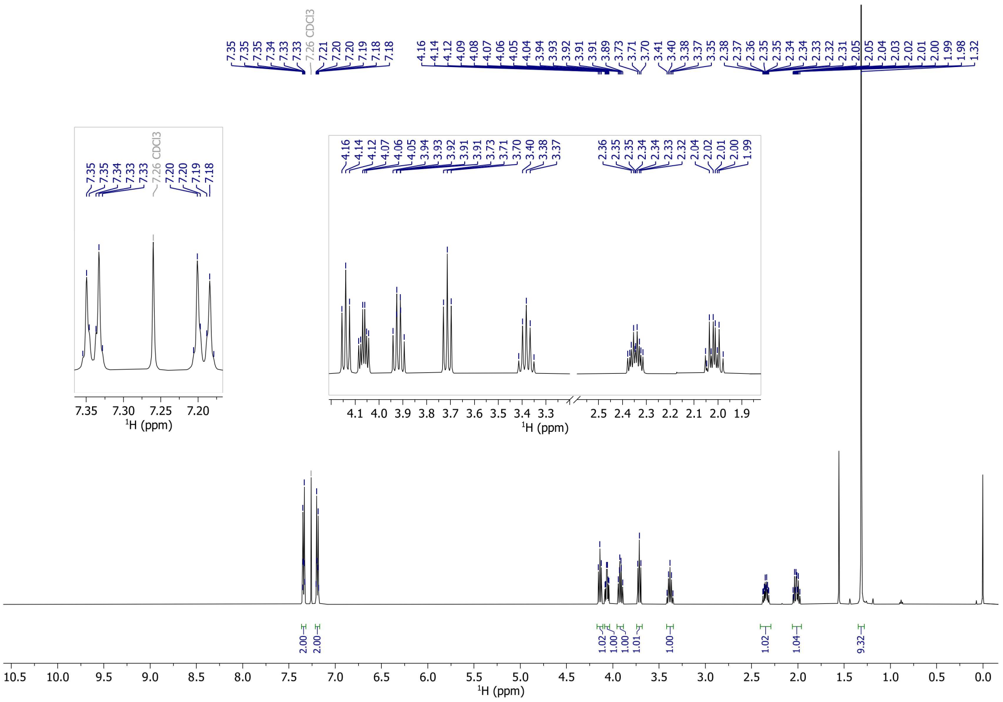

^{13}**C NMR** (126 MHz, $CDCl_3$)

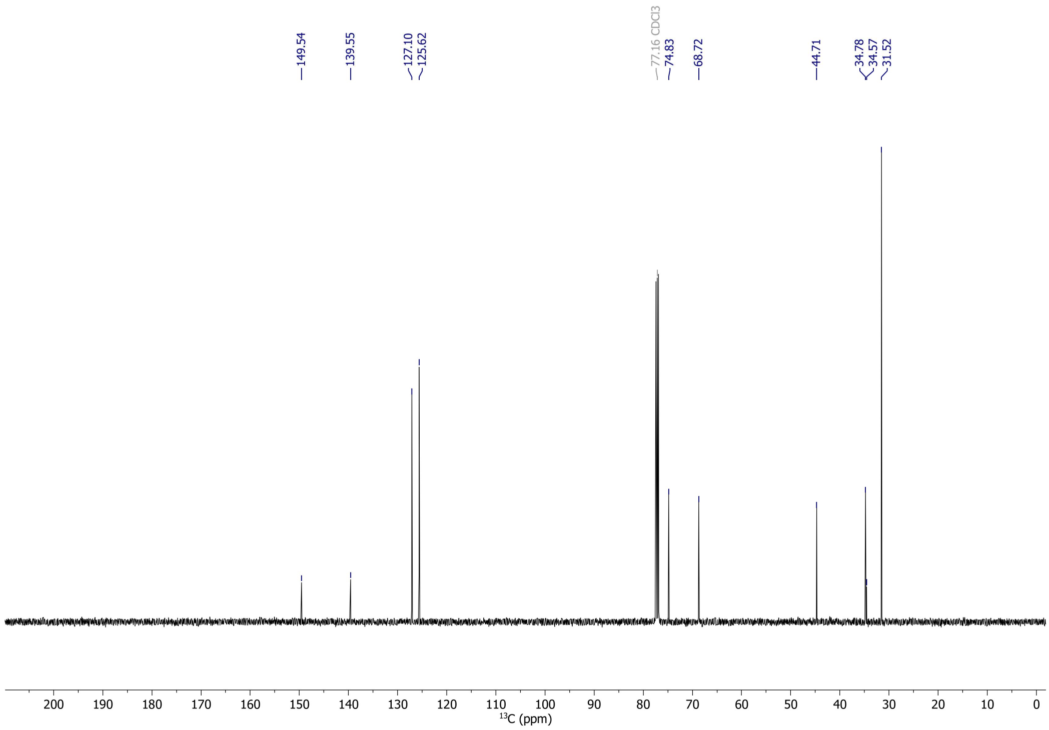

3-(*para*-Tolyl)-2,5-dihydrofuran, 2c

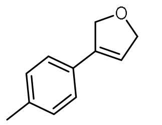

^{1}H NMR (501 MHz, $CDCl_3$)

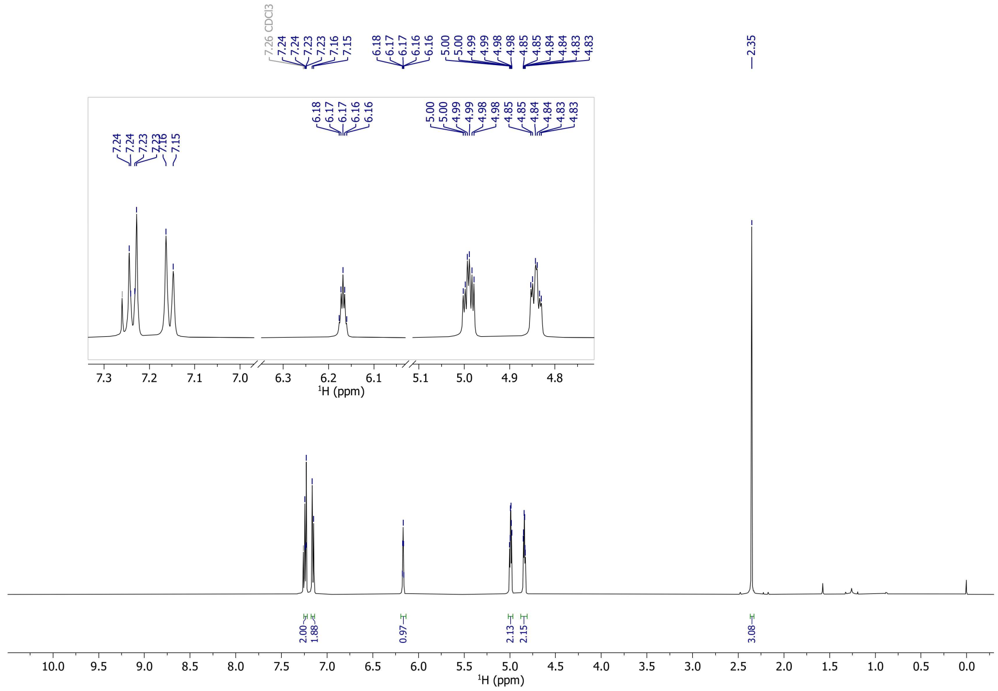

^{13}C NMR (126 MHz, $CDCl_3$)

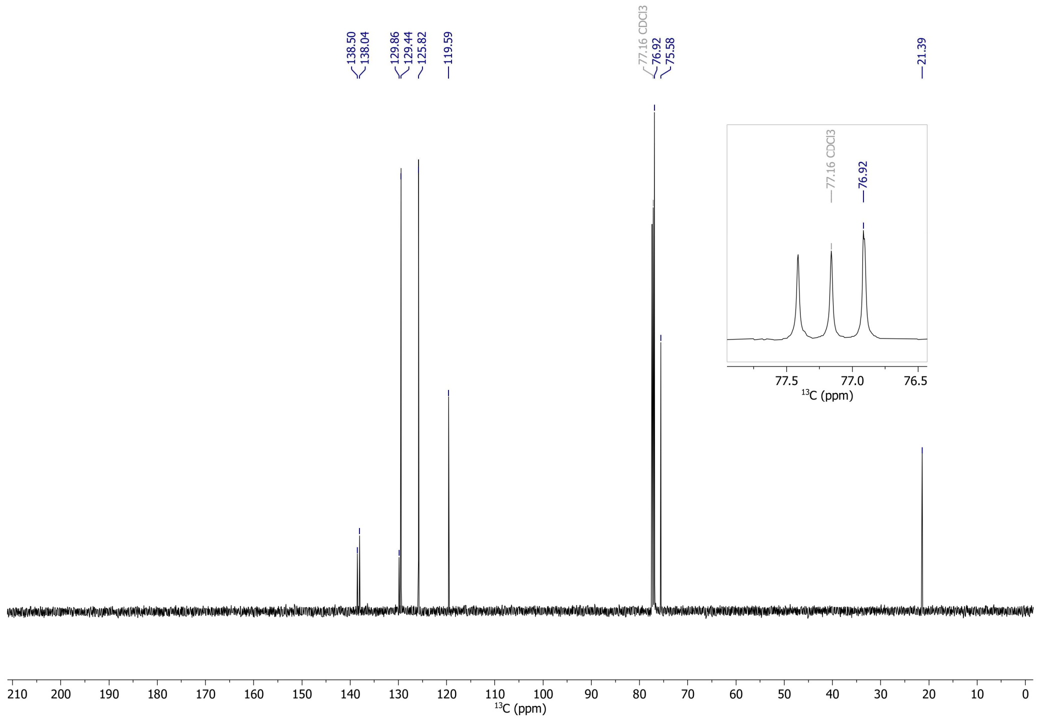

3-(*para*-Tolyl)tetrahydrofuran, 4c

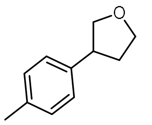

^{1}H NMR (501 MHz, $CDCl_3$)

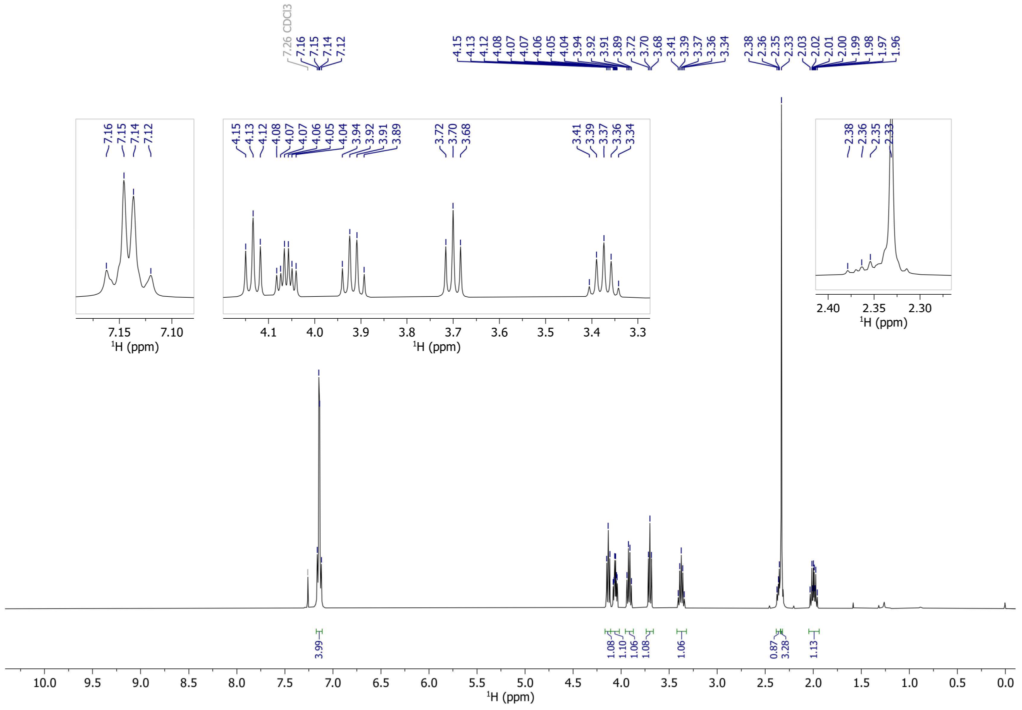

^{13}C NMR (126 MHz, $CDCl_3$)

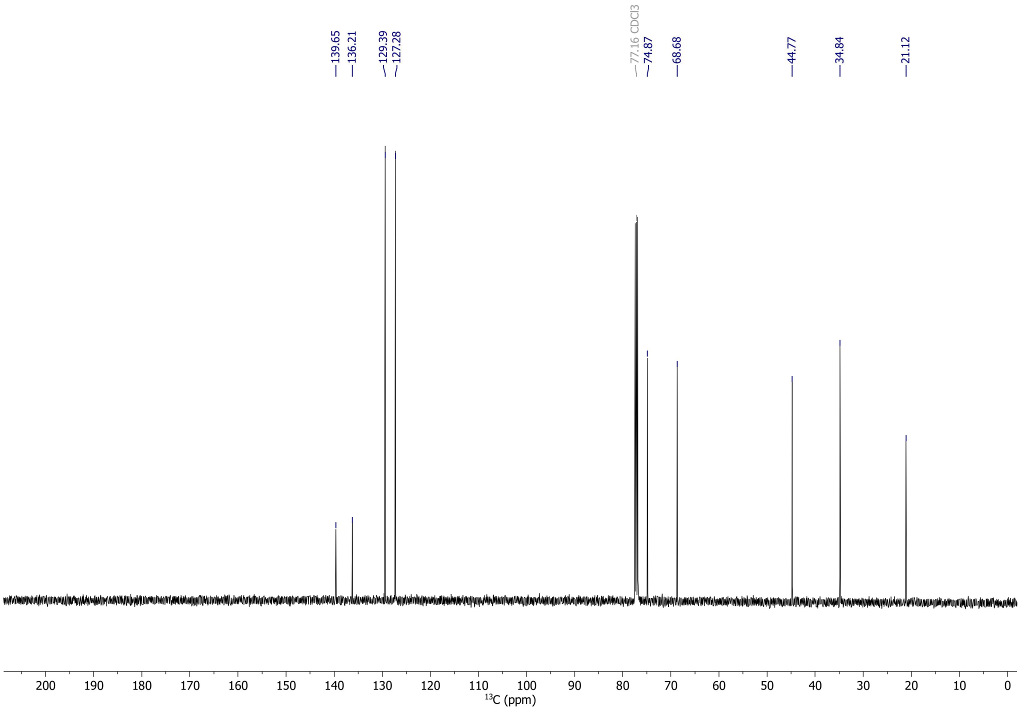

3-Phenyl-2,5-dihydrofuran, 2d

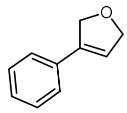

^{1}H NMR (501 MHz, $CDCl_3$)

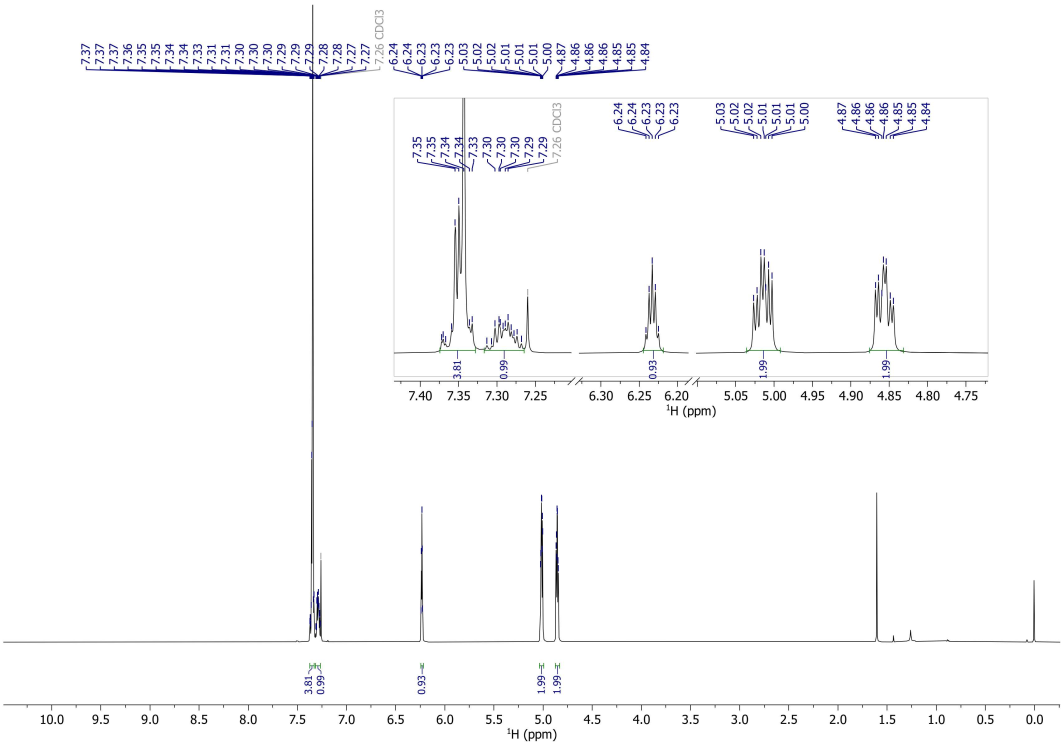

^{13}C NMR (126 MHz, $CDCl_3$)

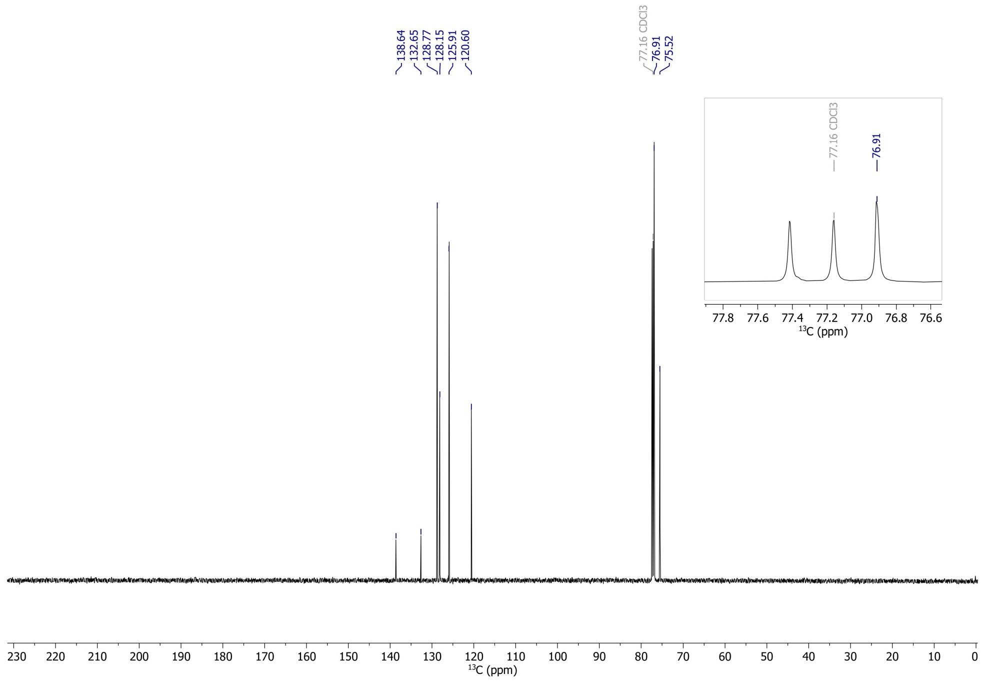

3-Phenyltetrahydrofuran, 4d

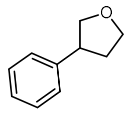

^{1}H NMR (501 MHz, CDCl$_3$)

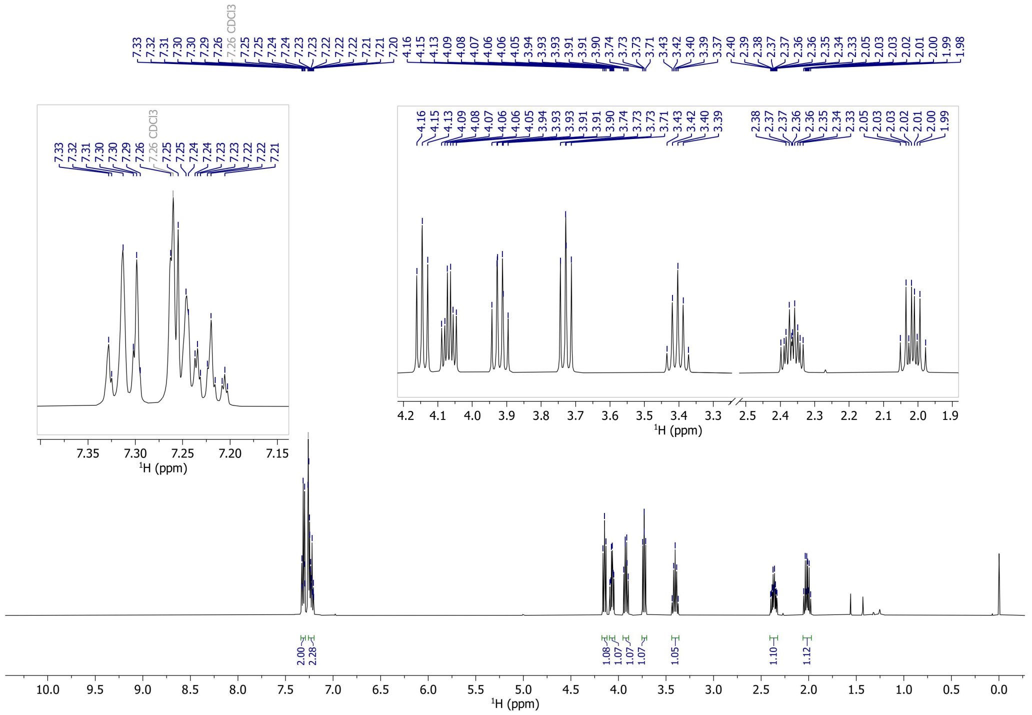

^{13}C NMR (126 MHz, CDCl$_3$)

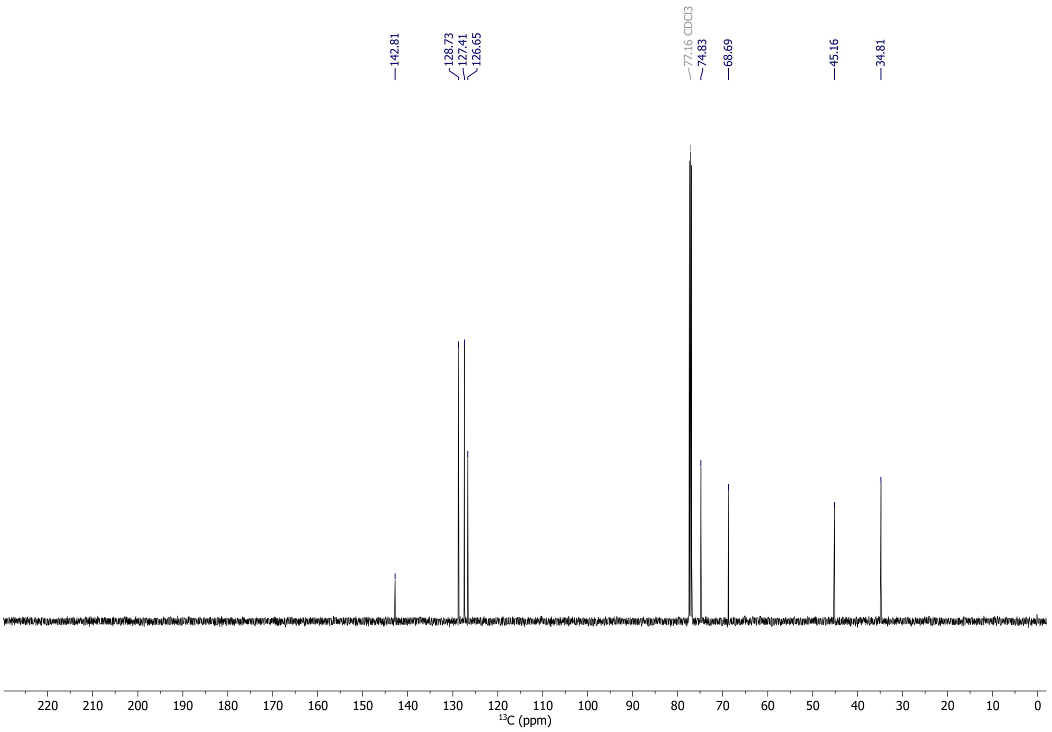

3-(4-Chlorophenyl)-2,5-dihydrofuran, 2e

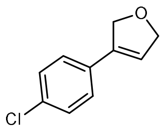

^{1}H NMR (501 MHz, $CDCl_3$)

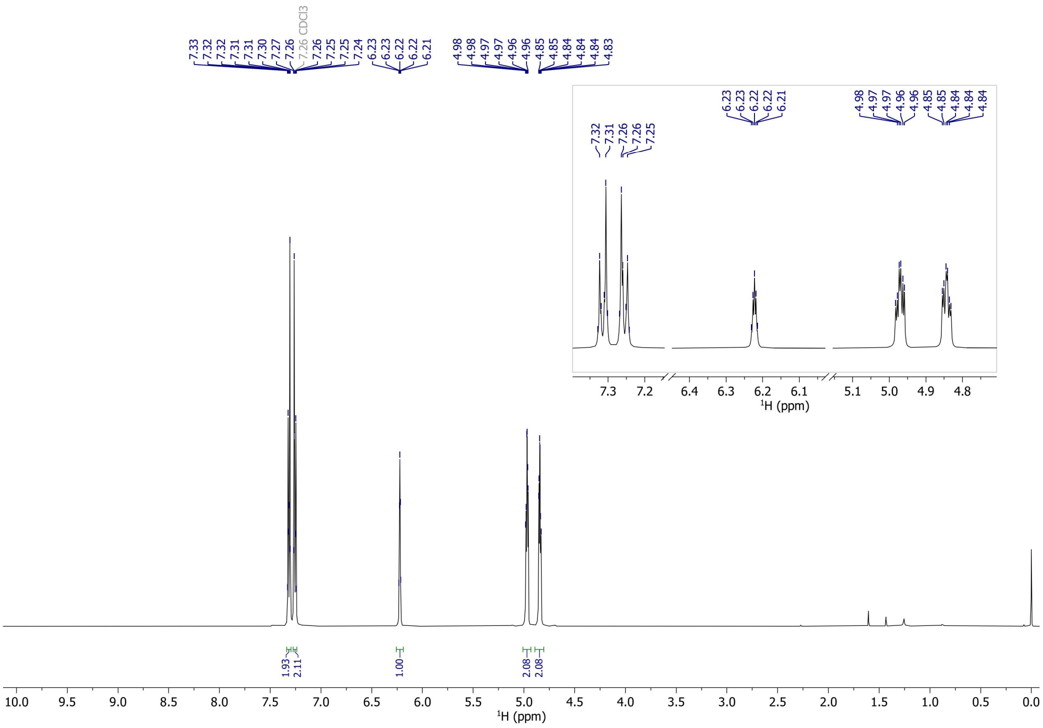

^{13}C NMR (126 MHz, $CDCl_3$)

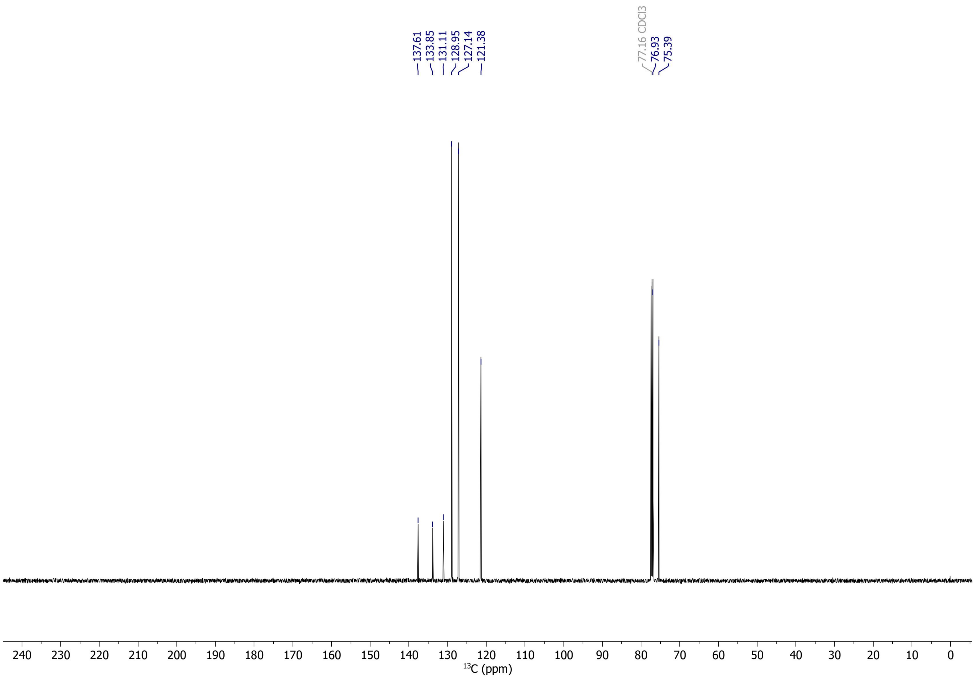

3-(4-Chlorophenyl)tetrahydrofuran, 4e

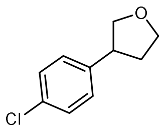

^{1}H NMR (501 MHz, $CDCl_3$)

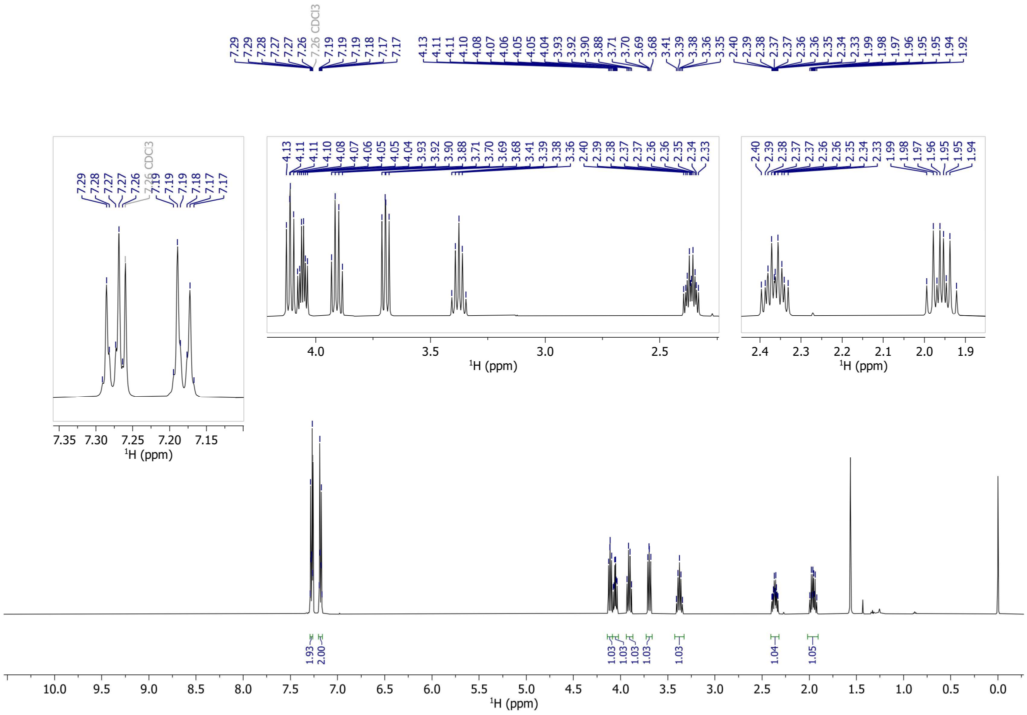

^{13}C NMR (126 MHz, $CDCl_3$)

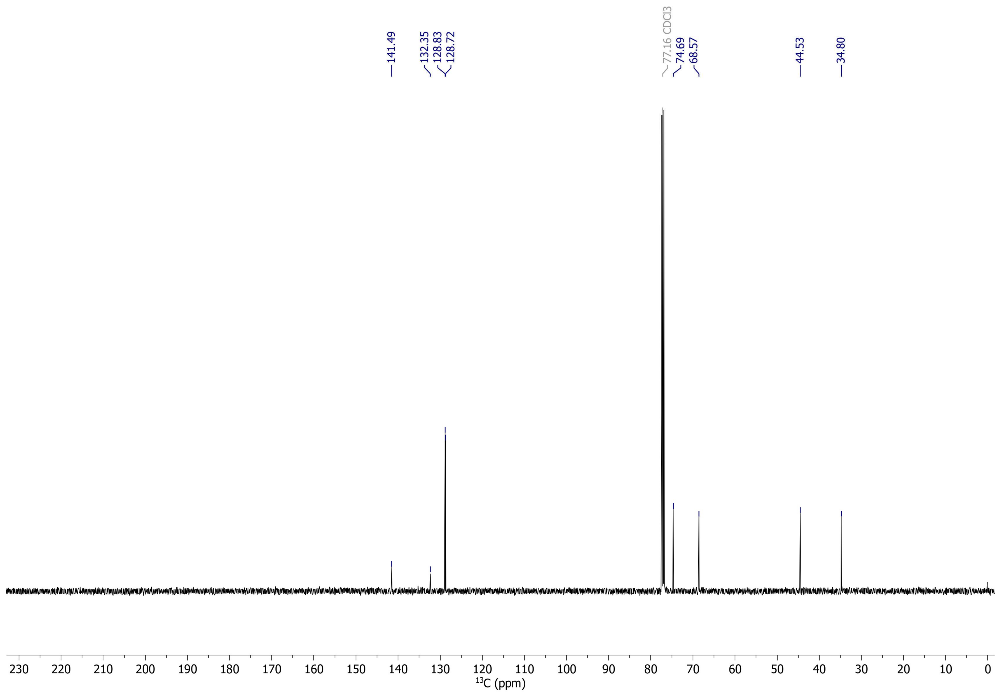

3-(4-(Trifluoromethyl)phenyl)-2,5-dihydrofuran, 2f

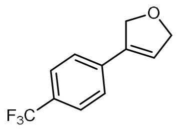

^{1}H NMR (500 MHz, $CDCl_3$)

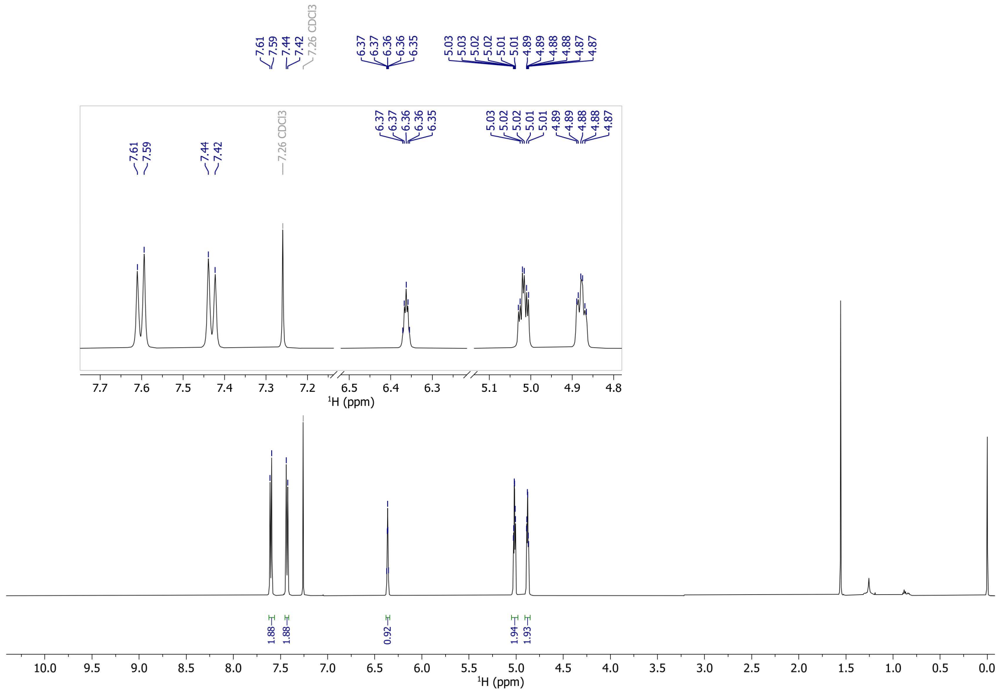

^{13}C NMR (151 MHz, $CDCl_3$)

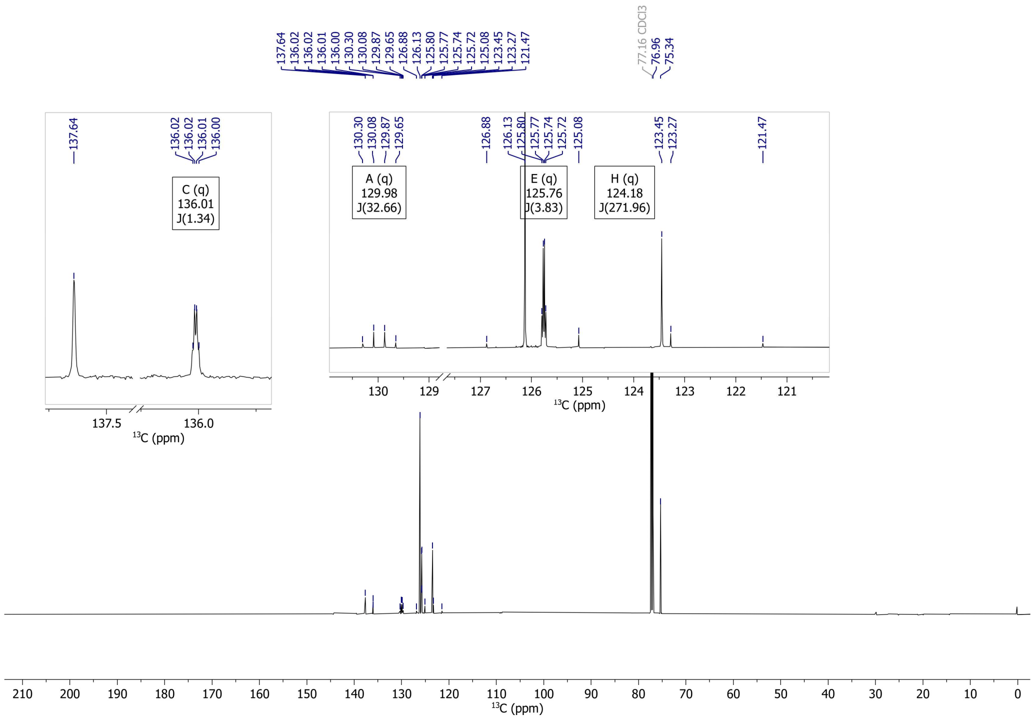

^{19}F NMR (565 MHz, $CDCl_3$)

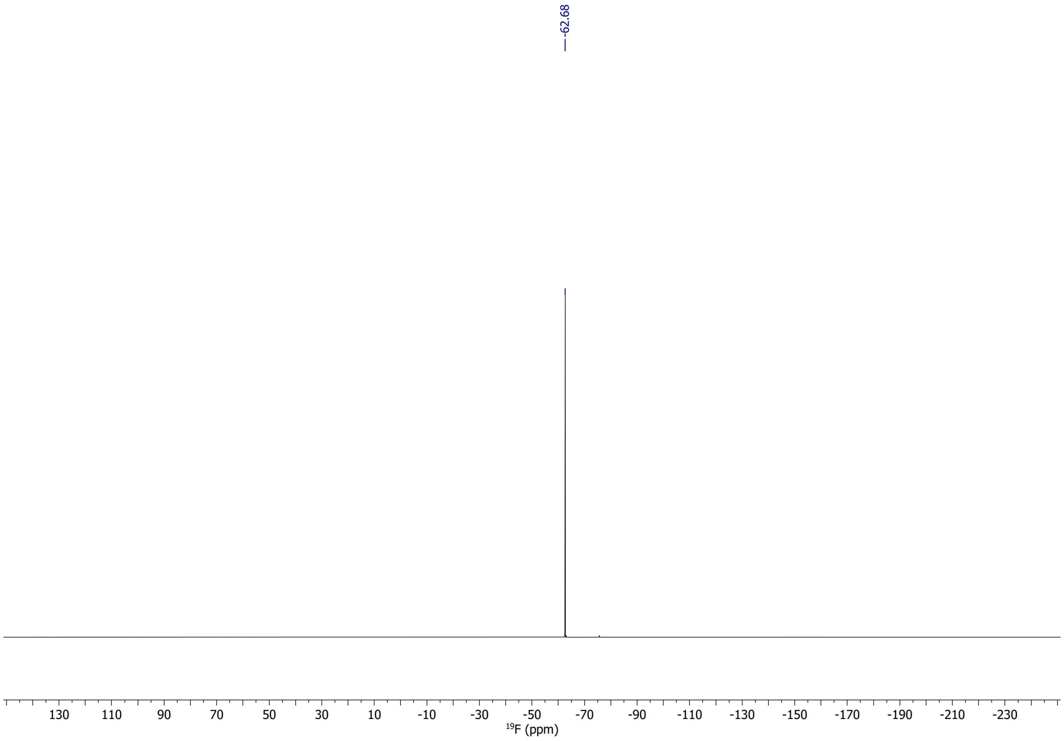

3-(4-Nitrophenyl)-2,5-dihydrofuran, 2g

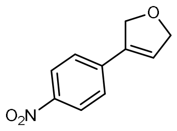

^{1}H NMR (501 MHz, $CDCl_3$)

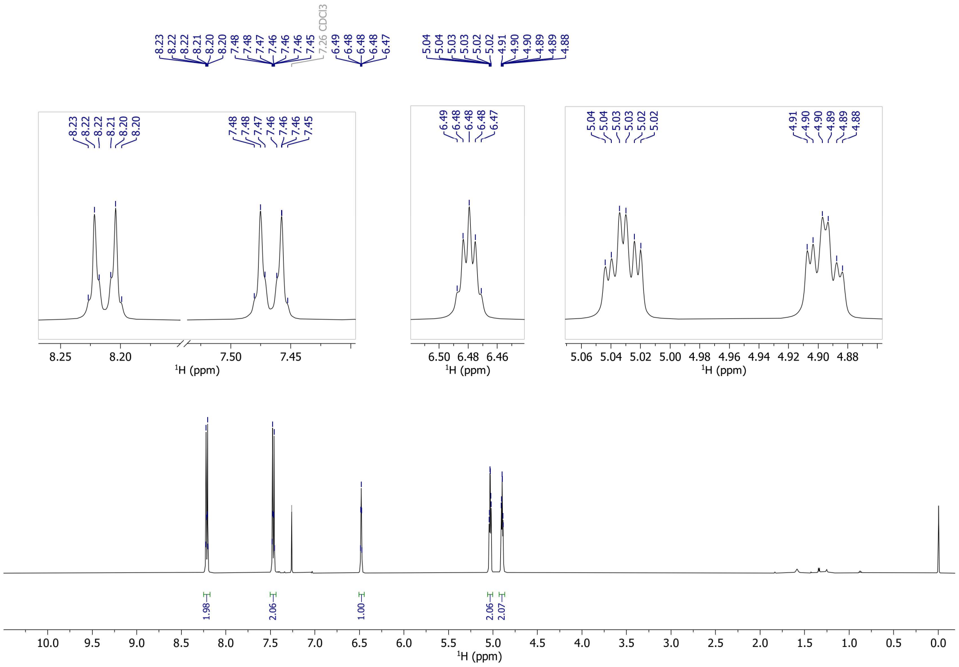

^{13}C NMR (126 MHz, $CDCl_3$)

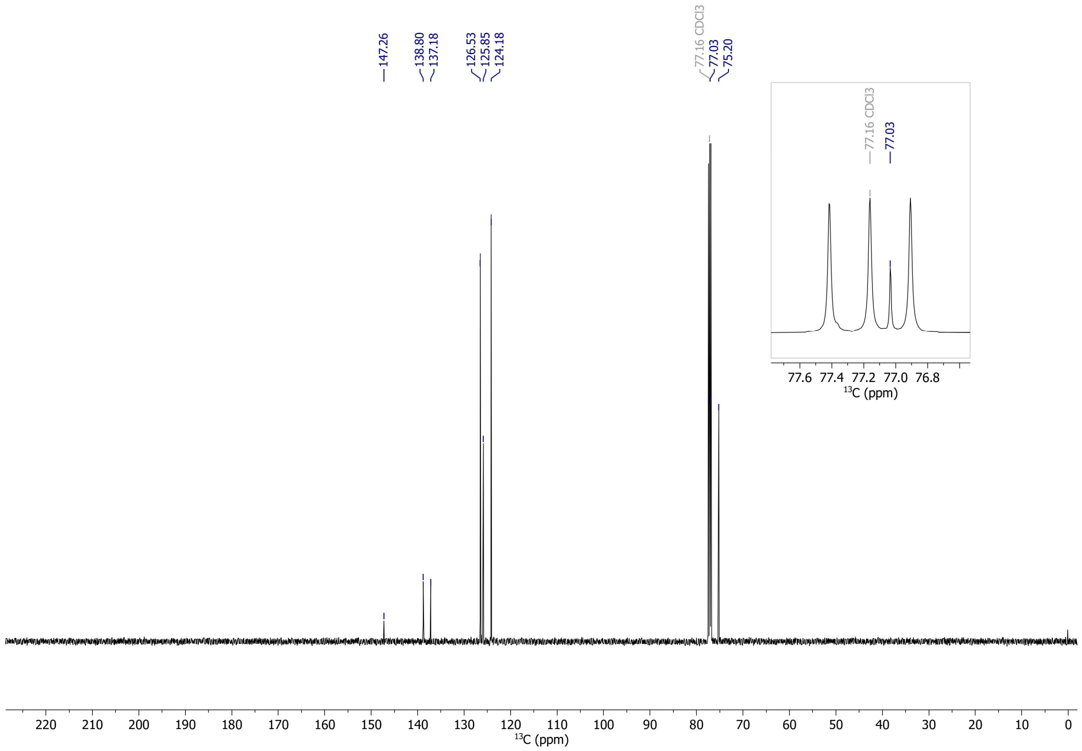

3-(Cyclohexylmethyl)-2,5-dihydrofuran, 2h

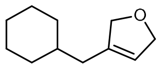

^{1}H NMR (501 MHz, $CDCl_3$)

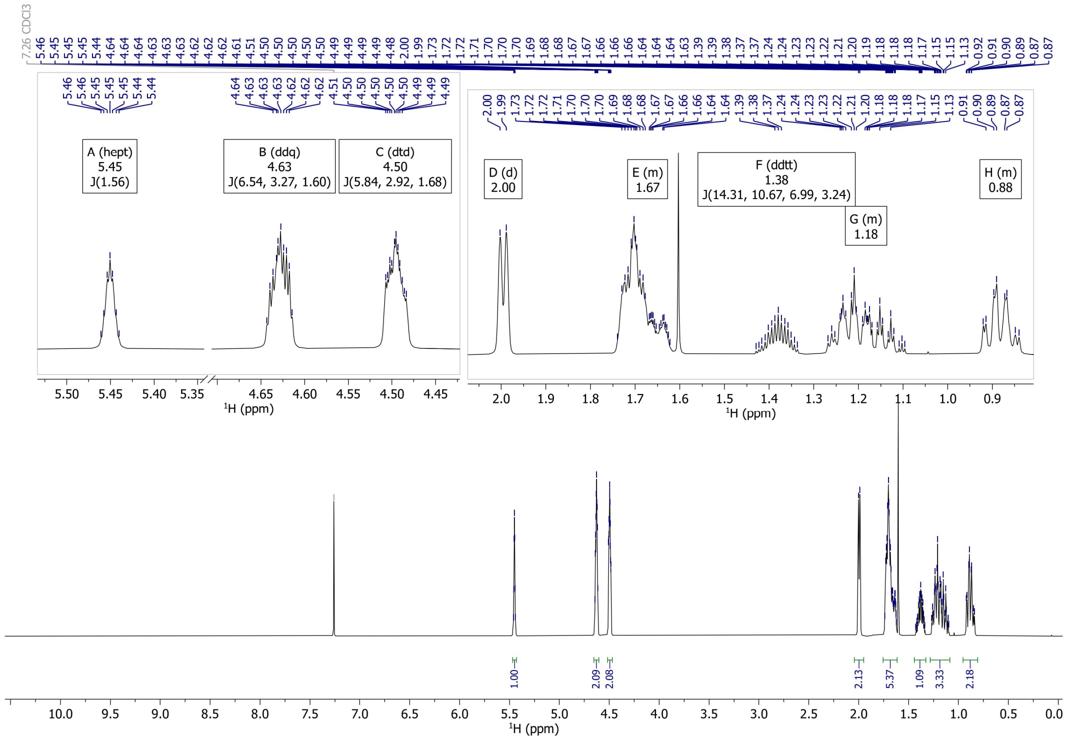

^{13}C NMR (126 MHz, $CDCl_3$)

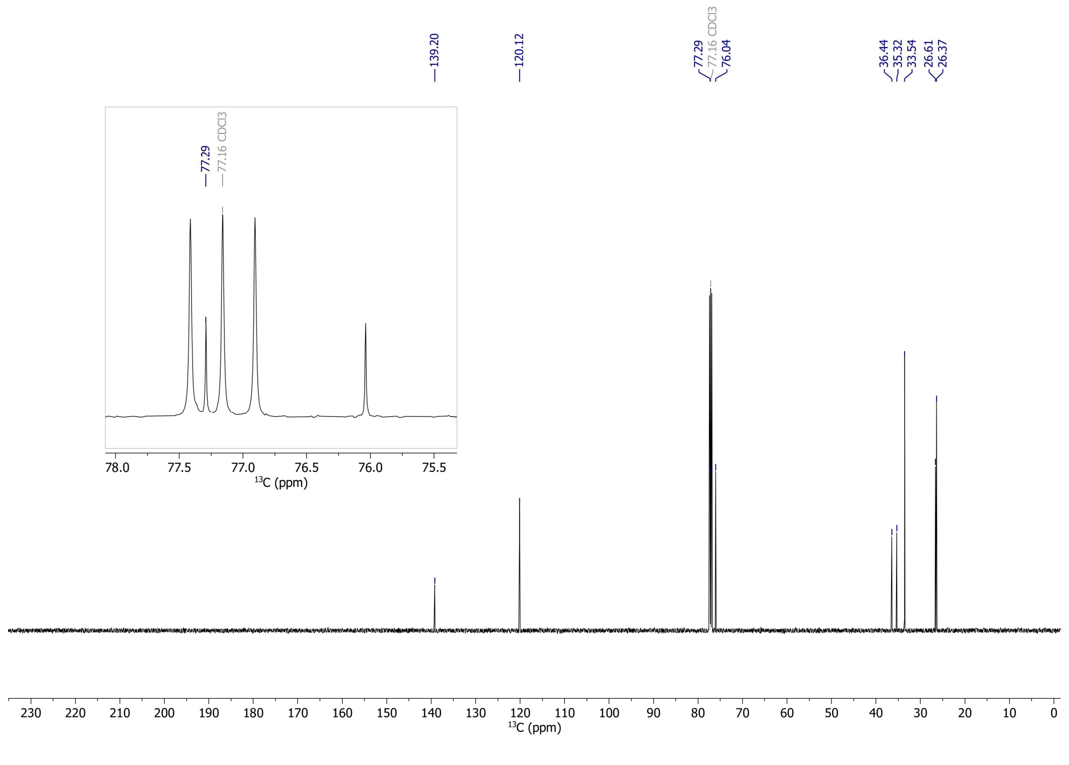

3-Heptyl-2,5-dihydrofuran, 2i

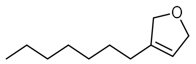

^{1}H NMR (501 MHz, $CDCl_3$)

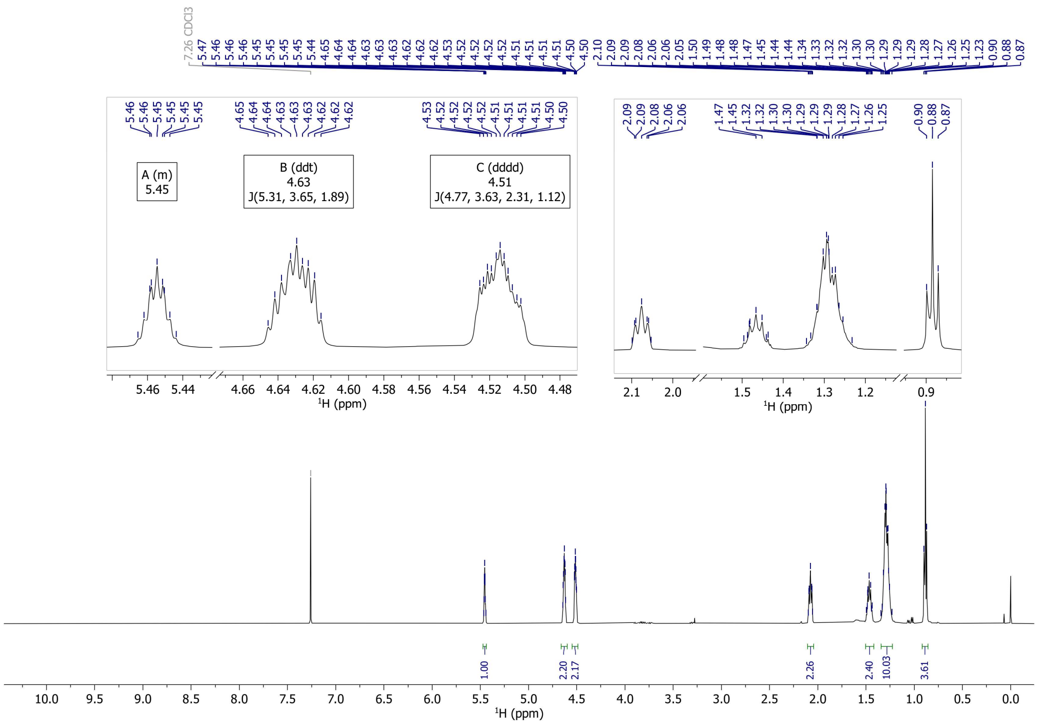

^{13}C NMR (126 MHz, $CDCl_3$)

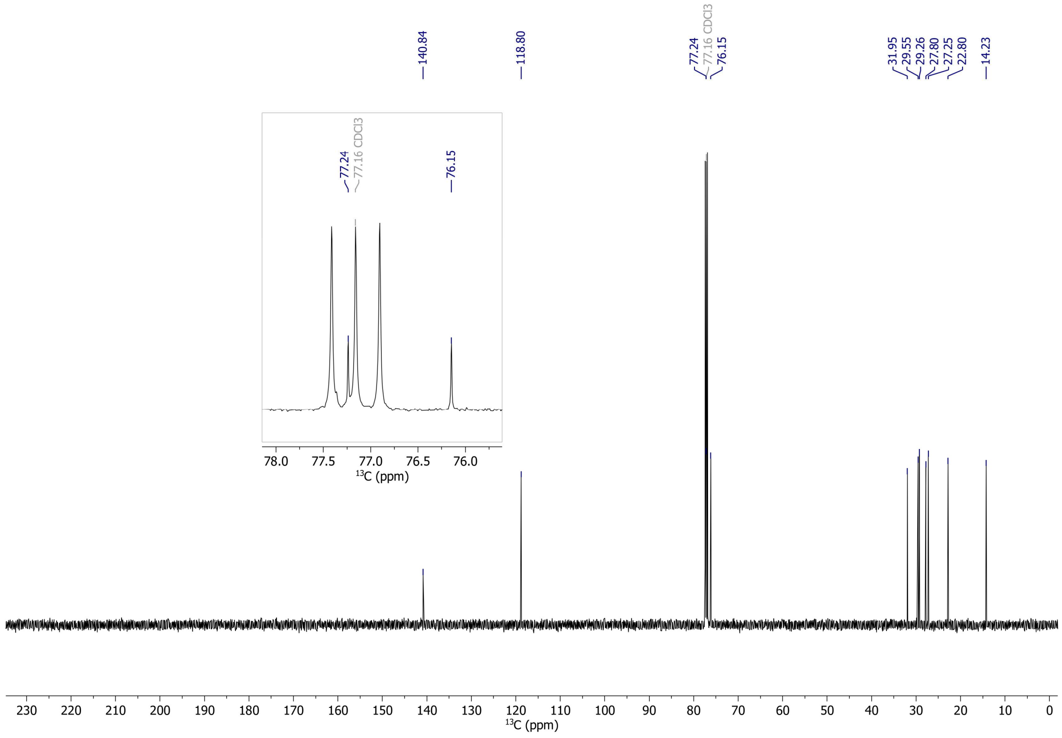

3-Cyclobutyl-2,5-dihydrofuran, 2j

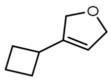

^{1}H NMR (501 MHz, $CDCl_3$)

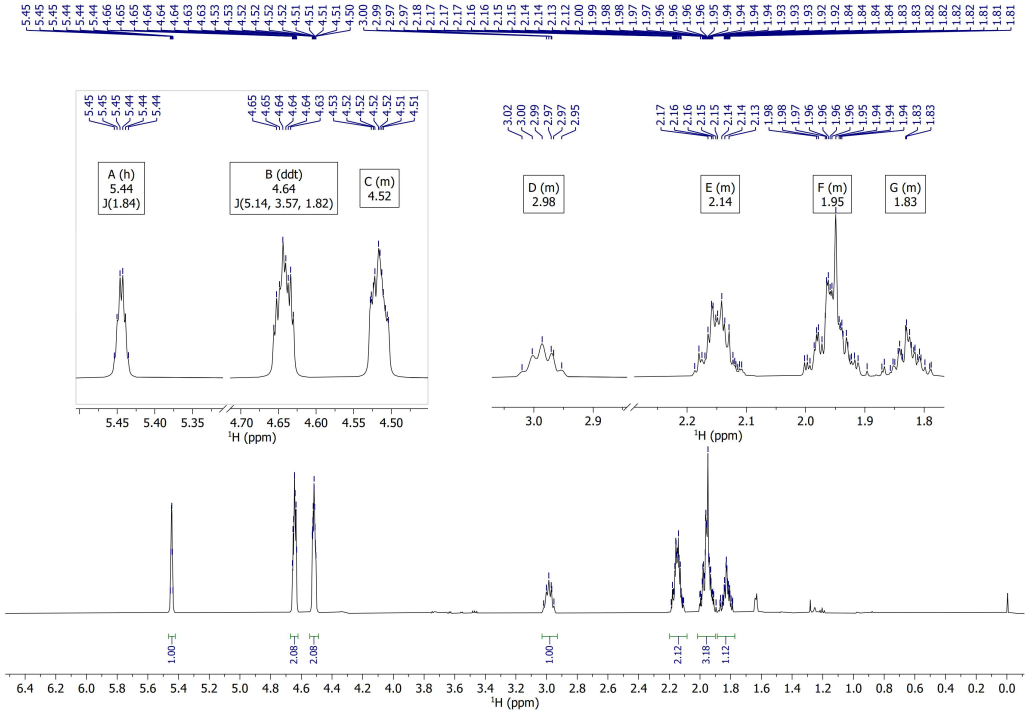

^{13}C NMR (126 MHz, $CDCl_3$)

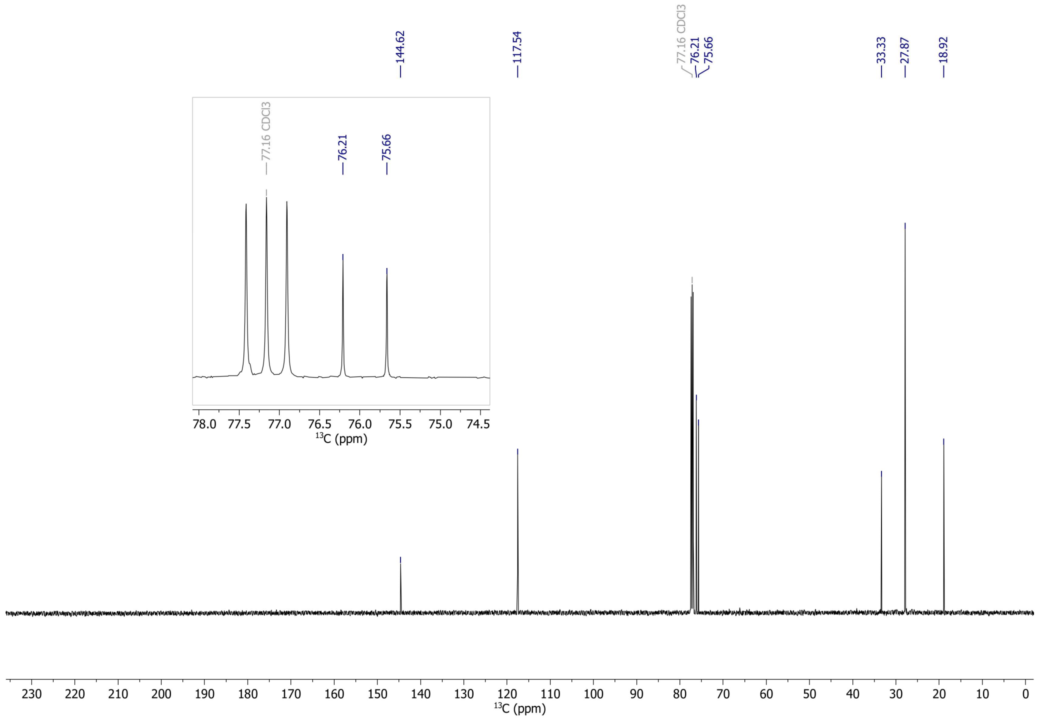

3-(3-Methoxypropyl)-2,5-dihydrofuran, 2k

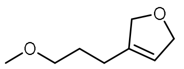

^{1}**H NMR** (501 MHz, $CDCl_3$)

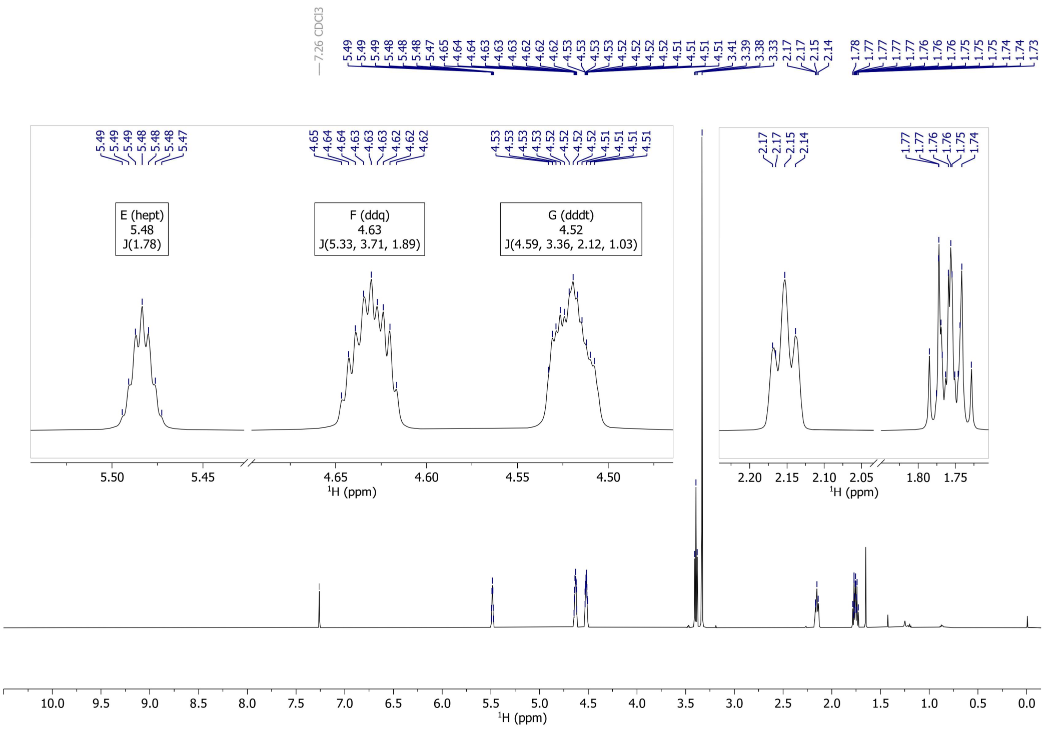

^{13}**C NMR** (126 MHz, $CDCl_3$)

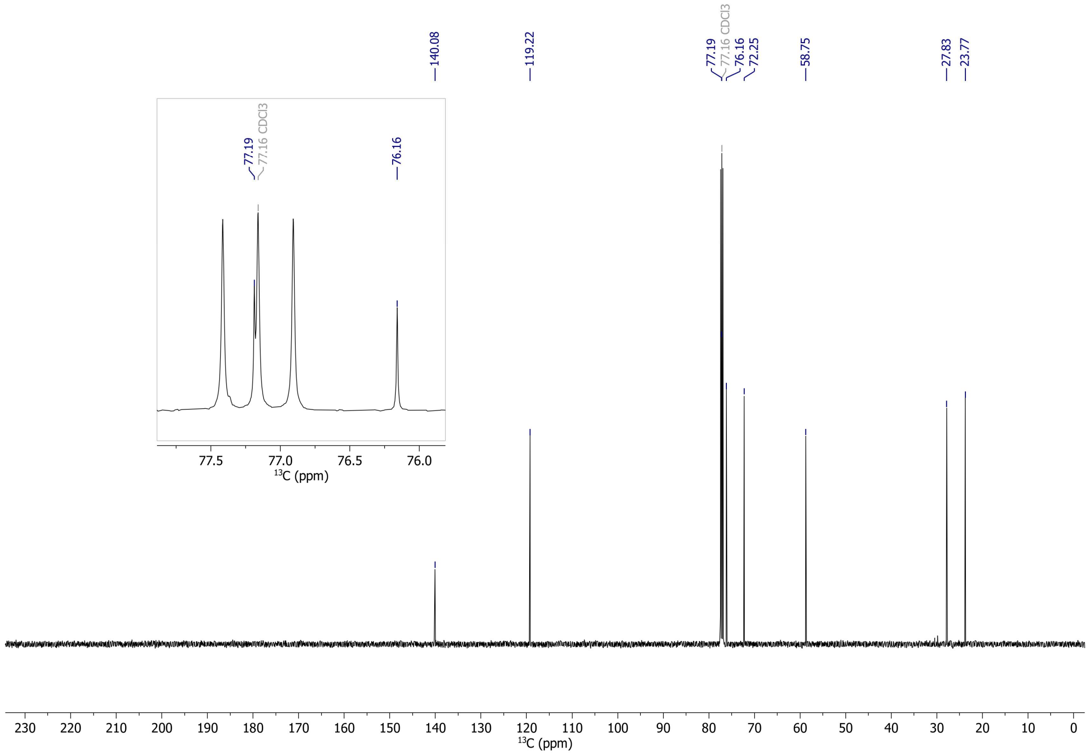

3-(But-3-en-1-yl)-2,5-dihydrofuran, 2n

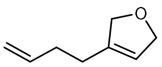

^{1}H NMR (501 MHz, $CDCl_3$)

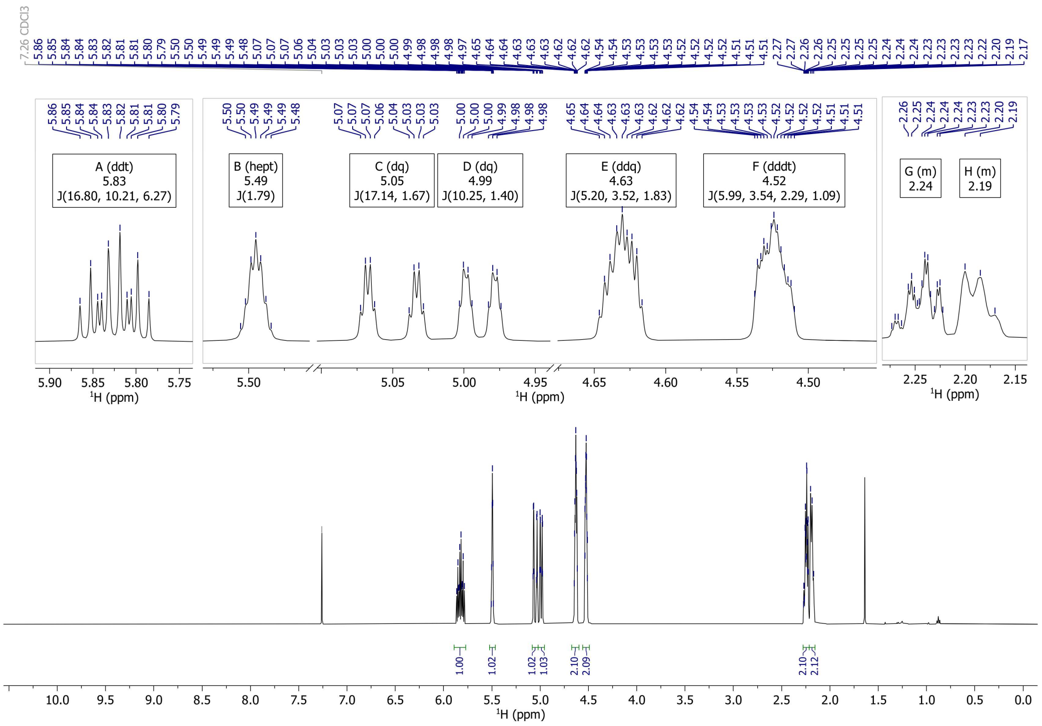

^{13}C NMR (126 MHz, $CDCl_3$)

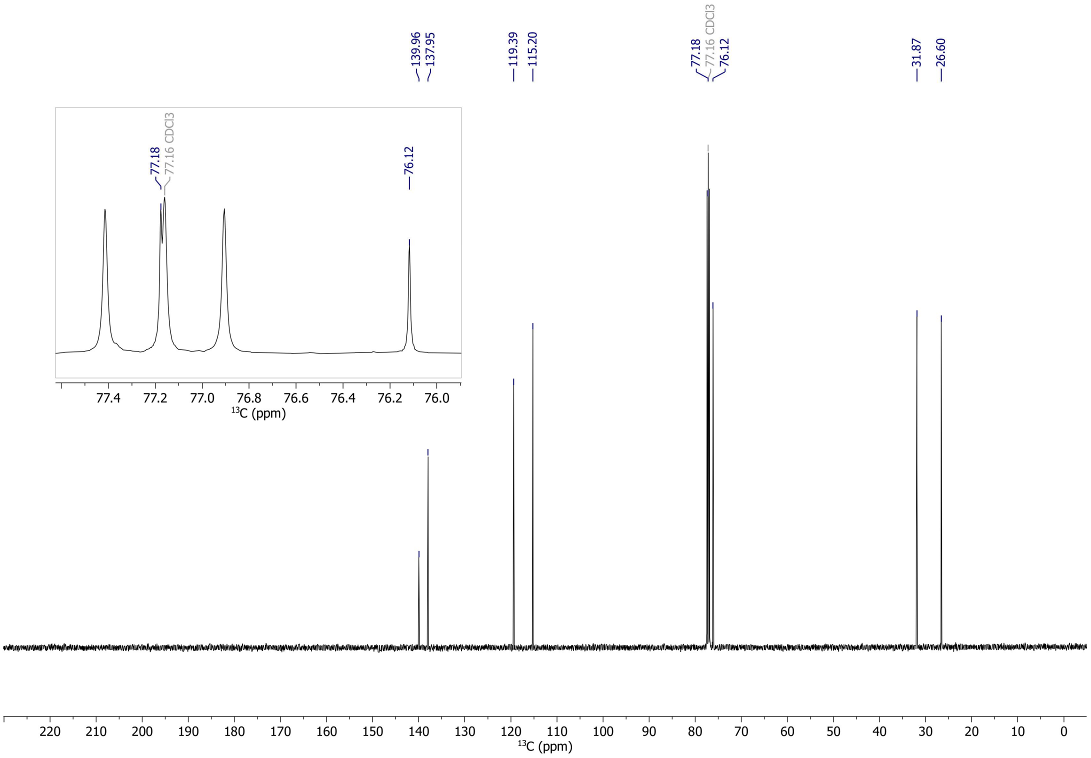

3-(4-Methylpent-3-en-1-yl)-2,5-dihydrofuran, 2,5-dihydro-*Perillene*, 2o

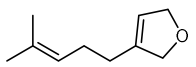

^{1}H NMR (501 MHz, $CDCl_3$)

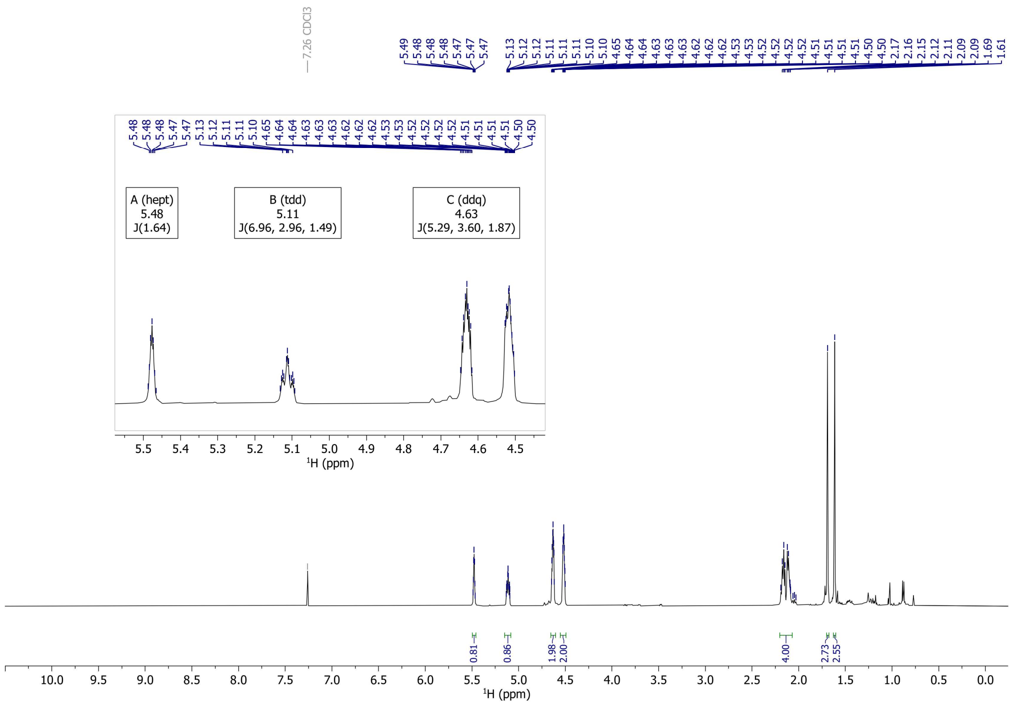

^{13}C NMR (126 MHz, $CDCl_3$)

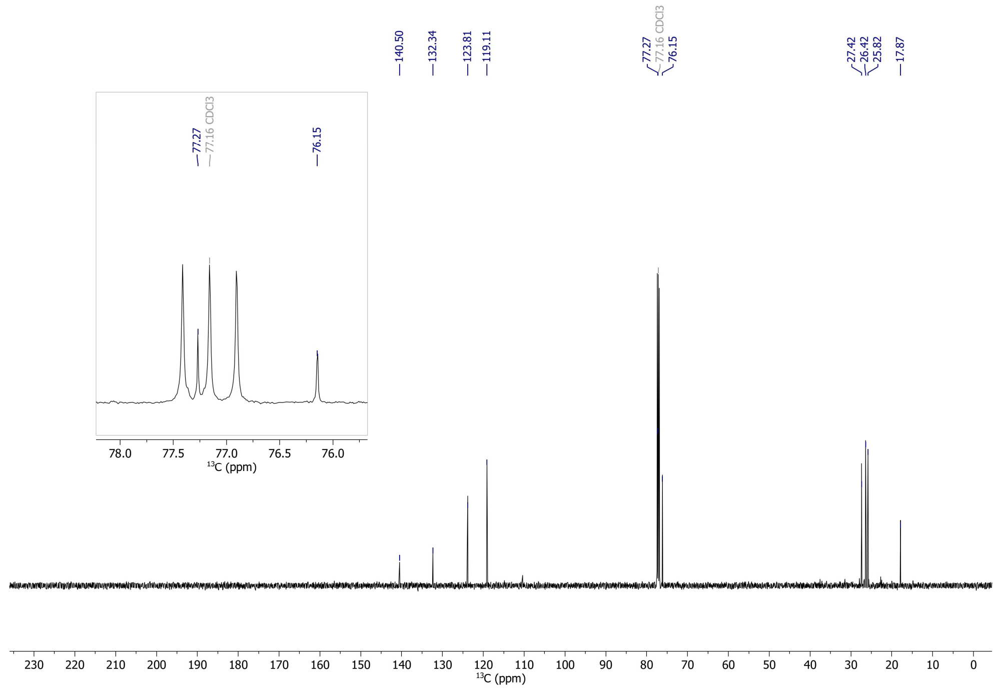

(6*R*,7a*R*)-3,6-Dimethyl-2,4,5,6,7,7a-hexahydrobenzofuran, 2,5-Dihydro-*Menthofuran*, 2p

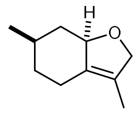

^{1}H NMR (600 MHz, $CDCl_3$)

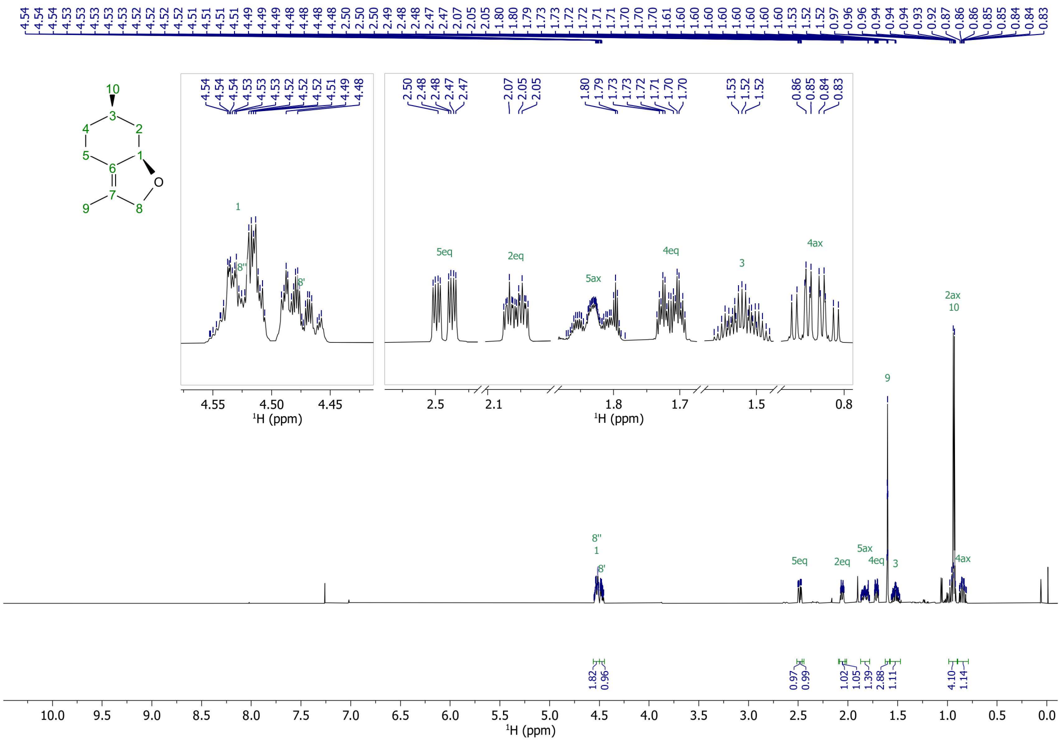

^{13}C NMR (151 MHz, $CDCl_3$)

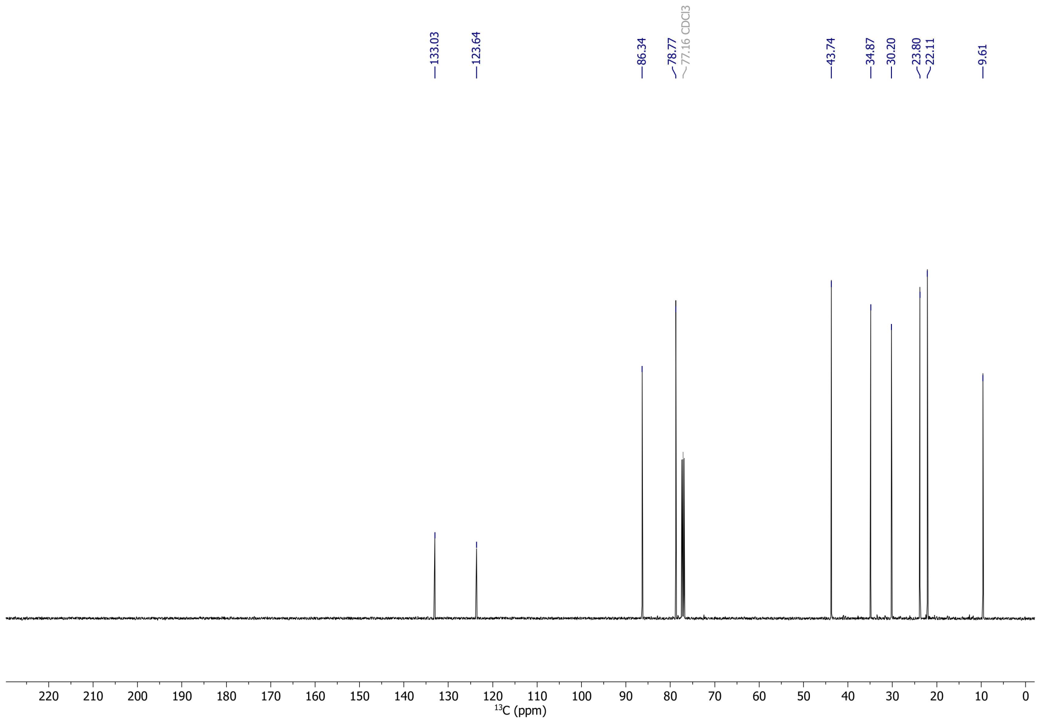

^{1}H–^{13}C HSQC NMR ($CDCl_3$)

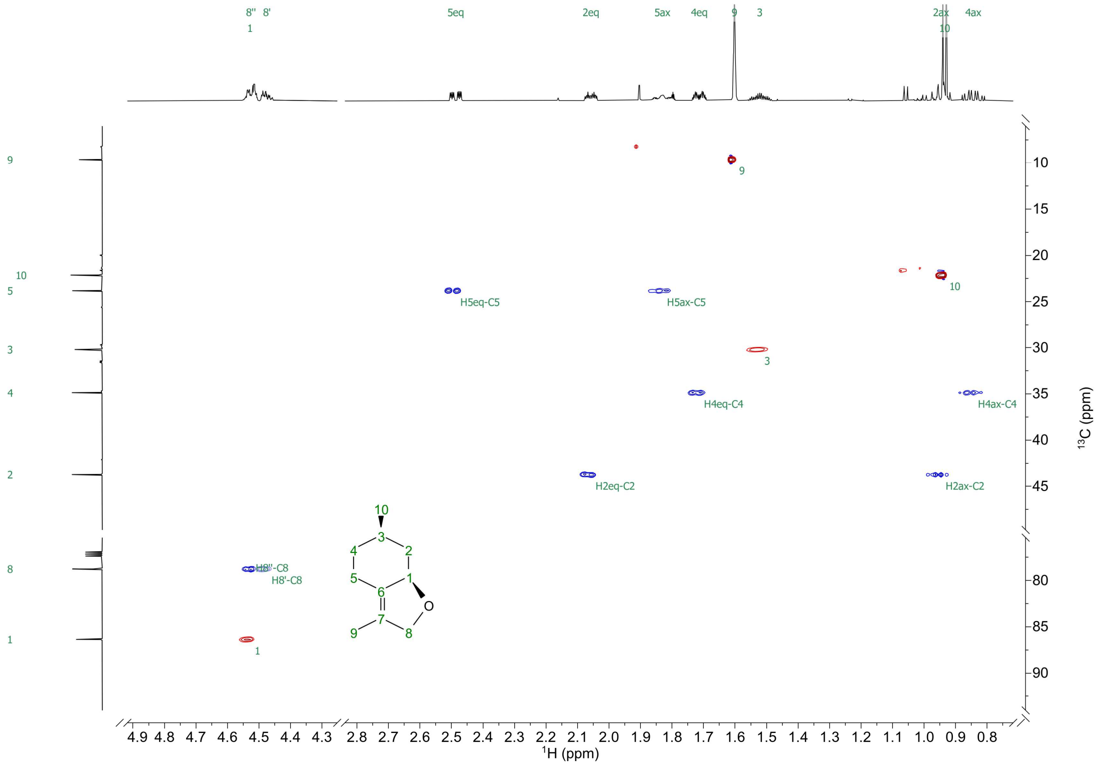

^{1}H–^{13}C HMBC NMR ($CDCl_3$)

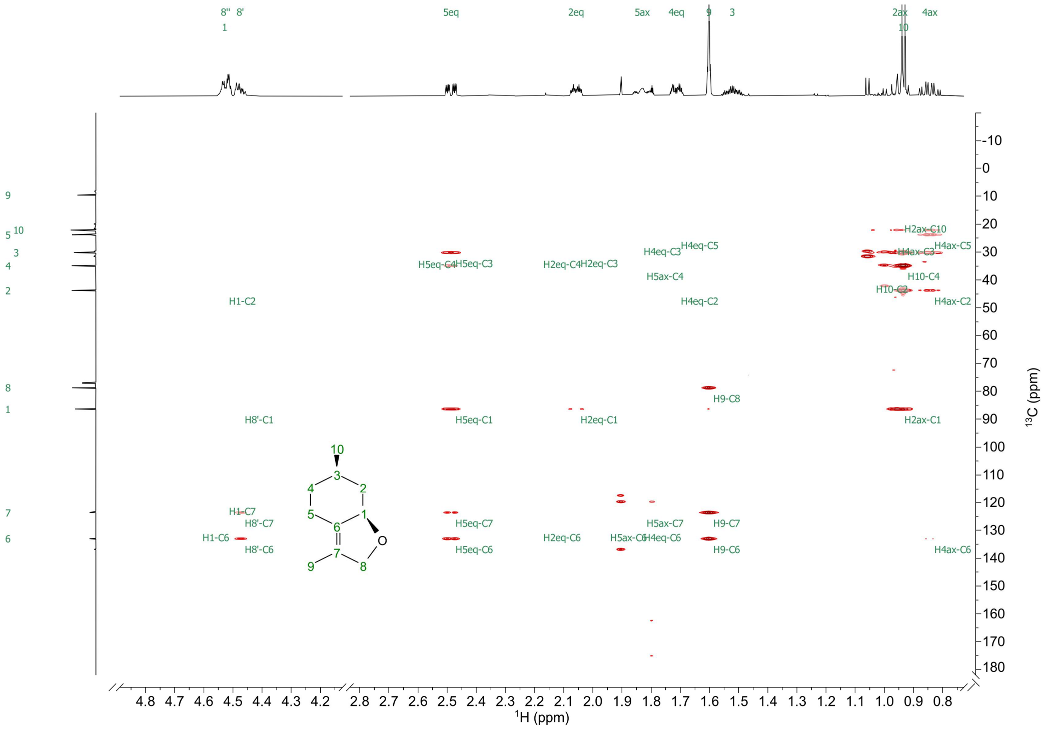

^1H–^1H COSY NMR ($CDCl_3$)

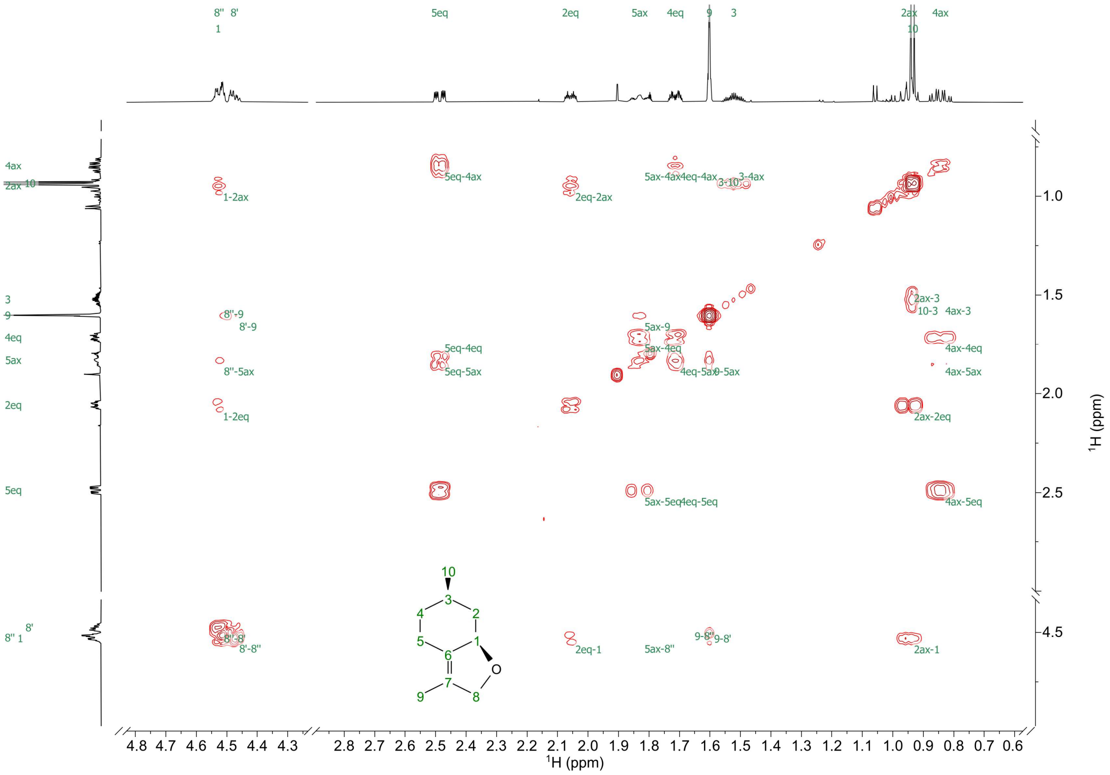

^1H–^1H NOESY NMR ($CDCl_3$)

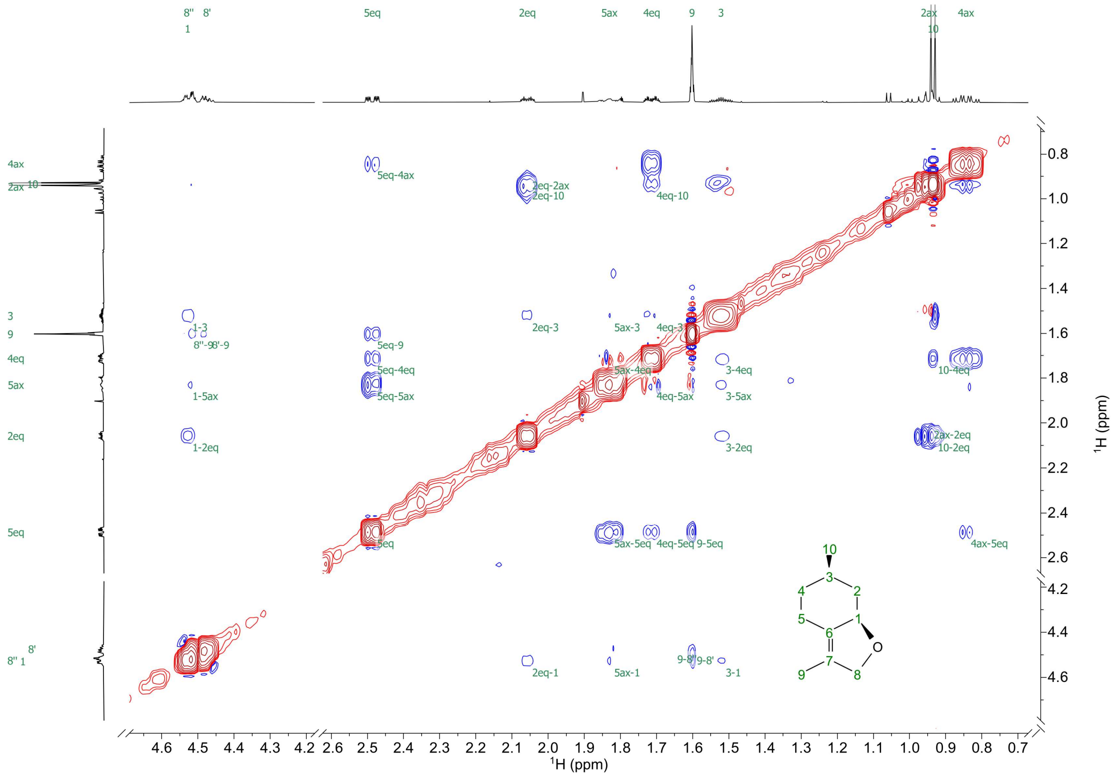

1D-selective NOESY (600 MHz, $CDCl_3$)

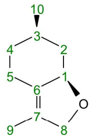

1D selective NOESY of H-9.

major NOEs: H-5eq, H-8', H-8''

1H{off},1D, 600.20 MHz,CDCl3,298.0K, pulse sequence: selnogpzs.2

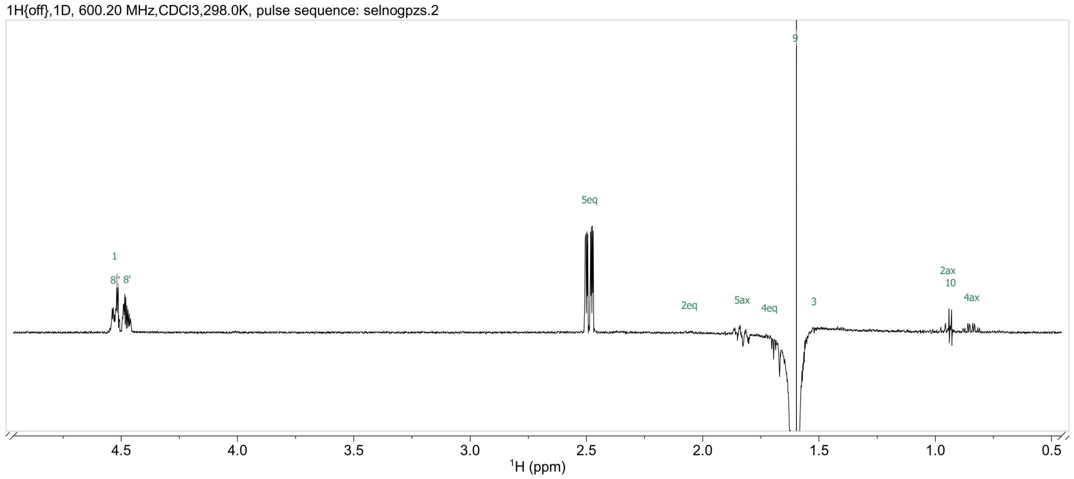

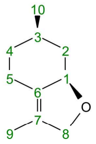

1D selective NOESY of H-3.

major NOEs: H-1, H-2eq,H-4eq, H-5ax

1H{off},1D, 600.20 MHz,CDCl3,298.0K, pulse sequence: selnogpzs.2

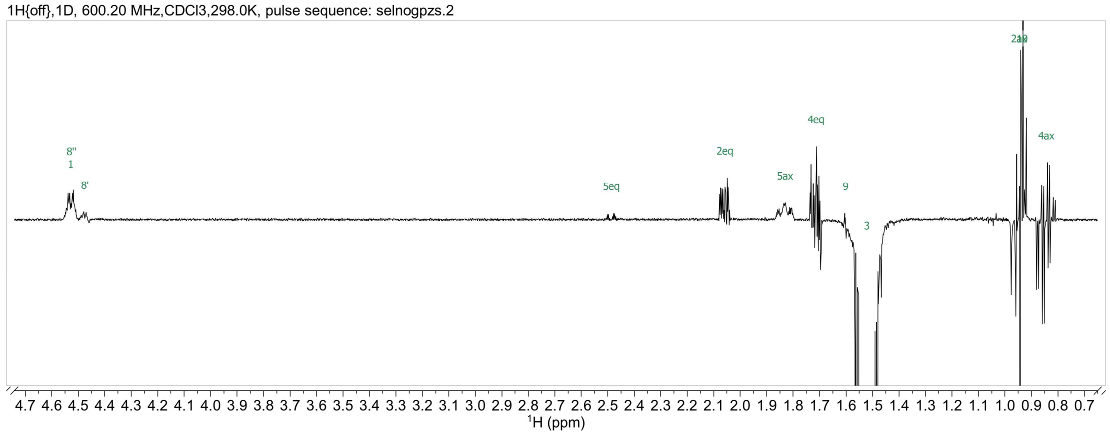

Methyl 2-(4-methyl-2,5-dihydrofuran-2-yl)acetate, 2q

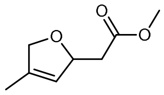

^{1}H NMR (501 MHz, $CDCl_3$)

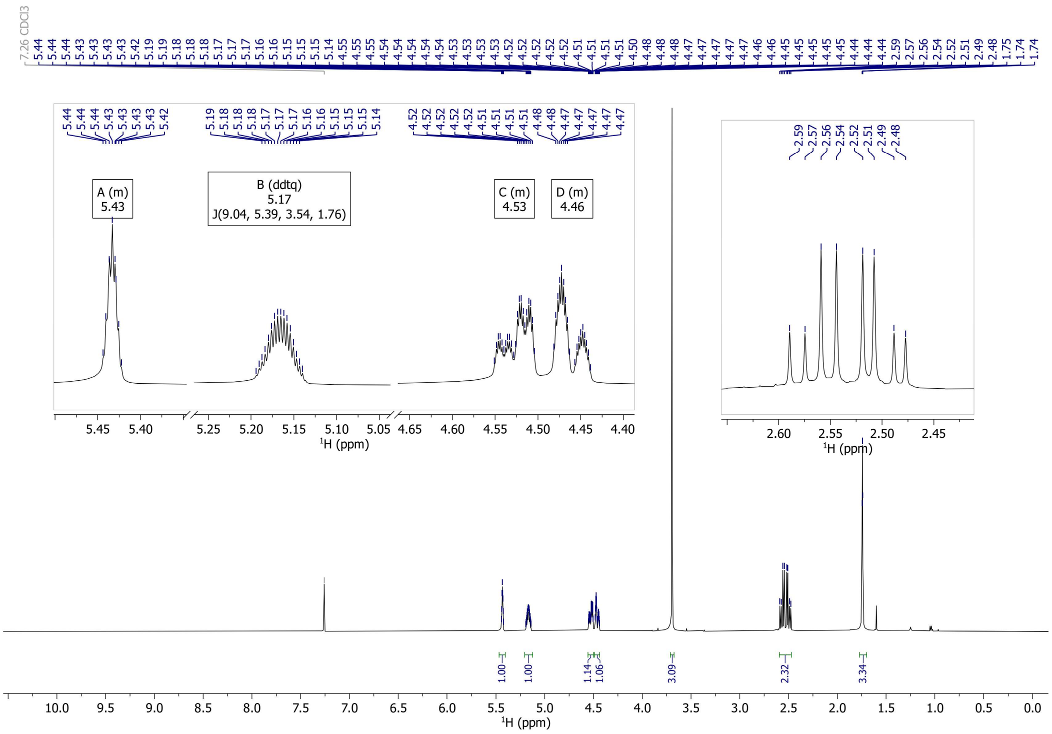

^{13}C NMR (126 MHz, $CDCl_3$)

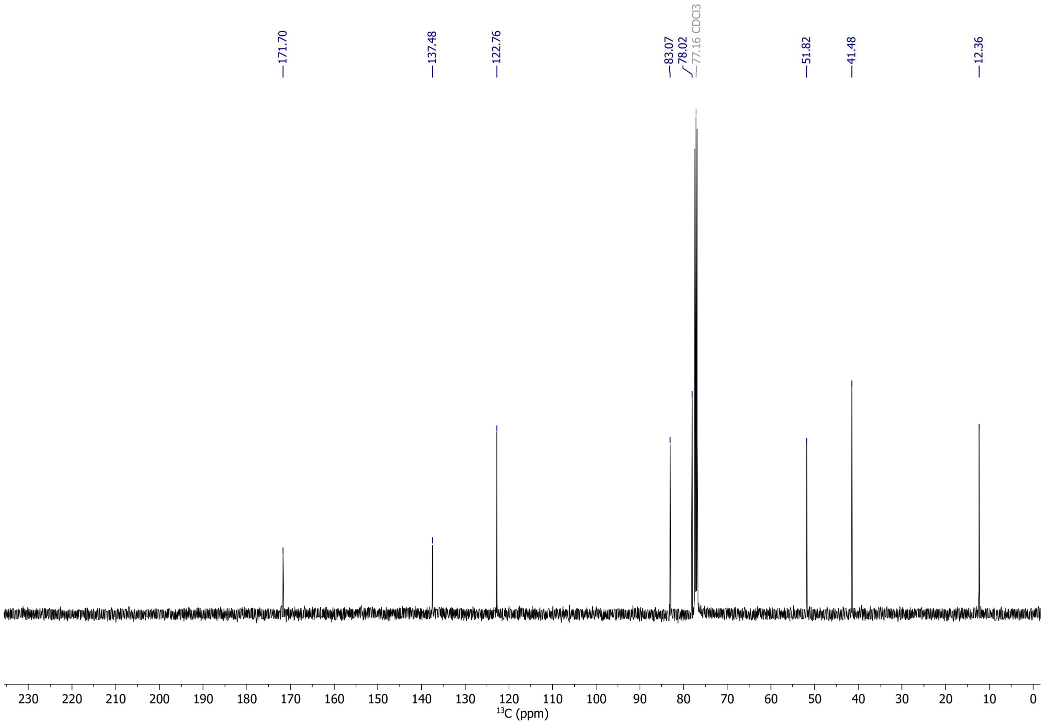

S-((2,5-Dihydrofuran-2-yl)methyl) propanethioate, 2r

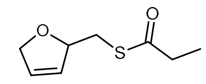

^{1}H NMR (501 MHz, $CDCl_3$)

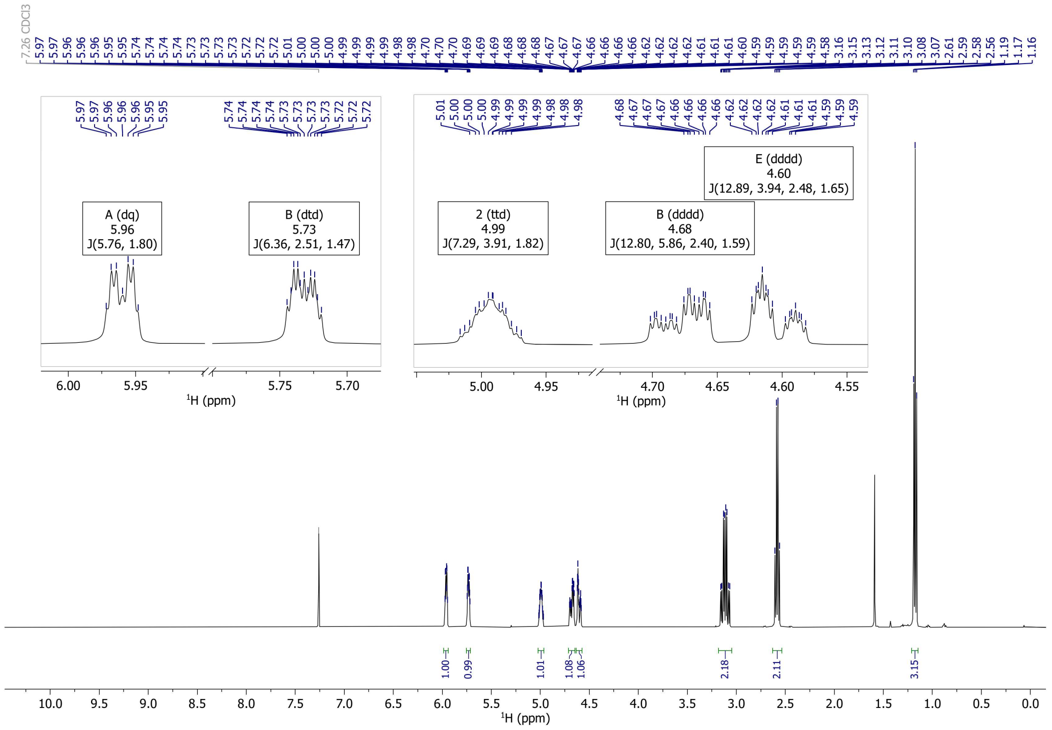

^{13}C NMR (126 MHz, $CDCl_3$)

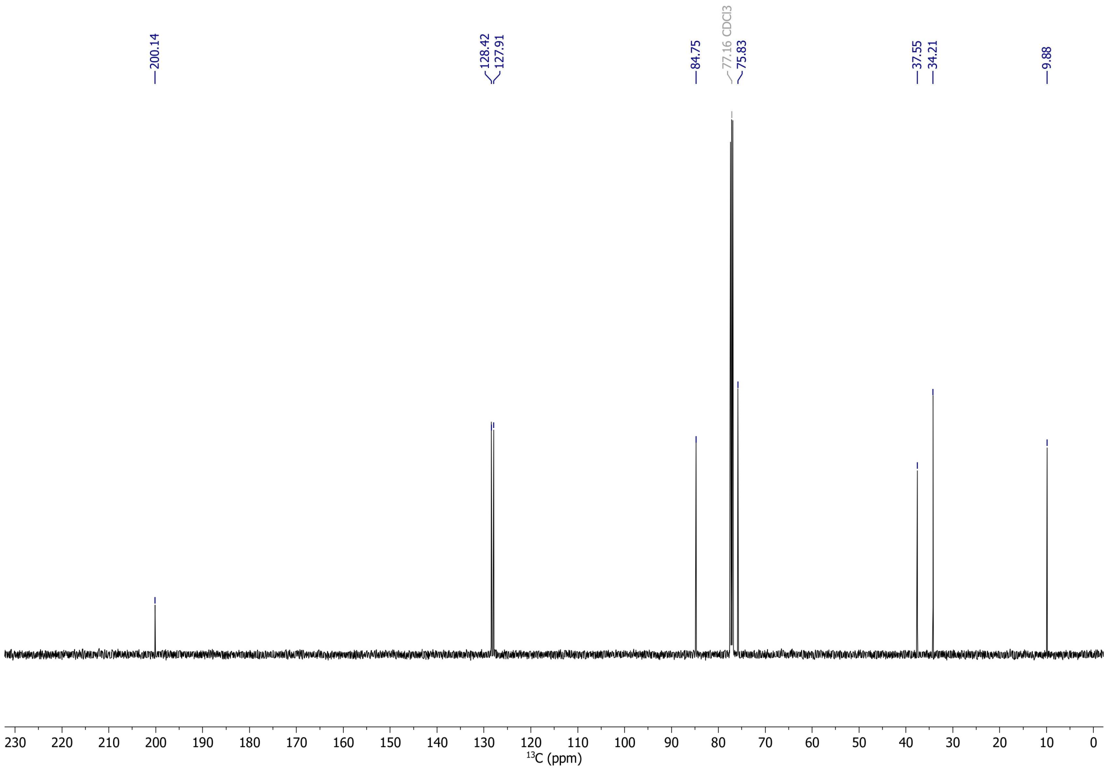

2-Methyl-3-(propyldisulfaneyl)-2,5-dihydrofuran, 2s

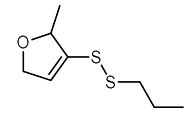

^{1}H NMR (501 MHz, CD_2Cl_2)

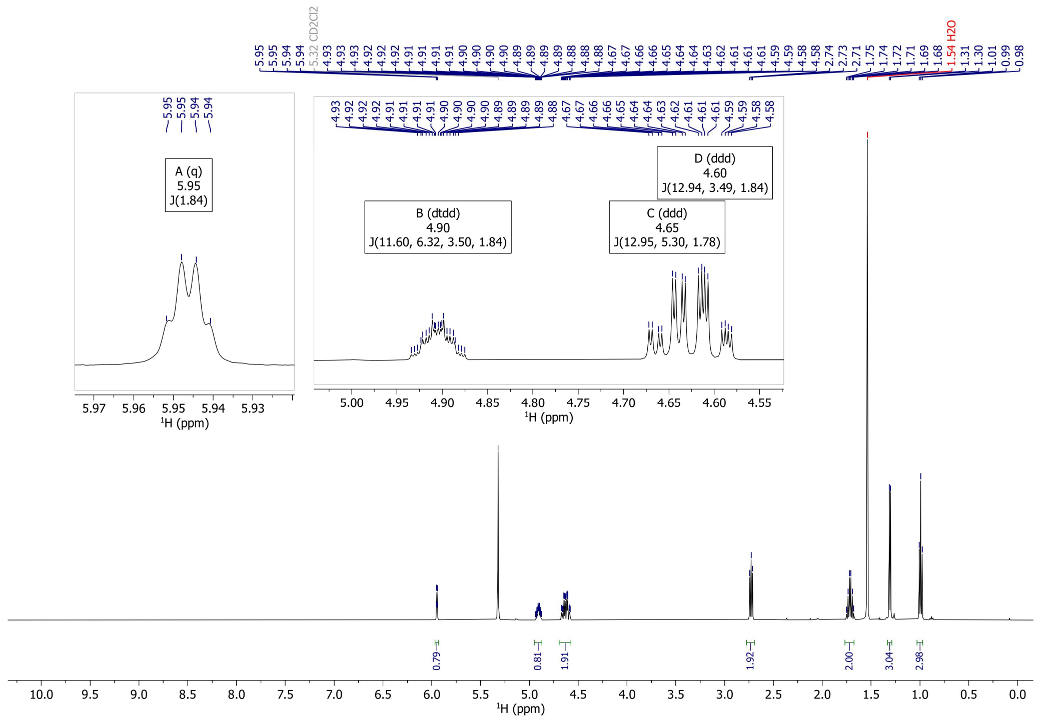

^{13}C NMR (151 MHz, CD_2Cl_2)

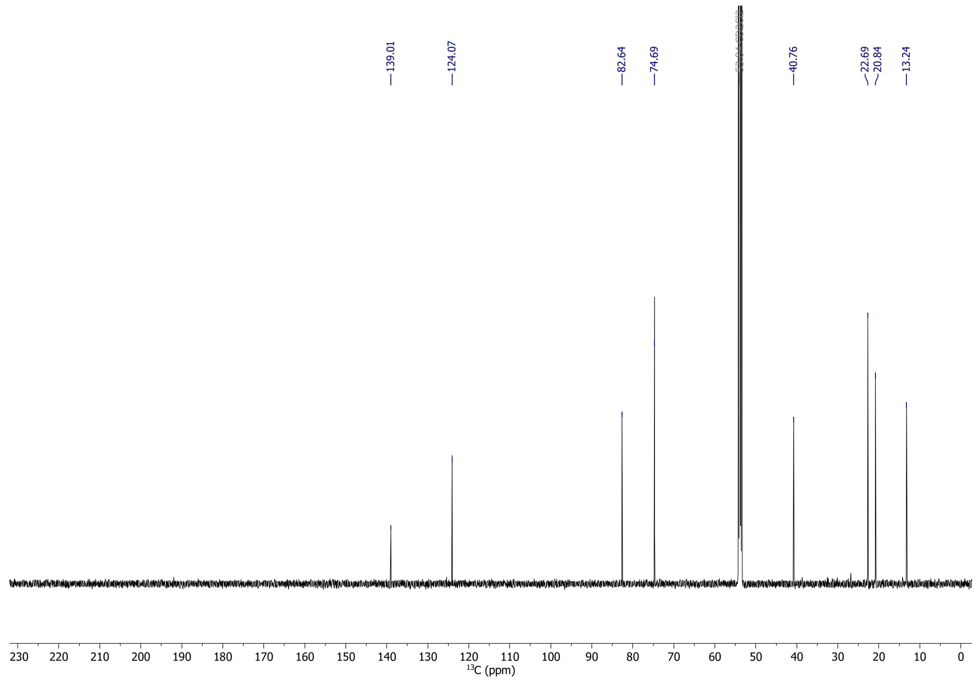

Methyl 3-methyl-2,5-dihydrofuran-2-carboxylate, 2t

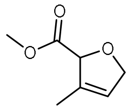

^{1}H NMR (501 MHz, $CDCl_3$)

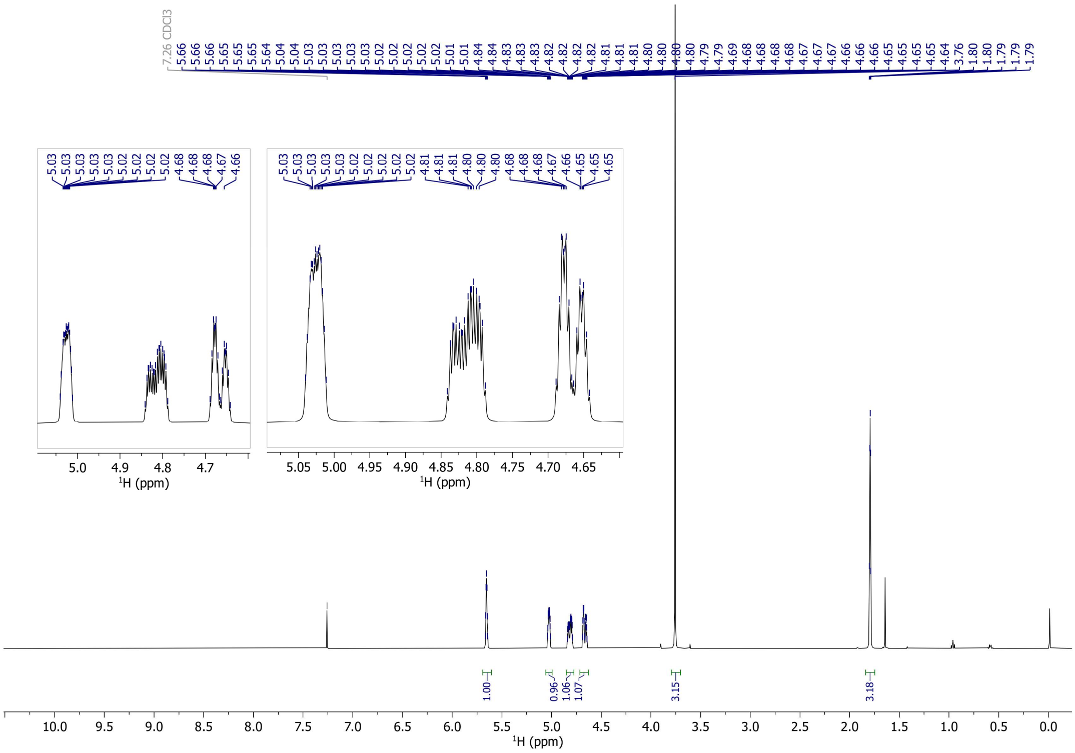

^{13}C NMR (126 MHz, $CDCl_3$)

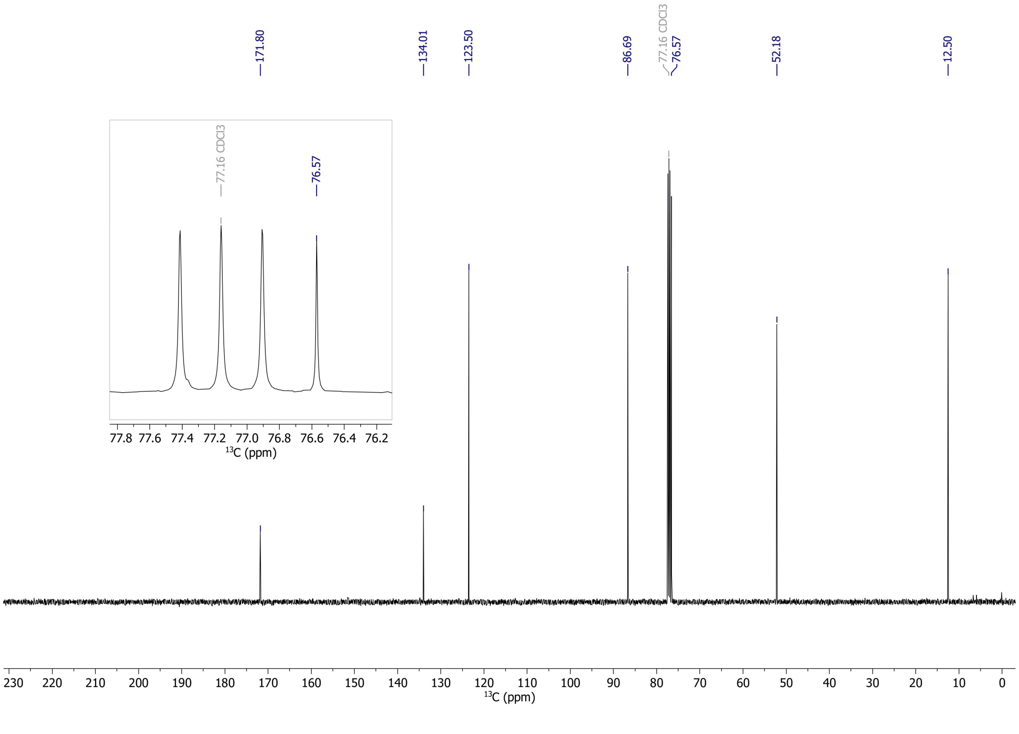

2-Pentyltetrahydrofuran, 4h

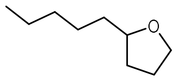

^{1}H NMR (501 MHz, $CDCl_3$)

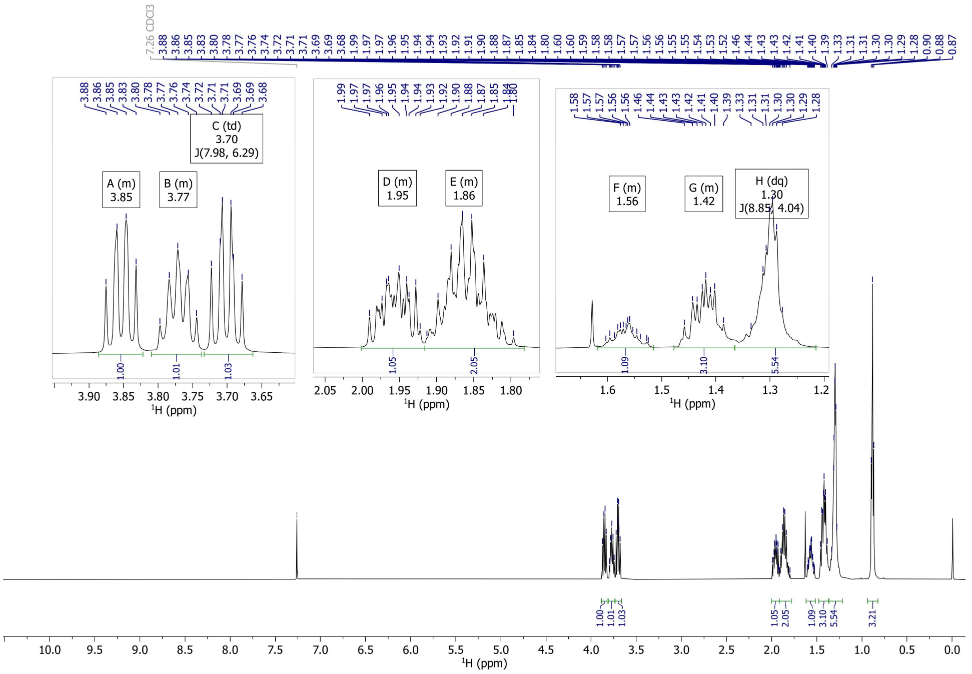

^{13}C NMR (126 MHz, $CDCl_3$)

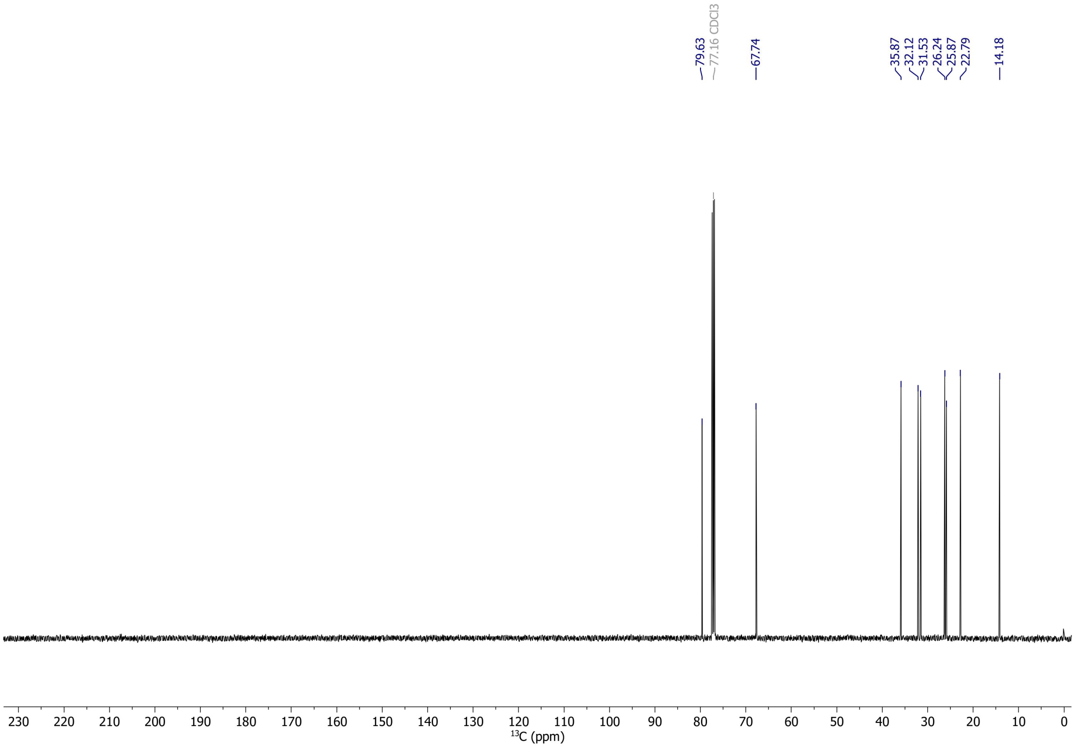

***Trans*-3-(4-(*tert*-butyl)phenyl)-2-methyltetrahydrofuran, 4j**

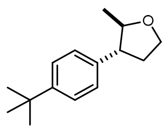

^{1}H NMR (501 MHz, $CDCl_3$)

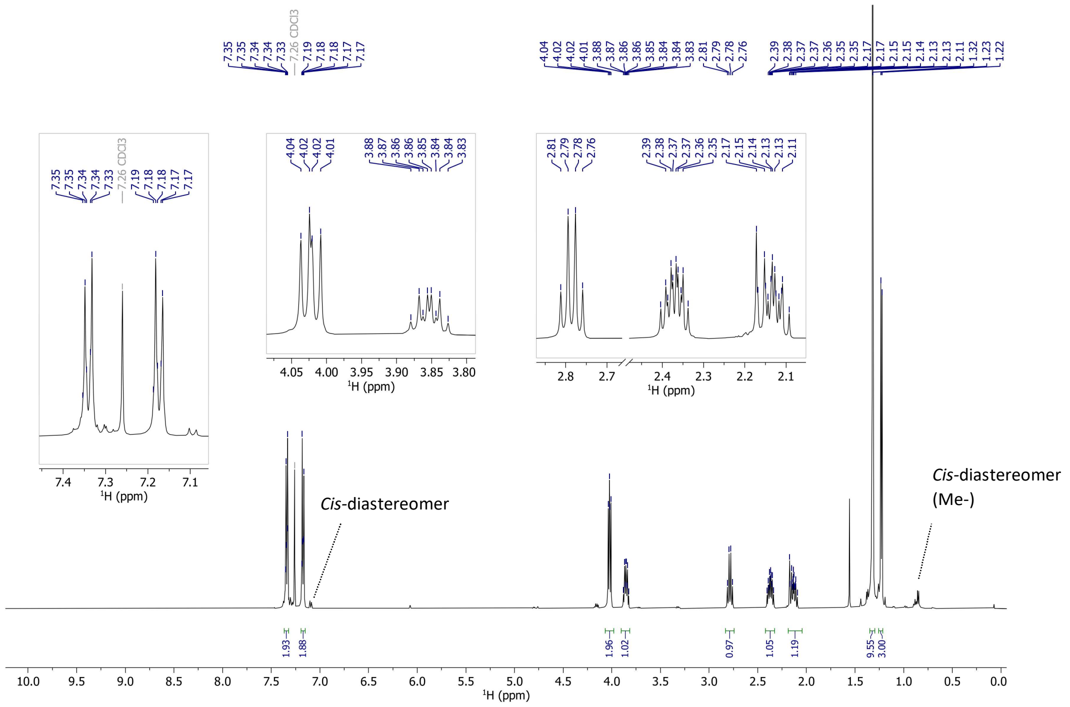

^{13}C NMR (126 MHz, $CDCl_3$)

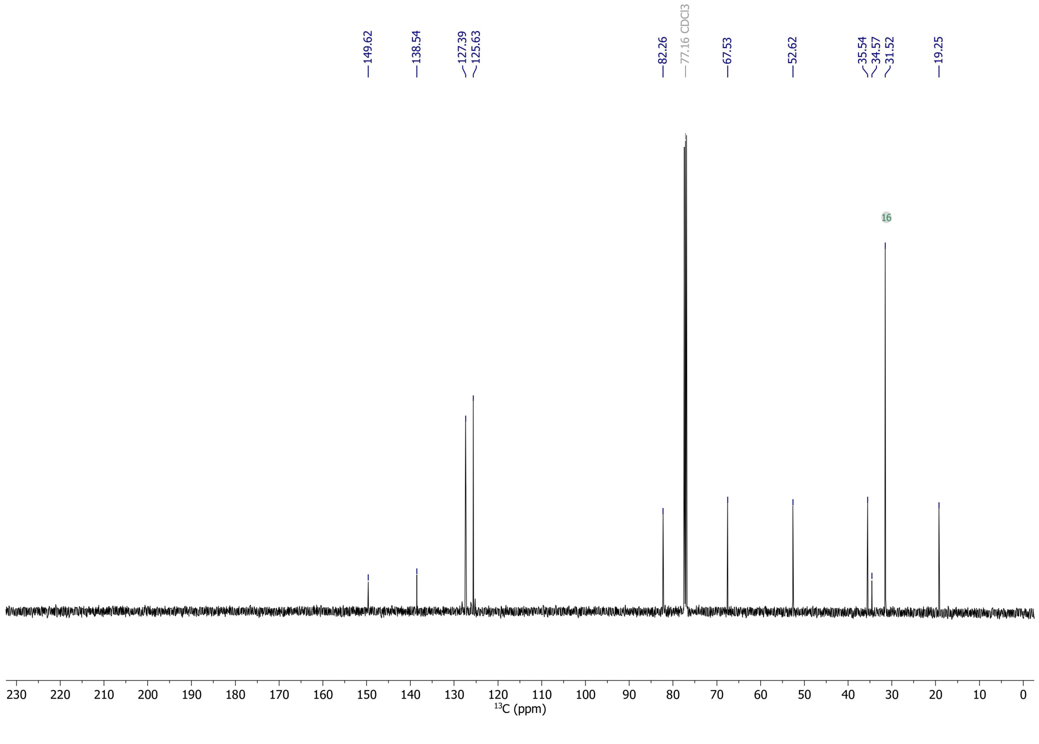

Analysis of a mixture of *Cis*- and *Trans*-3-(4-(*tert*-butyl)phenyl)-2-methyltetrahydrofuran, 4j

1D-TOCSY (600 MHz, $CDCl_3$)

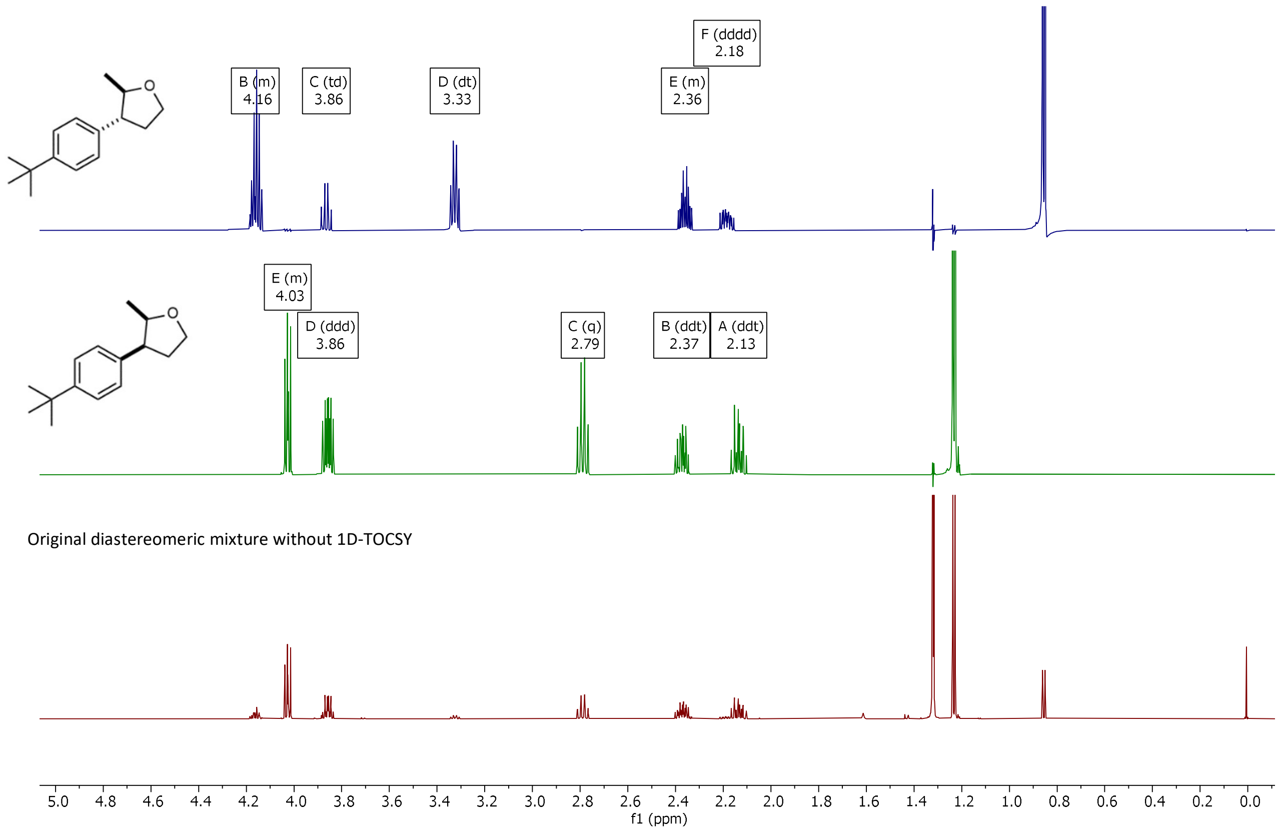

Ethyl *trans*-2-butyltetrahydrofuran-carboxylate, 4k

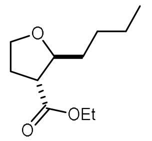

^{1}H NMR (600 MHz, C_6D_6)

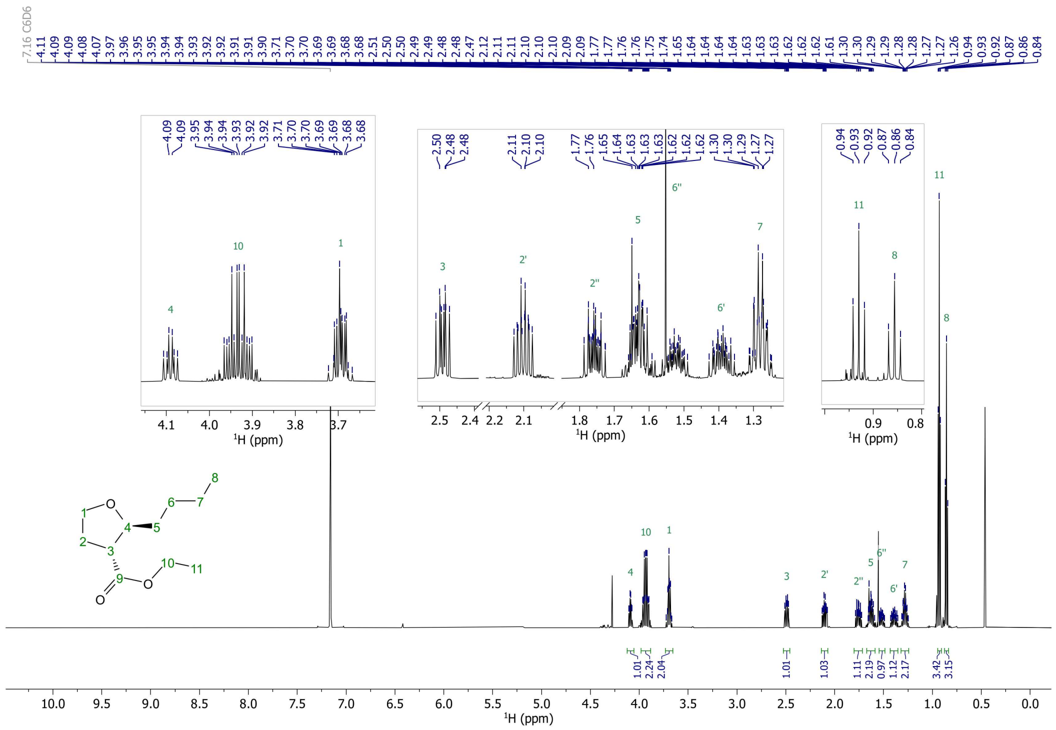

^{13}C NMR (150 MHz, C_6D_6)

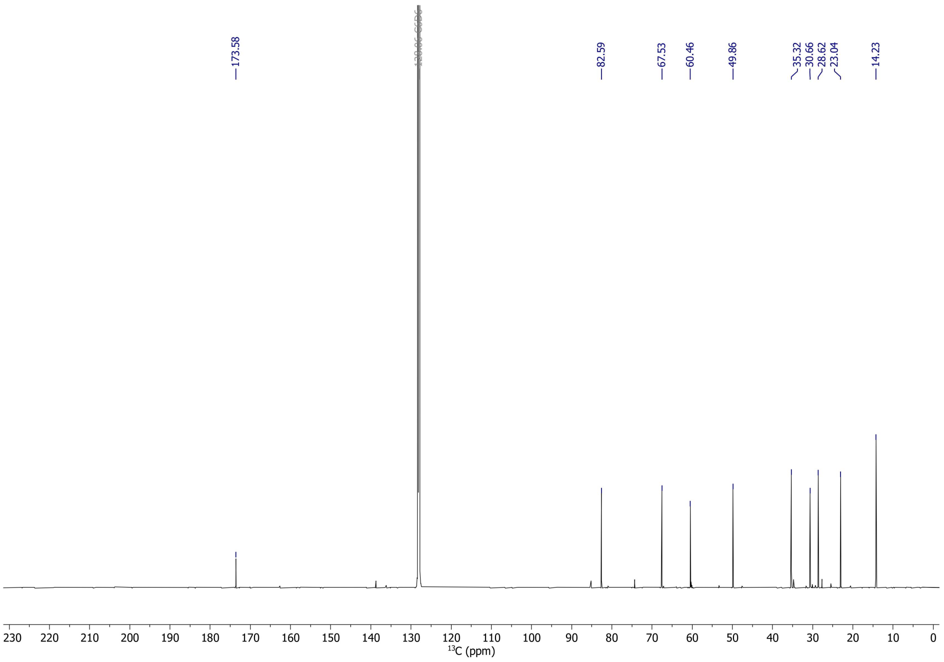

^{1}H–^{13}C HSQC NMR (C_6D_6)

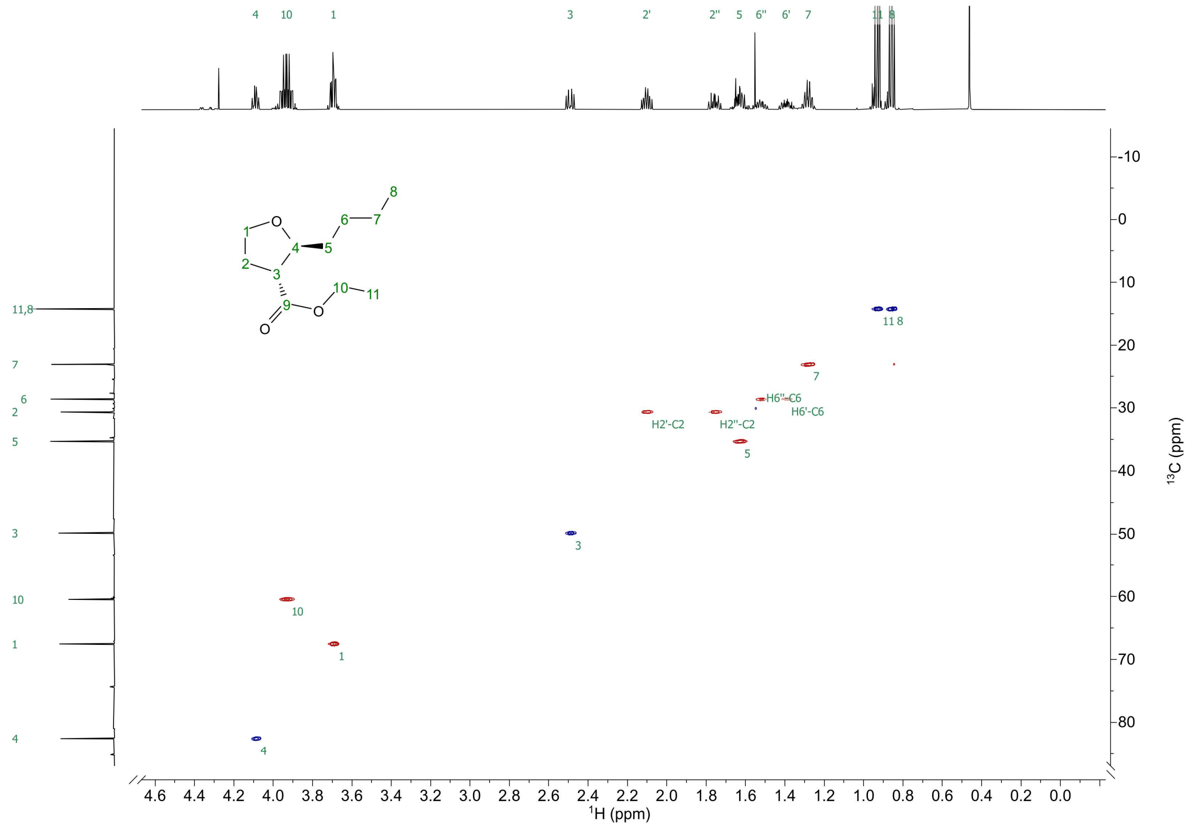

^{1}H–^{13}C HMBC NMR (C_6D_6)

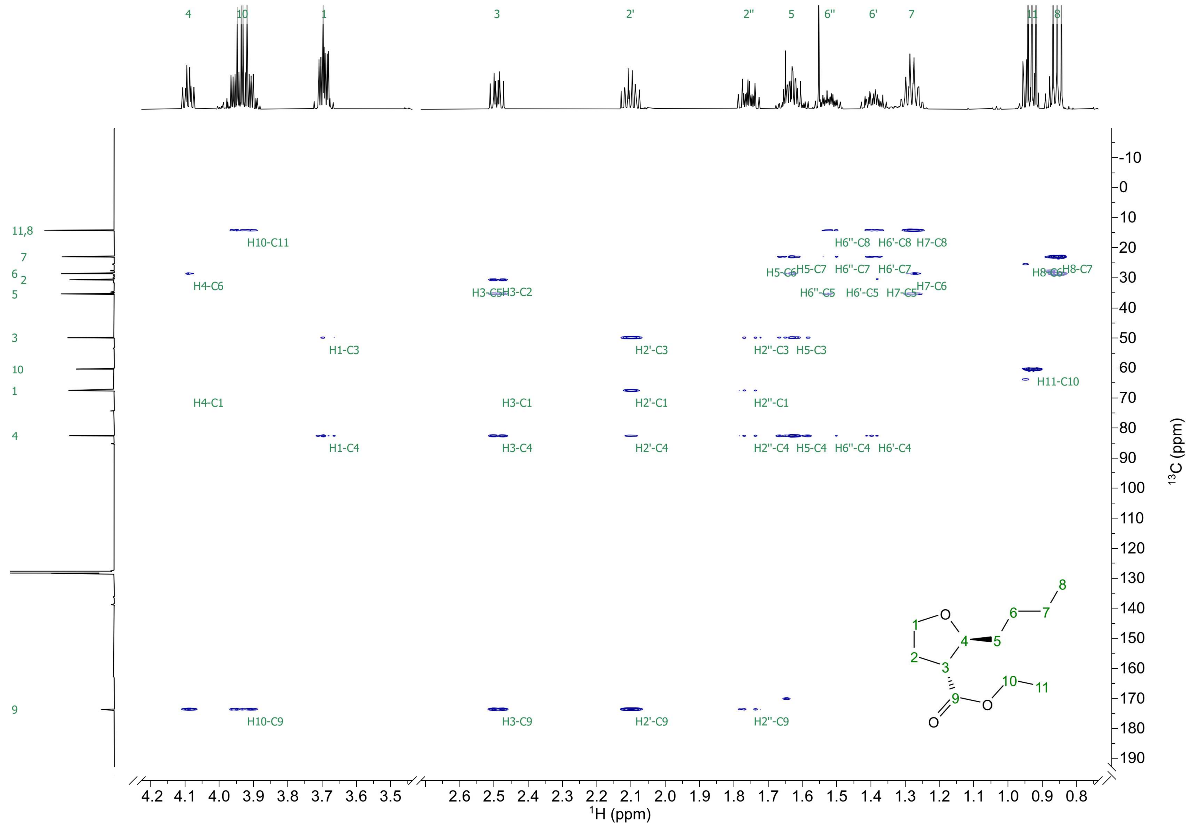

^{1}H–^{1}H COSY NMR (C_6D_6)

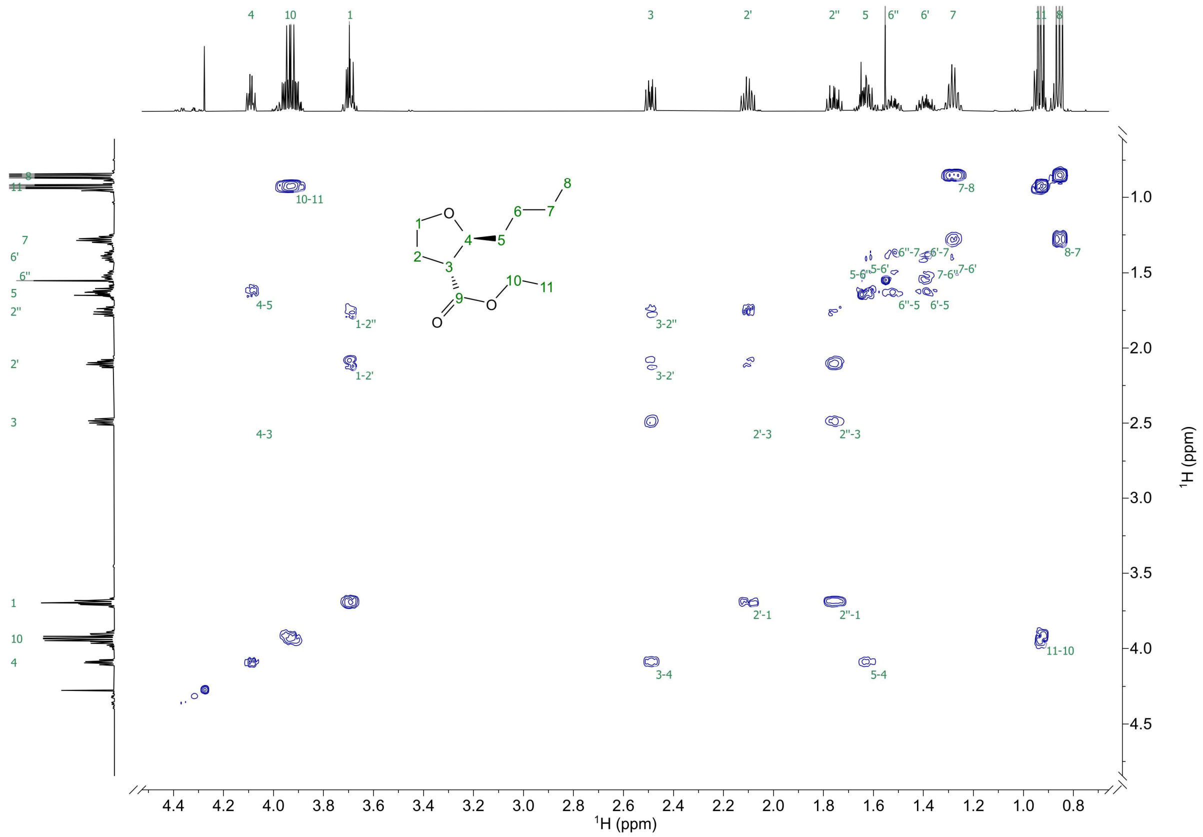

^{1}H–^{1}H NOESY NMR (C_6D_6)

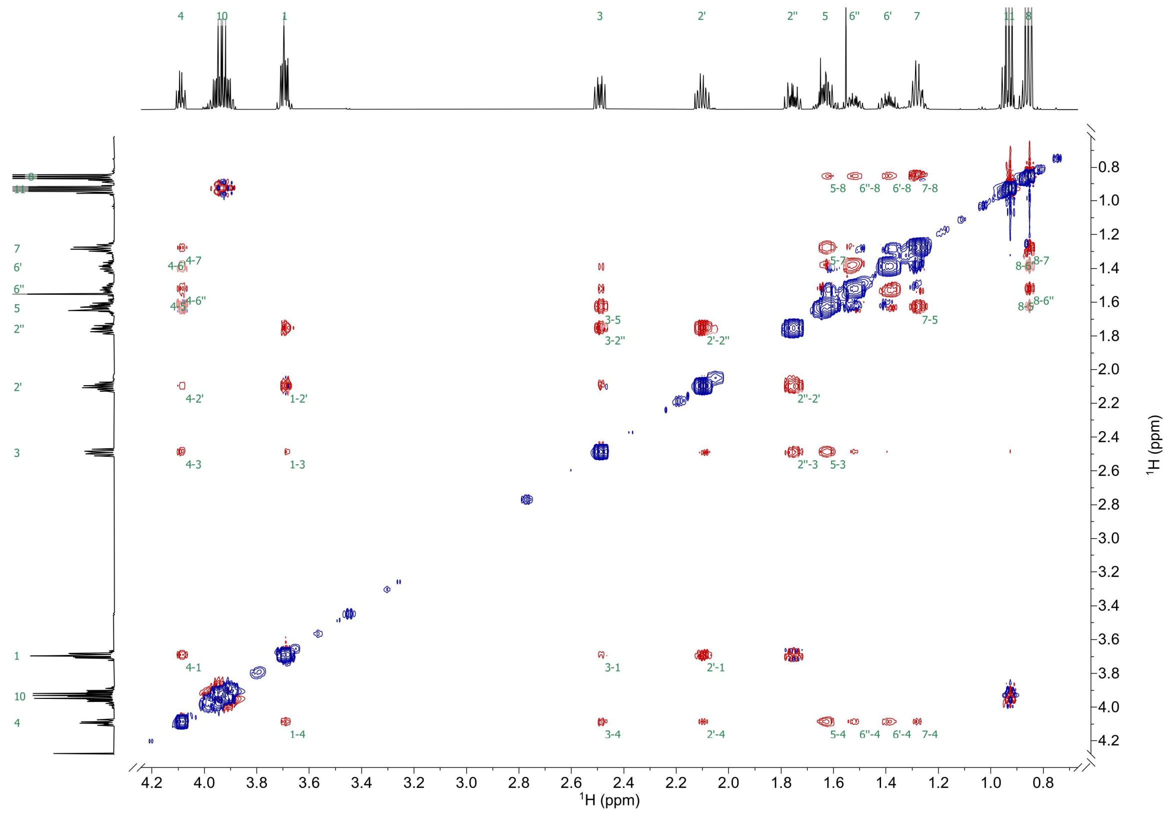

^{1}H NMR Titration (600 MHz, C_6D_6 + $CDCl_3$)

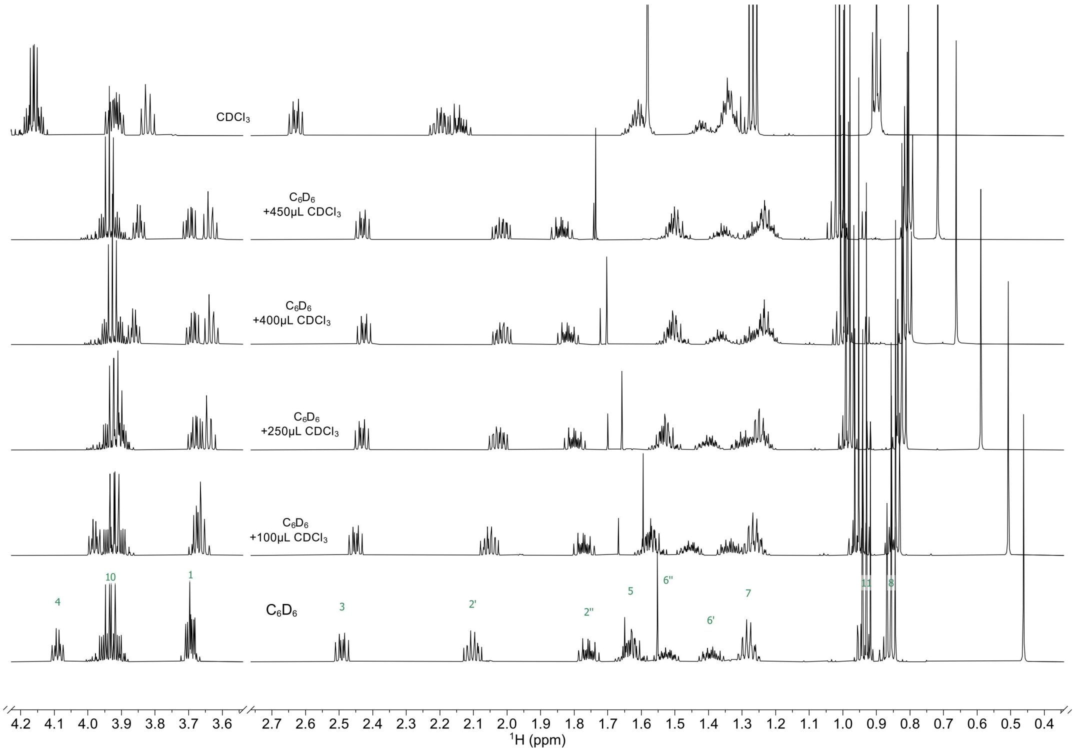

1D-selective NOESY Titration (600 MHz, C_6D_6 + $CDCl_3$)

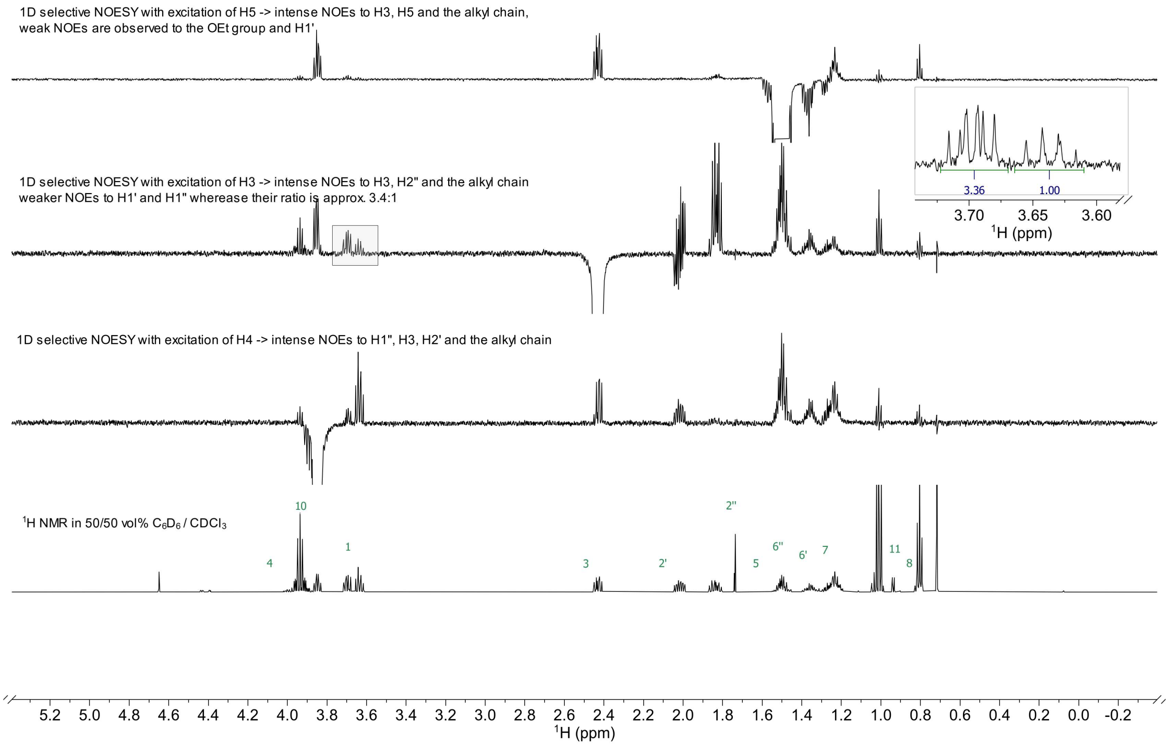

Ethyl 3-(tetrahydrofuran-2-yl)propanoate, 4l

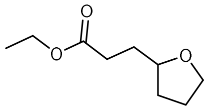

^{1}H NMR (501 MHz, $CDCl_3$)

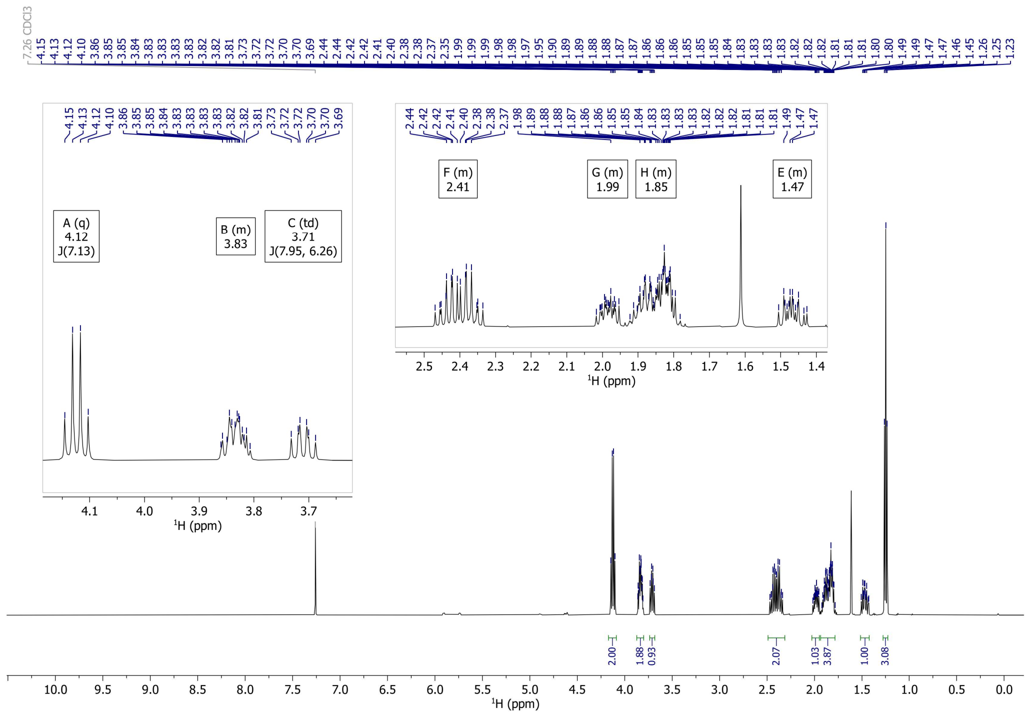

^{13}C NMR (126 MHz, $CDCl_3$)

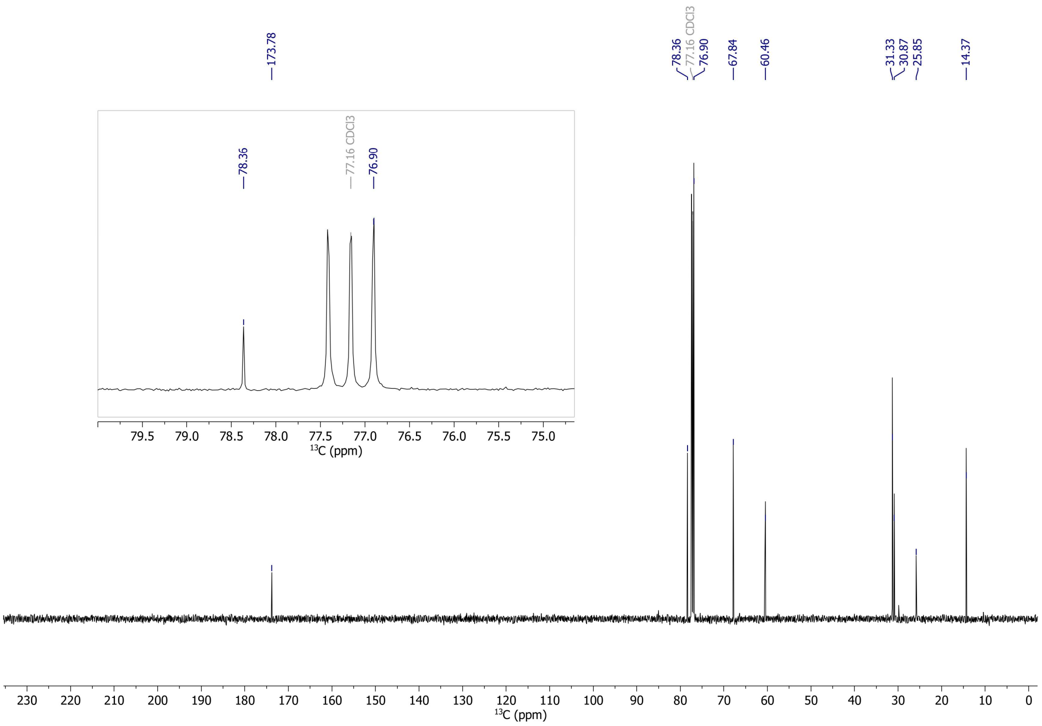

2-Pentyltetrahydrofuran, 4m

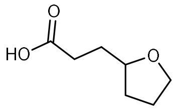

¹H NMR (501 MHz, $CDCl_3$)

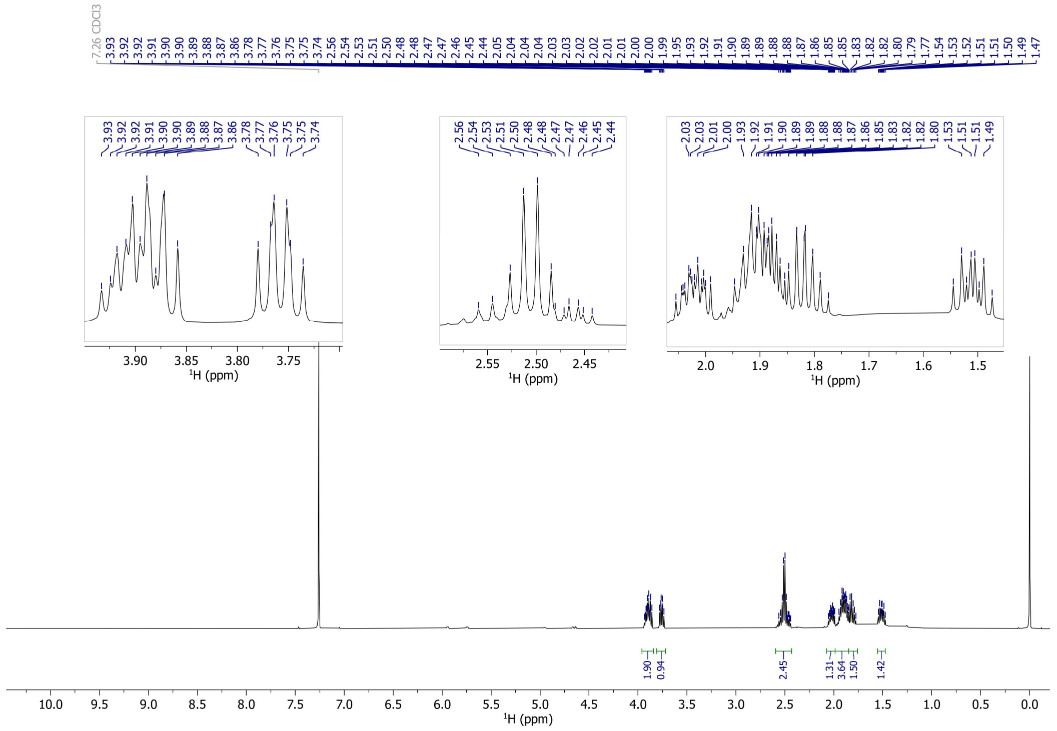

¹³C NMR (126 MHz, $CDCl_3$)

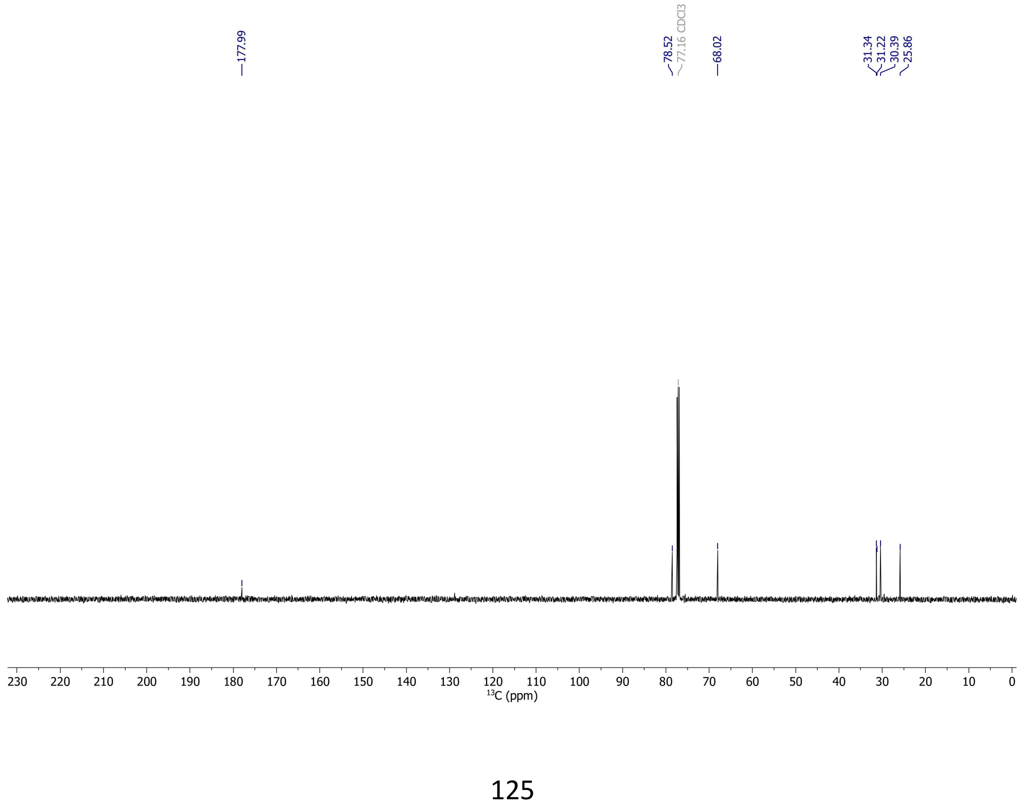

4-(Tetrahydrofuran-2-yl)benzonitrile, 4n

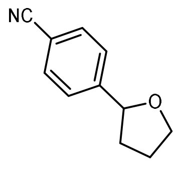

^{1}H NMR (501 MHz, $CDCl_3$)

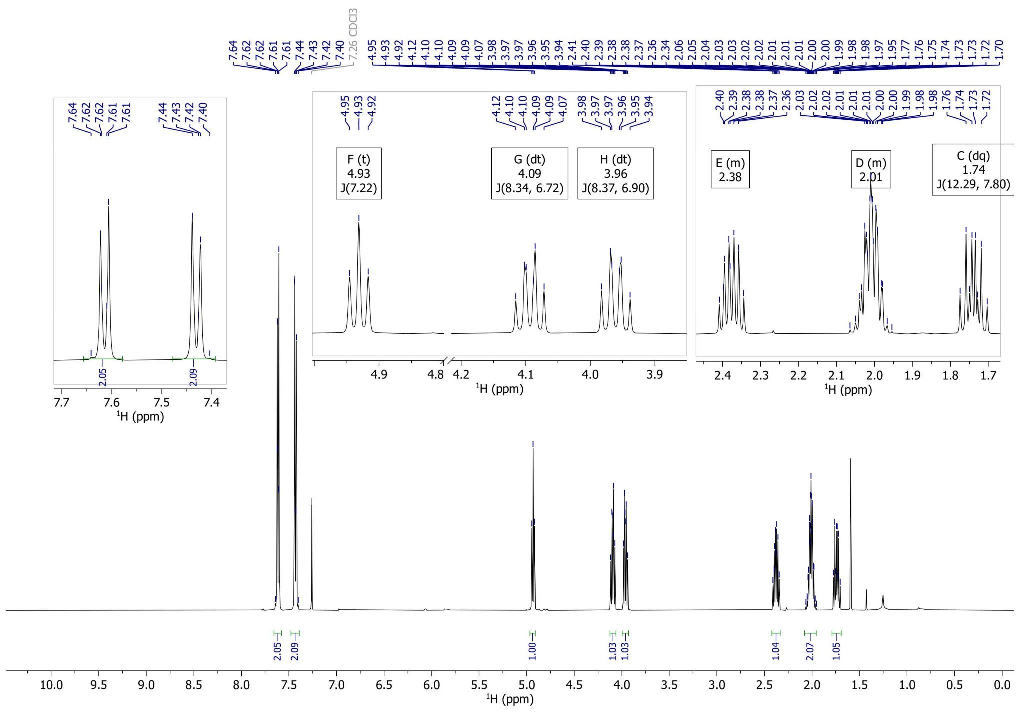

^{13}C NMR (126 MHz, $CDCl_3$)

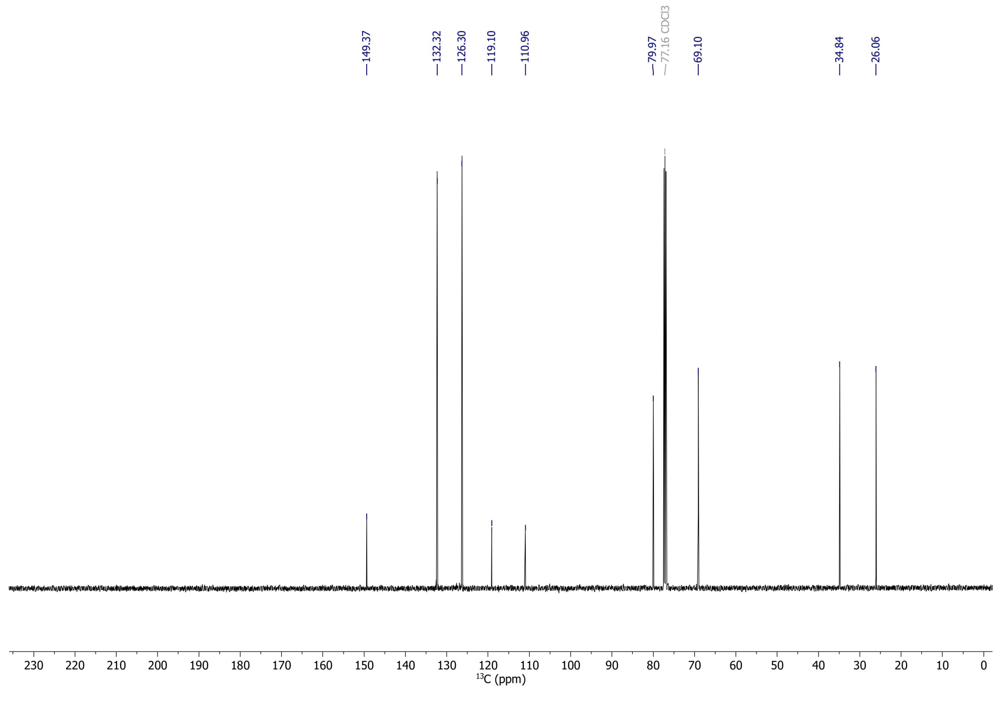

2-(4-Fluorophenyl)tetrahydrofuran, 4o

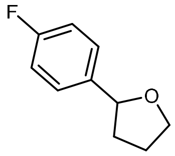

^{1}H NMR (501 MHz, $CDCl_3$)

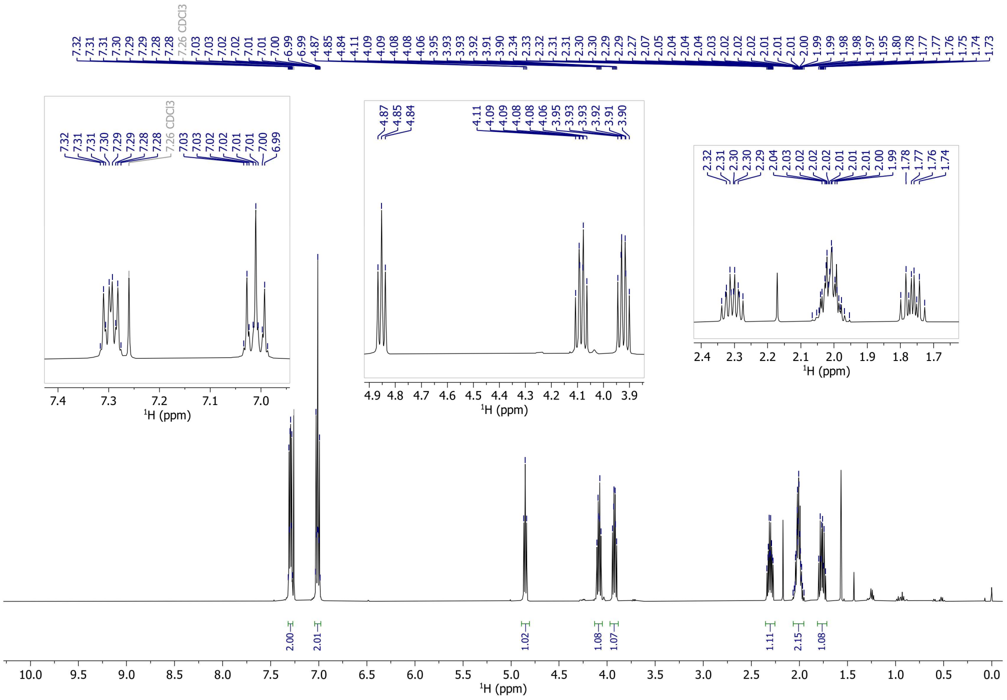

^{13}C NMR (126 MHz, $CDCl_3$)

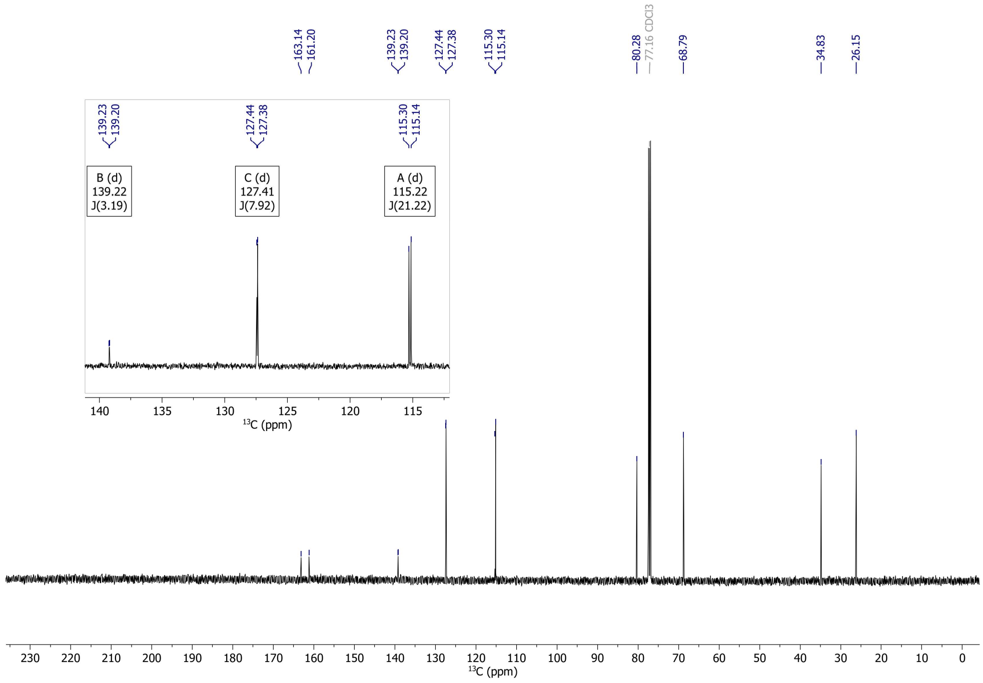

^{19}F NMR (471 MHz, $CDCl_3$)

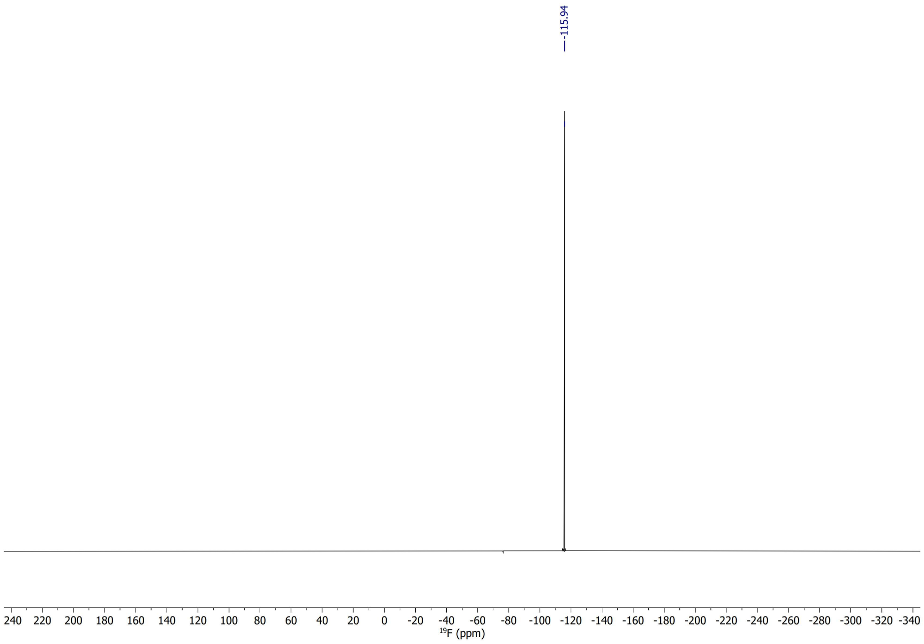

2-(3-(Trifluoromethyl)phenyl)tetrahydrofuran, 4p

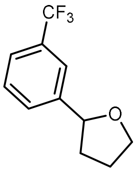

^{1}H NMR (501 MHz, $CDCl_3$)

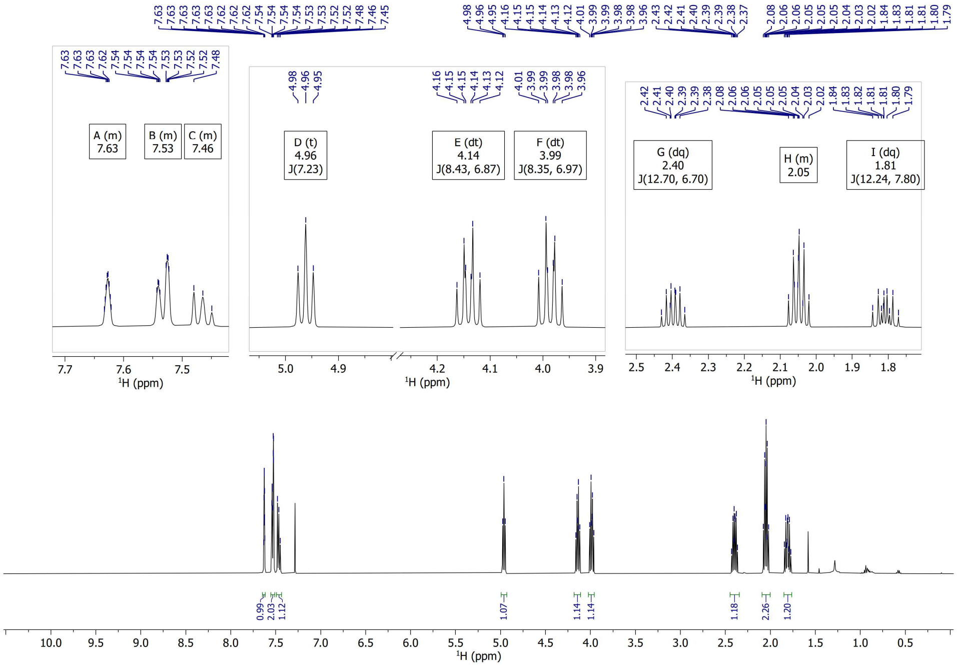

^{13}C NMR (126 MHz, $CDCl_3$)

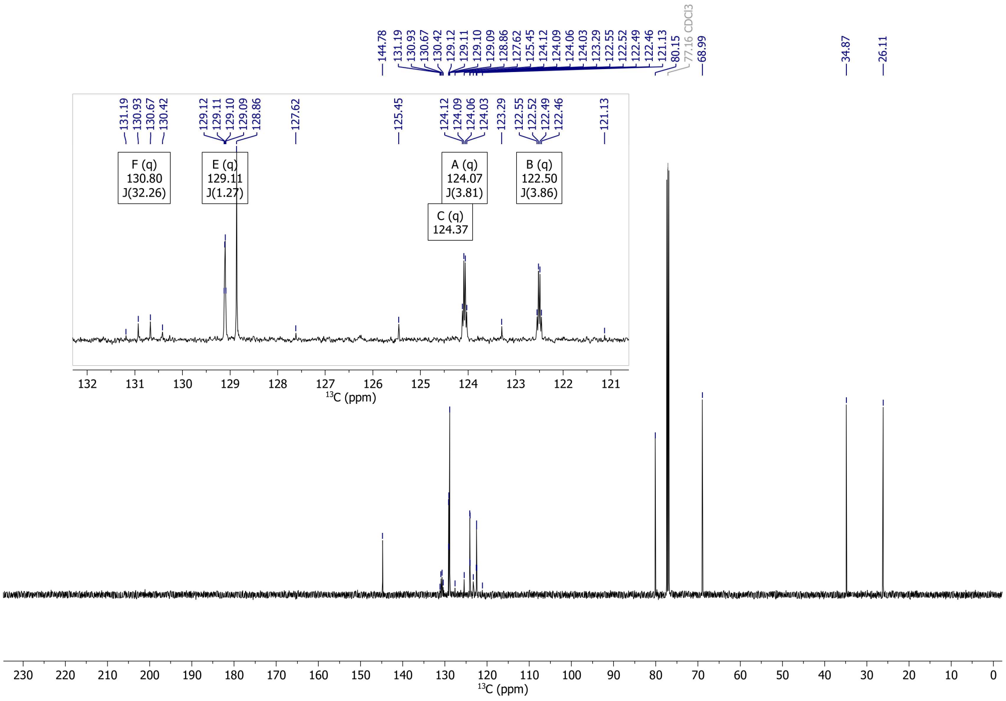

^{19}F NMR (471 MHz, $CDCl_3$)

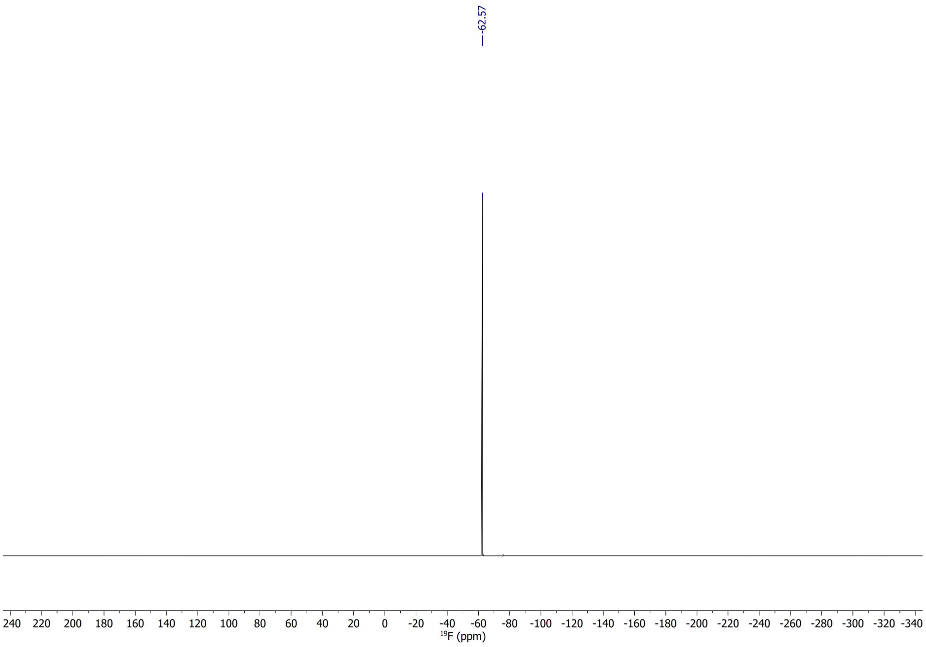

2-Phenyltetrahydrofuran, 4q

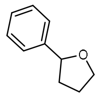

^{1}H NMR (501 MHz, $CDCl_3$)

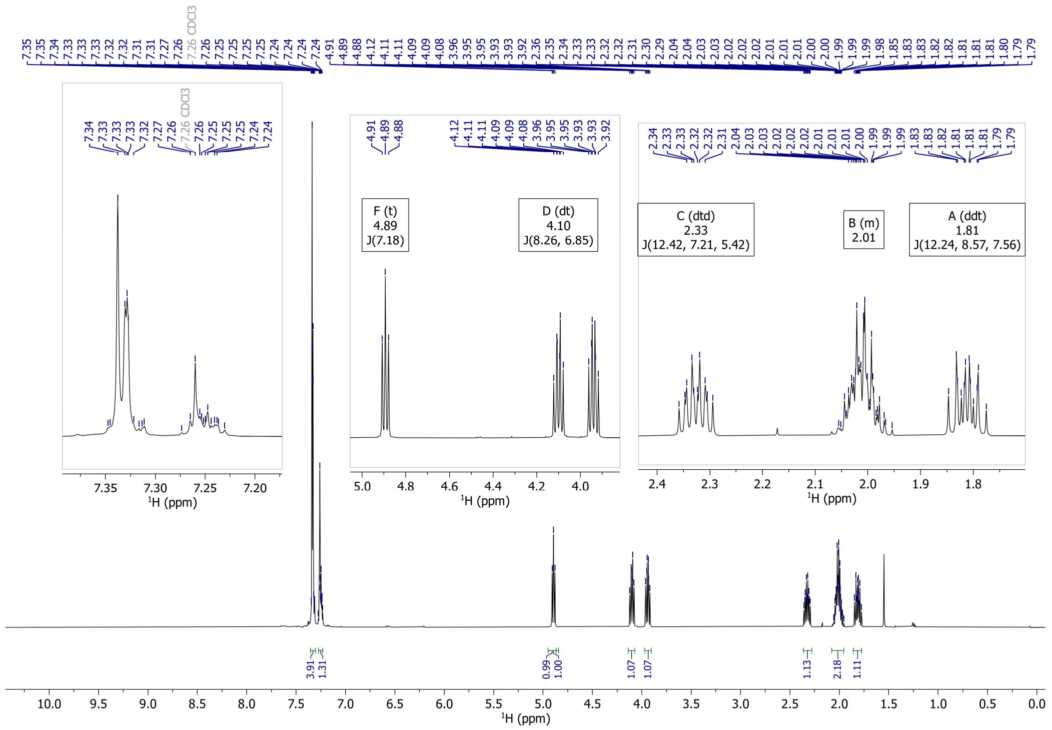

^{13}C NMR (126 MHz, $CDCl_3$)

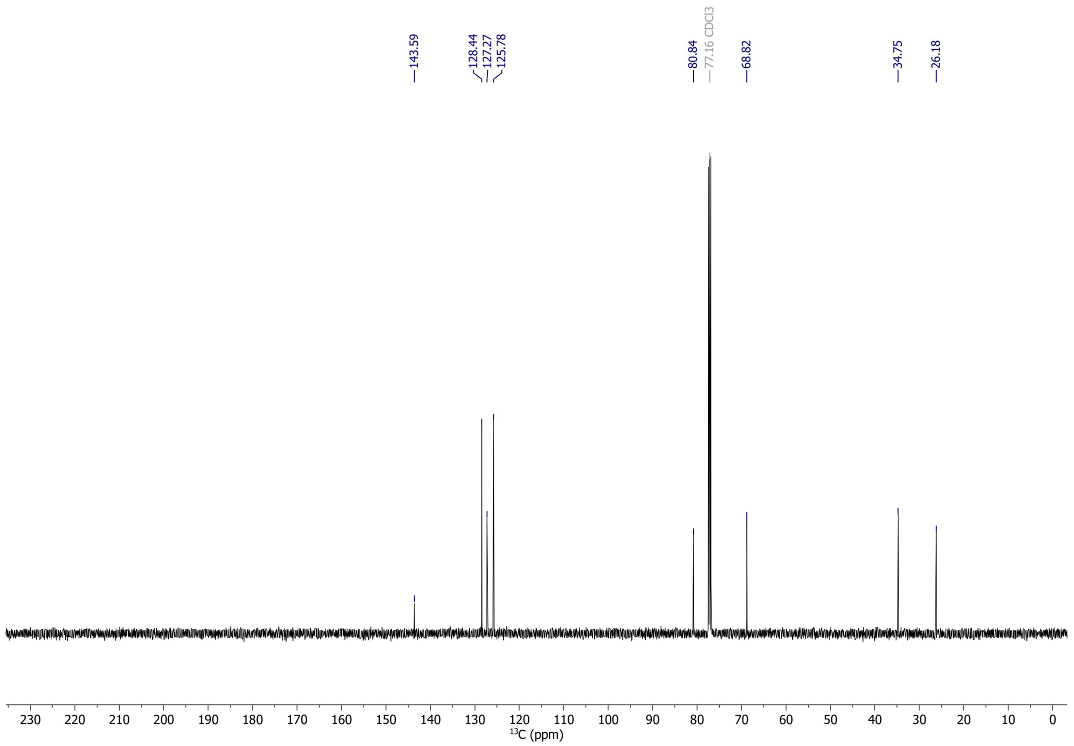

γ-Valerolactone, 4t

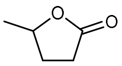

^{1}H NMR (501 MHz, $CDCl_3$)

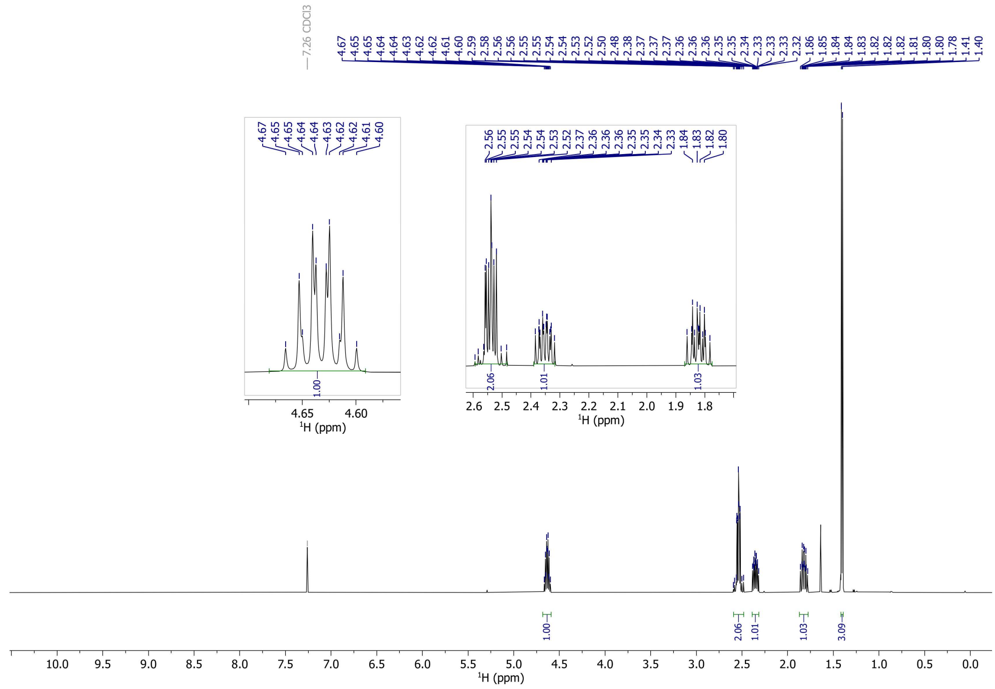

^{13}C NMR (126 MHz, $CDCl_3$)

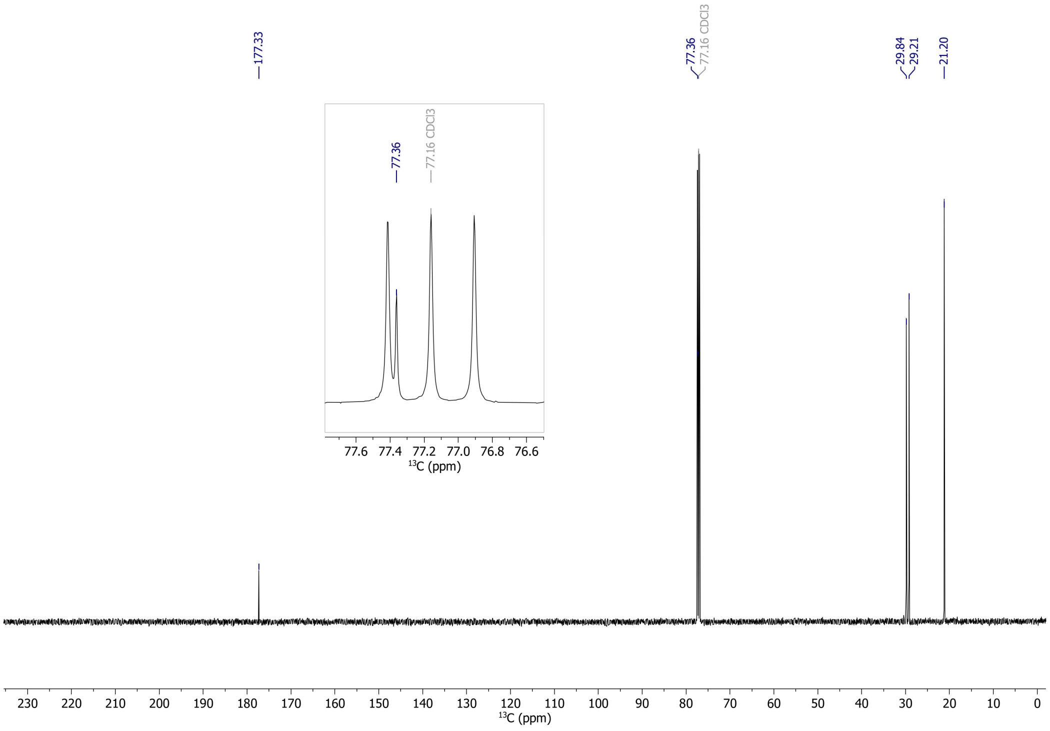

2,3-Dihydrobenzofuran 4u

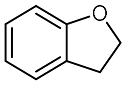

^{1}H NMR (600 MHz, $CDCl_3$)

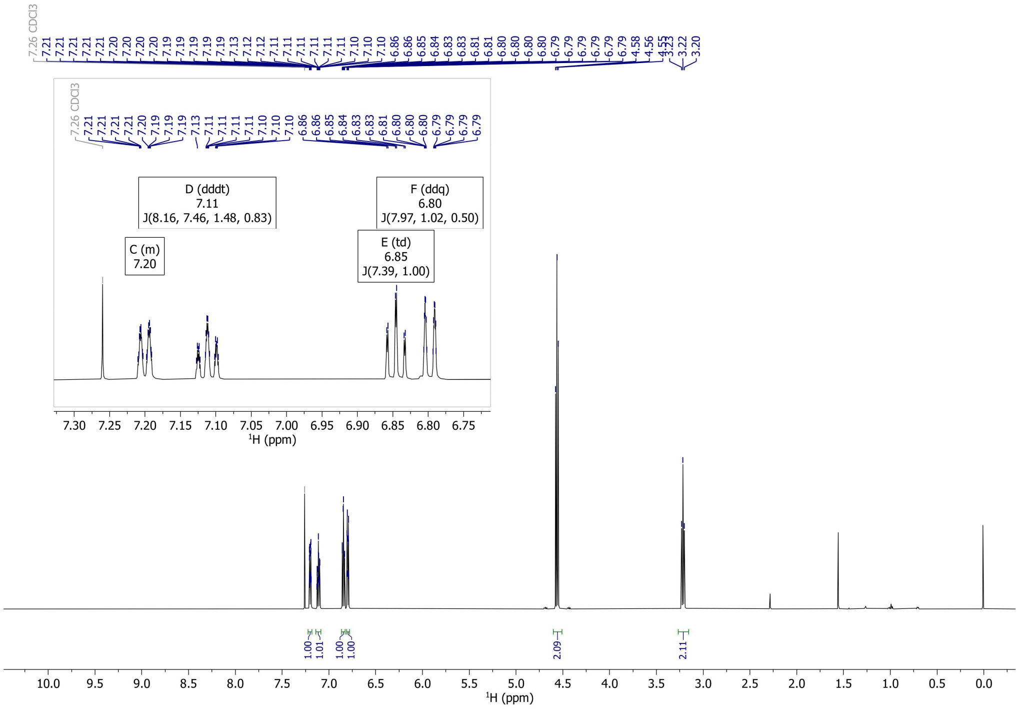

^{13}C NMR (150 MHz, $CDCl_3$)

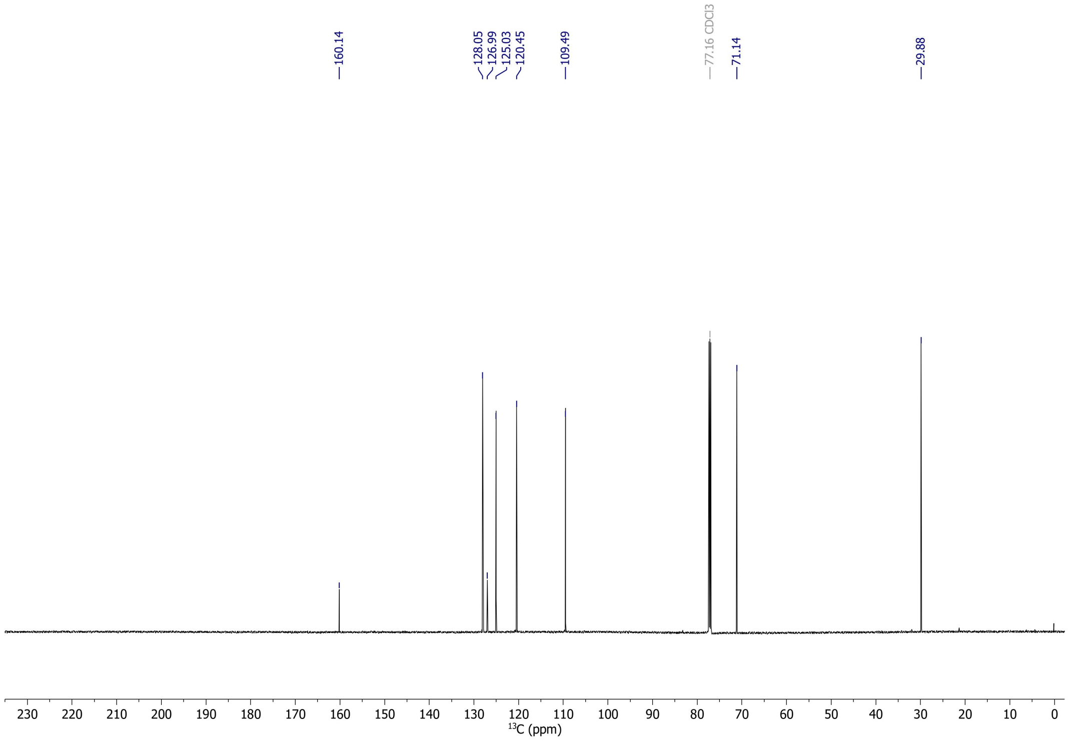

4-Phenylbutanol

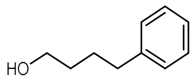

^{1}H NMR (501 MHz, $CDCl_3$)

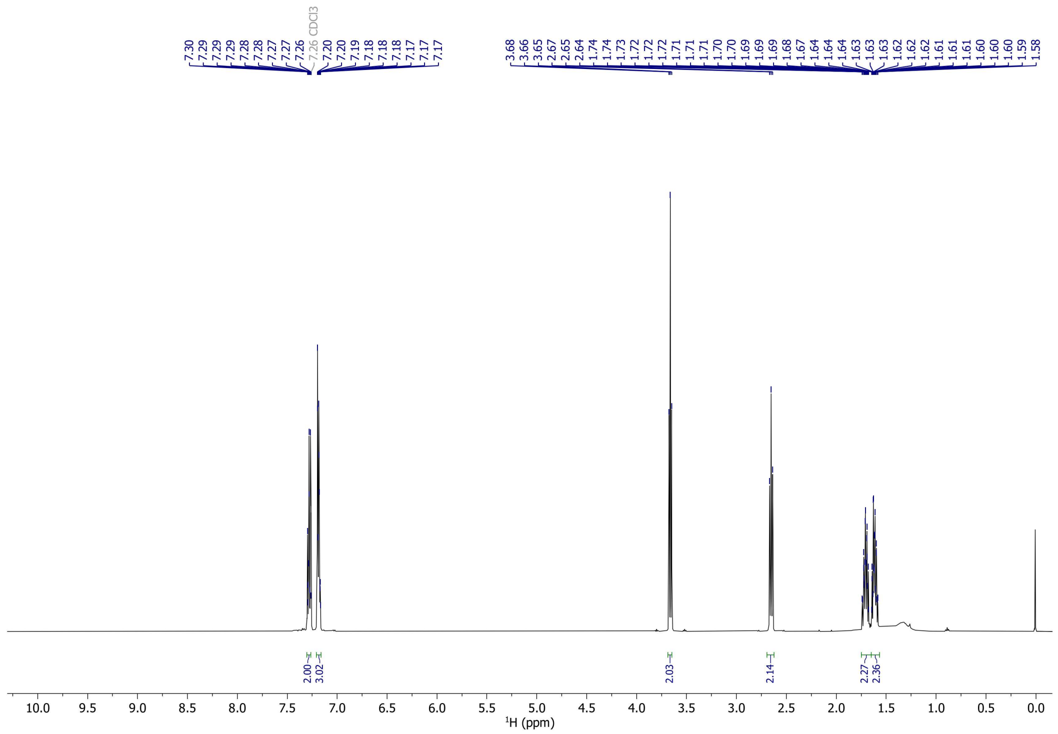

^{13}C NMR (126 MHz, $CDCl_3$)

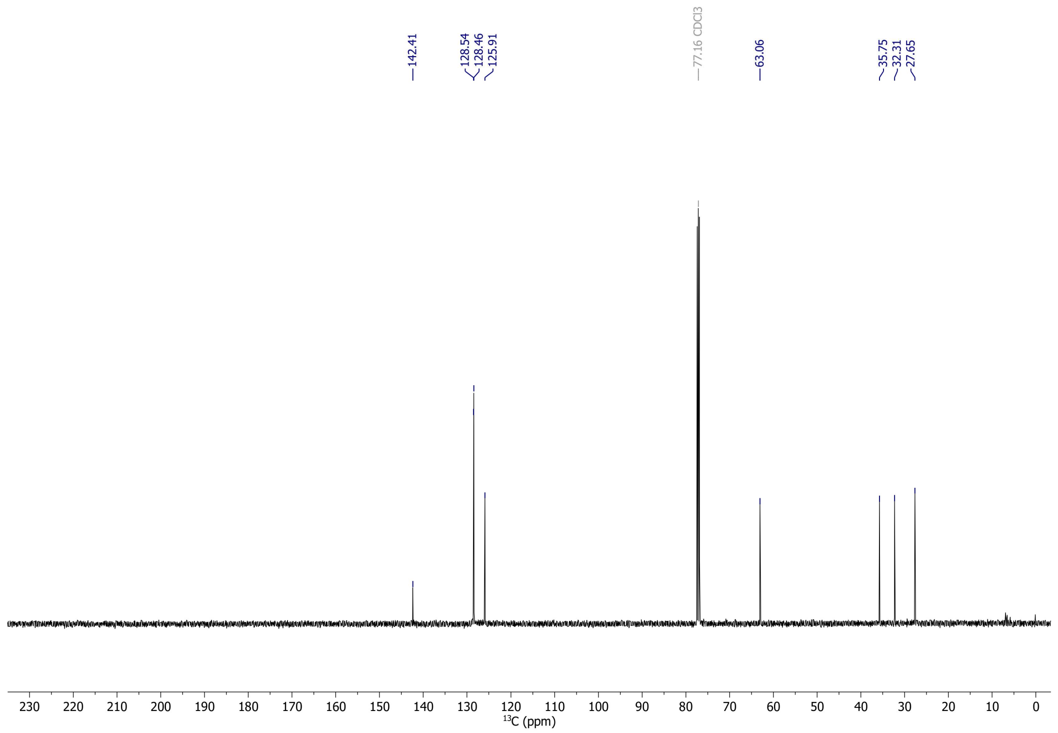

14. Appendix

^{1}H NMR Yields

3-Methyl-2,5-dihydrofuran, 2l

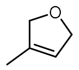

^{1}H NMR (501 MHz, $CDCl_3$)

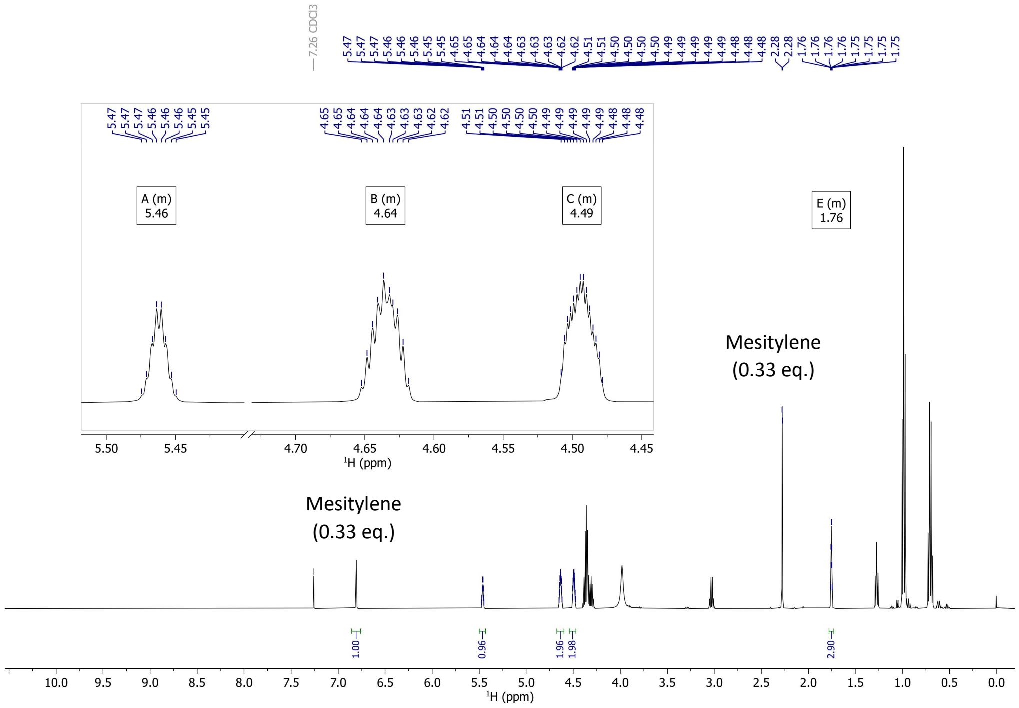

^{13}C NMR (126 MHz, $CDCl_3$)

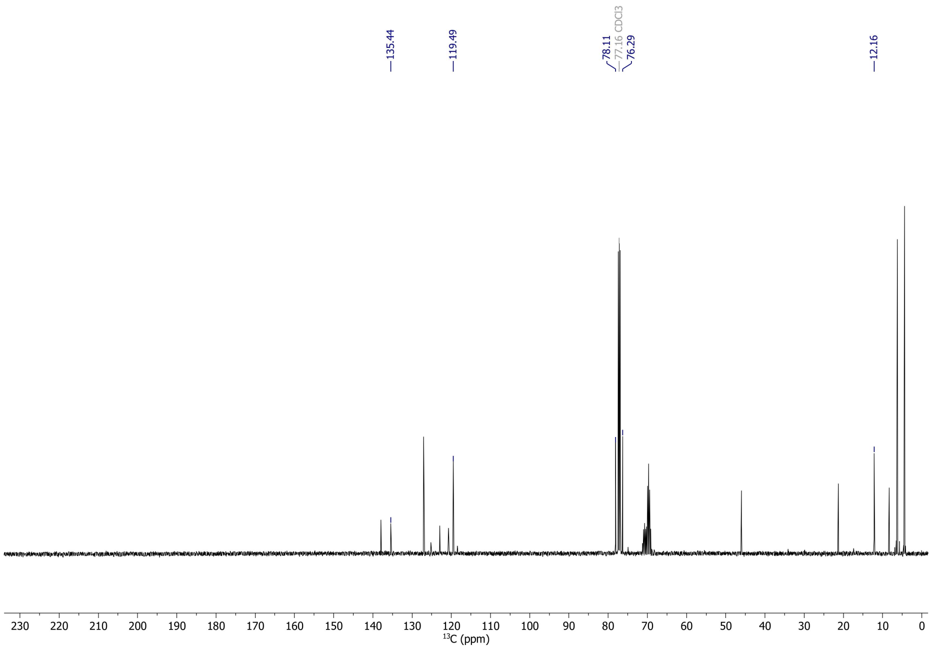

^{13}C NMR peaks were assigned based on HMBC and HSQC spectra:

^{1}H-^{13}C-HSQC (500 MHz, 126 MHz, $CDCl_3$)

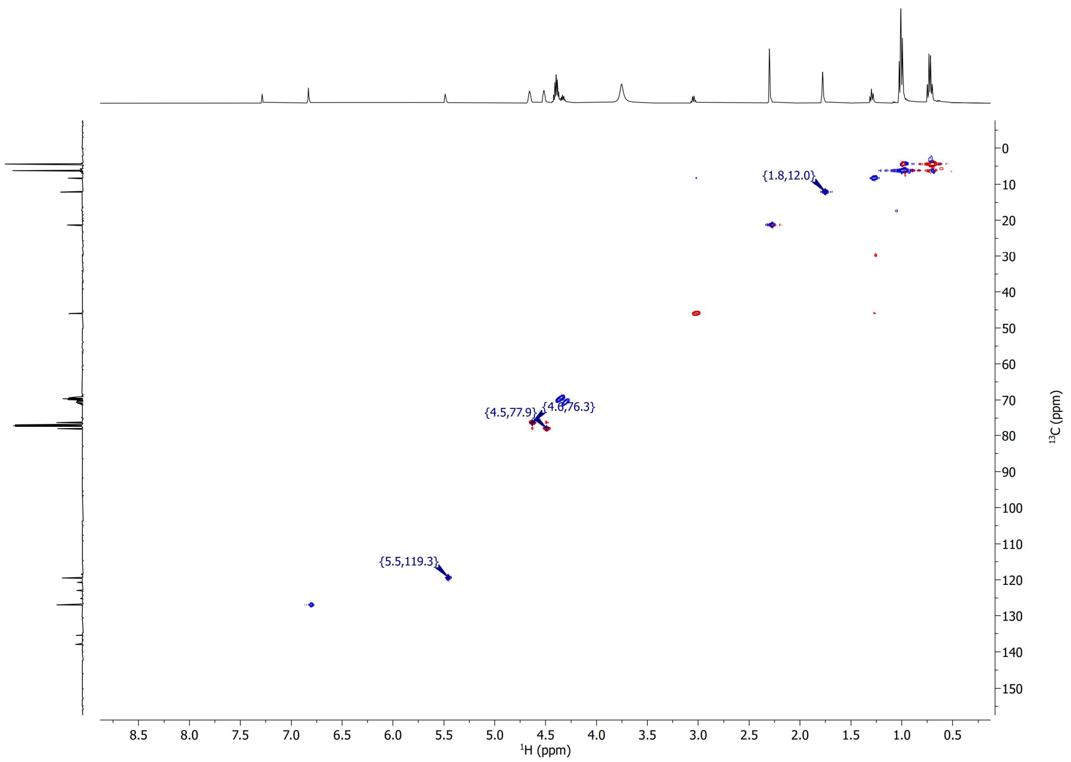

^{1}H-^{13}C-HMBC (500 MHz, 126 MHz, $CDCl_3$)

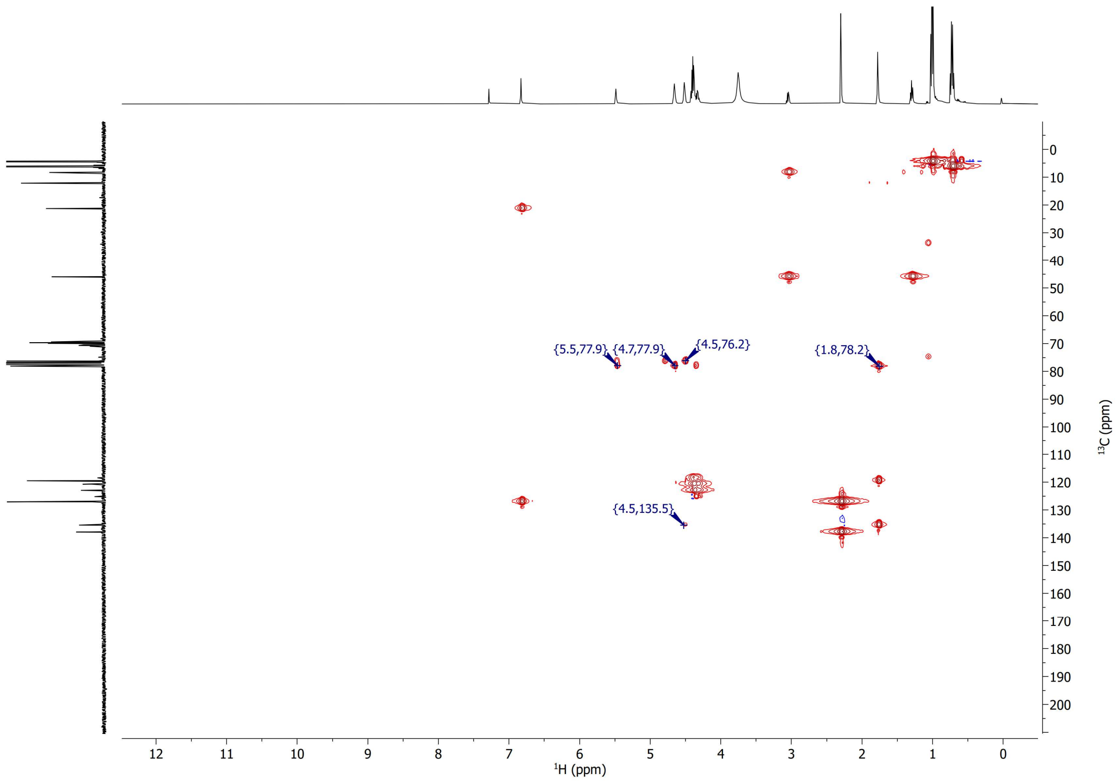

3-(Chloromethyl)-2,5-dihydrofuran, 2m

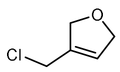

^{1}H NMR (600 MHz, CD_2Cl_2)

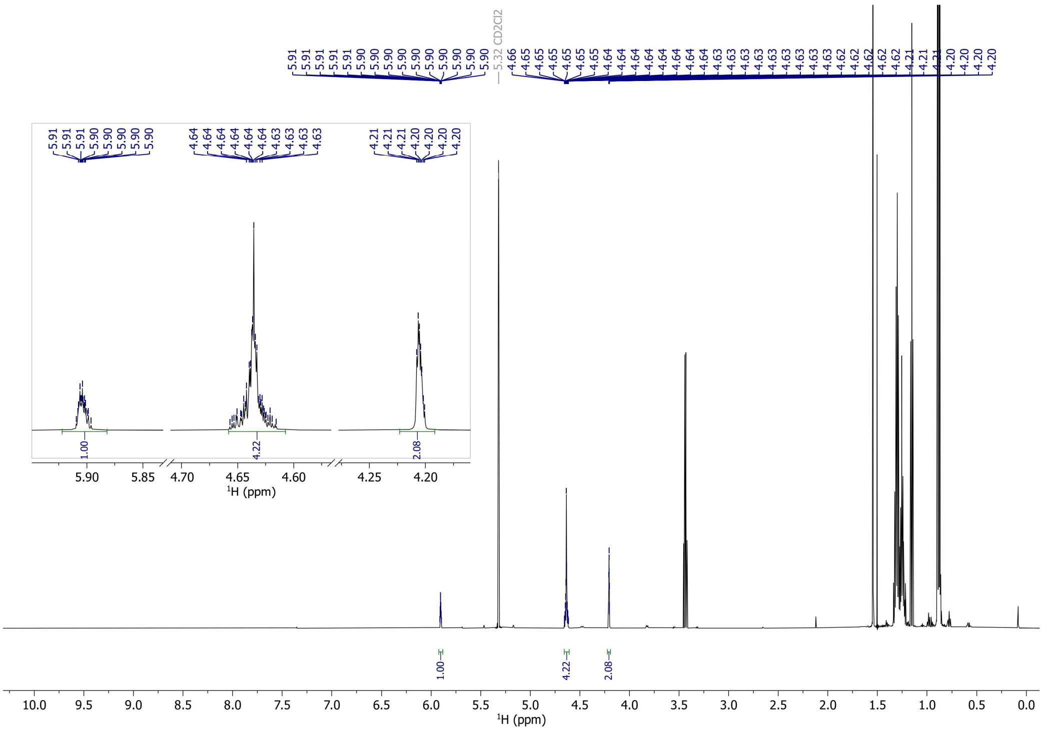

^{13}C NMR (151 MHz, CD_2Cl_2)

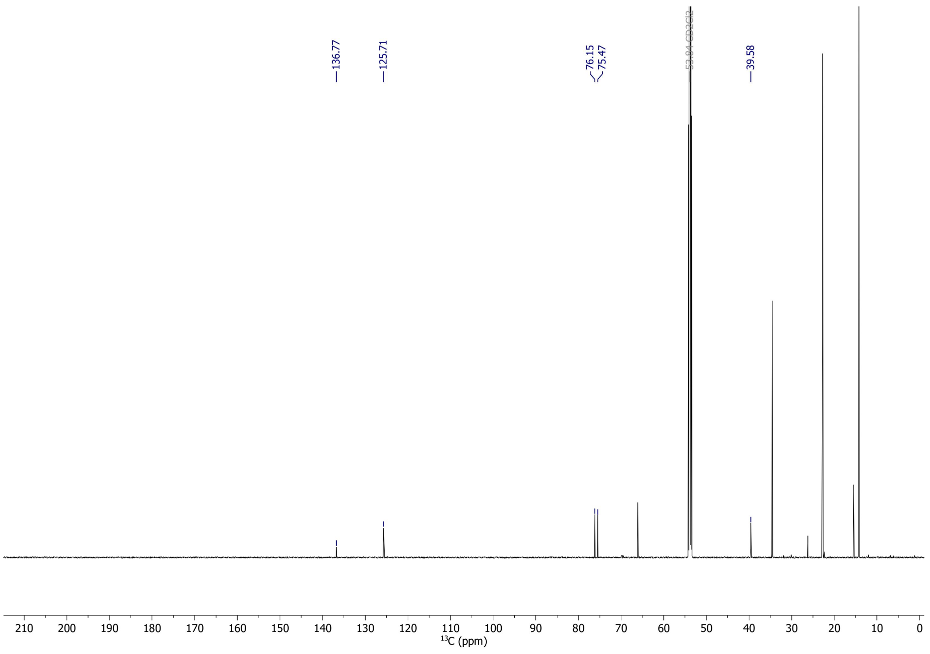

^{1}H-^{13}C-HSQC (500 MHz, 126 MHz, CD_2Cl_2)

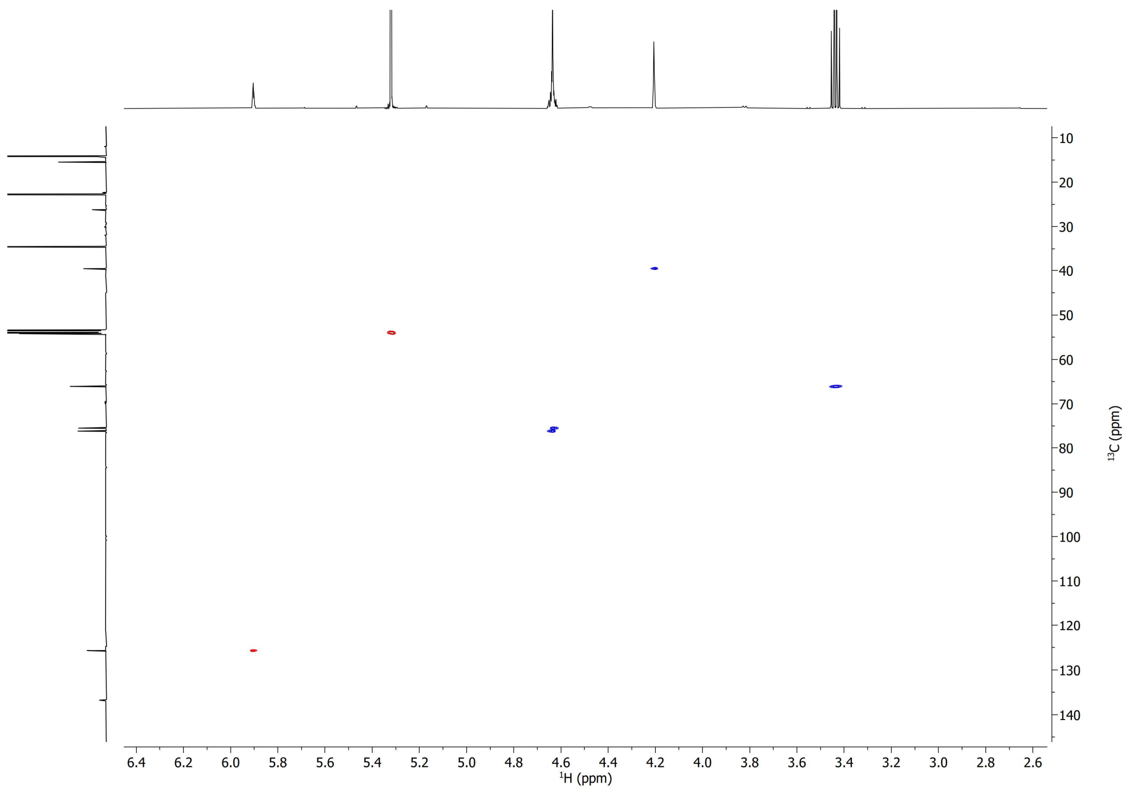

^{1}H-^{1}H-COSY (600 MHz, CD_2Cl_2)

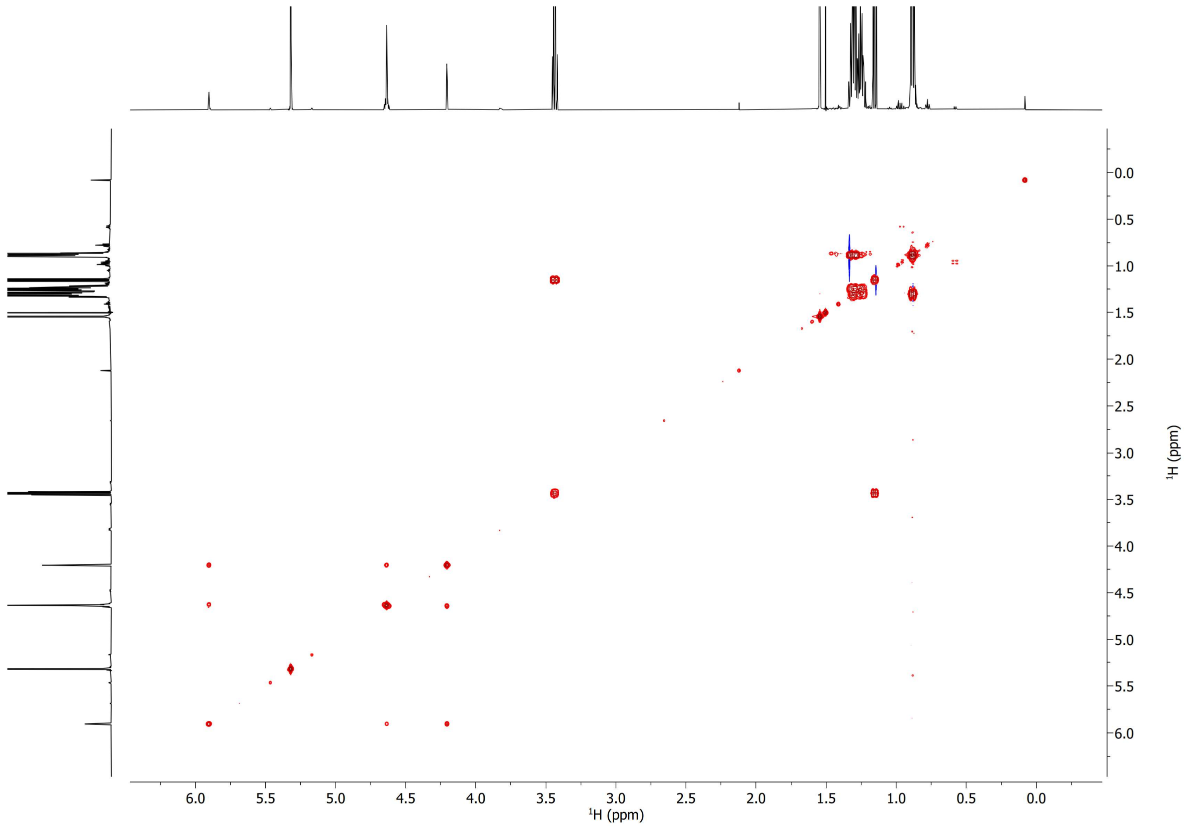

2,5-Dihydrofuran, 2u

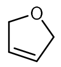

^{1}H NMR (501 MHz, $CDCl_3$)

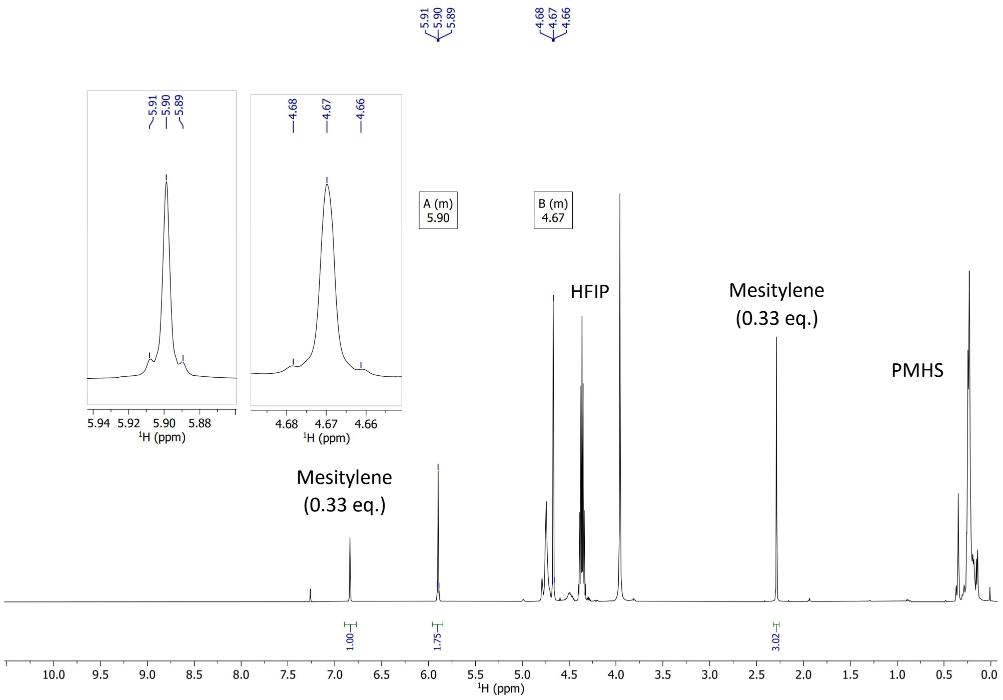

^{13}C NMR (126 MHz, $CDCl_3$)

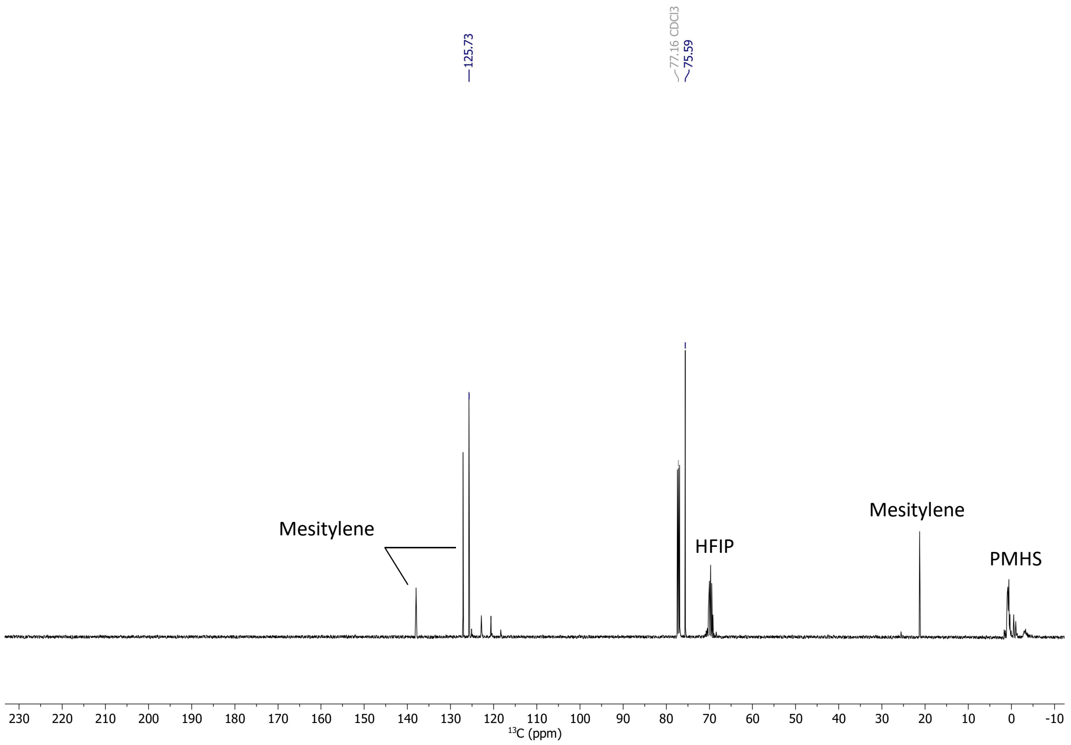

^{13}C NMR peaks were assigned based on HSQC spectra:

^{1}H-^{13}C-HSQC (500 MHz, 126 MHz, $CDCl_3$)

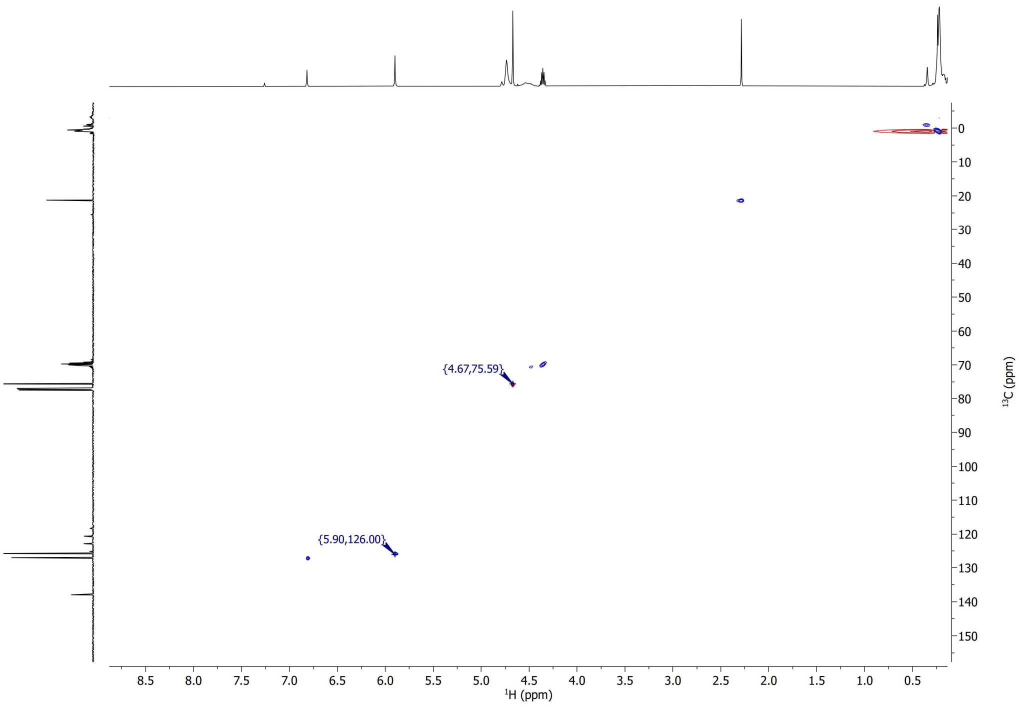

^{1}H-^{1}H-COSY (500 MHz, $CDCl_3$)

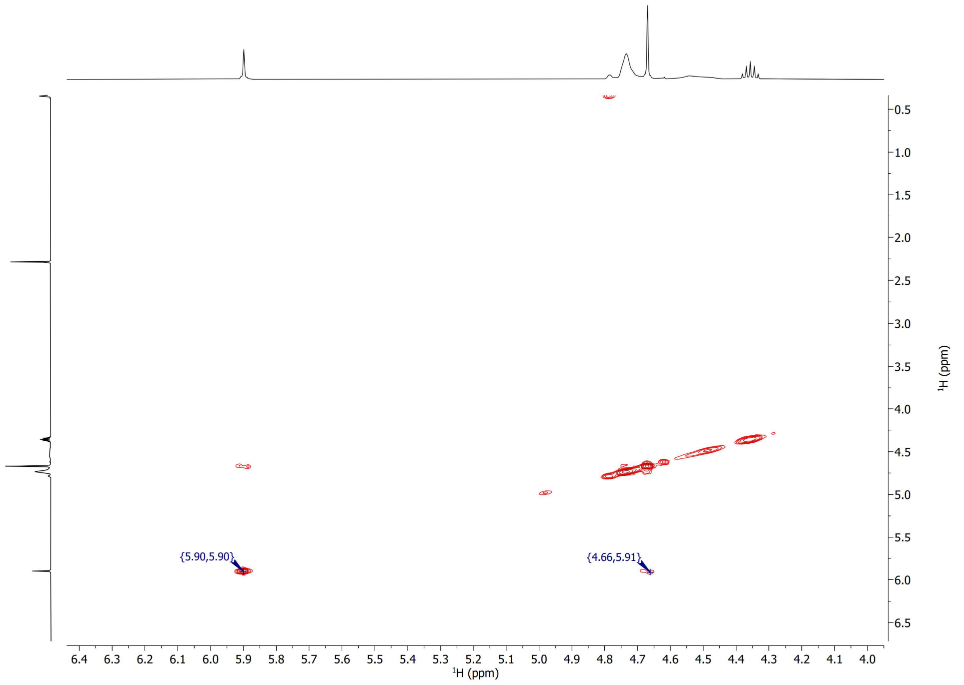

2-Methyltetrahydrofuran from 2-methylfuran, 4i

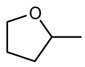

^{1}H NMR (501 MHz, $CDCl_3$)

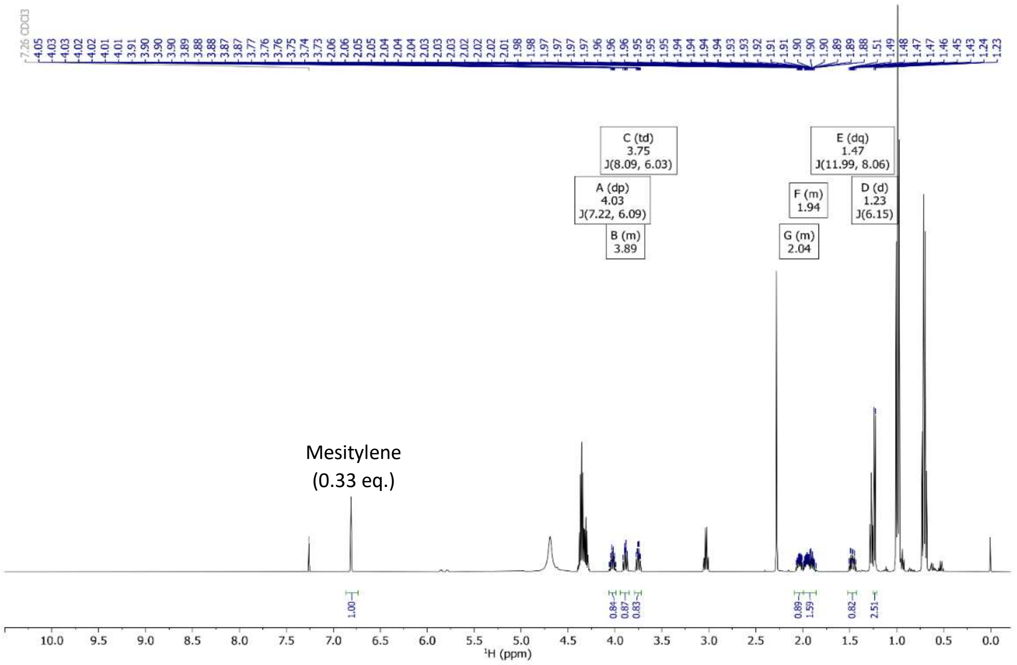

2-Methyltetrahydrofuran from Furfural, 4r

^{1}H NMR (501 MHz, $CDCl_3$)

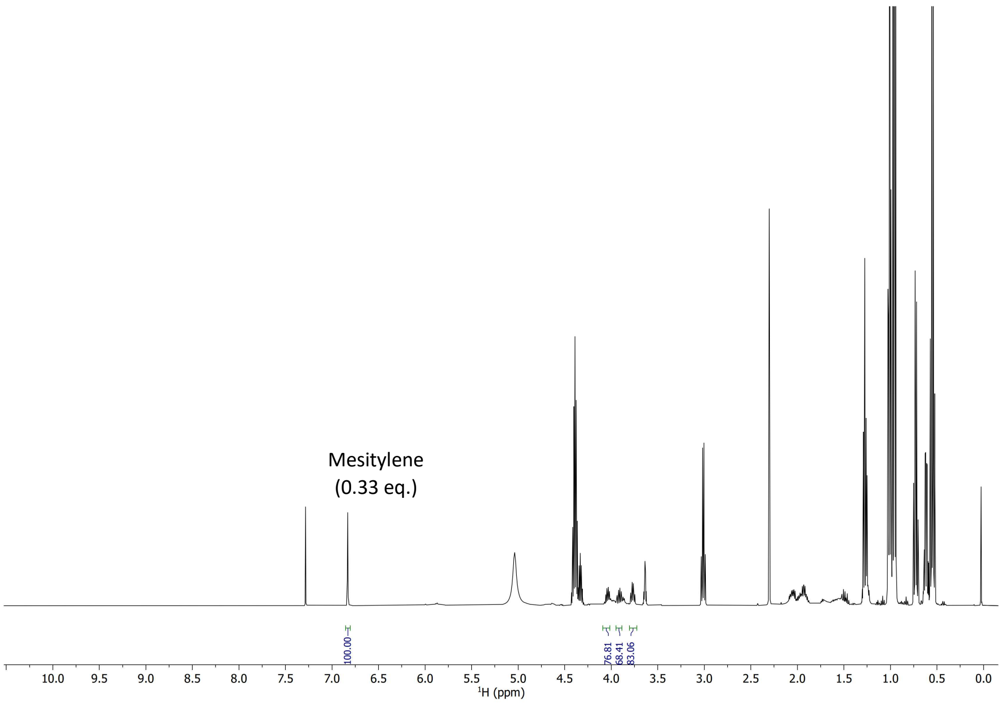

cis/*trans*-2,5-Dimethyltetrahydrofuran from 2,5-dimethylfuran, 4s

^{1}H NMR (501 MHz, $CDCl_3$)

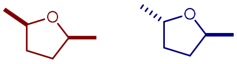

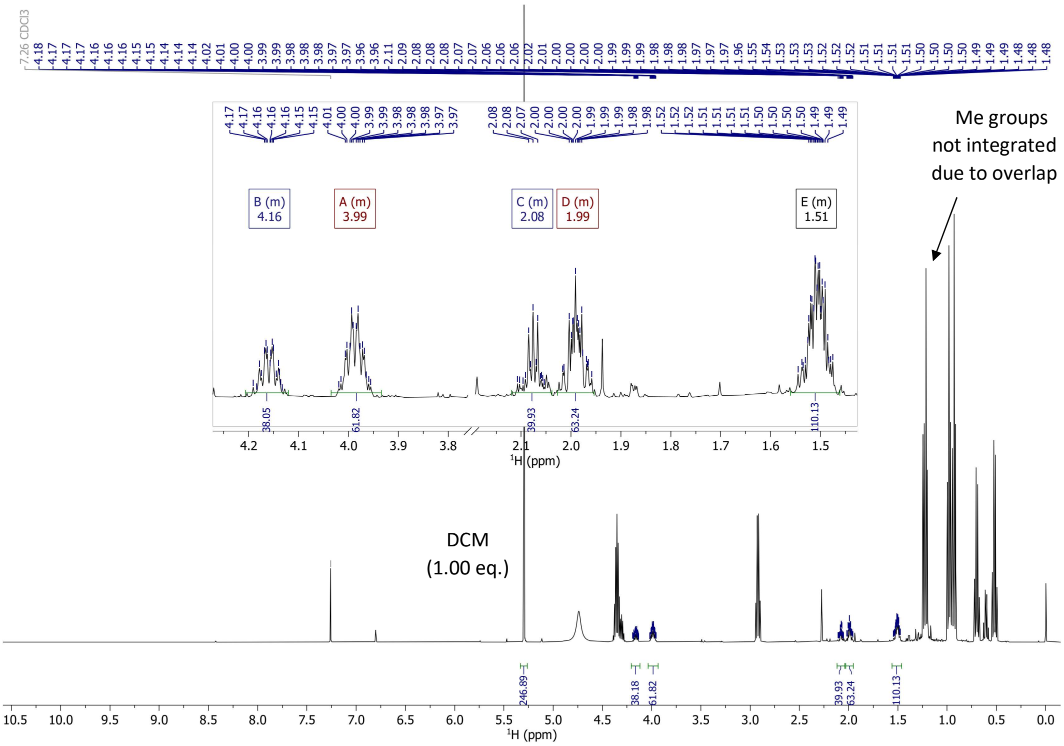

cis/*trans*-2,5-Dimethyltetrahydrofuran from Hydroxymethylfurfural (HMF), 4s

^{1}H NMR (500 MHz, $CDCl_3$)

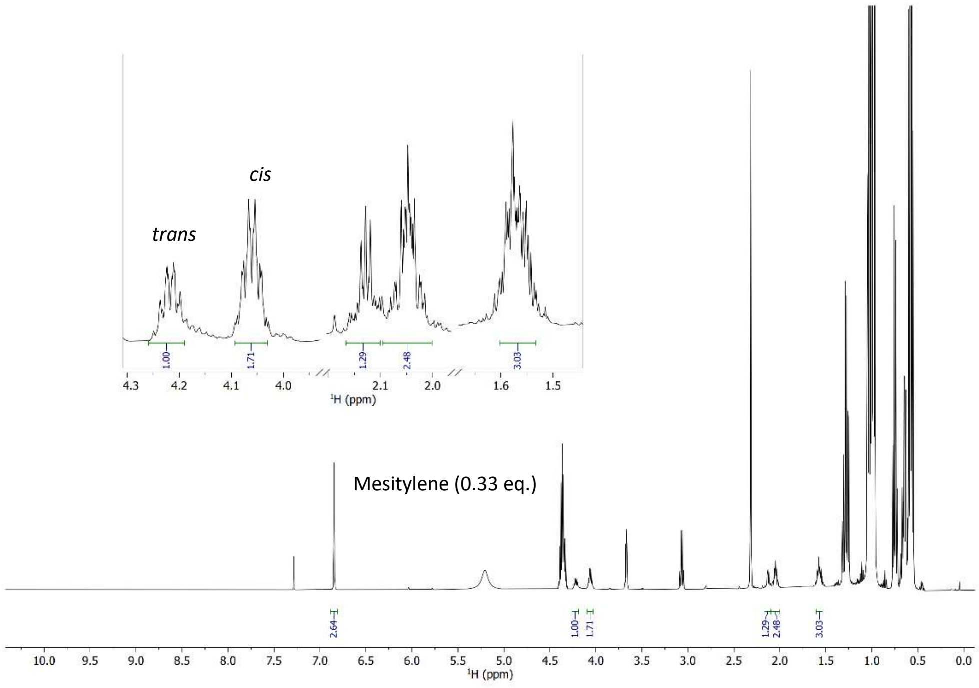

NMR Spectra of Dimeric Product III

^{1}H NMR (600 MHz, $CDCl_3$)

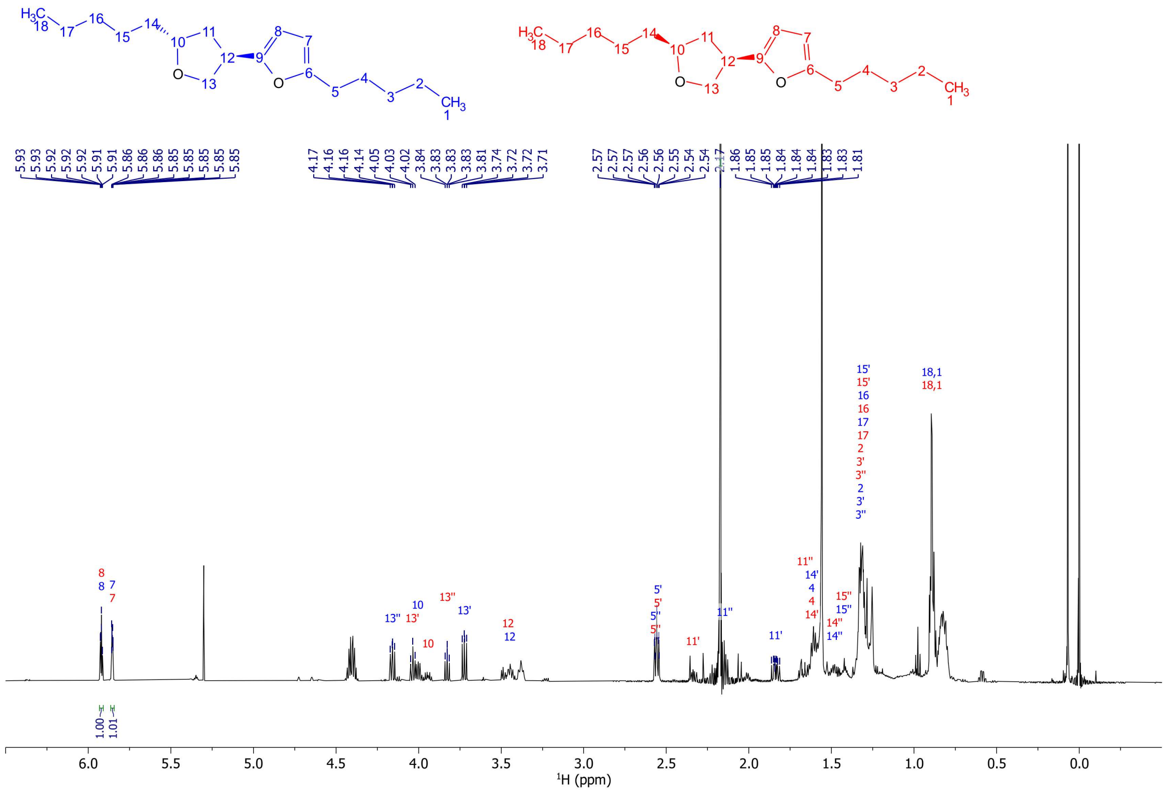

^{13}C NMR (151 MHz, $CDCl_3$)

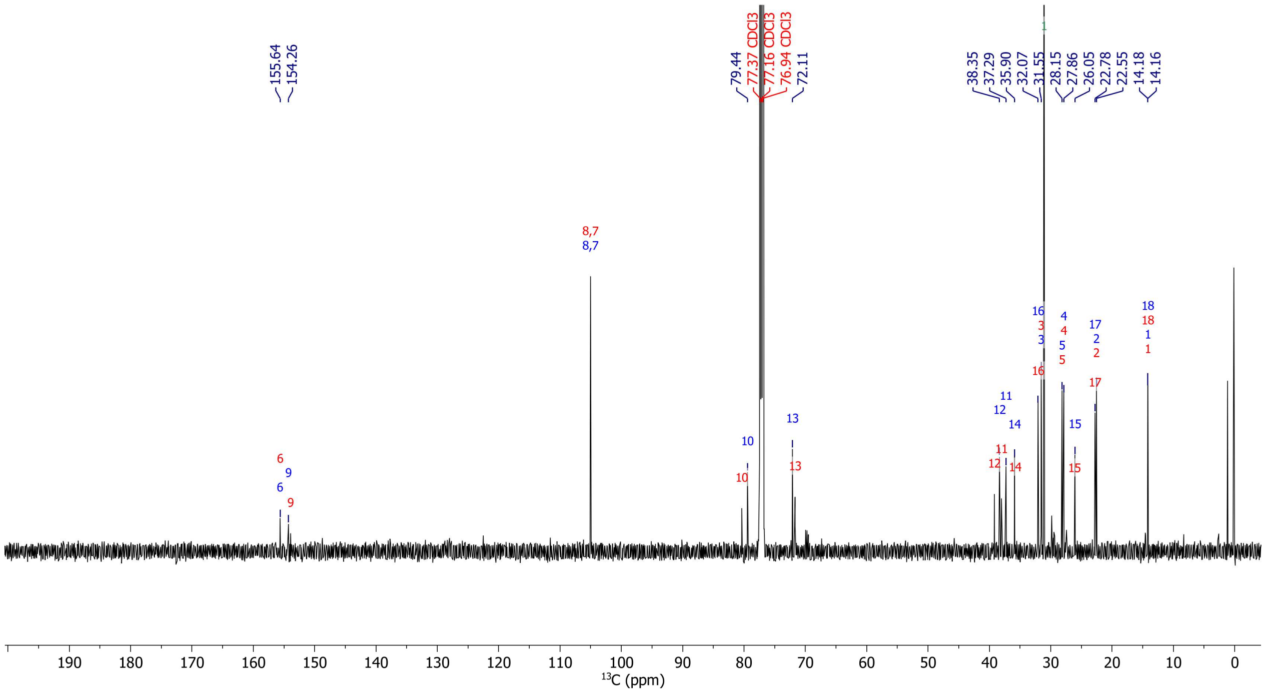

^{1}H-^{1}H-NOESY (600 MHz, CDCl$_3$)

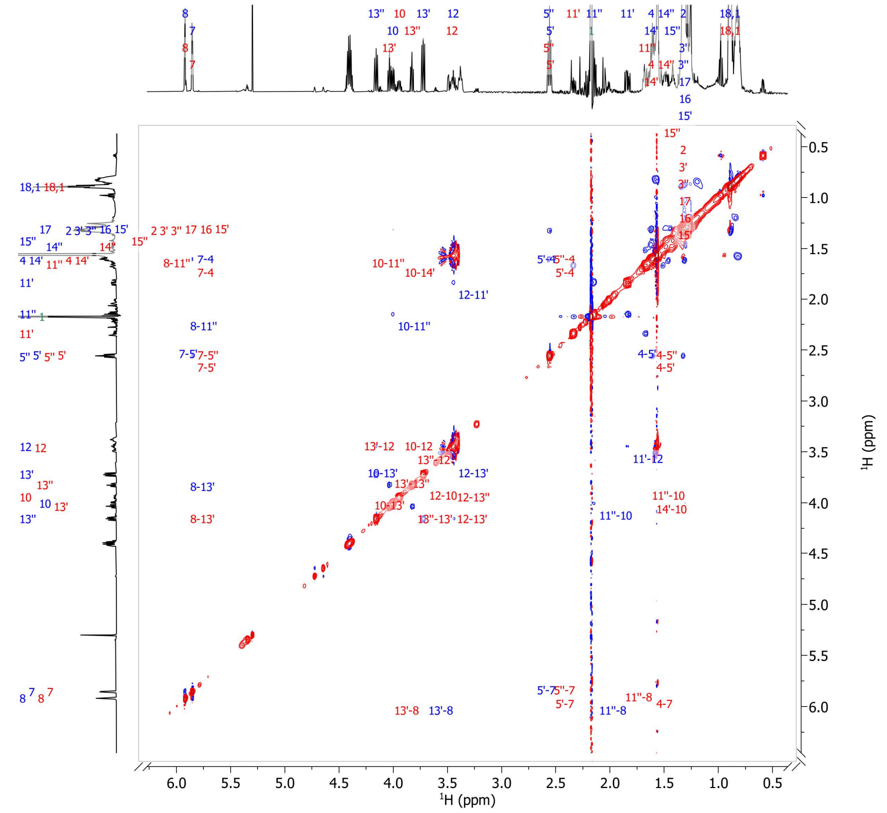

^{1}H-^{13}C-HSQC (600 MHz, 151 MHz, CDCl$_3$)

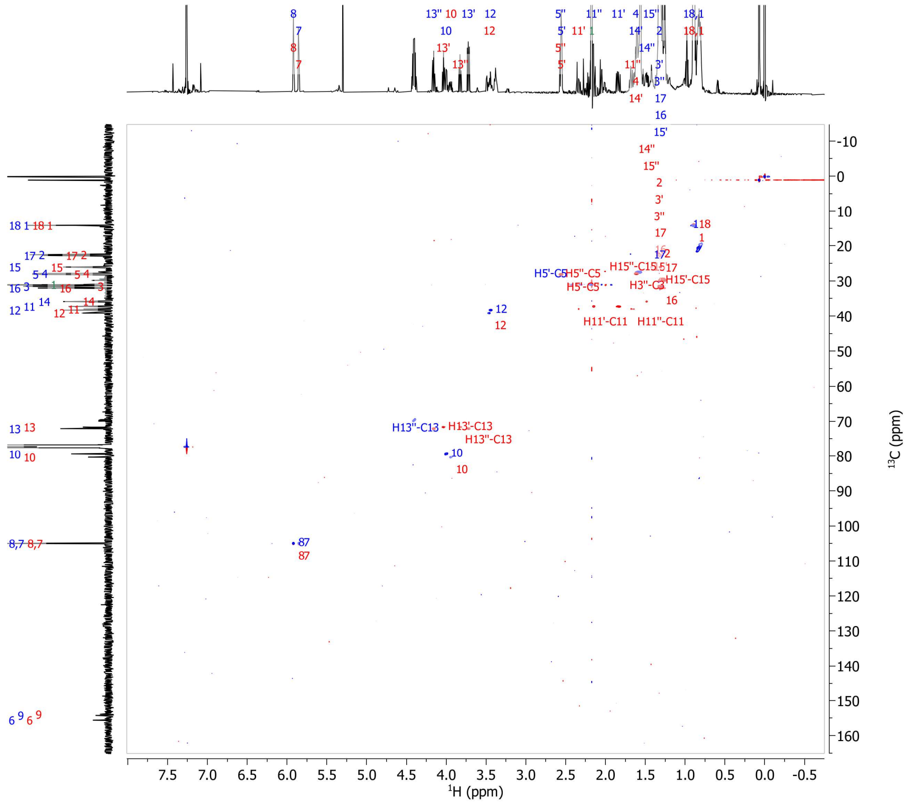

^{1}H-^{1}H-COSY (600 MHz, $CDCl_3$)

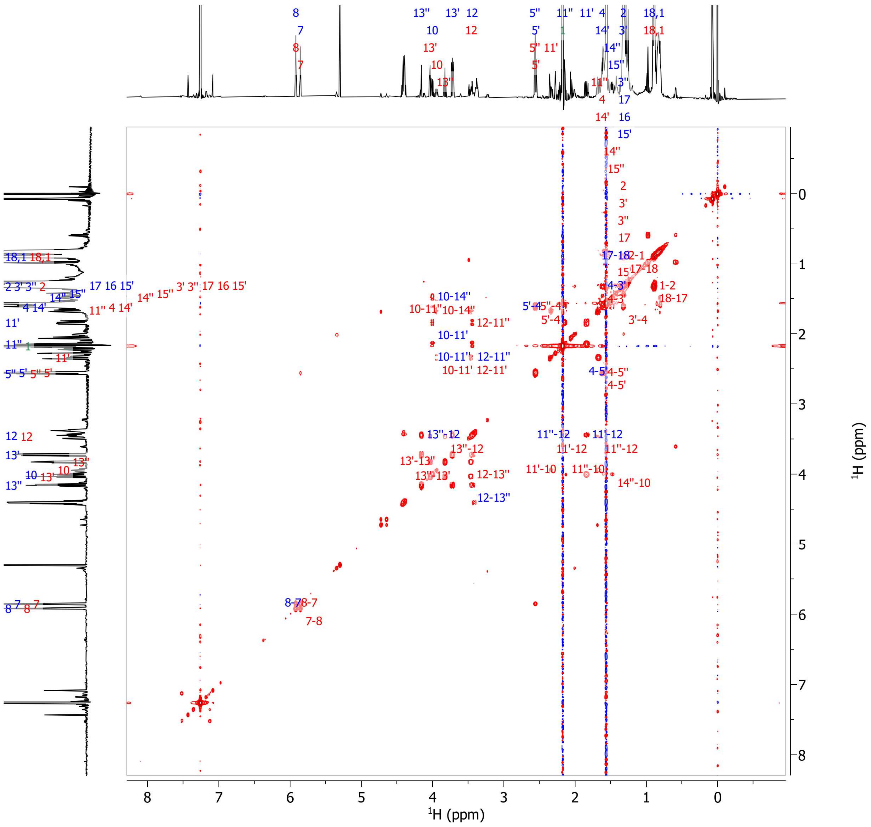

^{1}H-^{13}C-HMBC (600 MHz, 151 MHz, $CDCl_3$)

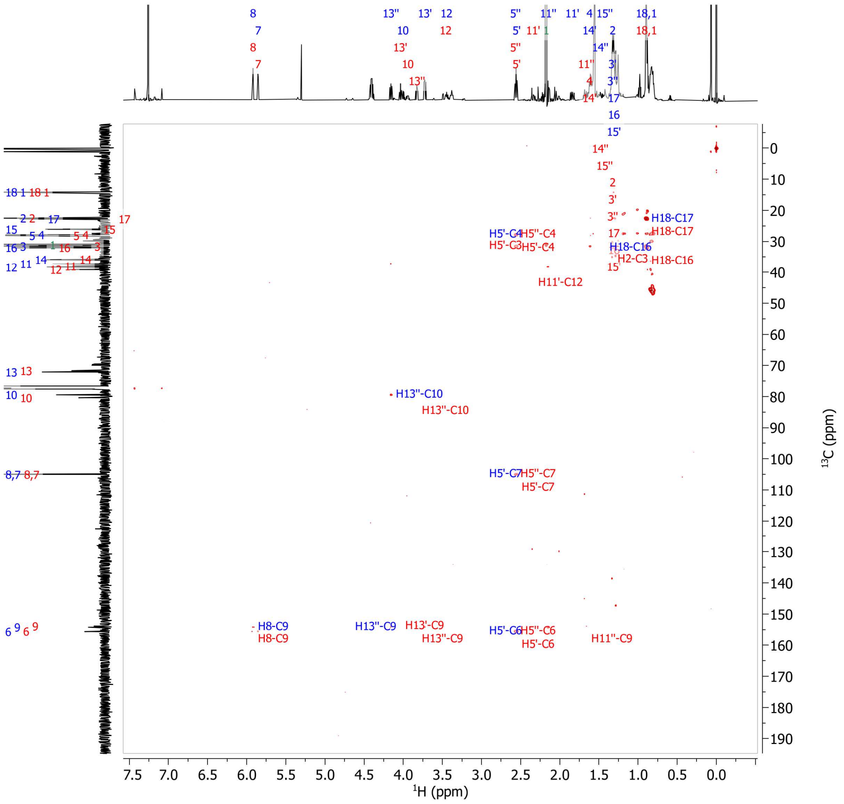

Spectra for Deuteration Studies

The ^{1}H and ^{2}D-NMR spectra of the crude reaction mixtures of the deuteration experiments are depicted below:

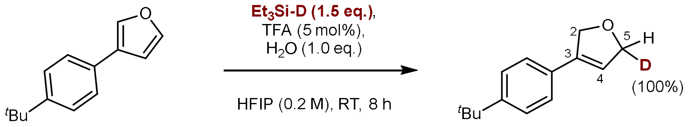

^{1}H NMR (600 MHz, $CDCl_3$)

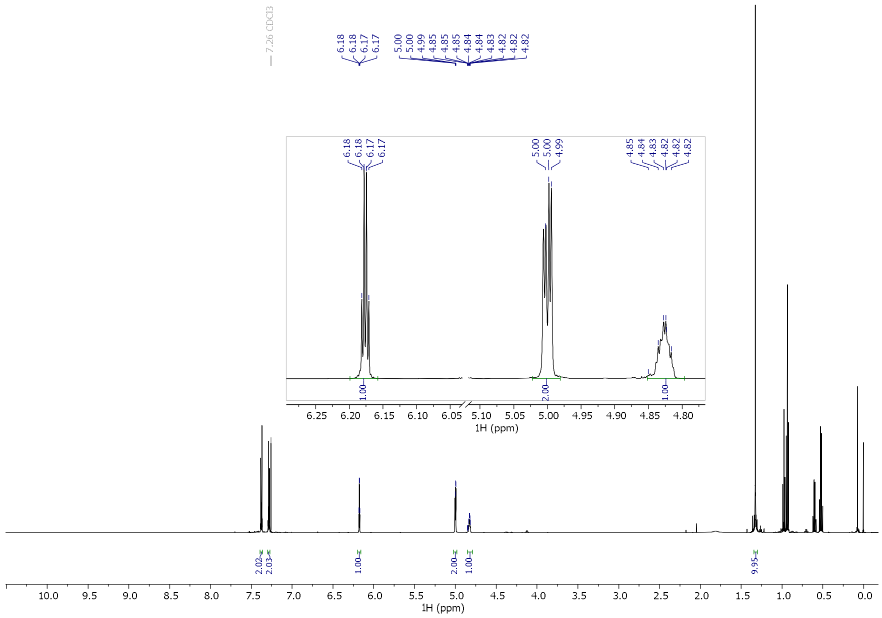

^{2}H NMR (92 MHz, $CDCl_3$)

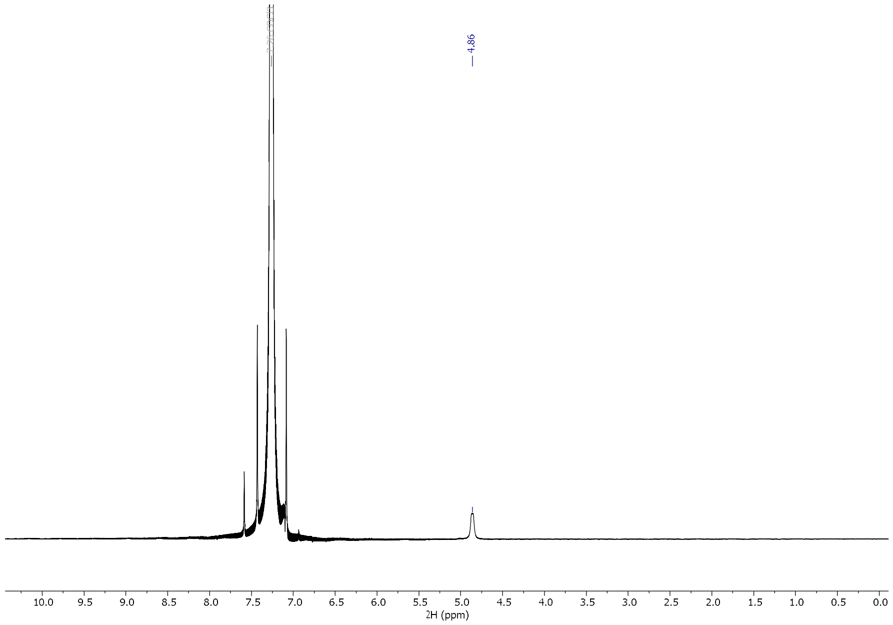

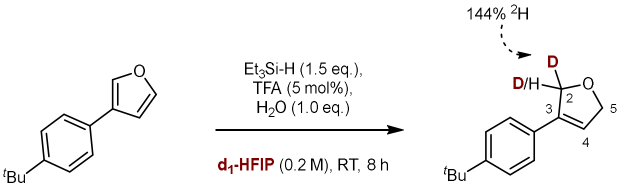

^{1}H NMR (600 MHz, $CDCl_3$)

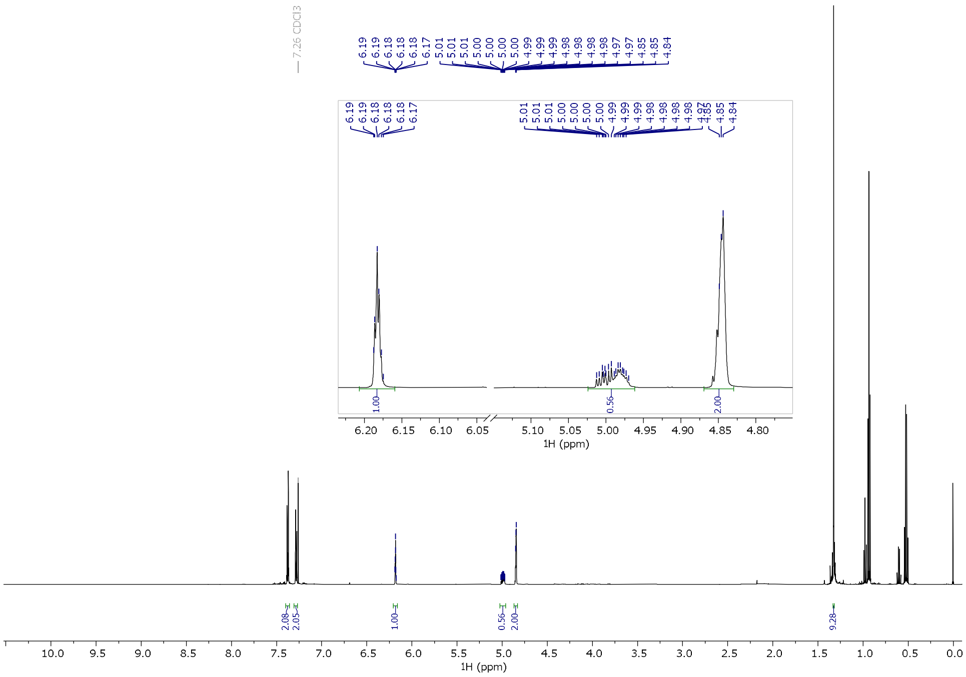

^{2}H NMR (92 MHz, $CDCl_3$)

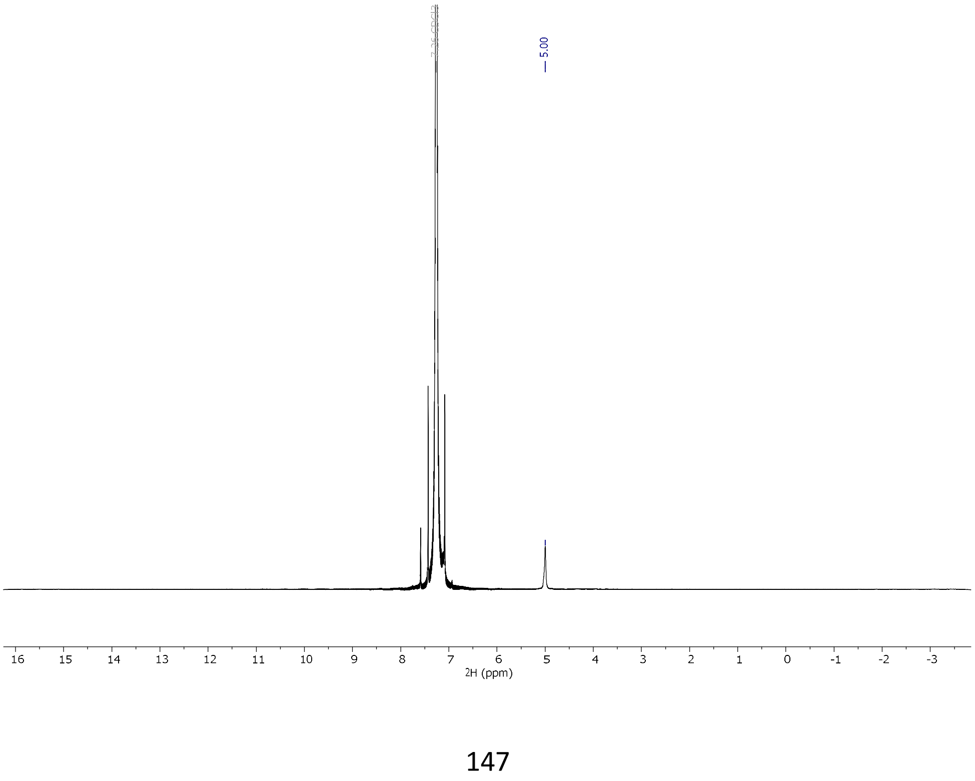

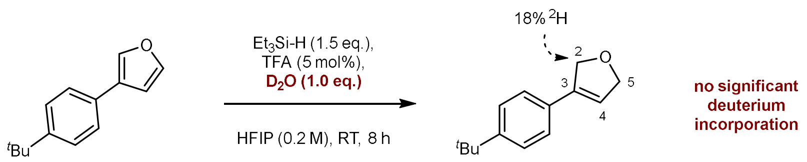

^{1}H NMR (600 MHz, CDCl$_3$)

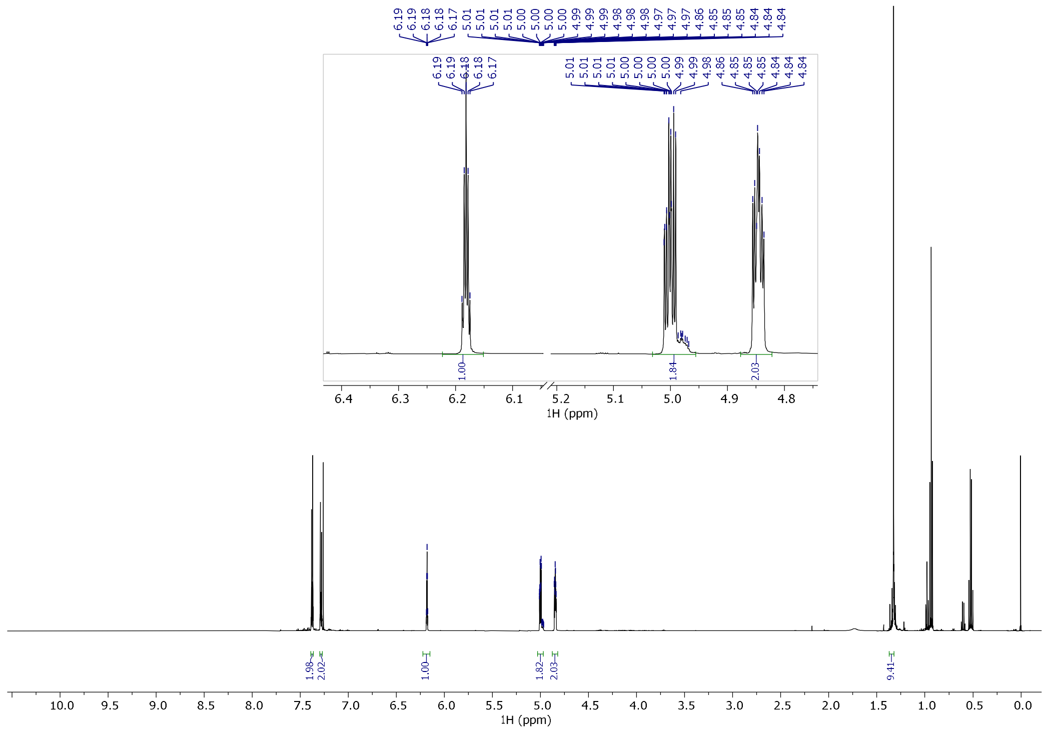

^{2}H NMR (92 MHz, CDCl$_3$)

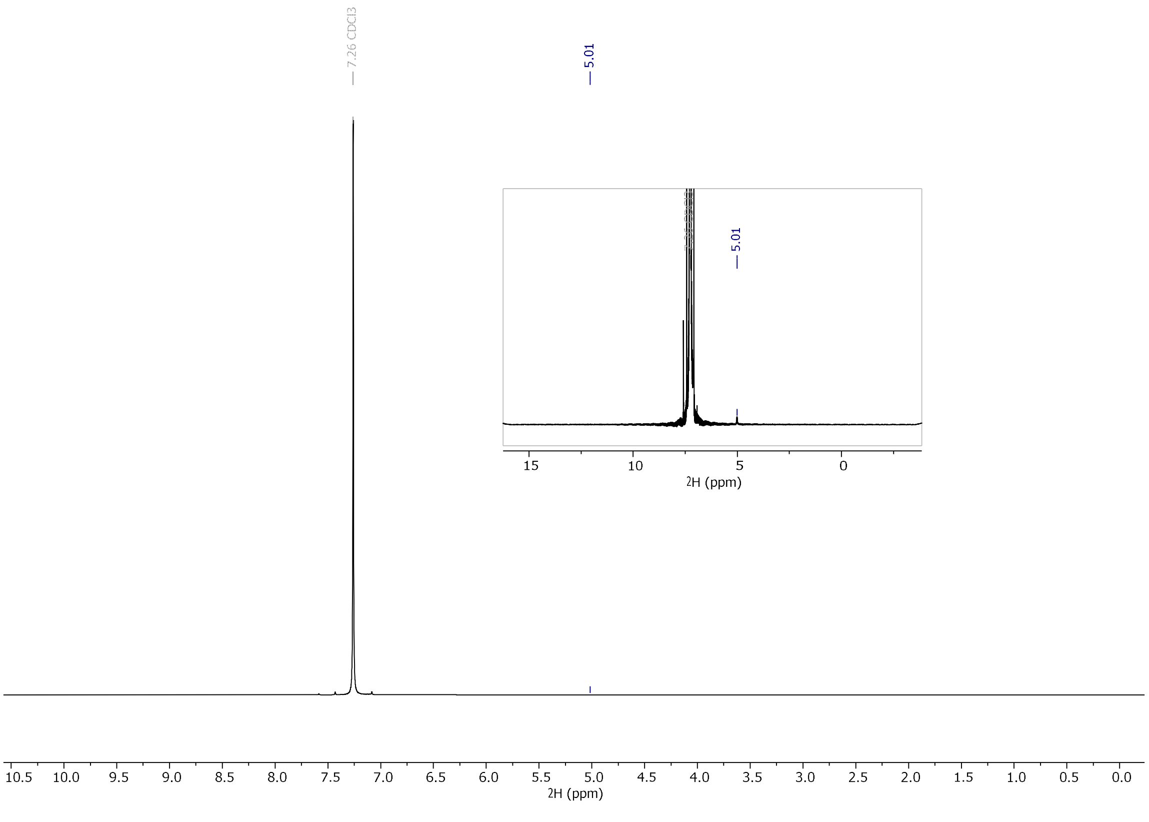

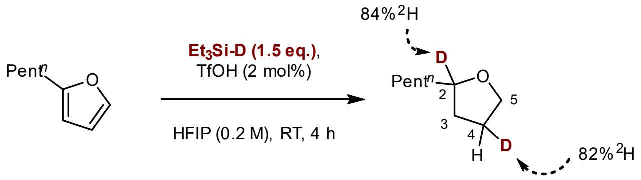

^{1}H NMR (600 MHz, $CDCl_3$)

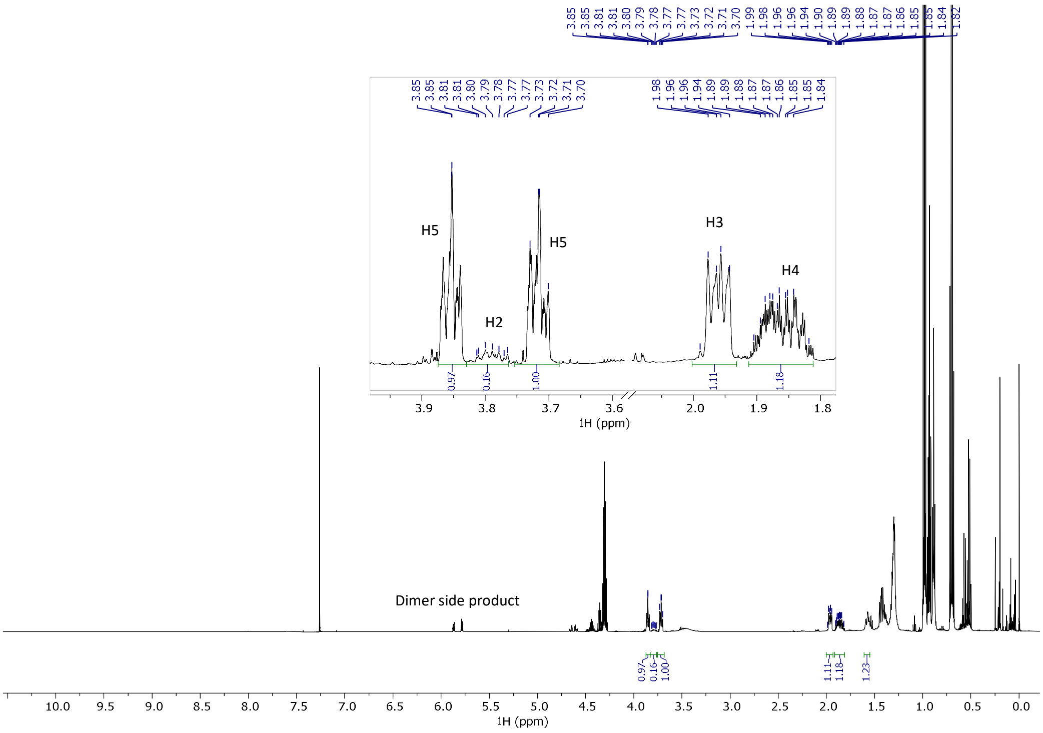

^{2}H NMR (92 MHz, $CDCl_3$)

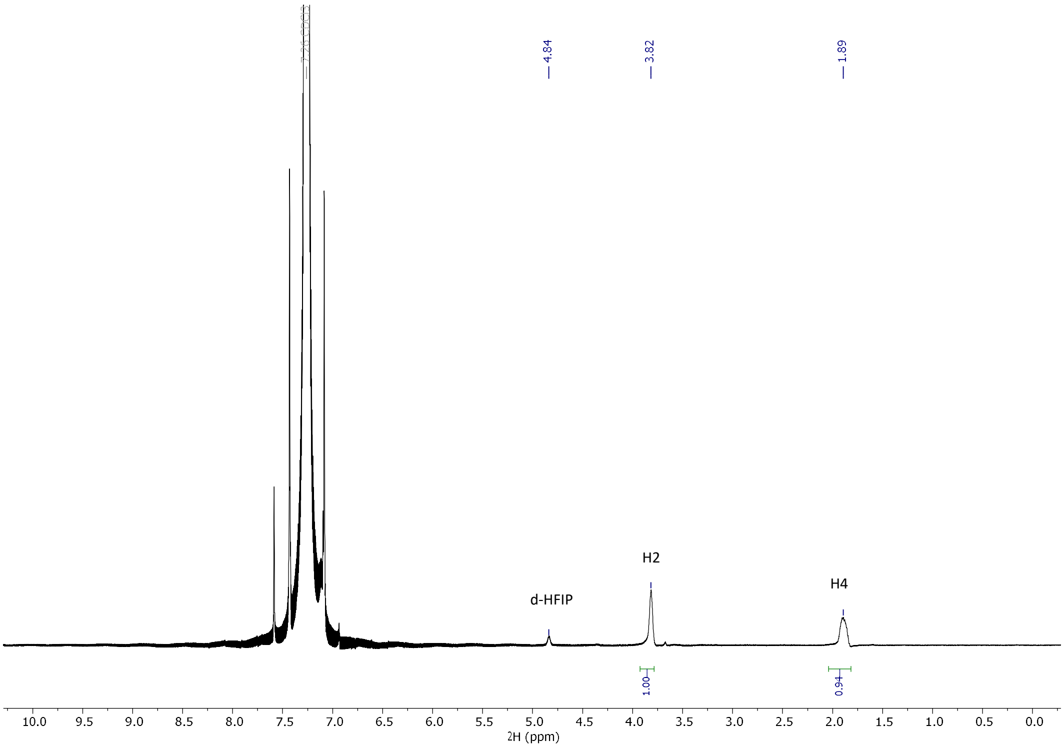

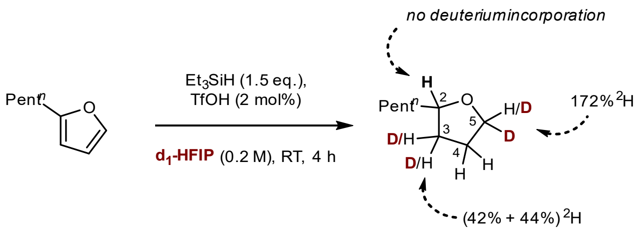

^{1}H NMR (600 MHz, $CDCl_3$)

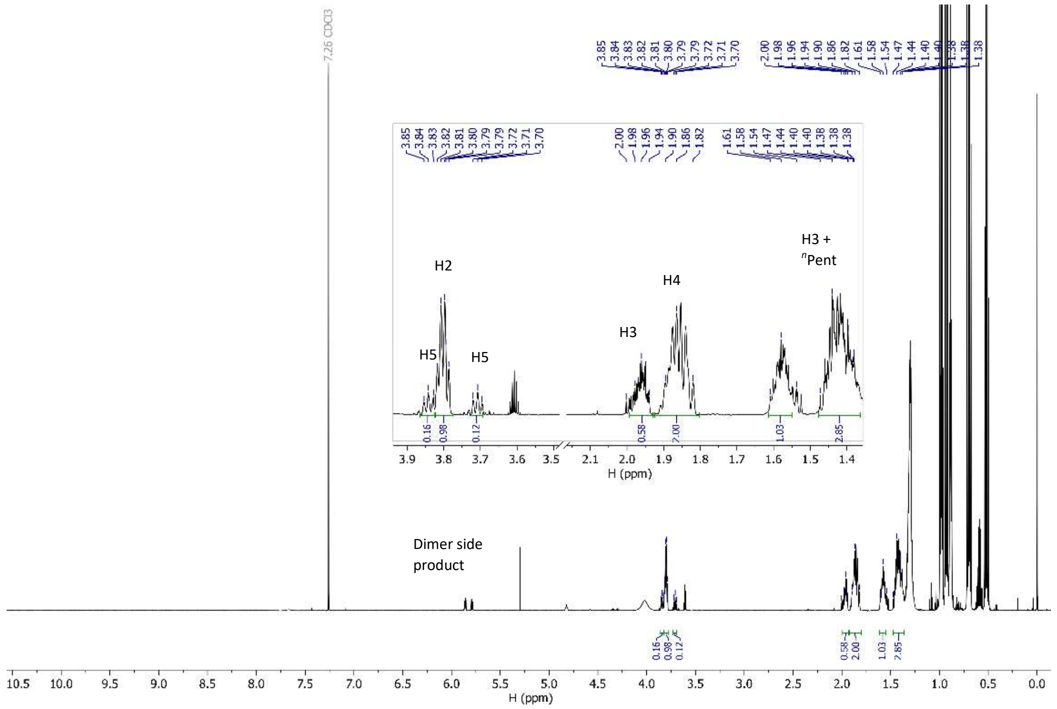

^{2}H NMR (92 MHz, $CDCl_3$)

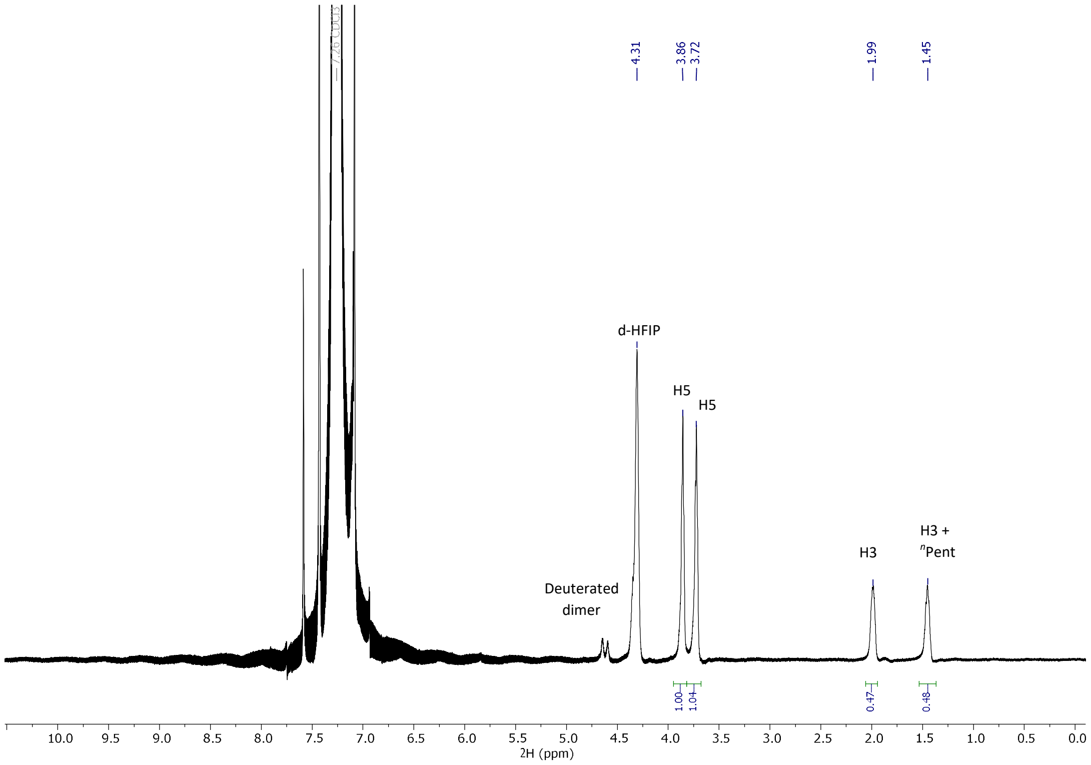

Note: H3 protons appear in two set of multiplets (1.94–2.00 ppm and 1.38–1.47 ppm overlapping with ^{n}Pent protons). Deuteration at this position is facially unselective. The deuteration degree of H3 was determined by a combined approach of ^{1}H NMR (for 1.94–2.00 ppm, H3) and relative integrals in the ^{2}H NMR (for 1.38–1.47 ppm; as overlap makes integration difficult in ^{1}H NMR).

Computational Discussion of 2D-Plot and Isomeric Analysis

Note: Not all protonation and hydride-reduction sites were considered if they were already excluded by our previous experiments, e.g. if 2,5-dihydro intermediates were observed experimentally the competing pathway over the 2,3-dihydrofuran was not considered computationally. Computations were performed at CPCM(HFIP)-DLPNO-CCSD(T)/def2-TZVPP//CPCM(HFIP)-B2PLYP-D3BJ/def2-SVP (298 K / 1 M) level of theory and associated data can be found in Table S9 and the Coordinates.zip data archieve.

3-Phenylfuran

Protonation at the C2 position is slightly preferred over the C2 and C4 positions and significantly preferred over the C4 position.

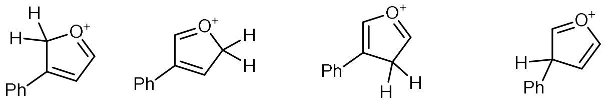

2 **0.0** | 2_2 **+5.01 kcal mol^{-1}** | 2_3 **+13.8 kcal mol^{-1}** | 2_4 **+20.0 kcal mol^{-1}**

The 2-position is similarly preferred for the protonation of 3-(4-methoxyphenyl)furan and 3-(4-nitrophenyl)furan although C3, C4 and C5 are less privileged.

3-Methylfuran

A similar thermodynamic situation was found for the protonated 3-methylfuran, although protonation at the 3- and 4-position is not feasible. Interestingly, the 4,5-dihydrofuran intermediate IM2_2 is thermodynamically most stable and not the experimentally obtained 2,5-dihydrofuran.

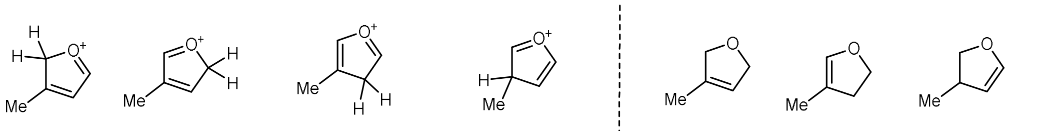

2 **0.0** | 2_2 **+3.2 kcal mol^{-1}** | 2_3 **+12.9 kcal mol^{-1}** | 2_4 **+17.4 kcal mol^{-1}** | IM2 **0.0** | IM2_2 **−1.4 kcal mol^{-1}** | IM2_3 **+0.12 kcal mol^{-1}**

Furan

Compared to the 3-aryl substituted furans, C2 protonation is much more preferred than C3 protonation by 9.6 kcal mol^{-1}. Interestingly, the 2,3-dihydrofuran is thermodynamically more stable than the experimentally observed 2,5-dihydrofuran by 3.1 kcal mol^{-1}. This hints towards a kinetic differentiation in the here developed methodology.

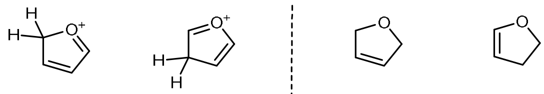

2 **0.0** | 2_2 **+9.6 kcal mol^{-1}** | IM2 **0.0** | IM2_2 **−3.1 kcal mol^{-1}**

2-Phenylfuran

Protonation of 2-Phenylfuran is only privileged on the C2 position. The 4,5-dihydrofuran **IM2** is 5.4 kcal mol^{-1} more stable than the 2,5-dihydrofuran **IM_2**.

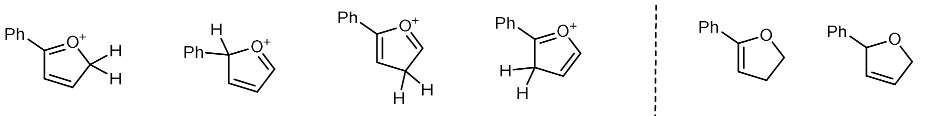

2	2_2	2_3	2_4	IM2	IM2_2
**0.0**	**+12.0 kcal mol^{-1}**	**+16.7 kcal mol^{-1}**	**+8.2 kcal mol^{-1}**	**0.0**	**+5.4 kcal mol^{-1}**

2- Methylesterfuran

Protonation of 2-Methylester furan is most privileged on the C2 position. As the protonation of these electron-poor furans were found to be thermodynamically unfavoured compared to the reference 3-phenylfuran, the second protonation stage was not computationally investigated.

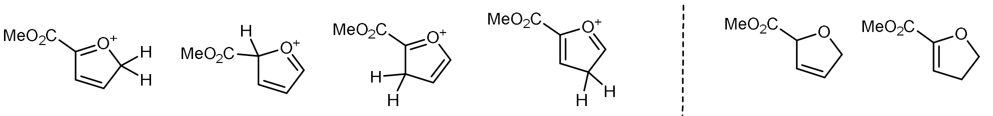

2	2_2	2_3	2_4	IM2	IM2_2
**0.0**	**+0.86 kcal mol^{-1}**	**+10.8 kcal mol^{-1}**	**+6.7 kcal mol^{-1}**	**0.0**	**−2.8 kcal mol^{-1}**

3-Methylesterfuran

Protonation of 3-Methylester furan is most privileged on the C2 position. As the protonation of these electron-poor furans were found to be thermodynamically unfavoured compared to the reference 3-phenylfuran, the second protonation stage was not computationally investigated.

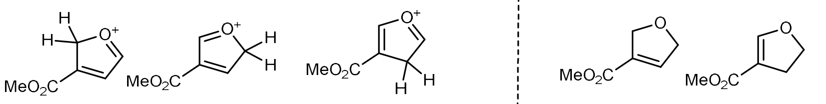

2	2_2	2_3	IM2	IM2_2
**0.0**	**+1.4 kcal mol^{-1}**	**+9.3 kcal mol^{-1}**	**0.0**	**−4.2 kcal mol^{-1}**

2-Ester 3-Methylfuran

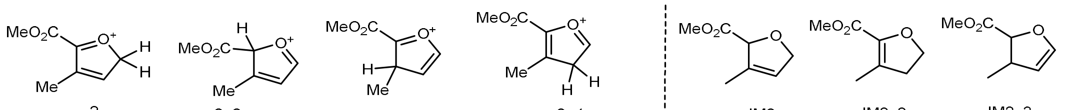

2 **0.0** — 2_2 **+0.86 kcal mol^{-1}** — 2_3 **+10.8 kcal mol^{-1}** — 2_4 **+6.7 kcal mol^{-1}** | IM2 **0.0** — IM2_2 **−1.5 kcal mol^{-1}** — IM2_3 **+0.60 kcal mol^{-1}**

Stage II:

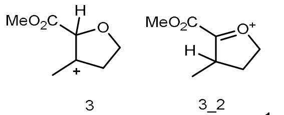

3 **0.0** — 3_2 **−18.2 kcal mol^{-1}**

2,3-Dimethylfuran

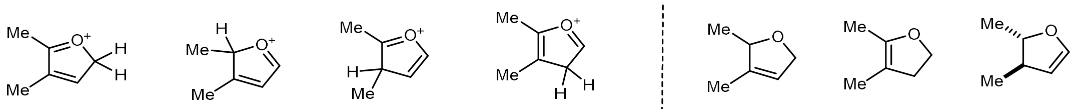

2 **0.0** — 2_2 **+4.83 kcal mol^{-1}** — 2_3 **+13.4 kcal mol^{-1}** — 2_4 **+14.6 kcal mol^{-1}** | IM2 **0.0** — IM2_2 **−3.12 kcal mol^{-1}** — IM2_3 **−0.40 kcal mol^{-1}**

Stage II:

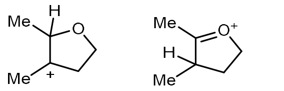

3 **0.0** — 3_2 **−29.5 kcal mol^{-1}**

Thermodynamic Data

Table S9. Thermodynamic quantities of the investigated reaction. Thermodynamic calculations were carried out at the CPCM(HFIP)-B2PLYP-D3BJ/def2-SVP level of theory (298 K / 1 M). Free energy G was calculated using the electronic energy of CPCM(HFIP)-DLPNO-CCSD(T)/def2-TZVPP//CPCM(MeCN)-B2PLYP-D3BJ/def2-SVP level of theory via $G = E_{el}(\text{DLPNO-CCSD(T)}) + [G(\text{B2PLYP-D3BJ}) - E_{el}(\text{B2PLYP-D3BJ})]$. Numerations **2_2**, **2_3**, etc. refer to different isomers. **Product** and **Product_dia** refer to two different diasteromers.

	**E_{el} B2PLYP-D3BJ/def2-SVP [Ha]**	**H B2PLYP-D3BJ/ def2-SVP [Ha]**	**G B2PLYP-D3BJ/ def2-SVP [Ha]**	**E_{el} DLPNO-CCSD(T)/ def2-TVPP [Ha]**	**Free Energy G**	**Relative G between isomers**
**3-Methyl-Furan**						
**SM**	-268.870377	-268.765990	-268.797922	-268.885222	-168682.5941	
**2**	-269.288876	-269.172232	-269.205071	-269.302352	-168937.2252	0
**2_2**	-269.282575	-269.165871	-269.198629	-269.297268	-168933.9460	3.27917397
**2_3**	-269.265546	-269.150111	-269.182640	-269.280869	-168924.3081	12.9171109
**2_4**	-269.257569	-269.141519	-269.173723	-269.274709	-168919.8532	17.3720158
**IM**	-270.055045	-269.927285	-269.961032	-270.082731	-169420.5139	0
**IM_2**	-270.059328	-269.931186	-269.964458	-270.085767	-169421.8819	-1.36803905
**IM_3**	-270.056158	-269.927977	-269.960759	-270.083918	-169420.3899	0.12397556
**3**	-270.451196	-270.313645	-270.347574	-270.475248	-169660.7925	
**Product**	-271.274263	-271.121555	-271.155423	-271.309285	-170174.6098	
**Furane**						
**SM**	-229.626709	-229.551770	-229.579000	-229.641887	-144072.5525	
**2**	-230.035207	-229.948010	-229.976470	-230.050410	-144321.9852	0
**2_2**	-230.016866	-229.931044	-229.959755	-230.033544	-144312.4212	9.56392275
**IM**	-230.808672	-230.710366	-230.739544	-230.836831	-144808.9510	0
**IM_2**	-230.814575	-230.715899	-230.745528	-230.841662	-144812.0336	-3.08261804
**3**	-231.186281	-231.078863	-231.109370	-231.211101	-145038.9248	
**Product**	-232.032266	-231.908998	-231.938899	-232.066730	-145565.5140	
**2-Methylester-Furan**						
**SM**	-457.139624	-457.016967	-457.055813	-457.195250	-286841.8200	
**2**	-457.533287	-457.39888	-457.439651	-457.587885	-287082.0376	0
**2_2**	-457.531007	-457.396154	-457.43578	-457.588106	-287081.1769	0.8606836
**2_3**	-457.514099	-457.381158	-457.421188	-457.569916	-287071.2162	10.8213803
**2_4**	-457.520003	-457.386685	-457.426484	-457.577077	-287075.3286	6.70902096
**IM**	-458.317507	-458.17150	-458.21197	-458.388975	-287577.2601	
**IM2**	-458.325531	-458.179326	-458.2198	-458.393568	-287580.0208	-2.76072432
**3**	-458.684586	-458.529425	-458.570551	-458.750857	-287799.0117	
**Product**	-459.542298	-459.371447	-459.412387	-459.619808	-288334.3252	

**3-Methyl-2-Methylester-Furan**						
**SM**	-496.385019	-496.232877	-496.275518	-496.439306	-311451.7212	
**2**	-496.781572	-496.617590	-496.660806	-496.834910	-311692.8975	0
**2_2**	-496.786953	-496.622661	-496.665742	-496.841300	-311696.6285	-3.73096993
**2_3**	-496.755818	-496.592467	-496.635872	-496.811600	-311678.7847	14.1127846
**2_4**	-496.771067	-496.608130	-496.651629	-496.825912	-311688.0846	4.81292666
**IM**	-497.565303	-497.389734	-497.433453	-497.635734	-312188.4669	0
**IM2**	-497.571646	-497.395970	-497.439934	-497.638055	-312190.0101	-1.54322804
**IM3**	-497.564718	-497.389086	-497.432104	-497.635535	-312187.8629	0.6039557
**3**	-497.952040	-497.766872	-497.811115	-498.016289	-312421.5742	0
**3_2**	-497.983629	-497.794566	-497.838622	-498.049338	-312439.7512	-18.1770287
**Product**	-498.784814	-498.584328	-498.628068	-498.862386	-312942.5807	0
**Product_dia**	-498.784041	-498.583692	-498.627502	-498.862569	-312942.8252	-0.24450699

**3-Phenyl-Furan**						
**SM**	-460.283669	-460.122686	-460.162149	-460.265817	-288744.9676	
**2**	-460.703706	-460.529873	-460.569669	-460.681886	-288998.2000	0
**2_2**	-460.691630	-460.518287	-460.558169	-460.673332	-288993.1942	5.00578845
**2_3**	-460.675736	-460.503646	-460.543860	-460.657763	-288984.4194	13.7805687
**2_4**	-460.662880	-460.490535	-460.530774	-460.648136	-288978.2336	19.9664044
**IM**	-461.467999	-461.283451	-461.324950	-461.463060	-289482.7393	0
**3**	-461.877046	-461.680621	-461.721850	-461.867337	-289728.8048	
**Product**	-462.684029	-462.474584	-462.515732	-462.687165	-290235.0336	

**3-(*p*-NO_2-Phenyl)-Furan**						
**SM**	-664.475146	-664.309332	-664.355074	-664.505074	-416907.9717	
**2**	-664.886704	-664.708297	-664.754369	-664.914452	-417157.1657	0
**2_2**	-664.878030	-664.699974	-664.746014	-664.908183	-417153.4319	3.73376885
**2_3**	-664.862267	-664.685440	-664.731987	-664.892952	-417144.9636	12.2021332
**2_4**	-664.849198	-664.672069	-664.718760	-664.882891	-417138.5515	18.6141942
**IM**	-665.658929	-665.469605	-665.517341	-665.701896	-417645.4881	0
**IM_2**	-665.658593	-665.46909	-665.515965	-665.702514	-417645.2231	0.26494694
**IM_3**	-665.668733	-665.478899	-665.526147	-665.708108	-417648.7601	-3.27204196
**3**	-666.053652	-665.853224	-665.901155	-666.093219	-417884.2012	
**Product**	-666.876076	-666.661853	-666.709442	-666.927142	-418398.6248	

3-(*p*-OMe-Phenyl)-Furan						
**SM**	-574.616416	-574.420191	-574.465370	-574.623738	-360487.1332	
**2**	-575.043602	-574.834382	-574.879849	-575.046084	-360744.1854	0
**2_2**	-575.026375	-574.817830	-574.863887	-575.032689	-360736.5747	7.61072276
**2_3**	-575.011012	-574.803627	-574.849415	-575.016972	-360727.271	16.9144755
**2_4**	-574.996999	-574.789344	-574.835097	-575.007207	-360720.952	23.2334556
**IM**	-575.801455	-575.581649	-575.628763	-575.821646	-361225.2498	0
**IM_2**	-575.799575	-575.579687	-575.626092	-575.820882	-361224.2741	0.97575102
**IM_3**	-575.807343	-575.587237	-575.633957	-575.825350	-361227.1381	-1.88826866
**3**	-576.225295	-575.992785	-576.039545	-576.239899	-361479.5134	
**Product**	-577.016311	-576.771674	-576.818584	-577.044665	-361976.9962	

2,3-Dimethyl-Furan						
**SM**	-308.117522	-307.983785	-308.019716	-308.132048	-193294.4467	
**2**	-308.542017	-308.396123	-308.432466	-308.556256	-193553.2711	0
**2_2**	-308.536214	-308.389613	-308.425206	-308.550016	-193548.4411	4.82997728
**2_3**	-308.518511	-308.373092	-308.408961	-308.534924	-193539.8857	13.3854115
**2_4**	-308.516937	-308.372032	-308.408366	-308.531952	-193538.6353	14.6357795
**IM**	-309.301334	-309.144056	-309.180481	-309.329585	-194031.4501	0
**IM_2**	-309.307907	-309.150382	-309.187259	-309.334358	-194034.5732	-3.12311872
**IM_3**	-309.302739	-309.145260	-309.181023	-309.331089	-194031.8520	-0.40183527
**3**	-309.698310	-309.530982	-309.568058	-309.722549	-194272.1411	0
**3_2**	-309.749085	-309.578390	-309.614958	-309.773458	-194301.6554	-29.5143068
**Product**	-310.520517	-310.338487	-310.375262	-310.556375	-194785.9604	0
**Product_dia**	-310.518523	-310.336289	-310.372554	-310.554369	-194784.2533	1.70708295

2-Ph-Furan						
**SM**	-460.288047	-460.127179	-460.166665	-460.269079	-288747.1010	
**2**	-460.708401	-460.53485	-460.574731	-460.688601	-289002.6442	0
**2_2**	-460.685731	-460.512398	-460.552276	-460.669238	-288990.6287	12.0154141
**2_3**	-460.677495	-460.505607	-460.545814	-460.659965	-288985.923	16.7211945
**2_4**	-460.693401	-460.521039	-460.561035	-460.674196	-288994.4232	8.22099757
**IM**	-461.476134	-461.291475	-461.332417	-461.469382	-289486.2869	0
**IM_2**	-461.463834	-461.279709	-461.320613	-461.460263	-289480.8758	5.41105567
**3**	-461.921749	-461.723647	-461.764555	-461.91077	-289754.8058	
**Product**	-462.687427	-462.478434	-462.519594	-462.690267	-290237.2713	

3-Methylester-Furan						
**SM**	-457.127704	-457.004927	-457.043997	-457.184292	-286835.0087	
**2**	-457.525121	-457.390477	-457.430596	-457.581454	-287077.4434	0
**2_2**	-457.523191	-457.388588	-457.428098	-457.579757	-287076.0217	1.42168867
**2_3**	-457.508283	-457.374756	-457.41447	-457.565867	-287068.1087	9.33466321
**IM1**	-458.310075	-458.163898	-458.204403	-458.379735	-287571.3778	0

**IM2**	-458.310113	-458.163904	-458.204408	-458.379688	-287571.3275	0.05033873
**IM3**	-458.320644	-458.173789	-458.21387	-458.387562	-287575.5980	-4.22022512
**3_1**	-458.735517	-458.576042	-458.616932	-458.803684	-287829.3069	
**3_2**	-458.738568	-458.579395	-458.620423	-458.806488	-287831.3418	
**3_3**	-458.679182	-458.523289	-458.565282	-458.743388	-287794.4101	
**Product**	-459.541736	-459.37064	-459.413081	-459.619762	-288335.0843	
**2-(*p*-OMe-Ph)-Furan**						
**SM**	-574.62101	-574.424883	-574.470465	-574.62707	-360489.5392	
**2**	-575.049175	-574.840226	-574.886075	-575.052724	-360748.7629	
**2_2**	not calculated					
**IM**	-575.809412	-575.589527	-575.636507	-575.827591	-361228.8467	0
**IM2**	-575.796634	-575.577257	-575.62439	-575.818550	-361223.5876	5.25907121
**IM3**	-575.809424	-575.589559	-575.636515	-575.827591	-361228.8434	0.0032202
**3**	-576.263378	-576.029829	-576.076557	-576.275378	-361501.1041	
**3_Isomer**	not calculated					
**Product**	-577.019966	-576.775759	-576.822762	-577.048074	-361979.4637	
**2-(*p*-CN-Ph)-Furan**						
**SM**	-552.390524	-552.229457	-552.273473	-552.369944	-346543.9964	
**2**	-552.802663	-552.629248	-552.673794	-552.781387	-346794.7650	
**2_2**	not calculated					
**IM**	-553.578210	-553.393422	-553.438948	-553.570134	-347283.1892	0
**IM_2**	-553.566258	-553.381854	-553.427452	-553.561382	-347277.9840	5.20516513
**IM_3**	-553.570562	-553.386017	-553.431485	-553.56494	-347280.0462	3.14298127
**3**	-554.015292	-553.817284	-553.862741	-554.00327	-347546.6482	
**3_Isomer**	not calculated					
**Product**	-554.790294	-554.581194	-554.626623	-554.791974	-348034.5884	
**2,3-dihydro**	-427.020511	-426.772474	-426.818587	-427.050568	-426.8486444	
**2,5-dihydro**	-427.011206	-426.763609	-426.809774	-427.043294	-426.841862	4.25600307

15. References

(1) Dunlop, A. P.; Peters, F. N. *The Furans*; Reinhold Publishing Corporation, 1953.

(2) Hansch, C.; Leo, A.; Taft, R. W. A Survey of Hammett Substituent Constants and Resonance and Field Parameters. *Chem. Rev.* 1991, *91* (2), 165–195. https://doi.org/10.1021/cr00002a004.

(3) Hoye, T. R.; Eklov, B. M.; Ryba, T. D.; Voloshin, M.; Yao, L. J. No-D NMR (No-Deuterium Proton NMR) Spectroscopy: A Simple Yet Powerful Method for Analyzing Reaction and Reagent Solutions. *Org. Lett.* 2004, *6* (6), 953–956. https://doi.org/10.1021/ol049979+.

(4) Burés, J. Variable Time Normalization Analysis: General Graphical Elucidation of Reaction Orders from Concentration Profiles. *Angew. Chem. Int. Ed.* 2016, *55* (52), 16084–16087. https://doi.org/10.1002/anie.201609757.

(5) Monaco, M. R.; Poladura, B.; Bernardos, M. D. de L.; Leutzsch, M.; Goddard, R.; List, B. Activation of Carboxylic Acids in Asymmetric Organocatalysis. *Angew. Chem. Int. Ed.* 2014, *53* (27), 7063–7067. https://doi.org/10.1002/anie.201400169.

(6) Larsen, C. H.; Ridgway, B. H.; Shaw, J. T.; Woerpel, K. A. A Stereoelectronic Model To Explain the Highly Stereoselective Reactions of Nucleophiles with Five-Membered-Ring Oxocarbenium Ions. *J. Am. Chem. Soc.* 1999, *121* (51), 12208–12209. https://doi.org/10.1021/ja993349z.

(7) Rijssel, E. R. van; Delft, P. van; Lodder, G.; Overkleeft, H. S.; Marel, G. A. van der; Filippov, D. V.; Codée, J. D. C. Furanosyl Oxocarbenium Ion Stability and Stereoselectivity. *Angew. Chem. Int. Ed.* 2014, *53* (39), 10381–10385. https://doi.org/10.1002/anie.201405477.

(8) Neese, F. Software Update: The ORCA Program System—Version 5.0. *Wiley Interdiscip. Rev.: Comput. Mol. Sci.* 2022, *12* (5). https://doi.org/10.1002/wcms.1606.

(9) Neese, F. The ORCA Program System. *Wiley Interdiscip. Rev.: Comput. Mol. Sci.* 2012, *2* (1), 73–78. https://doi.org/10.1002/wcms.81.

(10) Neese, F.; Wennmohs, F.; Hansen, A.; Becker, U. Efficient, Approximate and Parallel Hartree–Fock and Hybrid DFT Calculations. A 'Chain-of-Spheres' Algorithm for the Hartree–Fock Exchange. *Chem. Phys.* 2009, *356* (1–3), 98–109. https://doi.org/10.1016/j.chemphys.2008.10.036.

(11) Stoychev, G. L.; Auer, A. A.; Neese, F. Automatic Generation of Auxiliary Basis Sets. *J. Chem. Theory Comput.* 2017, *13* (2), 554–562. https://doi.org/10.1021/acs.jctc.6b01041.

(12) Pracht, P.; Bohle, F.; Grimme, S. Automated Exploration of the Low-Energy Chemical Space with Fast Quantum Chemical Methods. *Phys. Chem. Chem. Phys.* 2020, *22* (14), 7169–7192. https://doi.org/10.1039/c9cp06869d.

(13) Bannwarth, C.; Ehlert, S.; Grimme, S. GFN2-XTB—An Accurate and Broadly Parametrized Self-Consistent Tight-Binding Quantum Chemical Method with Multipole Electrostatics and Density-Dependent Dispersion Contributions. *J. Chem. Theory Comput.* 2019, *15* (3), 1652–1671. https://doi.org/10.1021/acs.jctc.8b01176.

(14) Grimme, S. Semiempirical Hybrid Density Functional with Perturbative Second-Order Correlation. *J. Chem. Phys.* 2006, *124* (3), 034108. https://doi.org/10.1063/1.2148954.

(15) Penocchio, E.; Piccardo, M.; Barone, V. Semiexperimental Equilibrium Structures for Building Blocks of Organic and Biological Molecules: The B2PLYP Route. *J. Chem. Theory Comput.* 2015, *11* (10), 4689–4707. https://doi.org/10.1021/acs.jctc.5b00622.

(16) Barone, V.; Cossi, M. Quantum Calculation of Molecular Energies and Energy Gradients in Solution by a Conductor Solvent Model. *J. Phys. Chem. A* 1998, *102* (11), 1995–2001. https://doi.org/10.1021/jp9716997.

(17) Riplinger, C.; Neese, F. An Efficient and near Linear Scaling Pair Natural Orbital Based Local Coupled Cluster Method. *J. Chem. Phys.* 2013, *138* (3), 034106. https://doi.org/10.1063/1.4773581.

(18) Sandler, I.; Chen, J.; Taylor, M.; Sharma, S.; Ho, J. Accuracy of DLPNO-CCSD(T): Effect of Basis Set and System Size. *J. Phys. Chem. A* 2021, *125* (7), 1553–1563. https://doi.org/10.1021/acs.jpca.0c11270.

(19) Lorenz, U. J.; Lemaire, J.; Maitre, P.; Crestoni, M.-E.; Fornarini, S.; Dopfer, O. Protonation of Heterocyclic Aromatic Molecules: IR Signature of the Protonation Site of Furan and Pyrrole. *Int. J. Mass Spectrom.* 2007, *267* (1–3), 43–53. https://doi.org/10.1016/j.ijms.2007.02.017.

(20) Tanis, S. P. A Simple Synthesis of 3-Substituted Furans. The Preparations of Dendrolasin, Perillene and Congeners. *Tetrahedron Lett.* 1982, *23* (31), 3115–3118. https://doi.org/10.1016/s0040-4039(00)88573-1.

(21) New, D. G.; Tesfai, Z.; Moeller, K. D. Intramolecular Anodic Olefin Coupling Reactions and the Use of Electron-Rich Aryl Rings1. *J. Org. Chem.* 1996, *61* (5), 1578–1598. https://doi.org/10.1021/jo9518359.

(22) Sun, Y.-N.; Wang, Q.; He, L.; Wang, X.; Li, W.-D. Z. Syntheses of Perillene and Natural Congeners via Li2CuCl4-Catalyzed Cross-Coupling Reaction of Allylic Carbonates. *Tetrahedron Lett.* 2022, *90*, 153610. https://doi.org/10.1016/j.tetlet.2021.153610.

(23) Chen, V. Y.; Kwon, O. Unified Approach to Furan Natural Products via Phosphine-Palladium Catalysis. *Angew. Chem. Int. Ed.* 2021, *60* (16), 8874–8881. https://doi.org/10.1002/anie.202015232.

(24) Kumar, M.; Bromhead, L.; Anderson, Z.; Overy, A.; Burton, J. W. Short, Tin-Free Synthesis of All Three Inthomycins. *Chem. A Eur. J.* 2018, *24* (63), 16753–16756. https://doi.org/10.1002/chem.201803794.

(25) Nienałtowski, T.; Szczepanik, P.; Małecki, P.; Czajkowska-Szczykowska, D.; Czarnocki, S.; Pawłowska, J.; Kajetanowicz, A.; Grela, K. Large-Scale Synthesis of a Niche Olefin Metathesis Catalyst Bearing an Unsymmetrical N-Heterocyclic Carbene (NHC) Ligand and Its Application in a Green Pharmaceutical Context. *Chem. A Eur. J.* 2020, *26* (67), 15708–15717. https://doi.org/10.1002/chem.202003830.

(26) Hedenström, E.; Edlund, H.; Lund, S.; Abersten, M.; Persson, D. Synthesis and Lipase Catalysed Stereoselective Acylation of Some 3-Methylalkan-2-Ols, Identified as Sex Pheromone

Precursors in Females of Pine Sawfly Species. *J. Chem. Soc., Perkin Trans. 1* 2002, *0* (15), 1810–1817. https://doi.org/10.1039/b201395a.

(27) Arceo, E.; Ellman, J. A.; Bergman, R. G. Rhenium-Catalyzed Didehydroxylation of Vicinal Diols to Alkenes Using a Simple Alcohol as a Reducing Agent. *J. Am. Chem. Soc.* 2010, *132* (33), 11408–11409. https://doi.org/10.1021/ja103436v.

(28) Ji, P.; Feng, X.; Oliveres, P.; Li, Z.; Murakami, A.; Wang, C.; Lin, W. Strongly Lewis Acidic Metal–Organic Frameworks for Continuous Flow Catalysis. *J. Am. Chem. Soc.* 2019, *141* (37), 14878–14888. https://doi.org/10.1021/jacs.9b07891.

(29) Mihailović, M. Lj.; Mamuzić, R. I.; Žigić-Mamuzić, Lj.; Bošnjak, J.; Čeković, Ž. Assignment of Cis-Trans Configuration to Constitutionally Symmetrical 2,5-Dialkyl-Tetrahydrofurans. *Tetrahedron* 1967, *23* (1), 215–226. https://doi.org/10.1016/s0040-4020(01)83303-2.
